# Kresoxim-methyl primes *Medicago truncatula* plants against abiotic stress factors via altered reactive oxygen and nitrogen species signalling leading to downstream transcriptional and metabolic readjustment

**DOI:** 10.1093/jxb/erv516

**Published:** 2015-12-27

**Authors:** Panagiota Filippou, Chrystalla Antoniou, Toshihiro Obata, Katrien Van Der Kelen, Vaggelis Harokopos, Loukas Kanetis, Vassilis Aidinis, Frank Van Breusegem, Alisdair R Fernie, Vasileios Fotopoulos

**Affiliations:** ^1^Department of Agricultural Sciences, Biotechnology and Food Science, Cyprus University of Technology, PO Box 50329 Limassol, Cyprus; ^2^Max-Planck-Institut für Molekulare Pflanzenphysiologie, Am Mühlenberg 1, 14476 Potsdam-Golm, Germany; ^3^Institute of Immunology, Biomedical Sciences Research Center Alexander Fleming, 34 Fleming Street, 16672 Athens, Greece; ^4^Department of Plant Systems Biology, VIB, 9052 Gent, Belgium; ^5^Department of Plant Biotechnology and Bioinformatics, Ghent University, 9052 Gent, Belgium

**Keywords:** Drought, priming, reactive species, salinity, strobilurins, systems biology.

## Abstract

Biotic and abiotic stresses, such as fungal infection and drought, cause major yield losses in modern agriculture. Kresoxim-methyl (KM) belongs to the strobilurins, one of the most important classes of agricultural fungicides displaying a direct effect on several plant physiological and developmental processes. However, the impact of KM treatment on salt and drought stress tolerance is unknown. In this study we demonstrate that KM pre-treatment of *Medicago truncatula* plants results in increased protection to drought and salt stress. Foliar application with KM prior to stress imposition resulted in improvement of physiological parameters compared with stressed-only plants. This protective effect was further supported by increased proline biosynthesis, modified reactive oxygen and nitrogen species signalling, and attenuation of cellular damage. In addition, comprehensive transcriptome analysis identified a number of transcripts that are differentially accumulating in drought- and salinity-stressed plants (646 and 57, respectively) after KM pre-treatment compared with stressed plants with no KM pre-treatment. Metabolomic analysis suggests that the priming role of KM in drought- and to a lesser extent in salinity-stressed plants can be attributed to the regulation of key metabolites (including sugars and amino acids) resulting in protection against abiotic stress factors. Overall, the present study highlights the potential use of this commonly used fungicide as a priming agent against key abiotic stress conditions.

## Highlight

The fungicide kresoxim-methyl displays novel priming properties against key abiotic stress factors (drought and salinity) by modifying reactive oxygen and nitrogen species signalling, inducing osmoprotection through increased proline biosynthesis and suppressing proteolysis.

## Introduction

Drought and salinity are two of the most important abiotic stress factors limiting plant growth and crop productivity worldwide (Krasensky and Jonak, 2012), including leguminous crops such as *Medicago truncatula* (Filippou *et al.*, 2011, Mhadhbi *et al.*, 2011). In general, drought conditions cause osmotic stress (Osakabe *et al.*, 2013), whereas salt stress causes both osmotic and ionic stress (Zhang *et al.*, 2009), both leading to cell death under extreme conditions. Prior to that, detrimental effects occur including a deficiency in energy dissipation as a consequence of the stress-induced photosynthesis limitation and cellular damage produced by the accumulation of reactive oxygen species (ROS) leading to oxidative stress (Apel and Hirt, 2004; Ahmad *et al.*, 2010).

Similarly, reactive nitrogen species (RNS) have also emerged as key players in a plant’s response to a multitude of stresses such as salinity, drought and heavy metals (Corpas *et al.*, 2008, Molassiotis and Fotopoulos, 2011). Nitric oxide (NO), one of the main forms of RNS, can either have a toxic or protective effect against abiotic stress factors, as it additionally alleviates the deleterious effects of ROS (Beligni and Lamattina, 1999; Qiao and Fan, 2008). NO regulation is often associated with the regulation of the activity of the key enzyme of the nitrate assimilation pathway in higher plants, nitrate reductase (NR; EC 1.6.6.1.). Generally, NR enzymatic activity in plant tissues is subjected to complex regulation in response to different environmental stimuli and it has been shown to be modified by both salinity (Reda *et al.*, 2011) and drought (Fresneau *et al.*, 2007) stresses.

The physiological mechanisms governing plant responses to salinity and drought imply that both stresses are perceived by the plant cell as deprivation of water (Jakab *et al.*, 2005). The defence response of the plant to these conditions is the reduction of stomatal conductance (Chaves *et al.*, 2009), the accumulation of compatible osmolytes, such as sugar alcohols, crucial amino acids (proline), and glycine-betaine (Ashraf and Foolad, 2007), as well as the expression of antioxidant defence genes, which are triggered to defend against cellular oxidative damage (Miller *et al.*, 2010). For instance, proline is important for protecting cells against ROS accumulation, thus proline accumulation under stress might occur due to an increase in Δ^1^-pyrroline-5-carboxylate synthase (p5CS), the rate-limiting enzyme in proline biosynthesis (Szabados and Savouré, 2009).

There are many strategies to overcome the negative effects of drought and salinity. Adaptation to stress has been suggested to be mediated by both pre-existing and induced defences (Hasegawa *et al.*, 2000; Pastori and Foyer, 2002). The selection of priming agents resulting in increased plant protection seems a good strategy. Seed priming techniques and hormonal priming have been used to induce drought tolerance in many field crops (Jisha *et al.*, 2013). Nowadays, boosting the plant’s internal defence mechanism to survive adverse environmental conditions is crucial for plant improvement. In this context, exogenous application of plant protective chemical compounds is a promising option.

Strobilurins belong to a group of agrochemical fungicides that exert their mode of action by blocking the electron transport in complex III of the mitochondrial respiratory chain and often stimulate the fungal mitochondrial alternative oxidase active in respiration (Sauter, 2007). They also trigger a positive effect on plant physiology and growth, likely through an interaction with electron transfer in plant mitochondria (Diaz-Espejo *et al.*, 2012) but the exact mechanism is still unknown. Higher yields and better cereal quality have been reported following strobilurin application (Bertelsen *et al.*, 2001), as well as higher photosynthetic activity of green tissues (Beck *et al.*, 2002), delayed senescence with enhanced concentrations of nitrogen and chlorophyll (Wu and von Tiedemann, 2001; Ruske *et al.*, 2003) and changes in hormonal status (Grossmann *et al.*, 1999).

The synthetic fungicide kresoxim-methyl [Methyl(*E*)-α-(methoxyimino)-2-(2-methylphenoxymethyl)phenylacetate] (KM) is a modification of the naturally occurring compound strobilurin A (Bartlett *et al.*, 2002). Like other strobilurins, this compound acts by blocking the fungal electron transfer at the cytochrome-bc complex of mitochondrial respiration (Ammermann *et al.*, 1992) and its application exhibits an increased plant biomass and better yield (Grossmann and Retzlaff, 1997). Here we assessed the effect of KM as a potential priming agent to prevent abiotic stress-caused penalties in plants. Therefore we examined the ameliorative effects of KM pre-treatments on *M. truncatula* plants subsequently exposed to drought and salinity conditions, two major global climate change-related abiotic stress factors limiting agricultural productivity worldwide. This was done by employing a multi-faceted performance analysis at the physiological, biochemical, molecular and metabolome level in order to identify the *modus operandi* of KM’s protective function under abiotic stress conditions.

## Materials and methods

### Plant material and treatments

Mature (40 d) *Medicago truncatula* ecotype Jemalong A17 plants were used in this study. Seeds were sown in sterile perlite:sand (1:3) pots and placed at 4^o^C for 4 d for stratification. Plants were grown in a growth chamber at 22/16°C day/night temperature, at 60–70% relative humidity (RH), with a photosynthetic photon flux density of 100 μmol m^2^ s^−1^ and a 16/8h photoperiod. Drought treatment was applied to 40-day-old plants by withholding water for 9 d (Filippou *et al.*, 2011), while salinity was imposed by watering plants with 200mM NaCl for 48h (Mhadhbi *et al.*, 2011). Control samples were water-treated in both cases. Plants were pre-treated prior to stress imposition by spraying with 10^–8^ M KM (Sigma-Aldrich, USA) dissolved in water. Optimal concentration of KM was determined based on preliminary analysis of a concentration gradient following assessment of cellular damage (lipid peroxidation) levels and physiological parameters in subsequently stressed plants (data not shown). Analyses were carried out using a minimum of three independent biological replicates in each experiment, with each replicate consisting of pooled samples from three independent plants.

### Physiological measurements

Stomatal conductance was measured using a ΔΤ-Porometer AP4 (Delta-T Devices-Cambridge) according to the manufacturer’s instructions.

### Lipid peroxidation

Lipid peroxidation was determined from the measurement of malondialdehyde (MDA) content resulting from the thiobarbituric acid (TBA) reaction (Minotti and Aust, 1987) using an extinction coefficient of 155mM^−1^ cm^−1^.

### Hydrogen peroxide and nitric oxide quantification

Hydrogen peroxide was quantified using the KI method, as described by Velikova *et al.* (2000). NO content was measured indirectly (nitrite-derived NO) using the Griess reagent in homogenates prepared in an ice-cold Na-acetate buffer (pH 3.6) as described by Zhou *et al.* (2005).

### Proline content

Free proline levels were determined using the ninhydrin reaction (Bates *et al.*, 1973) in addition to the findings obtained with GC-TOF-MS (Lisec *et al.*, 2006). Proline concentration was estimated from a proline standard curve.

### Enzymatic activity assays

#### p5CS

Plant cell extraction and p5CS activity measurements were processed according to Filippou *et al.* (2013). Leaves were homogenized in an extraction buffer (100mM Tris-Cl, pH 7.5, 10mM β-mercaptoethanol, 10mM MgCl_2_, 1mM PMSF) in pre-chilled eppendorf tubes on ice. Extracts were centrifuged at 4^o^C for 20min at 10 000 ×*g*. Supernatants were further clarified by centrifugation at 10 000 ×*g* for 20min at 4^o^C. p5CS enzymatic assay was carried out in 100mM Tris-Cl (pH 7.2), 25mM MgCl_2_, 75mM Na-glutamate, 5mM ATP, 0.4mM NADPH, and the appropriate crude protein extract. The reaction velocity was measured as the rate of consumption of NADPH, monitored as the decrease in absorption at 340nm as a function of time. Total protein content was determined according to Bradford method (Bradford, 1976). p5CS specific enzyme activity was expressed as units/mg protein.

#### Nitrate reductase

The assay was performed essentially as described in Liu *et al.* (2011), with some modifications. The buffer used for preparation of crude extracts contained 100mM potassium phosphate (pH 7.5), 5mM (CH_3_COO)_2_Mg, 10% (v/v) glycerol, 10% (w/v) polyvinylpyrollidone, 0,1% (v/v) Triton X-100, 1mM EDTA, 1mM DTT, 1mM PMSF, 1mM benzamidine (prepared fresh) and 1mM 6-aminocaproic acid. Leaf tissue was extracted in the appropriate buffer using a mortar and pestle and the mixture was thoroughly homogenized. Cell extract was centrifuged at 14 000 ×*g* for 15min and the clear supernatant was used immediately for measurement (Wray and Filner, 1970). Total protein content was determined according to the Bradford method (Bradford, 1976). NR activity was expressed as specific enzymatic activity (units/mg protein).

### qRT-PCR analysis

Total RNA was extracted from leaves using TRIzol (TRI reagent; Sigma-Aldrich, USA), followed by DNase digestion (RNase-free DNase Set; Qiagen). RNA integrity was analysed spectrophotometrically and by gel electrophoresis. One microgram of total RNA was converted into cDNA using Primescript 1st Strand Synthesis Kit (Takara, Japan) according to the manufacturer’s protocol. Subsequently, real-time RT-PCR was performed with Biorad IQ5 (Biorad, USA). Primer sequences of the products are listed in Supplementary Table S1 at *JXB* online. Relative quantification of gene expression and statistical analysis of all qRT-PCR data (pairwise fixed reallocation randomization test) were performed using the REST software according to Pfaffl *et al.* (2002). The actin 11 gene was used as a housekeeping reference gene (Filippou *et al.*, 2013).

### RNA labelling and Affymetrix expression array processing

RNA integrity screening, probe synthesis, hybridization and scanning were conducted by the BSRC Alexander Fleming’s Expression Profiling Unit. 300ng of total RNA was used to generate biotinylated complementary RNA (cRNA) for each treatment group using the GeneChip® 3’ IVT Express Protocol (Affymetrix, Santa Clara, CA) from the GeneChip® 3’ IVT Express Kit User Manual (Rev.8). In short, isolated total RNA was checked for integrity using the RNA 6000 Nano LabChip kit on the Agilent Bioanalyzer 2100 (Agilent Technologies, Inc., Palo Alto, CA) and concentration using the ND-1000Nanodrop (Thermo Fisher Scientific, Wilmington, DE). Poly-A RNA controls were added in each total RNA sample and were reverse transcribed using the included buffer and enzyme mixes. Double stranded cDNA was synthesized, labelled by *in vitro* transcription and purified with the appropriate protocol using beads (Affymetrix, Santa Clara, CA). Prior to hybridization, the cRNA was fragmented and 12.5 µg from each experimental sample were hybridized for 16h to Medicago Genome arrays in an Affymetrix GeneChip® Hybridization Oven 640. Affymetrix GeneChip® Fluidics Station 450 was used to wash and stain the arrays with streptavidin-phycoerythrin (Moleculer Probes, Eugene, OR), biotinylated anti-streptavidin (Vector Laboratories, Burlingame, CA) according to the standard antibody amplification protocol. Arrays were scanned with an Affymetrix GeneChip® Scanner 3000 at 570nm. All cRNA was synthesized at the same time. Images and data were acquired using the Affymetrix® GeneChip® Command Console® Software (AGCC) where initial quality check of the experiment was performed. The quality of the hybridizations was checked and one of the drought-treated samples was removed from subsequent analyses. The raw data was processed using the RMA algorithm (Irizarry *et al.*, 2003; affypackage of Bioconductor). A *t*-test statistic for comparison between drought samples and KM pre-treated drought samples was performed using the limma package of Bioconducter. The *P*-values of the *t*-test statistics were corrected for multiple testing to assess the false-discovery rate with the publicly available software QVALUE (http://genomine.org/qvalue; Storey and Tibshirani, 2003). Genes with *P*-values>0.001 and *Q*-value<0.05 were used for further analysis. The profiles of these genes were processed: expression values are inverse log-transformed RMA-processed values of the independent replicates. The absolute expression values are median-centred across each gene and again log2 transformed. The resulting data sets were subjected to linkage-means clustering (with a Euclidian distance metric; number of clusters is 6) with MultiExperiment Viewer of TM4 (Saeed *et al.*, 2003). GO enrichment analysis was performed for differentially expressed genes for both comparisons with cut-off values of logFC>1 and logFC<−1 using the GO enrichment tool of PLAZA 3.0 (dicots) (Proost *et al.*, 2014). GO terms were collected and summarized in lists of significantly enriched/depleted functional categories for each comparison.

### Metabolite profiling

GC-TOF-MS-based metabolite profiling was performed basically as described by Lisec *et al.* (2006). Polar metabolites were extracted from 50mg of frozen leaf material and 150 µl of each extract was used for the analysis. TagFinder (Luedemann *et al.*, 2012) was used for peak annotation and quantification with Golm Metabolome Database (http://gmd.mpimp-golm.mpg.de; Kopka *et al.*, 2005) as a reference library. The parameters used for the peak annotation are listed in Supplementary Table S2 according to Fernie *et al.* (2011). The intensity of each fragment was normalized by that of ribitol which was added into extraction solution as an internal standard. The intensity was further normalized by the mean of the values obtained from 0-day control samples and referred as metabolite levels. The changes in metabolite levels were evaluated by analysis of variance (ANOVA) followed by post-hoc testing using Tukey’s honest significance test conducted by aov, glht and cld functions in multcomp package in R.

### Statistical analysis

Statistical analysis of physiological and biochemical measurements was carried out using the software package SPSS version 21.0 (SPSS, Chicago, USA) and the comparison of averages of each treatment was based on the analysis of variance (one-way ANOVA) according to Duncan’s multiple range test at a significance level of 5% (*P*≤0.05).

## Results

### KM pre-treatment alleviates drought and salt stress-induced physiological damage

**Fig. 1.**
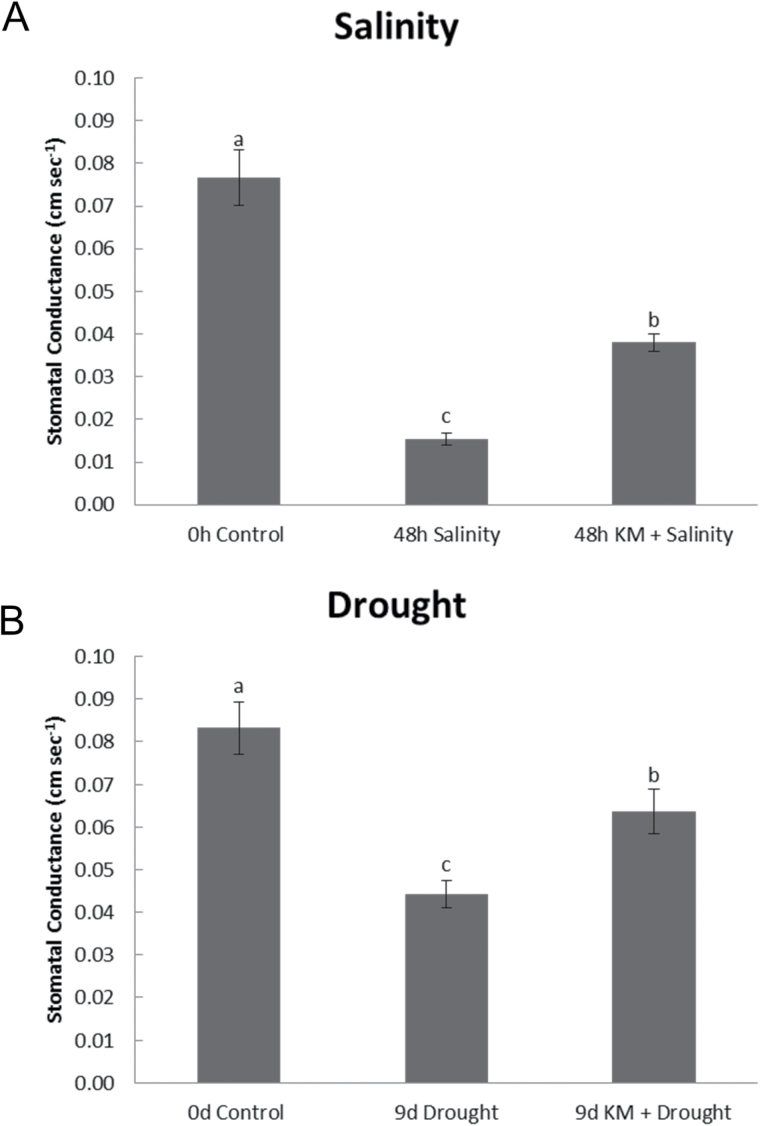
Leaf stomatal conductance in leaves of (A) salinity-stressed and (B) drought‐stressed *M. truncatula* plants in the absence or presence of KM pre‐treatment (KM 10^‐8^M).

Physiological processes were monitored by means of stomatal conductance measurements in leaves of *M. truncatula* plants. Imposition of drought and salinity stress to *M. truncatula* plants significantly lowered stomatal conductance (Fig. 1). However, pre-treatment with 10^–8^ M KM resulted in significantly higher readings in conductance compared with non-treated samples under both drought and salinity conditions (Fig. 1). Interestingly, the alleviation of the physiological parameter following KM pre-treatment was similar under both stresses.

### KM pre-treatment alleviates drought and salinity-induced oxidative stress

**Fig. 2.**
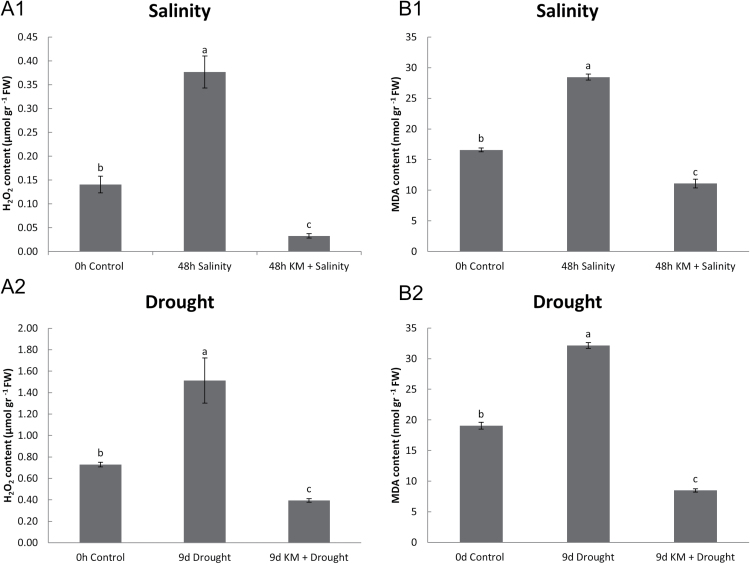
(A) Hydrogen peroxide content and (B) cellular damage (indicated by leaf MDA content) in salinity-stressed (A1, B1) and drought-stressed (A2, B2) *M. truncatula* plants in the absence or presence of KM pre-treatment (KM 10^–8^ M).

Hydrogen peroxide levels are massively induced in salt and drought-stressed plants, while pre-treatment with KM reverses this effect (Fig. 2A). Cellular damage due to increased ROS levels (Fig. 2A) was monitored by means of spectrophotometric determination of lipid peroxidation (Fotopoulos *et al.*, 2006) (Fig. 2B). Significant membrane damage was observed both under drought and salinity conditions; however, MDA content was significantly lowered following KM pre-treatment under both stress conditions, suggesting a protective role for KM (Fig. 2B). KM pre-treatment in control plants did not have any significant effect in MDA and H_2_O_2_ content in short- and long-term KM application (Supplementary Fig. S1A, B), revealing the compound was non-toxic at the concentration applied.

### KM pre-treated stressed plants demonstrate enhanced RNS content and NO biosynthetic enzyme activity

In addition to the enhanced accumulation of ROS following abiotic stress, recent reports indicated the participation of NO and other RNS in plant cell response. Most importantly, recent findings have suggested the existence of a cross-talk between ROS and RNS (Molassiotis and Fotopoulos, 2011).

**Fig. 3.**
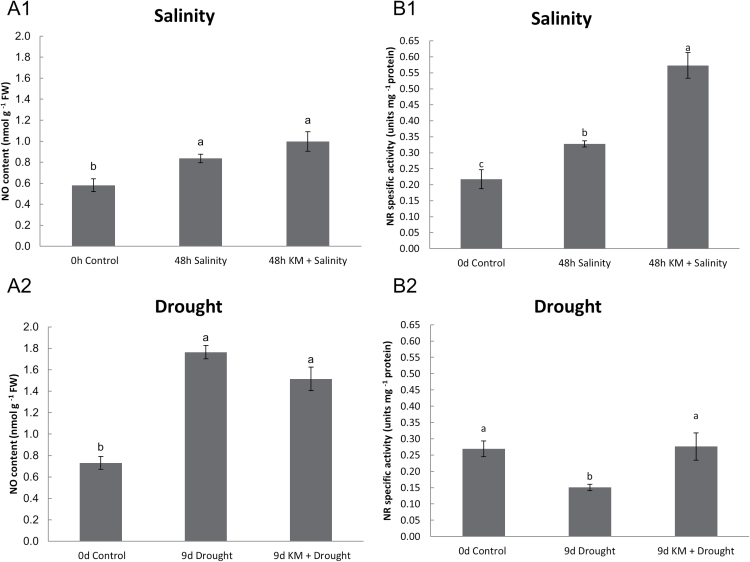
(A) Nitric oxide content and (B) nitrate reductase (NR) activity in salinity-stressed (A1, B1) and drought‐stressed (A2, B2) *M. truncatula* plants in the absence or presence of KM pre-treatment (KM 10^–8^ M).

To investigate the effect of KM on RNS content, NO was quantified in leaves of *M. truncatula* plants subjected to drought and salinity stress in the presence or absence of KM pre-treatment. Although both stress conditions resulted in increased NO content, maximum NO contents were recorded in drought-stressed plants (Fig. 3A). Interestingly, KM application had a different impact on NO content depending on the different stress applied. Pre-treatment of KM followed by salt stress induced a further increase of NO content, while the opposite effect was seen for plants with KM pre-treatment followed by drought-stress (Fig. 3A).

Subsequently, we measured NR enzyme activity in plants as this represents a major NO biosynthetic enzyme (Meyer *et al.*, 2005). In accordance with previous NO measurements, NR activity was differentially regulated depending on the different stresses applied (Fig. 3B). Salt stress application caused activation of NR activity (Fig. 3B1) in contrast to drought-stressed plants (Fig. 3B2). Upon KM pre-treatment, NR activation was observed in both salinity- and drought-stressed *M. truncatula* plants (Fig. 3B). NR activity was similar when KM pre-treated plants subjected to drought stress were compared with non-stressed, control plants, whereas drought stress significantly lowered NR activity (Fig. 3B). However, NR activity increased further in KM pre-treated salinity-stressed plants compared with non-stressed and salt-stressed plants in this order (Fig. 3B). Finally, KM pre-treatment in control plants (48h and 9 d) demonstrated no significant increase in NO content or NR activity (Supplementary Fig. S2).

### Effect of KM pre-treatment on proline content and p5CS enzymatic activity of stressed *M. truncatula* plants

**Fig. 4.**
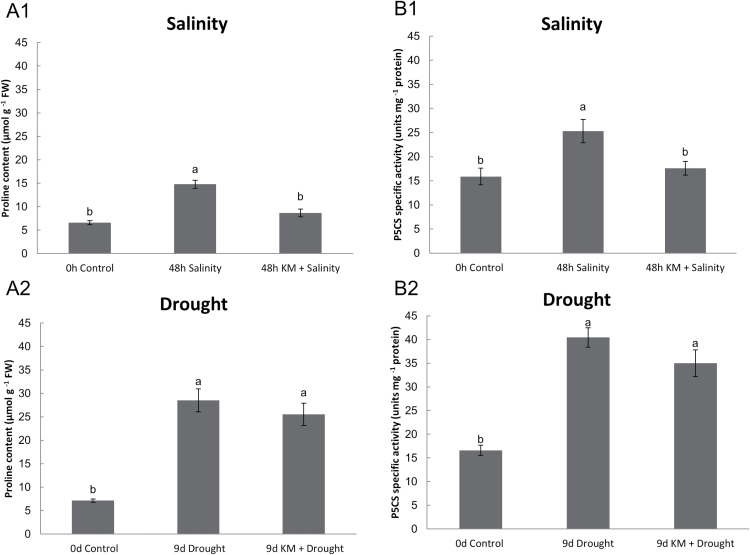
(A) Proline content and (B) P5CS (Δ1‐pyrroline‐5‐carboxylate synthetase) enzymatic activity measurements in salinity‐stressed (A1, B1) and drought-stressed (A2, B2) *M. truncatula* plants in the absence or presence of 10^‐8^ M KM pre‐treatment.

Free proline content and p5CS enzymatic activity, the key regulatory and rate-limiting enzyme in the proline biosynthetic pathway, were measured in drought- and salinity-stressed *M. truncatula* plants with or without KM pre-treatment (Fig. 4). Proline content increased under both stress factors, with the highest increase recorded under drought-stress conditions (~5-fold increase compared with controls) (Fig. 4A). Both stresses cause an increase in p5CS activity in parallel (Fig. 4B) with the increased proline levels (Fig. 4A).

KM pre-treatment resulted in a significant decrease in proline content in parallel with a decrease in activity of its biosynthetic enzyme in KM pre-treated and salt-stressed plants in comparison with salt-stressed plants (Fig. 4A1, B1). In contrast, proline content (Fig. 4A2) remained unchanged in drought-stressed plants following KM pre-treatment, in line with similar p5CS activity levels (Fig. 4B2). Interestingly, proline content increased in control KM pre-treated plants after long-term (9 d) KM application (Supplementary Fig. S1C).

### KM pre-treatment alters gene expression of important metabolic pathways in abiotic stressed plants

To assess the effect of KM pre-treatments on the transcriptome, we compared the transcriptome of pre-treated plants followed by salt or drought stress with that of stressed plants using Affymetrix GeneChip(r) Medicago Genome Arrays. Initially, to identify genes differentially expressed, a *t*-test for pairwise comparison (limma package from Bioconductor was used) was performed. A gene was considered to be significantly differentially expressed between two conditions if *P*<0.01 and *Q*<0.05 for all comparisons. KM pre-treatment differentially affected 646 and 57 transcripts (*P*<0.05; *Q*<0.01) in drought and salt-stressed plants, respectively (Supplementary Fig. S3). Surprisingly, only four transcripts were regulated in common for both stresses. Differential expression of these four transcripts was used to validate the microarray results by qRT-PCR on a biological repeat experiment (Table 1).

**Table 1.** Gene expression analysis of some key genes involved in protective defence mechanisms in leaves of drought- and salinity-stressed plants pre-treated with 10^–8^ M KM compared with respective stressed samples. The relative expression (fold change) of specific regulatory genes was determined by qRT-PCR in leaves of M. truncatula plants (values in bold letters indicate P<0.05, according to pairwise fixed reallocation randomization test). Microarray analysis expression values are also given for comparison purposes.

Genes	Salinity		Drought	
	qRT-PCR	Microarrays	qRT-PCR	Microarrays
**Proteolysis genes**				
Mt 7g 111 060	**−1.90**	**−1.57**	−1.33	0.32
Mt 7g 111 050	**−1.58**	**−1.61**	**−1.18**	0.03
Mt 4g 077470	−1.15	0.83	1.45	**−1.51**
Mt 5g 061690	1.16	0.34	−1.30	**−1.28**
**Common genes**				
Mt 1g 074950	1.51	**−1.70**	**−7.75**	**−4.90**
Mt 7g 093100	1.50	**1.76**	**−2.73**	**−3.19**
Mt 3g 070860	−2,09	**−1,53**	−1,62	**−1,79**
Mt 7g 024750	−1,69	**−1,74**	**5,22**	**2,01**

**Fig. 5.**
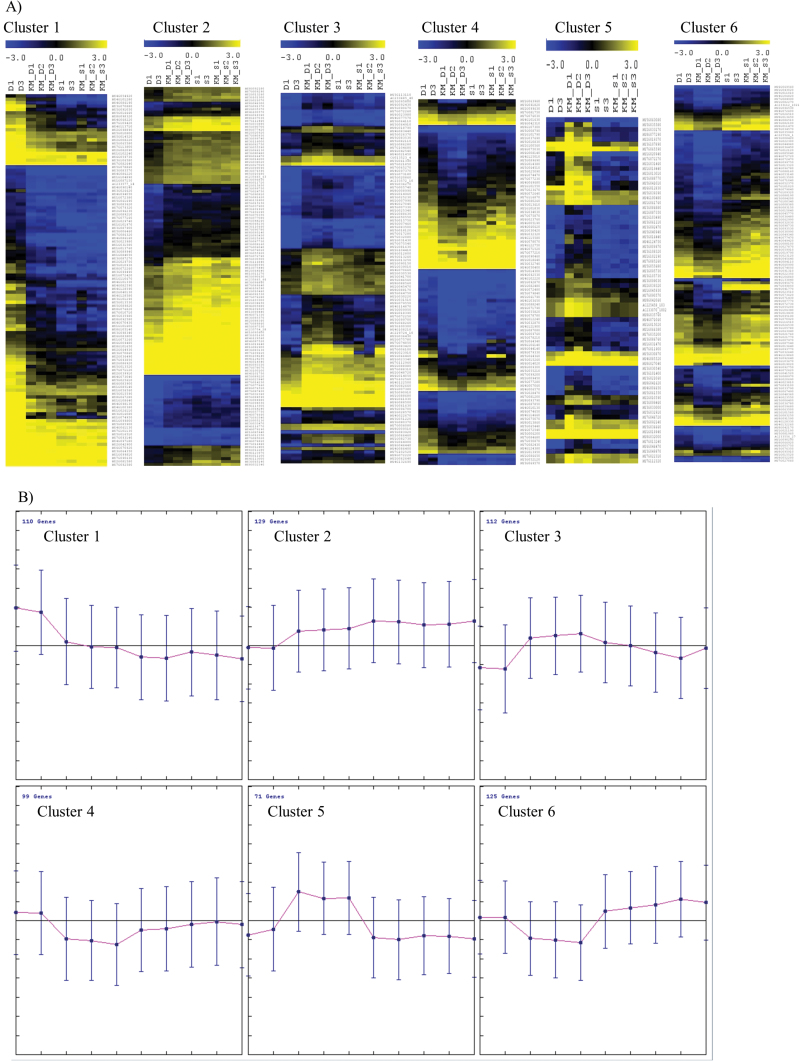
K‐means clustering (number of clusters: 6) of significantly expressed genes between drought‐stressed samples vs. drought‐stressed samples pre‐treated with 10^‐8^ M KM (FDR; *P*<0.05). (A) Heat map of median-centred values. (B) Average expression trends of the genes belonging to each cluster. The respective median-centred values of salt‐stressed samples with and without pre‐treatment with 10^‐8^ M KM are included in the clusters and depicted in the graphs. (This figure is available in colour at *JXB* online.)

K-means clustering analysis was performed between genes which consolidated the reproducibility of the different treatments (Fig. 5). Heat maps of six clusters are depicted in Fig. 5A; trend lines for each cluster are depicted in Fig. 5B. Six clusters were identified taking into account the gene regulation in both stresses (Fig. 5; Supplementary Table S3). Cluster 1 contains genes for which the change in expression levels is more pronounced under drought than after KM pre-treatment prior to drought stress (Fig. 5). Not surprisingly, genes that are mainly expressed under drought stress are genes that are responsive to abiotic stimuli (e.g. response to oxidative stress). Regulation of genes in cluster 1 suggested an enhanced response activity to different kinds of stimuli (response to hormone, carbohydrate, abscisic acid and jasmonic acid stimulus), as well as MAPK signalling pathways under stress conditions. Remarkably, among the genes for which the change in expression levels is less pronounced following KM pre-treatment compared with drought-stressed plants are genes related to several metabolic processes, i.e. ethylene responsive factors, carbohydrate, and cellular nucleotide-sugar metabolic processes (clusters 4 and 6, Fig. 5).

In contrast, clusters 2, 3 and 5 contain genes for which the change in expression levels is less pronounced under drought conditions than in KM pre-treated plants prior to drought (Fig. 5). These clusters are enriched with genes implicated in primary and secondary metabolic processes (clusters 3 and 5, Fig. 5; Supplementary Table S3) and also included proteins with specific molecular functions like oxidoreductase activity (cluster 5, Fig. 5) and transporter activity (i.e. amino acid transmembrane transporter activity) (cluster 3, Fig. 5; Supplementary Table S3). More specifically, cluster 3 contains genes which are implicated in cellular amino acid metabolic processes as well as amino acid transport (Fig. 5). Notably, there are also genes implicated in flavonoid metabolic processes (Supplementary Table S3).

### KM affects the proteolysis pathway at the transcriptional level

Next, we examined the effect of KM pre-treatment on the cellular processes related to protein hydrolysis in drought-stressed plants. Out of 646 significantly regulated genes, 38 are involved in proteolysis or amino acid metabolism (~6%) in drought with and without KM pre-treatment. We composed a customized ‘proteolysis’ list containing 1155 genes. Out of 38, 21 are up-regulated in the pre-treated samples, whereas 17 out of 38 are down-regulated in the pre-treated samples. Interestingly, 2 out of 57 significantly regulated genes in KM pre-treated salt-stressed samples are involved in proteolysis or amino acid metabolism (~4%) and are both down-regulated in the pre-treated samples (Table 1). The expression of these two hydrolysis genes and two additional genes selected out of the 38 genes was verified by qRT-PCR analysis (Table 1).

**Fig. 6.**
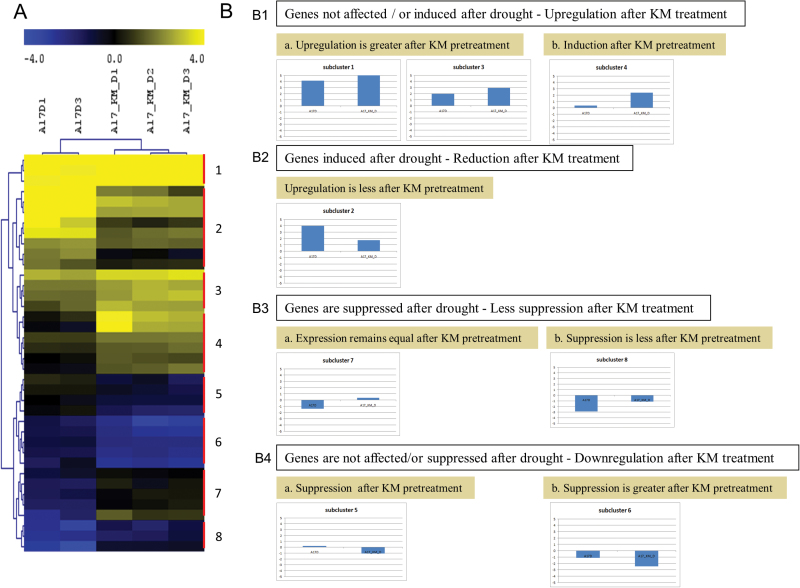
Effect of KM pre-treatment in drought-stressed regulated proteolytic genes. (A) Hierarchical clustering of significantly expressed genes D_Km vs. D (FDR; *P*<0.05) involved in proteolysis. (B) Summary of the expression profiles per subcluster based on the average of the median-centred values of genes belonging to the subcluster. (This figure is available in colour at *JXB* online.)

Hierarchical cluster analysis of D-KM vs. D (Supplementary Table S4) stressed samples and the subsequent grouping of similar response clusters (Fig. 6B; Supplementary Table S4) suggested the differential regulation of a number of proteins responsible for protein degradation as well as proteins regulating peptidases and their inhibitors (Fig. 6).

Genes from subclusters 1, 3 and 4 were up-regulated following KM pre-treatment (Fig. 6B1). These subclusters consisted either of genes in which the up-regulation was increased following KM pre-treatment (subclusters 1, 3; Fig. 6B1) or of genes that were induced following KM pre-treatment, although drought stress did not affect their expression (Subcluster 4). In contrast, subcluster 2 consisted of genes (most of them protease inhibitors) that were induced by drought but their up-regulation was less pronounced following KM treatment (Fig. 6B2). Moreover, further examination of the results revealed the regulation of genes involved in proteolysis that are suppressed under drought stress and which are further suppressed following KM pre-treatment (subcluster 6), or genes that are not affected by drought but are suppressed following KM treatment (subcluster 5) (Fig. 6B4).

In subclusters 7 and 8, the expression of genes involved in protein hydrolysis and peptidase activity remains equal (subcluster 7) or is down-regulated to a lower extent (subcluster 8) after KM pre-treatment compared with drought-stressed plants (Fig. 6B3).

### KM pre-treatment affects the metabolite profile of drought and salinity-stressed plants

**Fig. 7.**
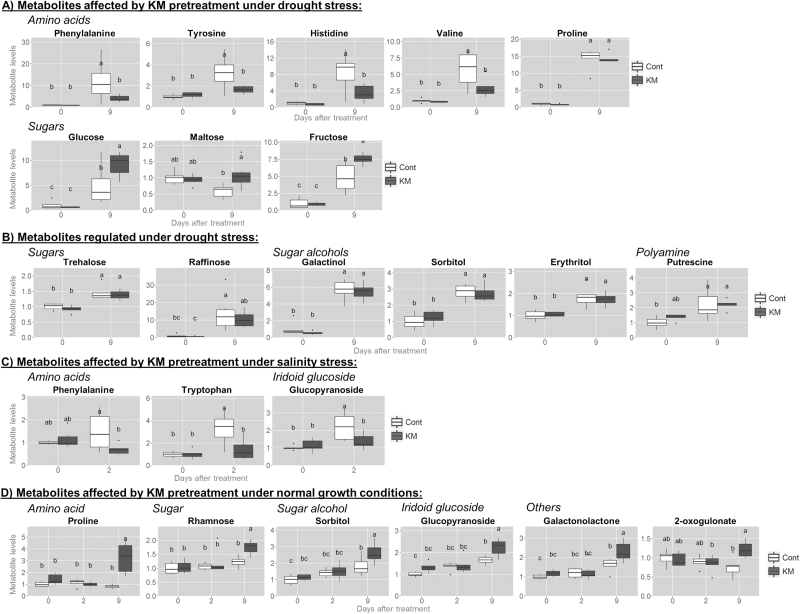
Exemplary profiling of (A) metabolites significantly regulated by 10^–8^ M KM pre-treatment under drought-stress conditions, (B) metabolites significantly regulated under drought-stress conditions, (C) metabolites significantly regulated by 10^–8^ M KM pre-treatment under salt-stress conditions, and (D) metabolites significantly regulated by 10^–8^ M KM pre-treatment under normal growth conditions in leaves of *M. truncatula* plants.

To further clarify the effect of KM, GC-MS analysis was performed in salinity and drought-stressed samples with and without KM pre-treatment (Fig. 7). GC-MS analysis identified metabolites belonging to different classes, including two major groups of sugars and amino acids, as well as organic acids and various other compounds (nitrogenous compounds and polyols).Although the levels of many metabolites were not significantly different following KM treatment, ANOVA detected some metabolites that changed significantly (*P*<0.05, Fig. 7).

Notably, glucose, fructose and maltose levels increased significantly in KM pre-treated and drought-stressed plants compared with drought-stressed samples (Fig. 7A). Some sugar alcohols (i.e. galactinol, sorbitol and erythritol), sugars (trehalose and raffinose) and an amine (putrescine) increased in drought-stressed plants (Fig. 7B). These compounds were not significantly affected by KM pre-treatment (Supplementary Fig. S4C). Interestingly, KM regulation of sugar metabolism is different in salinity-stressed plants. No sugar is significantly increased and/or decreased in KM pre-treated salt-stressed plants (Supplementary Fig. S4B). Furthermore, the effect of KM alone in plants grown under normal conditions (pre-treated KM control plants after 48h and 9 d) indicated a significant increase in one sugar (rhamnose) and one sugar alcohol (sorbitol) after prolonged exposure to KM (9 d treatment) (Fig. 7D).

Moreover, no organic acids implicated in the TCA cycle do not significantly change in KM pre-treated and subsequently stressed plants (Supplementary Fig. S4A, B). Glucopyranoside is the only (non-amino acid) metabolite that is significantly affected following KM pre-treatment in salinity-stressed plants (Fig. 7C). A different regulatory mechanism of these metabolites was observed in KM pre-treated plants under control growth conditions. Glucopyranoside, galactonolactone and 2-oxogulonate were significantly increased in 9 d KM-treated samples (Fig. 7D).

### KM pre-treatment causes a decrease in specific amino acid levels in drought-stressed plants

KM pre-treatment results in a significant reduction of amino acids, irrespective of the stress treatment. Focusing on drought stress, the most dramatic differences were observed in aromatic amino acids (phenylalanine, tyrosine) (Fig. 7A). Significantly decreased levels in aromatic amino acids were also observed in histidine and valine levels. A remarkable exception to the trend of reduced amino acid levels following KM pre-treatment was proline content, indicative of its protective effect (Fig. 4A2).

Reduction in amino acid levels was less pronounced in salt-stressed plants. Only two of the amino acids (tryptophan and phenylalanine) significantly decreased following KM pre-treatment (Fig. 7C), while most of the amino acids remained at constant levels with and without KM pre-treatment (Supplementary Fig. S4B). Similar to drought stress conditions, KM pre-treatment and subsequent salt stress did not affect putrescine levels (Supplementary Fig. S4B).

The effect of KM after 48h and 9 d spraying without subsequent stress treatment demonstrated that amino acids are not affected in a similar manner compared with KM pre-treated stressed plants. Interestingly, amino acid levels differ according to the duration of application, although no significant change in amino acids levels was observed (Supplementary Fig. S4C). Moreover, proline levels increased dramatically upon long-term (9 d) KM pre-treatment (Fig. 7D). Proline significantly increased 9 d after KM treatment indicating the protective role of KM applied to the plant. Putrescine levels were also not affected by KM application.

## Discussion

Improving plant productivity under saline and drought conditions via the exogenous supply of chemical compounds constitutes a highly important agronomic challenge (Bartels and Sunkar, 2005). However, chemical approaches to increase growth and tolerance of plants to abiotic stresses by targeting specific enzymes or acting via established inhibitory modes of action have received considerably less attention (Schulz *et al.*, 2012). KM is an organic chemical compound synthesized from the secondary metabolite strobilurin A that was found to induce non-fungicidal physiological changes (Grossmann and Retzlaff, 1997; Grossmann *et al.*, 1999). In the present study, we tried to unravel the molecular and biochemical mechanisms implicated in the priming effect of KM in drought and salinity-stressed mature *M. truncatula* plants.

Stress conditions lead to limitations of photosynthetic capacity and stomatal closure (Gill and Tuteja, 2010). Indeed, stomatal conductance was ameliorated significantly under both salinity and drought stress conditions (Fig. 1) following KM pre-treatment, in accordance with previous reports showing KM-induced increase in stomatal conductance (Grossmann *et al.*, 1999), which could potentially lead to increased CO_2_ uptake and improved overall photosynthetic performance.

ROS production (H_2_O_2_ content) is one of the major primary stress responses, causing either cellular damage at higher concentrations or acting as a signal molecule to be transmitted at lower concentrations (Gechev and Hille, 2005). If this massive ROS production is not controlled by antioxidant mechanisms, lipid membrane peroxidation can occur, resulting in oxidative damage at the cellular level (Gill and Tuteja, 2010). The higher level of protection in stressed *M. truncatula* plants pre-treated with KM can be explained by the reduction of H_2_O_2_ levels (Fig. 2A) and the subsequent decrease of the cellular damage levels induced by both stresses (Fig. 2B).

In addition to ROS, RNS content (NO) was also measured. NO can act as a biomarker of nitrosative stress or as a protective signalling molecule (Zhao *et al.*, 2007). A further increase of NO content was observed following KM pre-treatment in salt-stressed plants, indicating the self-amplifying process of NO production induced by NO acting as a signalling molecule (Dwivedi and Choudhury, 2012) closely linked to KM. A different regulation pattern was observed in KM pre-treated, drought-stressed plants (Fig. 3A2). Alleviation of the accumulation of NO in drought-stressed plants following KM pre-treatment could be due to the inhibition of NO-producing enzymes including NR, in order to inhibit nitrosative stress (Fig. 3A2). A differential regulation of NR was observed between the two abiotic stresses (Fig. 3B), in accordance with NO production (Fig. 3A). KM pre-treatment increased the salt-induced NR activation (Filippou *et al.*, 2014), thus leading to the subsequent increase of NO content (Fig. 3A1). In contrast, drought-stressed *M. truncatula* plants resulted in a reduction (Rosales *et al.*, 2011) in NR activity (Fig. 3B2), possibly due to NO accumulation (Fig. 3A2), resulting in a negative feedback regulatory mechanism and/or cell nitrate depletion (Antoniou *et al.*, 2013). Regulation of NR activity might potentially occur via post-translational modification (Kaiser *et al.*, 2002), considering that *NR* gene expression did not show any significant changes in both KM pre-treated and subsequently drought- or salt-stressed plants compared with stressed-only plants (data not shown). The enhanced NR activity in drought- and salt-stressed plants (Fig. 3B1, B2) following KM pre-treatment compared with the respective stressed plants constitutes KM as a protective molecule that might act by recovering the stress-induced damage of the plasma membrane and/or enhancing nitrate uptake in stressed plants (Forde, 2002).

Surprisingly, the effect of KM on plants grown in the absence of stress (48h and 9 d treatment) did not affect the *in vitro* NR activity (Supplementary Fig. 2B), contrary to results by Glaab and Kaiser (1999), showing the *in vivo* KM-induced activation effect of NADH-NR. The fact that activation of NR was observed only in salt- and drought-stressed plants following KM pre-treatment (Fig. 3B) and not in pre-treated KM control plants (Supplementary Fig. 2B), with no difference in *NR* gene regulation (data not shown), indicates that NR regulation probably occurs via KM interference in the abiotic defence mechanism rather than via transcriptional/post-translational activation.

An important aspect among the wide variety of physiological and biochemical changes induced in plants for protection against stresses is the accumulation of osmolytes (Ashraf and Foolad, 2007). Accumulation of the osmolyte proline during drought and salinity stresses improves adaptation by acting as a protein-compatible hydrotrop and radical scavenger (Matysik *et al.*, 2002). Interestingly, no significant changes in proline accumulation were observed in drought-stressed plants following KM pre-treatment (Fig. 4A2), probably due to the significant proline induction after KM application on non-stressed plants (9 d after treatment) (Supplementary Fig. S1C). These results indicate the function of KM as a protective priming molecule, sustaining proline levels in abiotic stressed plants for maintaining the plant stress tolerance response (Fig. 4A). Additionally, proline accumulation in 9 d KM-treated control plants renders KM as an important factor to maintain high photosynthesis activity (Akbari *et al.*, 2011; Supplementary Fig. S1C). p5CS activity, which is the key regulatory and rate limiting stress-inducible enzyme in the proline biosynthetic pathway (Szabados and Savouré, 2009), is regulated in abiotic-stressed plants (Silva-Ortega *et al.*, 2008) (Fig. 4B) and KM pre-treated plants in line with proline levels (Fig. 4A).

Regarding the previous biochemical data, it is obvious that plant metabolism dramatically changes under different stress conditions (Obata and Fernie, 2012) and after KM foliar application. Therefore, the metabolomic approach was used as a powerful tool to gain an overview of the metabolite changes in KM pre-treated stressed plants. To that point, discussion will be focused mainly in drought-stressed plants since the transcriptomic (Fig. 6) and metabolomic regulation (Fig. 7) was more pronounced in KM pre-treated and subsequently drought-stressed plants.

Similar to previous studies (Seki *et al.*, 2007), drought stress induces the accumulation of several metabolites that act as antioxidants or scavengers, helping the plants to tolerate and/or avoid stresses. As shown in Fig. 7, levels of several amino acids, including the compatible osmolyte proline, some sugar alcohols (galactinol, sorbitol and erythritol) along with sugars (glucose, fructose, trehalose and raffinose) and a polyamine (putrescine) increase in drought-stressed plants (Fig. 7A, B).

KM pre-treatment in drought-stressed plants sustained the drought-induced levels of some key osmoprotective metabolites (for a review see Vinocur and Altman, 2005) (i.e. proline, trehalose, sucrose and *myo*-inositol, its direct and more downstream derivatives galactinol and associated raffinose-family oligosaccharides). Interestingly, KM pre-treatment in drought-stressed plants further enhanced the increase in soluble sugars (namely glucose and fructose) (Fig. 7A) that act as signalling molecules of water deficits (Chaves and Oliveira, 2004), possibly due to the change of the stress-induced interaction with hormones as part of the sugar sensing /signalling network in plants (Rolland *et al.*, 2006).

The activation of glycolysis and/or other pathways might contribute to plant survival by ensuring production of energy and metabolites (Baxter *et al.*, 2007). It seems that KM protects the plant with no need for extra energy, since there is no necessity of glucose catabolism (increase of glucose) following KM pre-treatment [the other metabolites (TCA cycle intermediates and organic acid levels) remain unchanged; no necessity for ATP synthesis] (Supplementary Fig. S4A). Surprisingly, no significant effect in almost any the metabolites was observed in KM pre-treated and subsequently salinity-stressed plants (Supplementary Fig. S4B), suggesting that KM exhibits regulation between the differential two stresses.

From the metabolite analysis, it is more than obvious that contents of several amino acids increased in drought-stressed plants (Fig. 7A), whereas a less severe effect was observed in salinity-stressed plants (Fig. 7C). Specific amino acids (aromatic amino acids and histidine) have been correlated with stress tolerance (Sharma and Dietz, 2006) and among the non-proteogenic amino acids (GABA and β-alanine), only β-alanine increased specifically under oxidative stress conditions (Lehmann *et al.*, 2012). The increase of amino acid levels in drought stress (Fig. 7A) might be related to an increased tissue damage and senescence (Sanchez *et al.*, 2008) due to H_2_O_2_ production (Sharma and Dietz, 2006) (Fig. 2A2), an increase in protein degradation and an inhibition of protein synthesis (Akbari *et al.*, 2011). Most of the amino acid accumulation was alleviated in KM pre-treated plants (Fig. 7A, C) possibly due to inhibition of protein hydrolysis (Fig. 6). Indeed, KM seems to increase the level of total protein, probably by its involvement in regulating protein hydrolysis (Fig. 6) or transcription and/or translation/post-translational regulation likewise to other molecules such as brassinosteroids (Bajguz and Hayat, 2009).

Investigating other amine compounds, accumulation of putrescine was observed in drought-stressed plants associated with stress tolerance (Alcázar *et al.*, 2010). Contrary to the accumulation of polyamines during pre-treatment with other molecules such as salicylic acid (Palma, 2013), a further induction of putrescine content was not observed in KM pre-treated, drought-stressed plants (Supplementary Fig. S4A). It is possible that the two other important polyamines (spermidine and spermine) are affected by KM application but this hypothesis needs to be tested.

A global *M. truncatula* transcriptome analysis for studying the effect of KM treatment on plants subjected to different abiotic stress conditions (salinity and drought) was performed. Because of the high number of regulated transcripts implicated in drought stress response following KM pre-treatment and bigger effect in metabolite analysis compared with the salt stress response (Fig. 7), we further focused in the differentially expressed genes between KM pre-treated plants subjected to drought. The difference in response on transcript and metabolite levels between pre-treated salt and drought-stressed plants (Figs 5, 7), suggests that each different stress condition generates a somewhat unique response and the existence of distinct mechanisms involved in the regulation of stress-responsive genes (Fowler and Thomashow, 2002; Seki *et al.*, 2002) following KM treatment.

Little overlap in transcript expression was found (only four genes) between the responses of plants to both KM-primed conditions (Table 1; Supplementary Fig. S3). Among these genes, two commonly regulated genes were down-regulated following KM pre-treatment in drought-stressed plants. These genes encode cell signalling proteins implicated in defence mechanisms (i.e. protein kinases; HAT family dimerisation domain) (Jain *et al.*, 2007) and antioxidant biosynthetic enzymes participating in flavonoid biosynthesis (Hernandez *et al.*, 2009).

K-means clustering analysis was performed and the differentially expressed genes were grouped in a total of six clusters with distinct expression trends. Cluster 1 (Fig. 5) contains genes for which the expression is less pronounced in KM pre-treated and subsequently drought-stressed plants compared with drought-stressed plants, Interestingly, this cluster includes several drought-inducible genes that have been recently identified (Matsui *et al.*, 2008). For instance, the activation and positive regulation of MAPK components involved in osmosensory signalling pathways (Boudsocq and Laurière, 2005) showed decreased expression following KM pre-treatment (cluster 1, Fig. 5). Moreover, the expression profile of genes responsive to osmotic stress and abiotic stimulus such as peroxidases (Kant *et al.*, 2008) is less pronounced following KM pre-treatment (cluster 1, Fig. 5). These results suggest that plant metabolism quickly adapts to drought stress conditions following KM pre-treatment for plant survival. KM-treated plants are constitutively displaying a ‘recovery response’ (Araujo *et al.*, 2012) as a result of the pre-adaptation of plants to subsequent stress factors (Sanchez *et al.*, 2011; Benina *et al.*, 2013), so there is no need for any further energy consumption for the activation of these crucial metabolic processes.

Moreover, since KM has been suggested as a hormone-like compound and/or hormonal regulator (Grossmann and Retzlaff, 1997; Grossmann *et al.*, 1999), the effect of KM in the expression of genes that are implicated in phytohormone metabolism was also studied in drought-stressed plants. The cellular response to drought stress triggers the production of various phytohormones (Golldack *et al.*, 2011), thereby leading to hormone stimulus such as abscisic acid (ABA), jasmonic acid (JA) and brassinosteroid stimuli (cluster 1, Fig. 5) for the induction of stress-inducible genes (Yamaguchi-Shinozaki and Shinozaki, 2005). KM might therefore alleviate the induction of phytohormone (ABA and JA) stimuli that act as key regulators in drought-induced signalling cascades (Golldack *et al.*, 2011).

It is well known that photosynthesis, energy homeostasis, redox balance and metabolism are closely related (Foyer, 2005). The effect of KM on the redox balance and photosynthesis might lead to growth enhancement associated with a reduced induction of protective pathways (Nunes-Nesi *et al.*, 2005). Clusters 2, 3 and 5 (Fig. 5) contain genes of which the expression is less pronounced under drought, compared with samples that were pre-treated with KM prior to stress imposition. These clusters are enriched with genes implicated in cell homeostasis, such as cytochrome P450 monoxygenases, GSTs and alcohol dehydrogenases. The increased expression levels of genes involved in redox metabolism following KM pre-treatment, suggests that KM protects the plant against drought stress by modulating cell redox metabolism. Similarly, KM had the most dramatic effect in genes belonging to cluster 5 (Fig. 5) which are encoding enzymes involved in metabolic processes i.e. enzymes with oxidoreductase activity, therefore suggesting a beneficial role for KM pre-treatment in redox metabolism.

Importantly, the expression of genes that belong to families of primary metabolic processes and protein binding, transcriptional factors and regulators (Supplementary Table S4) like MYC, bHLH and MYB proteins (Abe *et al.*, 2003), as well as WRKY proteins (Marè *et al.*, 2004) are less pronounced in KM pre-treated and subsequently drought-stressed plants than in drought-stressed plants (Supplementary Table S4). Moreover, the reduction in transcript levels of ethylene-responsive transcription factors (i.e. ERF5, ERF6, ERF019, ERF026 and TINY) in KM pre-treated and subsequently drought-stressed plants (Supplementary Table S4) comes in agreement with previous reports, suggesting that KM affects ACC synthase and inhibits ethylene biosynthesis. This is further accompanied by delayed senescence and reduced chlorophyll loss (Grossmann and Retzlaff, 1997). In contrast, biosynthesis of other hormones like cytokinins and auxins is further induced following KM treatment, therefore suggesting a broad cross-talk between KM and phytohormones (Grossmann and Retzlaff, 1997).

An interesting remaining question is the reason for the decrease in amino acid accumulation following KM pre-treatment in drought-stressed plants (Fig. 7). The lower amino acid accumulation in drought-stressed plants following KM pre-treatment could be due to an increase in export from the cell (amino acids transport activation) and/or reduced amino acid synthetic rate or catabolism in the TCA cycle, ultimately leading to the decrease in amino acid content (Fig. 7). Another viewpoint on the regulation of amino acid levels is enzyme hydrolysis (Sharma and Dietz, 2006). In an effort to unravel these questions, we decided to focus our transcriptomic analysis on genes involved in protein and amino acid metabolism. The transcriptome changes under drought alone and KM pre-treated and drought-stressed conditions revealed a change in transcripts involved in protein degradation (Fig. 6). Figure 6A shows a heat map of genes responsible for protein degradation as well as genes that regulate peptidases and their inhibitors (Supplementary Table S4). The overall decrease in amino acid levels (Fig. 7) is probably due to a decrease in proteolysis levels following KM pre-treatment (Fig. 6B4).

Moreover, endopeptidase/peptidase inhibitors are also down-regulated following KM pre-treatment prior to stress imposition compared with drought-stressed plants (subcluster 2 from heat map; Fig. 6B2), as a consequence of the lack of necessity for the cell to consume more energy. Notably, two important inhibitors (Bowman-Birk type proteinase inhibitor and Kunitz-type trypsin inhibitor-like 2 protein) were suppressed following KM pre-treatment compared with drought-stressed plants (Supplementary Table S4). The decrease in amino acids inside the cell following KM pre-treatment (Fig. 7) could also be due to the increase in cellular amino acid metabolic process and/or transport (Fig. 6, subclusters 1, 3, 4, 7 and 8). This can be explained by the induction of genes involved in cellular amino acid and derivative metabolic processes in KM pre-treated and subsequently drought-stressed plants (Supplementary Table S4).

In summary, a number of proteases were suppressed in drought-stressed plants following KM pre-treatment, (subclusters 5 and 6, Fig. 6) with a subsequent suppression of their protease inhibitors (subcluster 2, Fig. 6), resulting in a decrease in amino acid content. In contrast, the alleviation of the suppression following KM pre-treatment of some proteases (subclusters 7 and 8, Fig. 6) suggests that another mechanism can be responsible for the amino acid content decrease following treatment with this chemical compound.

Overall, the priming effect of KM against key abiotic stress factors was explored under controlled growth conditions, following a comprehensive, fundamental approach. However, it should be noted that downstream field verification is important in order to validate the commercially applied potential of this promising priming agent, as several studies have indeed reported contradictory findings between laboratory and field-grown plants. Examples include the work of Wituszynska *et al*. (2013), who demonstrated that runaway cell death in lsd1 mutant observed in laboratory (non-permissive) conditions was not visible in non-permissive field conditions while, in contrast, Kulheim *et al*. (2002) demonstrated that no visible phenotype was observed for npq1 mutants under laboratory conditions although a very clear phenotype was observed in the field.

## Conclusion

In conclusion, KM is an established chemical agent widely used as a strobilurin fungicide, which is now emerging as a novel priming inducer, associated with differential protection against two abiotic stress factors – drought and salinity. Foliar application of KM in *M. truncatula* plants significantly ameliorated the deleterious effects of salinity and drought on plant physiology, confirming the modulation of stress management via regulation of a multitude of cellular, biochemical and molecular processes. Acting as a protective molecule, KM sustains the abiotic stressed response mechanism by regulating plant metabolism. *M. truncatula* metabolism was demonstrated to be able to overcome stress-induced oxidative consequences following KM pre-treatment by regulating independent pathway-specific processes. Such a response after application of a priming agent is very beneficial for plant metabolism since it ensures both energy production and respiration, thus demonstrating the importance of the metabolic maintenance in KM-primed plants for plant survival. The fact that KM is a synthetically derived strobilurin fungicide, which is applied at extremely low doses and is capable of improving crop performance, makes strobilurins, and KM in particular, a suitable candidate for their future application in agriculture, especially under adverse climatic conditions. Future experiments should focus on the evaluation of this compound and the acquisition of agronomic data under field conditions, where plants are subjected to constantly fluctuating environmental conditions.

## Supplementary data

Supplementary data are available at *JXB* online.

Table S1. Sequences of gene-specific primers.

Table S2. Parameters used for metabolite peak annotation.

Table S3. Gene ID of different clusters from Fig. 5.

Table S4. (A) List of differentially expressed transcripts in stressed samples vs. primed and stressed with a log2 FC>1. (B) respective GO enrichment of drought vs. KM+drought samples. (C) List of differentially expressed transcripts in stressed samples vs. primed and stressed with a log2 FC<−1. (D) respective GO enrichment of drought vs. KM+drought samples.

Fig. S1. Effect of 10^−8^M KM pre-treatment in (A) hydrogen peroxide content, (B) cellular damage indicated by leaf MDA content and (C) proline content.

Fig. S2. Effect of 10^−8^M KM pre-treatment in (A) NO content and (B) nitrate reductase (NR) activity.

Fig. S3. Venn diagram showing number of significantly regulated transcripts in primed and stressed vs. stressed plants.

Fig. S4. Metabolite profiling of (A) drought- and (B) salinity-stressed samples compared with primed and stressed samples, and (C) KM pre-treated plants under normal conditions.

Supplementary Data
