# Supplementary material for: Kresoxim-methyl primes Medicago truncatula plants against abiotic stress factors via altered reactive oxygen and nitrogen species signalling leading to downstream transcriptional and metabolic readjustment
Source: J Exp Bot. 2015 Dec 27;67(5):1259–74. doi: 10.1093/jxb/erv516 (PMC4762377; doi:10.1093/jxb/erv516)
Supplement: Supplementary Data [file supp_67_5_1259__index.html]

Kresoxim-methyl primes Medicago truncatula plants against abiotic stress factors via altered reactive oxygen and nitrogen species signalling leading to downstream transcriptional and metabolic readjustment — Kresoxim-methyl primes Medicago truncatula plants against abiotic stress factors via altered reactive oxygen and nitrogen species signalling leading to downstream transcriptional and metabolic readjustment — Supplementary Data 

# Kresoxim-methyl primes *Medicago truncatula* plants against abiotic stress factors via altered reactive oxygen and nitrogen species signalling leading to downstream transcriptional and metabolic readjustment

## Supplementary Data

Data files

- Supplementary\_figures\_S1\_S4\_\_\_Table\_S1.pdf - Supplementary Data
- Supplementary\_Table\_S2.xlsx - Supplementary Data
- Supplementary\_Table\_S3.xlsx - Supplementary Data
- Supplementary\_Table\_S4.xlsx - Supplementary Data
