# Supplementary material for: Kresoxim-methyl primes Medicago truncatula plants against abiotic stress factors via altered reactive oxygen and nitrogen species signalling leading to downstream transcriptional and metabolic readjustment
Source: J Exp Bot. 2015 Dec 27;67(5):1259–74. doi: 10.1093/jxb/erv516 (PMC4762377; doi:10.1093/jxb/erv516)
Supplement: Supplementary Data [file supp_erv516_Supplementary_figures_S1_S4___Table_S1.pdf]

Kresoxim-methyl primes against abiotic stress factors via altered reactive oxygen and nitrogen species signalling leading to downstream transcriptional and metabolic readjustment in *Medicago truncatula*

Panagiota Filippou, Chrystalla Antoniou, Toshihiro Obata, Katrien Van Der Kelen, Vaggelis Harokopos, Loukas Kanetis, Vassilis Aidinis, Frank Van Breusegem, Alisdair R Fernie and Vasileios Fotopoulos

Supplementary Material

The following supplementary materials are available.

Supplementary Table S1. Sequences for gene-specific primers.

Mt7g111060-F	5'-TGGCTTCTTGCAAATCAGTG-3'
Mt7g111060-R	5'-AACACCAGCATCAACATCCA-3'
Mt7g111050-F	5'-AAACACCCCGAAGTGAACTG-3'
Mt7g111050-R	5'-ACCAAATGCCCAACAAGAAC-3'
Mt7g093100-F	5'-TGTGCAGCTCGCAATAAAAG-3'
Mt7g093100-R	5'-GGAGTTACATCACGGCCACT-3'
Mt3g070860-F	5'-CCACAATCTTGCAGGGAAGT-3'
Mt3g070860-R	5'-AATGTCAATTCTGGCCTTGG-3'
Mt1g074950-F	5'-GGCTTTGCTGAAACGAGTTC-3'
Mt1g074950-R	5'-AAGTTCCAAAGCAGGAACGA-3'
Mt7g024750-F	5'-GGCGCAGGCTATAAGTTTGA-3'
Mt7g024750-R	5'-ATGGCTGTGGTGAGAAATCC-3'
Mt4g077470-F	5'-CTTTTGCCCGAGAGGGTATT-3'
Mt4g077470-R	5'-CAGGGGTGGTGACAGAGTTT-3'
Mt5g061690-F	5'-TGGCTGTTGGTGTTCTTCTG-3'
Mt5g061690-R	5'-CCCTCTTAGGCCATGATTGA-3'

Supplementary Table S2. Parameters used for metabolite peak annotation.

Supplementary Table S3. Gene ID of different clusters from Fig 5: Gene ID (column B), gene description (column C) median centered expression values for each sample (column D-M). (B) Overview of GO enrichment for each cluster from Fig. 5.

Supplementary Table S4. (A) List of differentially expressed transcripts (FDR; $p<0.05$) in drought-stressed samples vs. samples pretreated with KM followed by drought (D_KM vs D) and salt-stressed samples vs. samples pretreated with KM followed by salt (S_KM vs S) with a log2 FC >1. (B) GO enrichment of differentially expressed transcripts (FDR; $p<0.05$) in drought-stressed samples vs. samples pretreated with KM followed by drought (D_KM vs D) (C) List of differentially expressed transcripts (FDR; $p<0.05$) in drought-stressed samples vs. samples pretreated with KM followed by drought (D_KM vs D) and salt-stressed samples vs. samples pretreated with KM followed by salt (S_KM vs S) with a log2 FC <-1. (D) GO enrichment of differentially expressed transcripts (FDR; $p<0.05$) in drought-stressed samples vs. samples pretreated with KM followed by drought (D_KM vs D) with a log2 FC <-1.

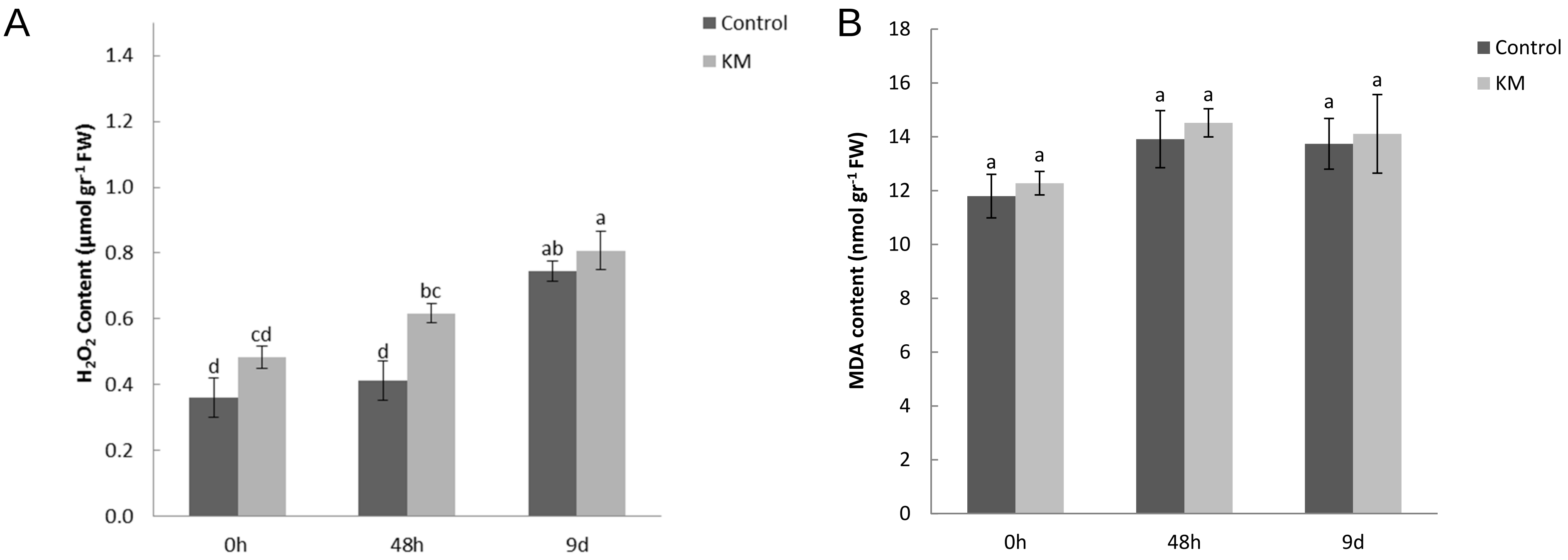

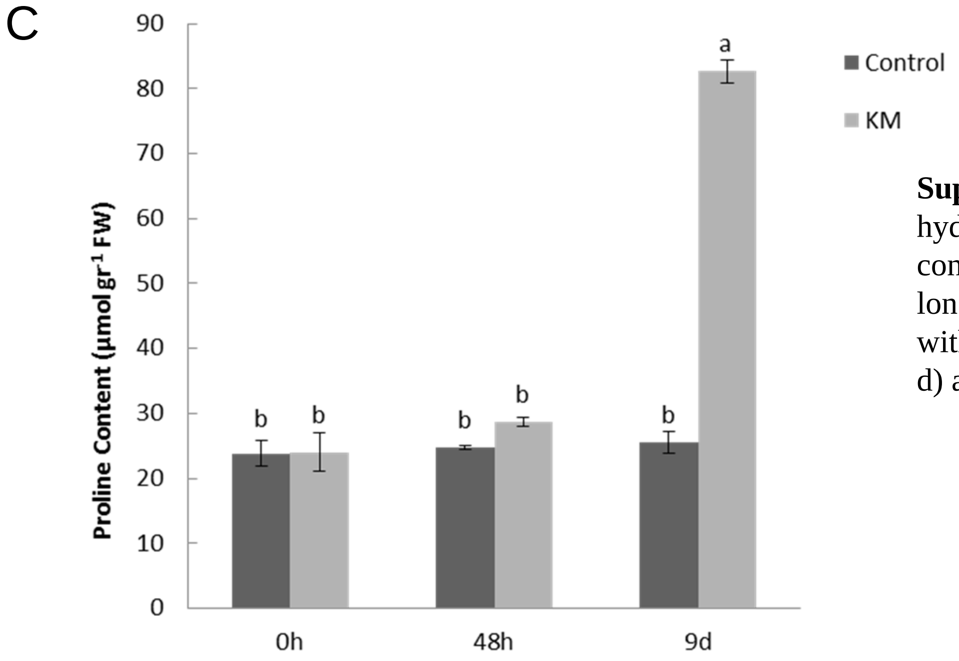

Supplementary Fig. S1. Effect of 10^{-8}M KM pre-treatment in (A) hydrogen peroxide content, (B) cellular damage indicated by leaf MDA content, (C) proline content in leaves of control (0 d), short (2 d) and long term (9 d) 10^{-8}M KM-treated *M. truncatula* plants. Data denoted with asterisks are statistically different to respective control samples (0 d) according to Tukey's pairwise comparison test ($p < 0.05$).

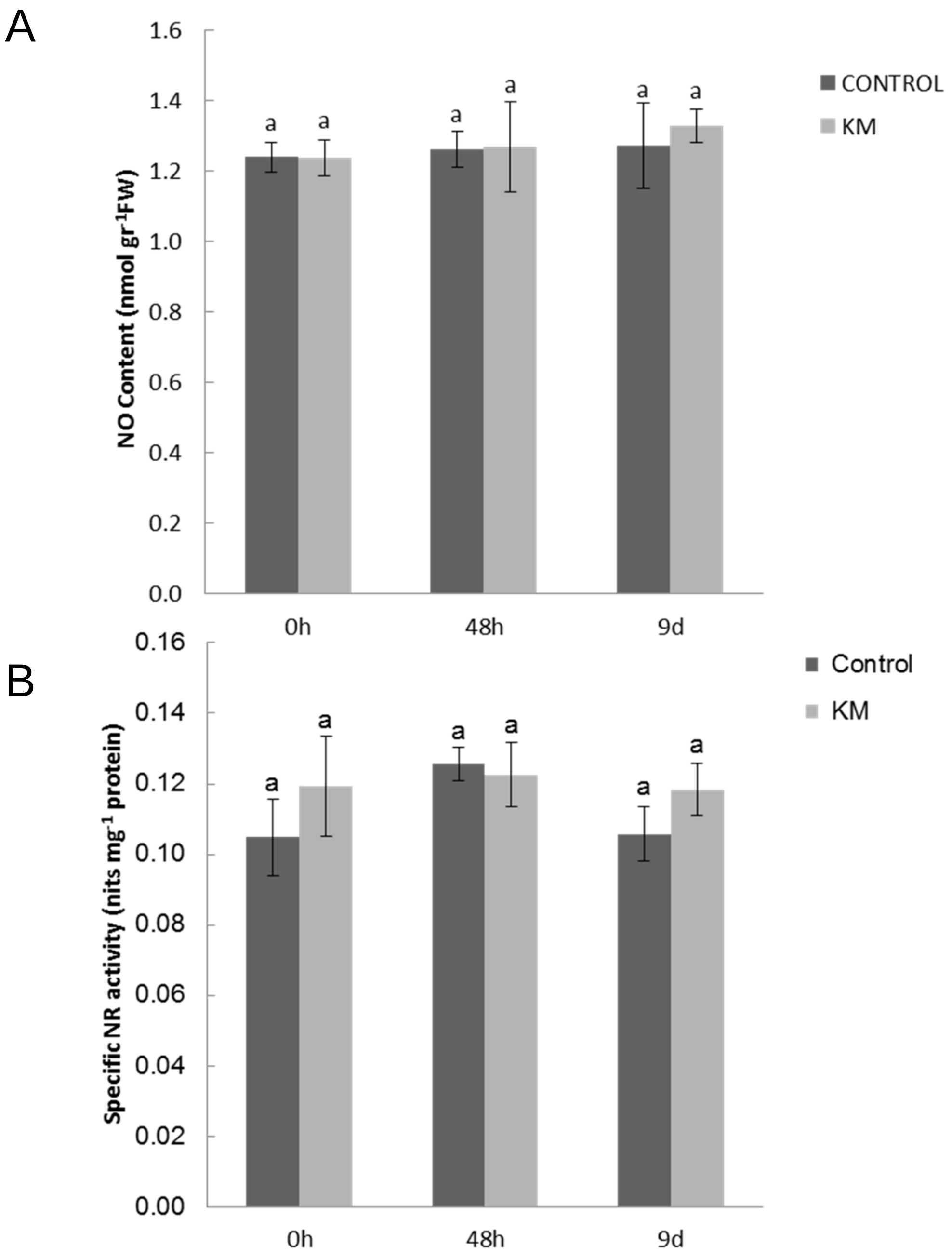

Supplementary Fig. S2. Effect of 10^{-8}M KM pre-treatment in (A) NO content and (B) nitrate reductase (NR) activity measurements in leaves of control (0 d), short (2 d) and long term (9 d) 10^{-8}M KM-treated *M. truncatula* plants. Data denoted with asterisks are statistically different to respective control samples (0 d) according to Tukey's pairwise comparison test ($p < 0.05$).

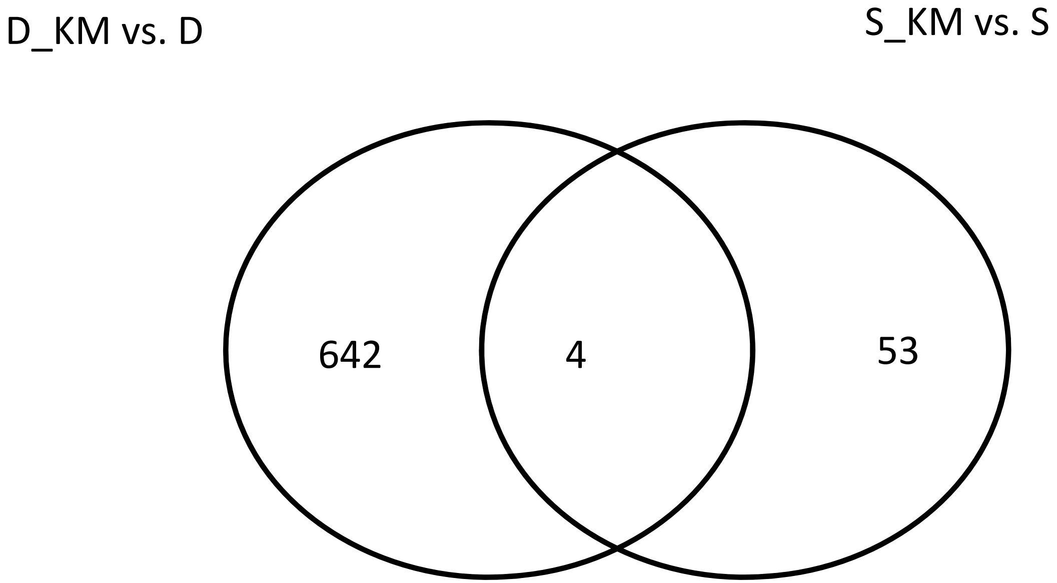

Supplementary Fig. S3. Venn diagram showing number of significantly regulated transcripts in drought-stressed samples vs. samples pre-treated with KM followed by drought (D_KM vs D) and salt-stressed samples vs. pre-treatment with KM and subsequent salt stress (S_KM vs S). Overlap signifies commonly regulated transcripts (FDR: $P<0.05$).

Supplementary Fig. S4. Metabolite profiling of drought (A) and salinity (B) stressed samples compared with respective drought and salinity stressed samples following 10^{-8} M KM pre-treatment in leaves of *M. truncatula* plants. (C) Metabolite profiling of pre-treated KM plants after 0 d (control), 2 d (short-term) and 9 d (long-term) KM foliar application in *M. truncatula* plants growing under normal conditions.

Supplementary Figure S4A

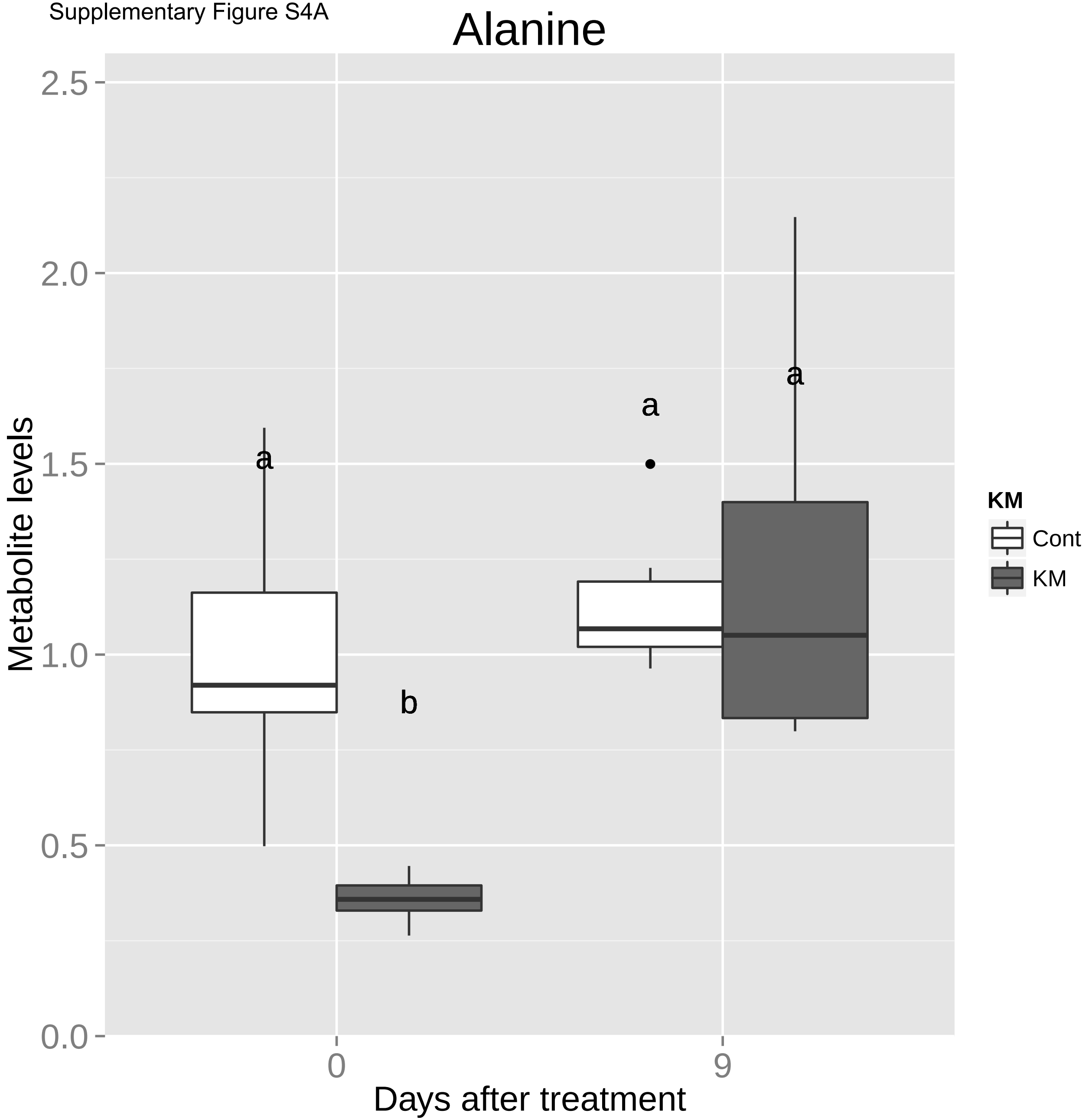

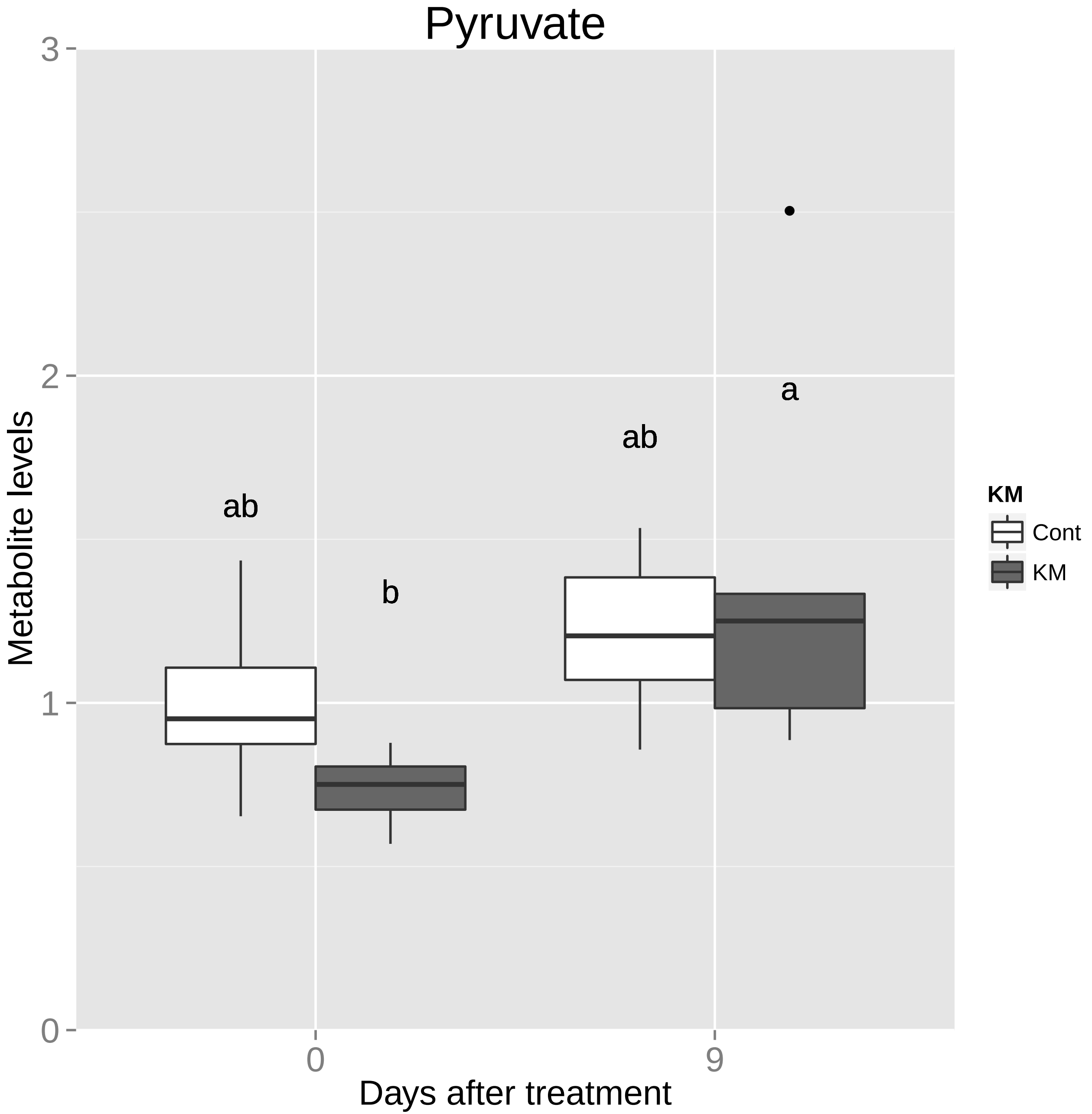

Pyruvate
3
2
1
0
Metabolite levels
ab
b
ab
a
0
9
Days after treatment
KM
Cont
KM

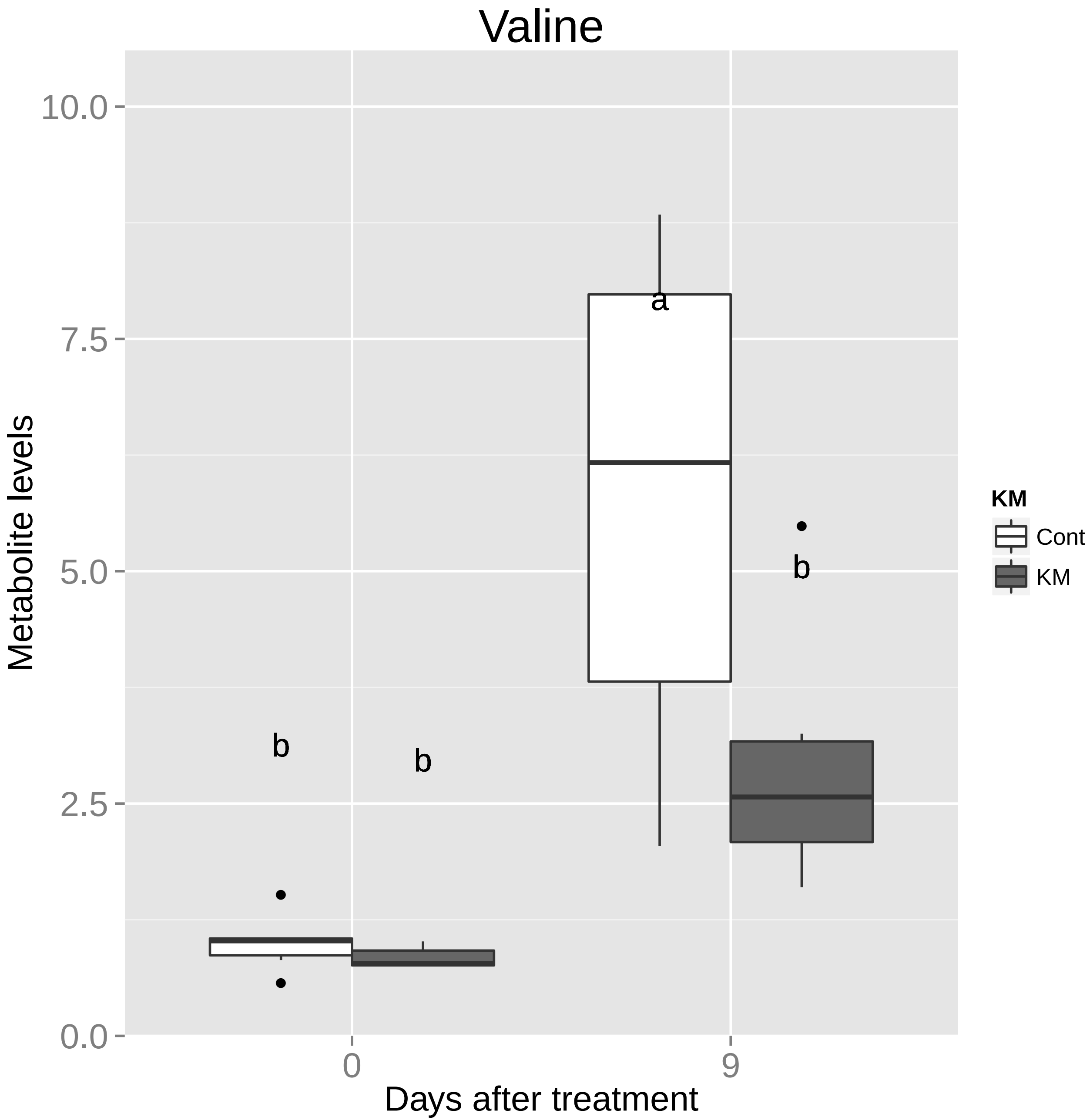
Valine
Metabolite levels
10.0
7.5
5.0
2.5
0.0
0
9
Days after treatment
a
b
b
b
KM
Cont
KM

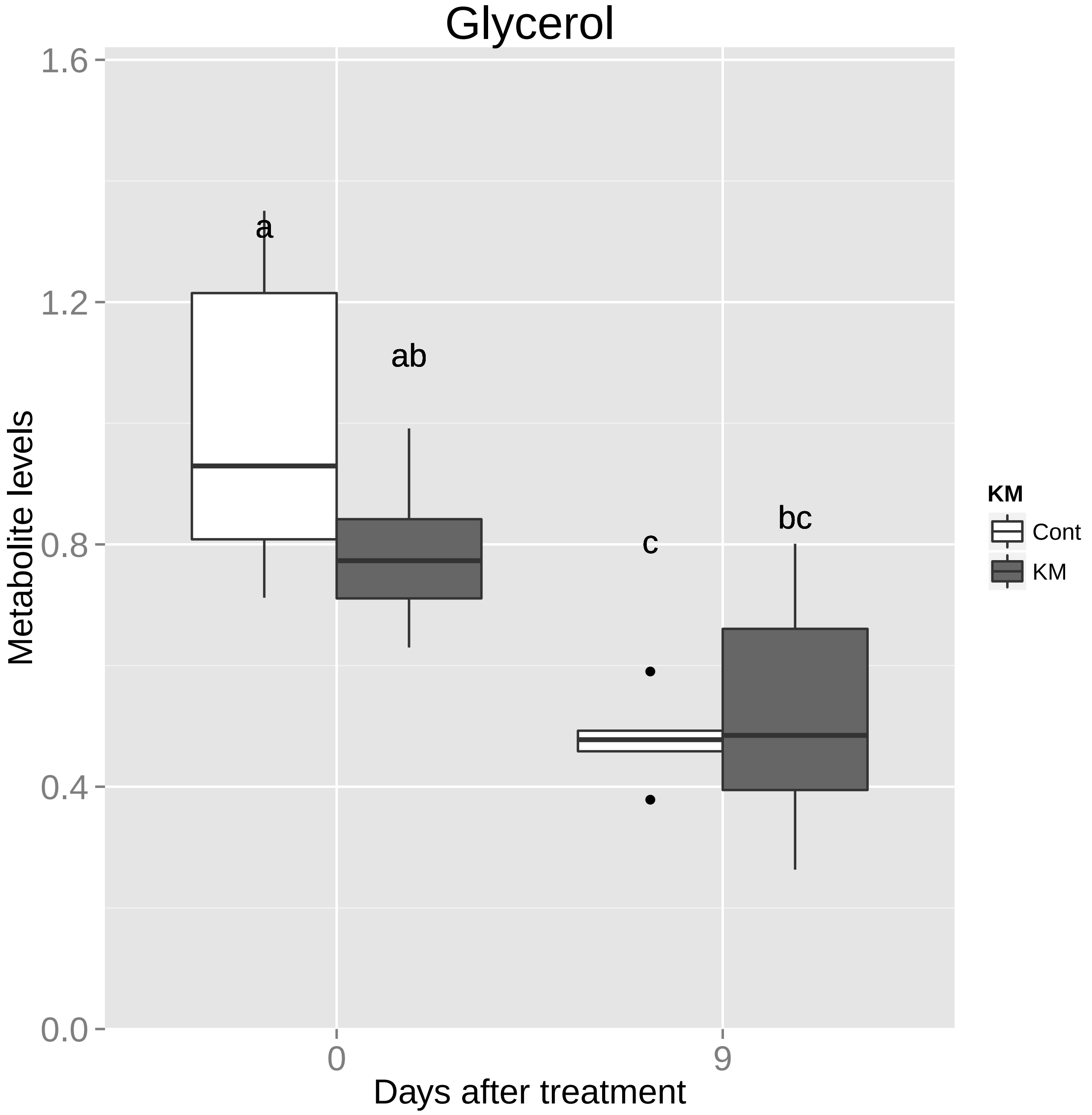

Glycerol
Metabolite levels
1.6
1.2
0.8
0.4
0.0
a
ab
c
bc
0
9
Days after treatment
KM
Cont
KM

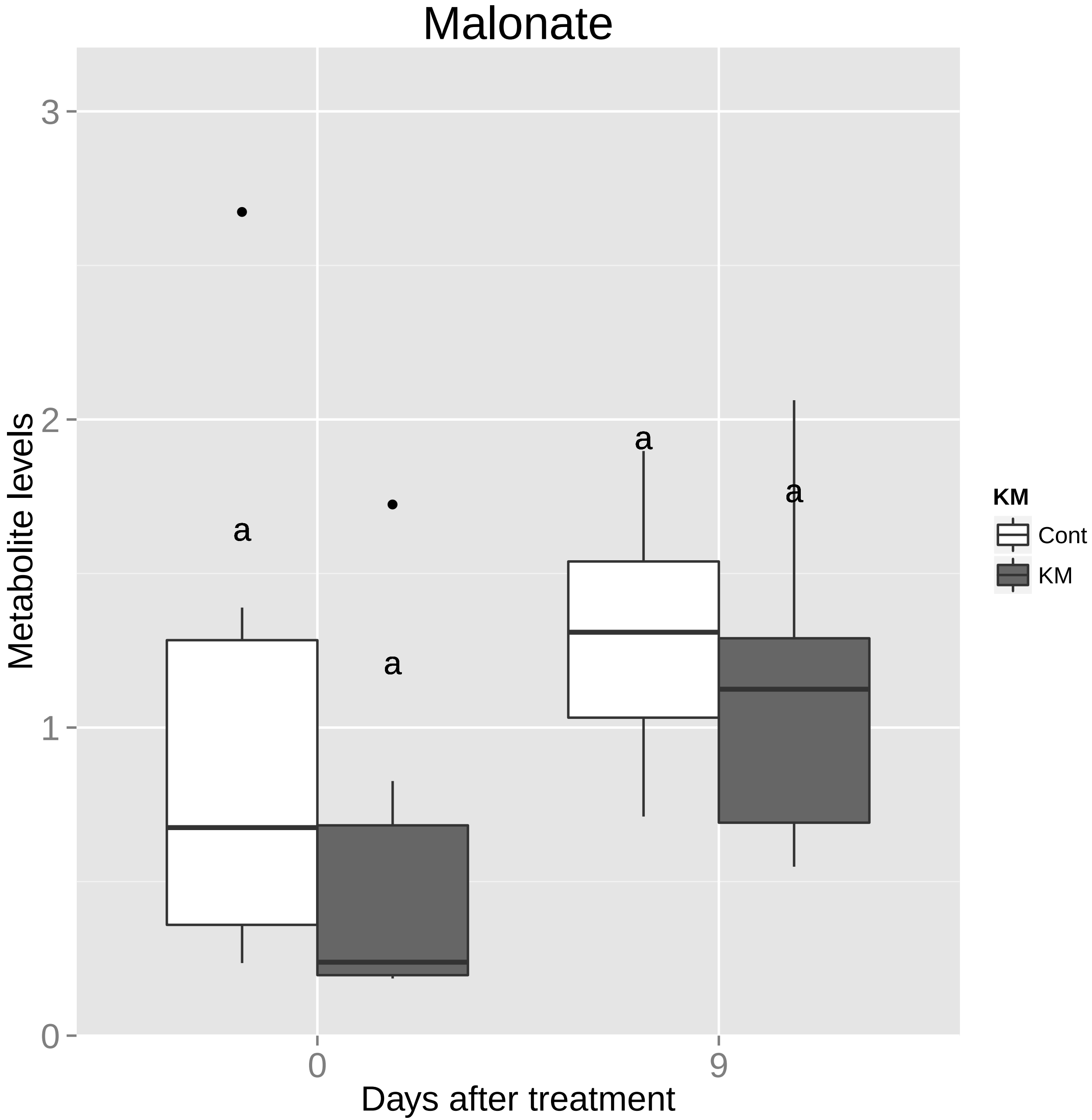
Malonate
Metabolite levels
Days after treatment
0
1
2
3
0
9
a
a
a
a
KM
Cont
KM

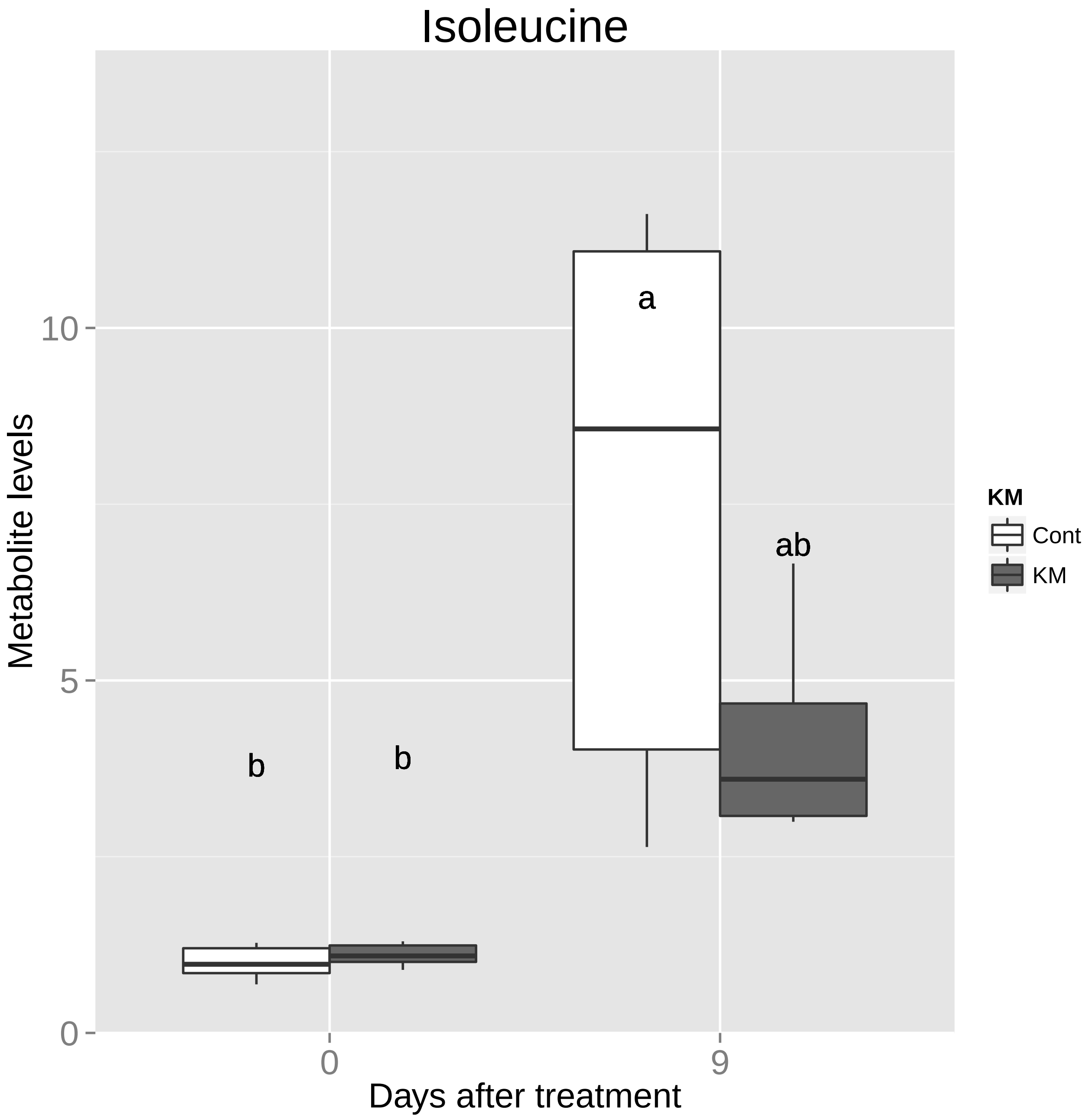

Isoleucine
Metabolite levels
10
5
0
0
9
Days after treatment
a
ab
b
b
KM
Cont
KM

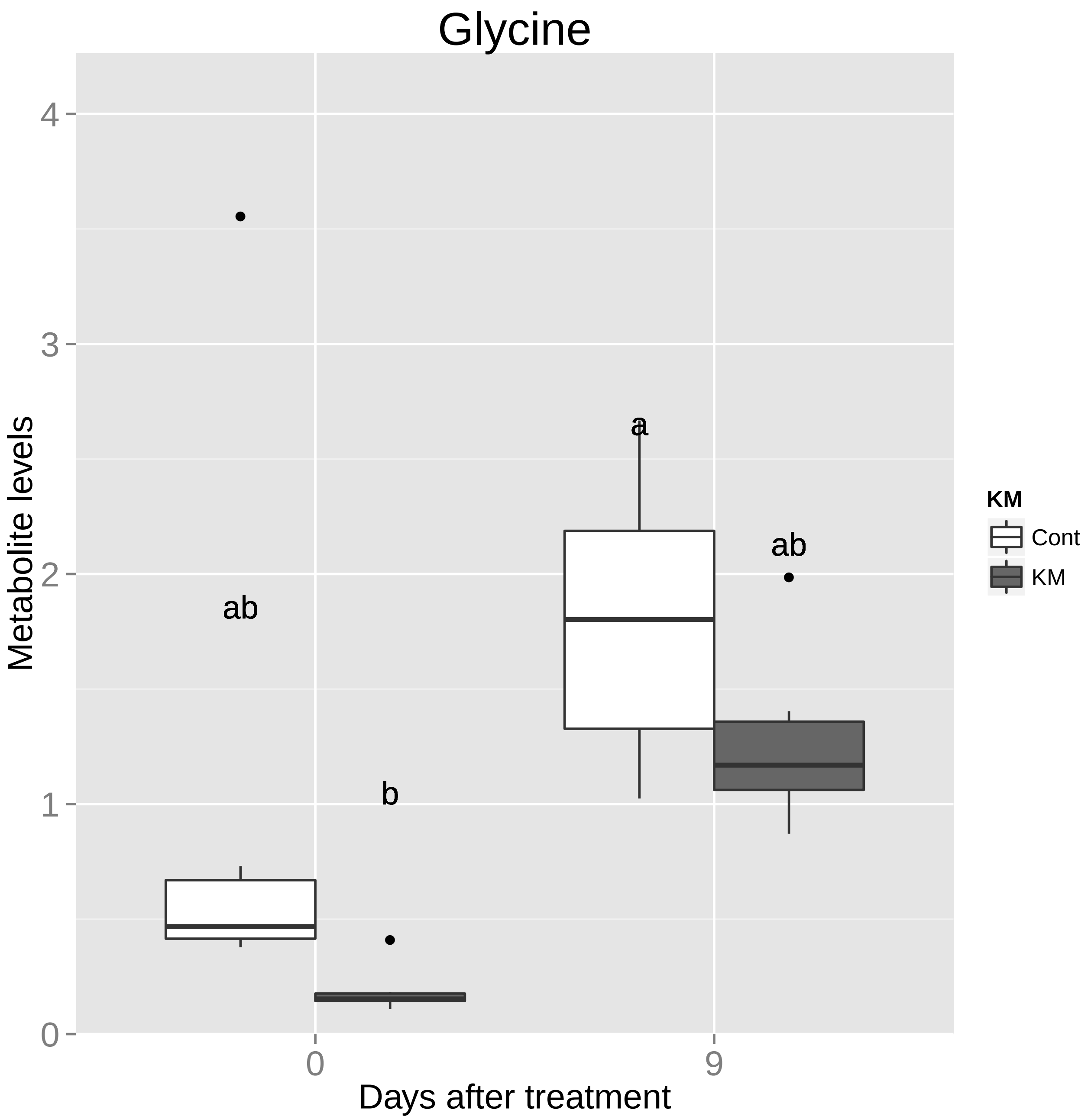
Glycine
Metabolite levels
0
1
2
3
4
ab
b
a
ab
0
9
Days after treatment
KM
Cont
KM

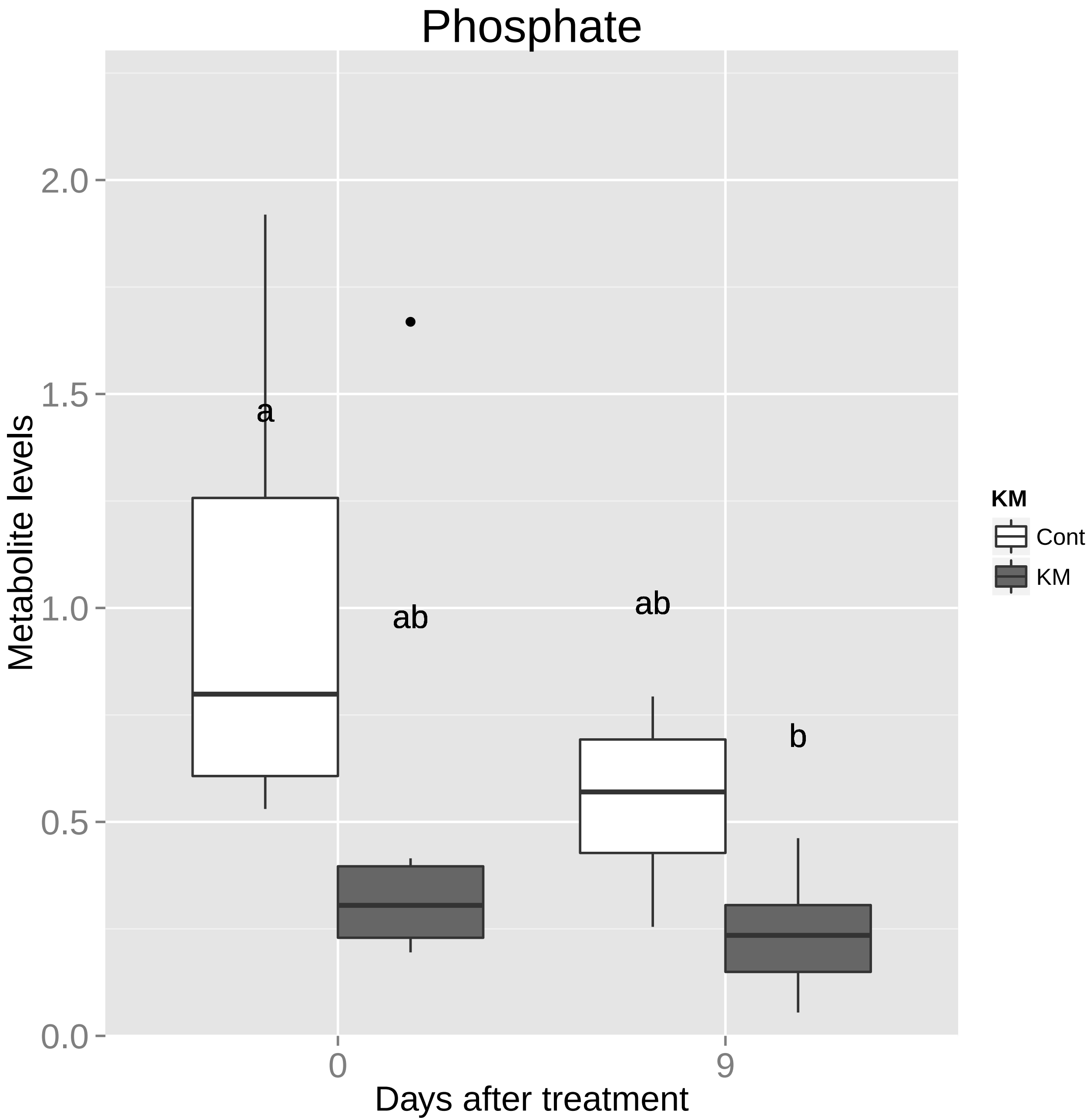

Phosphate
Metabolite levels
2.0
1.5
1.0
0.5
0.0
a
ab
ab
b
0
9
Days after treatment
KM
Cont
KM

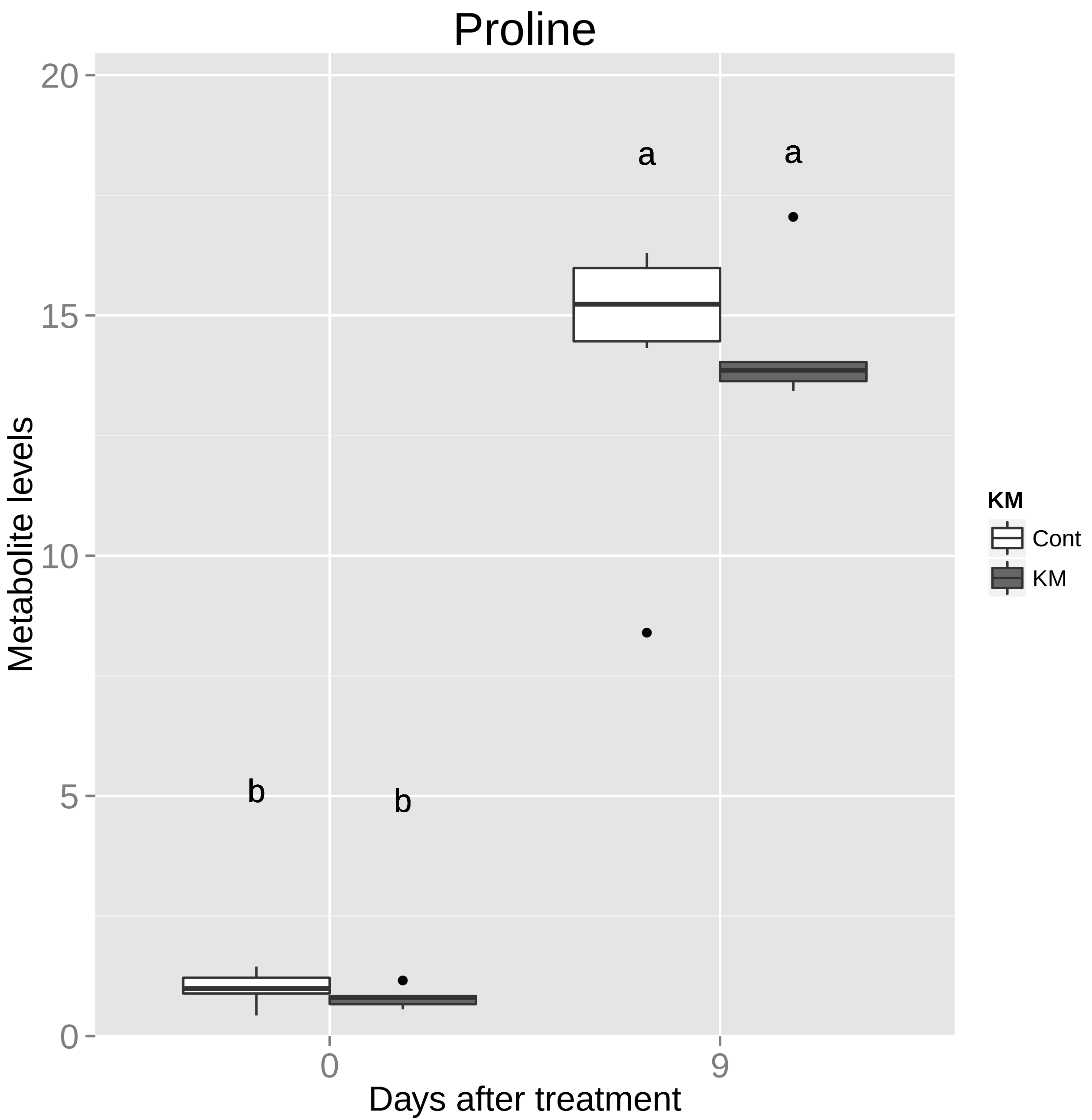

Proline
Metabolite levels
Days after treatment
20
15
10
5
0
0
9
a
a
b
b
KM
Cont
KM

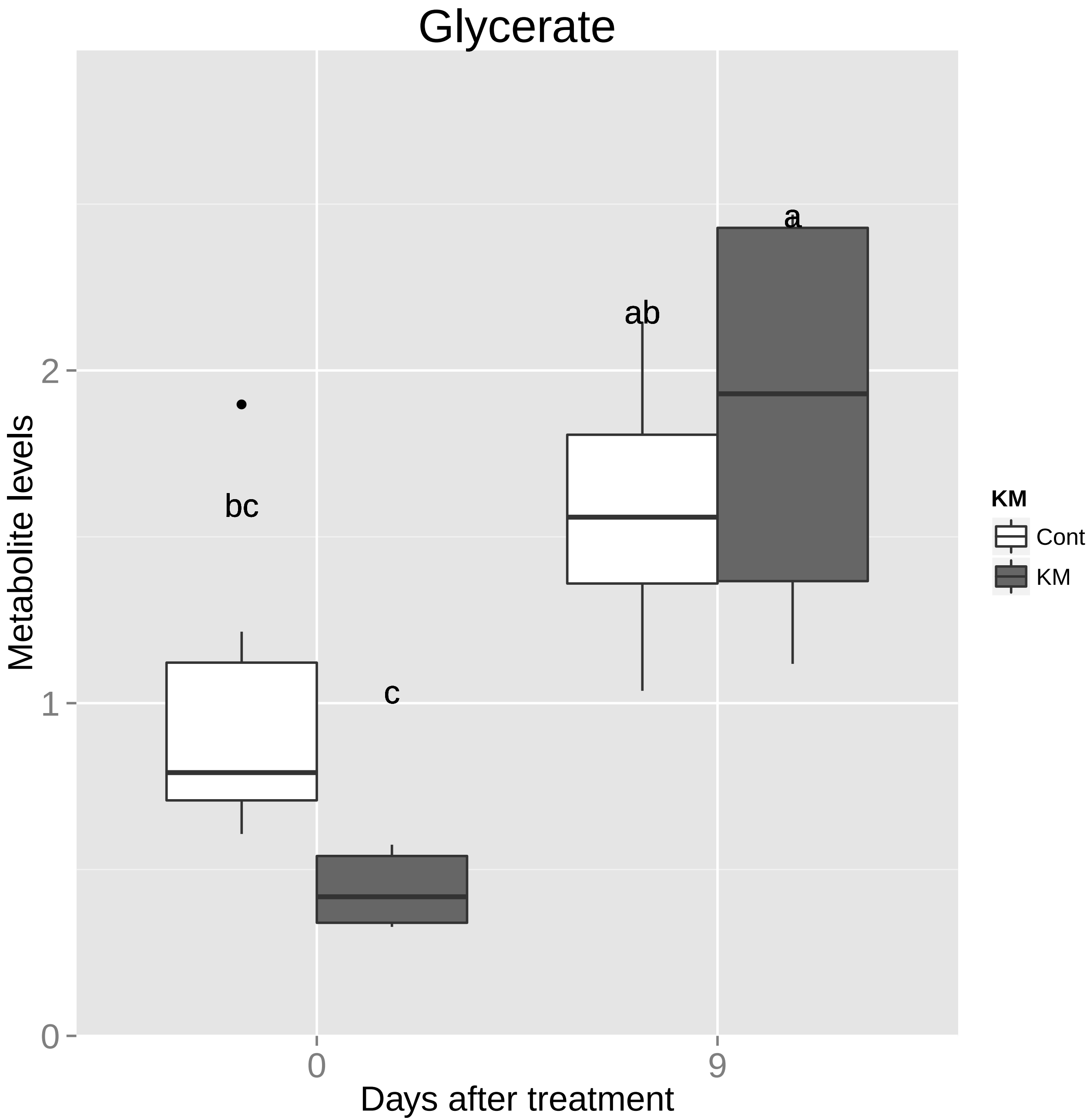
Glycerate
Metabolite levels
Days after treatment
0
1
2
0
9
bc
c
ab
a
KM
Cont
KM

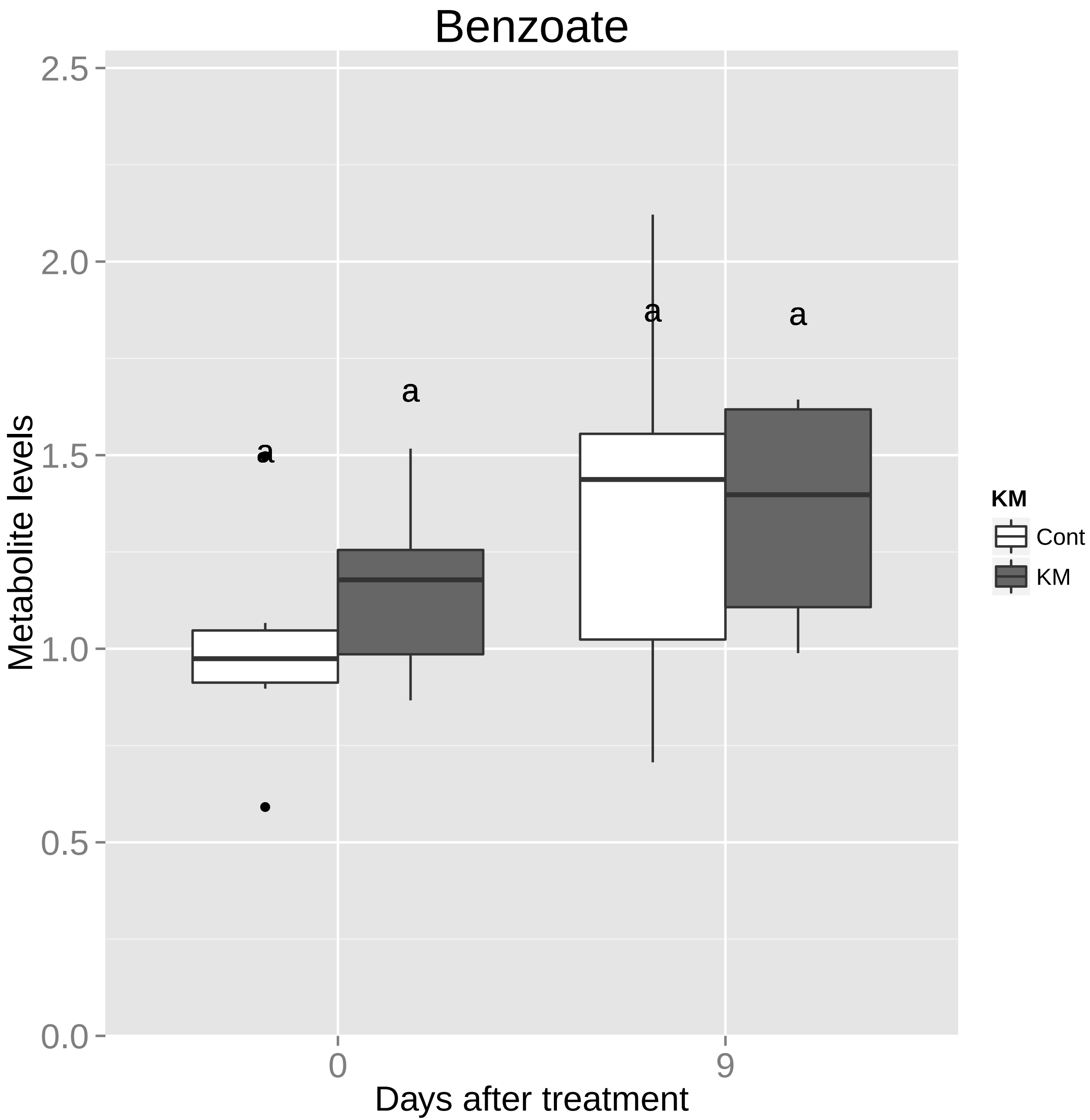
Benzoate
Metabolite levels
2.5
2.0
1.5
1.0
0.5
0.0
a
a
a
a
0
9
Days after treatment
KM
Cont
KM

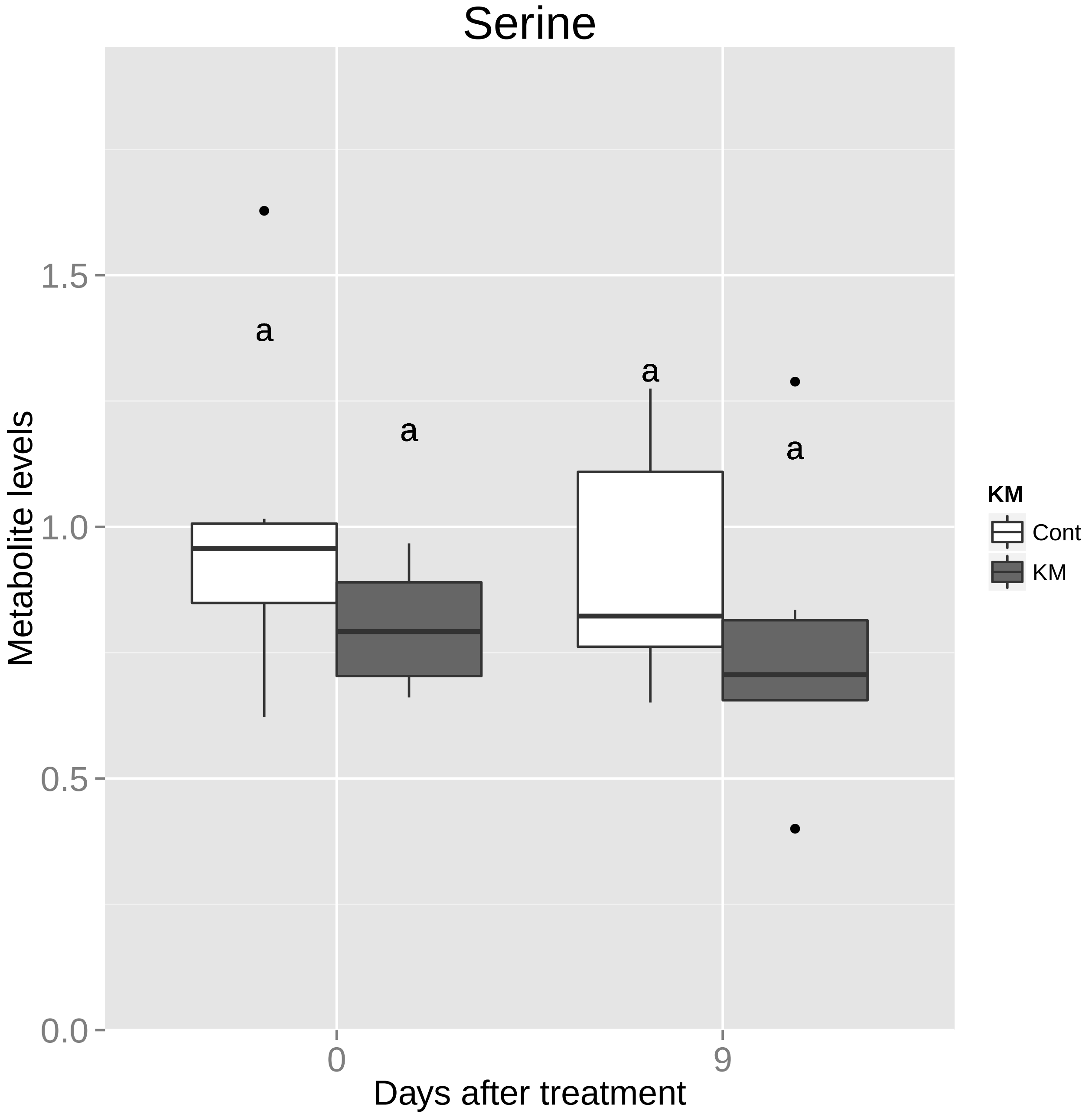
Serine
Metabolite levels
0.0
0.5
1.0
1.5
a
a
a
a
0
9
Days after treatment
KM
Cont
KM

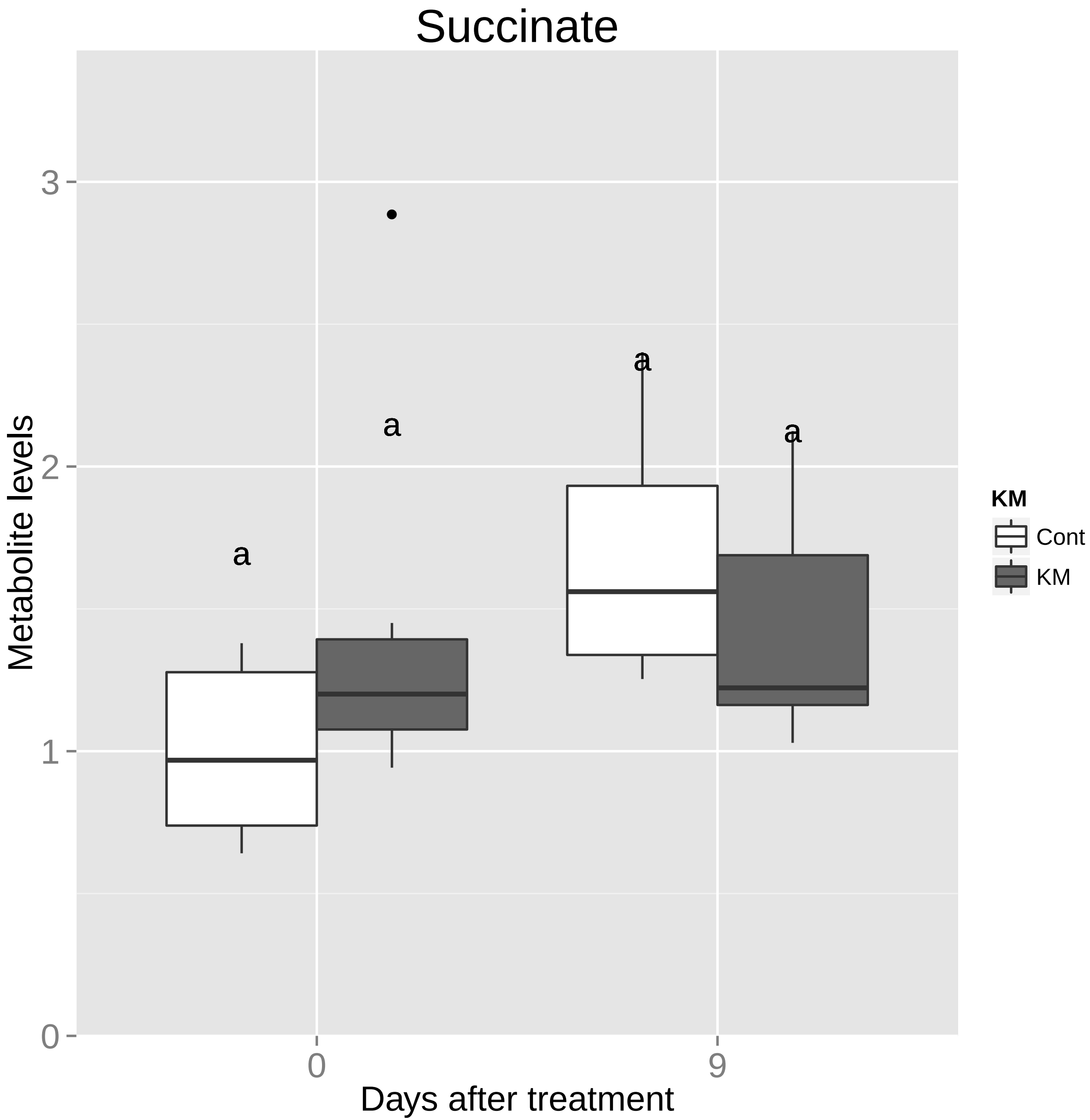
Succinate
Metabolite levels
Days after treatment
0
1
2
3
0
9
a
a
a
a
KM
Cont
KM

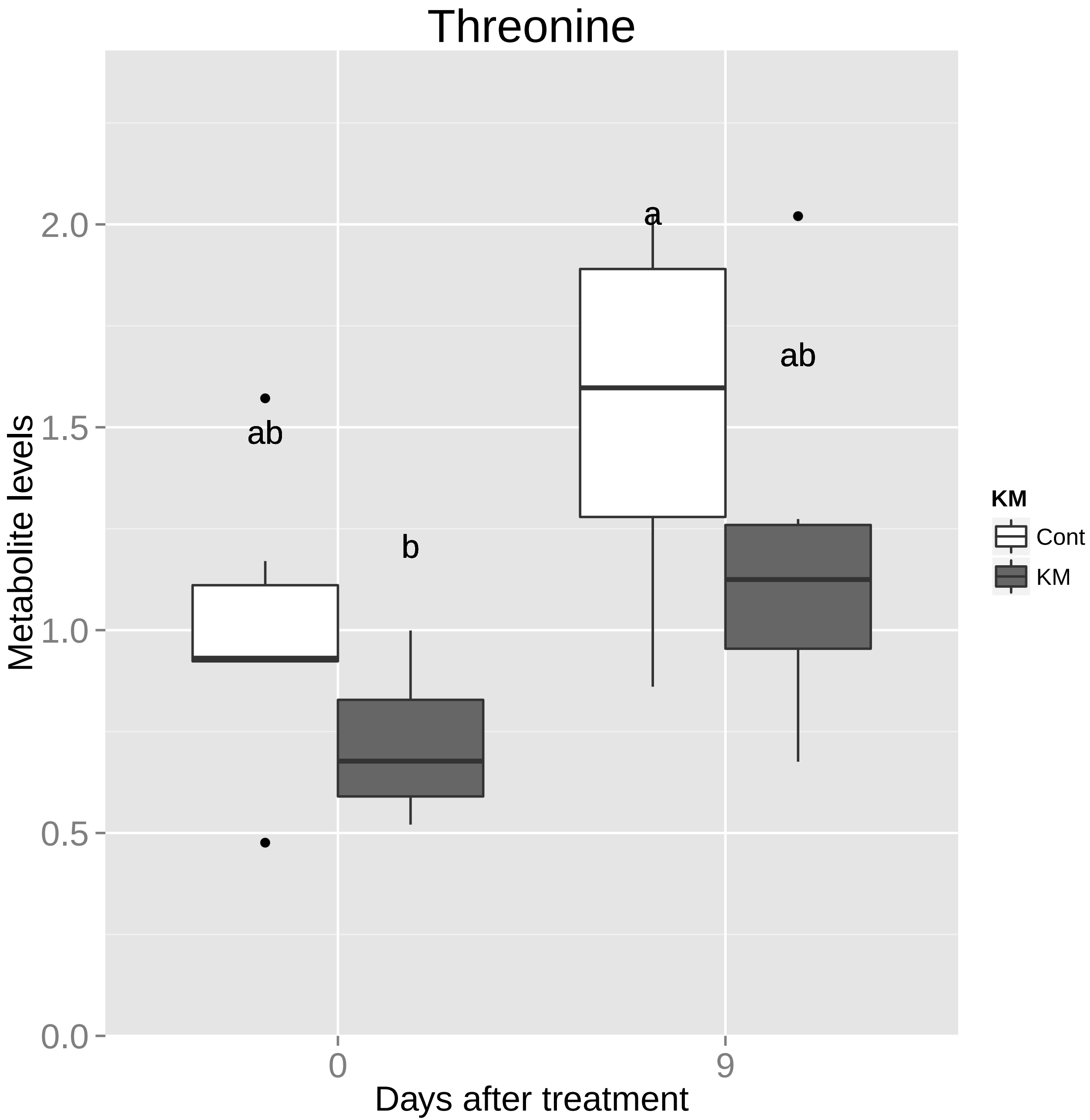
Threonine
Metabolite levels
0.0
0.5
1.0
1.5
2.0
0
9
Days after treatment
ab
b
a
ab
KM
Cont
KM

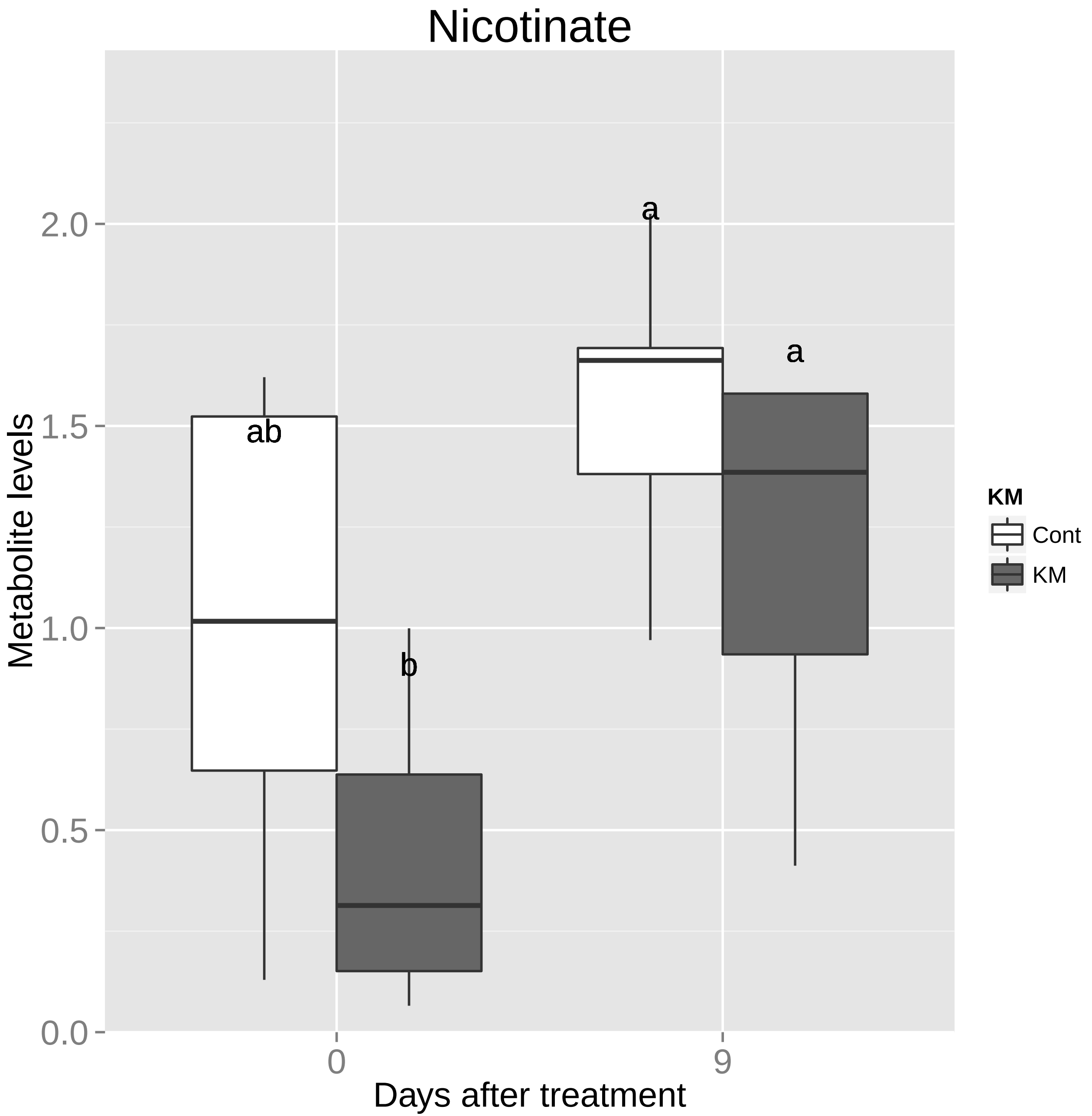
Nicotinate
Metabolite levels
2.0
1.5
1.0
0.5
0.0
ab
b
a
a
0
9
Days after treatment
KM
Cont
KM

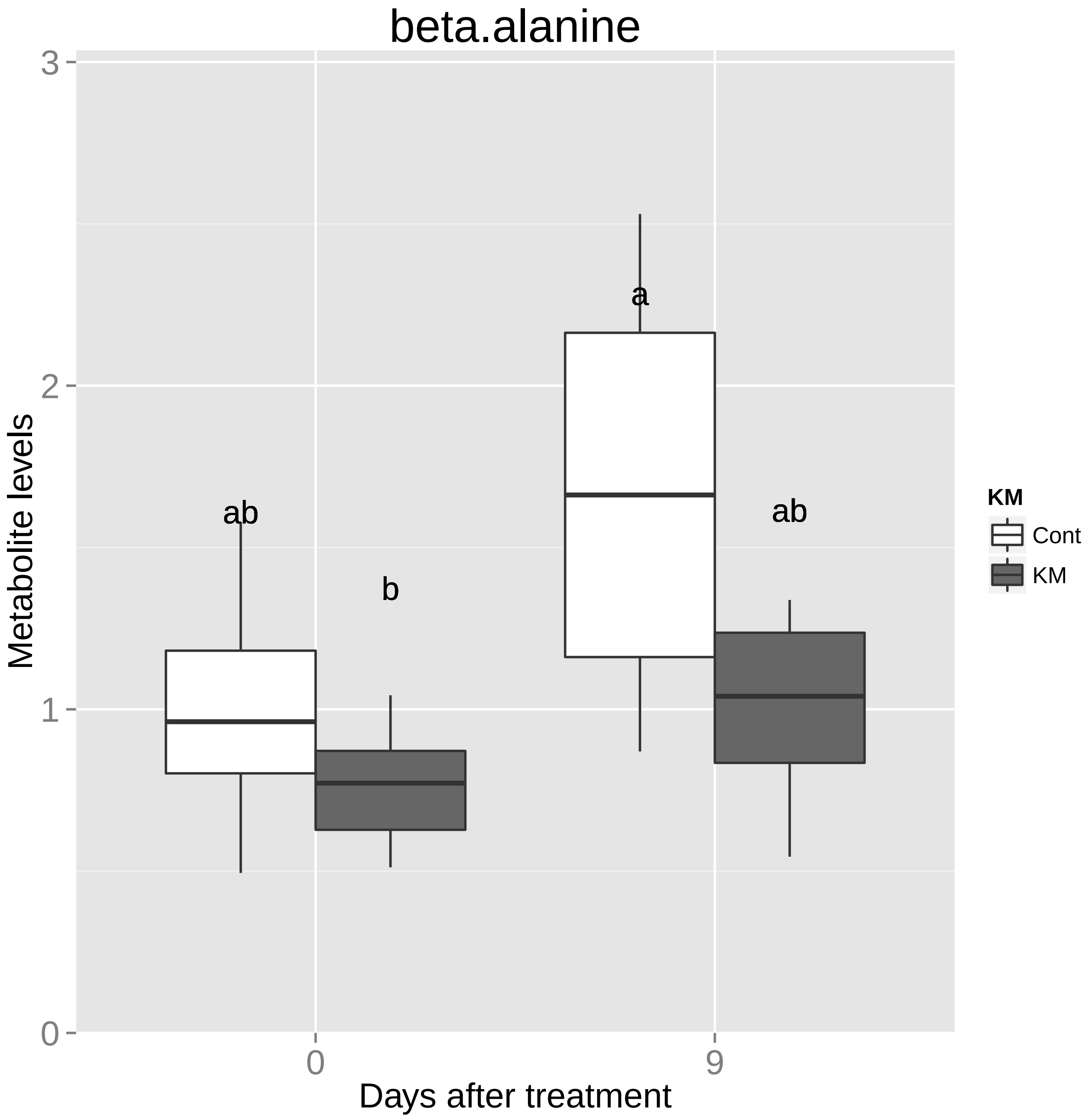

beta.alanine
Metabolite levels
3
2
1
0
a
ab
b
ab
0
9
Days after treatment
KM
Cont
KM

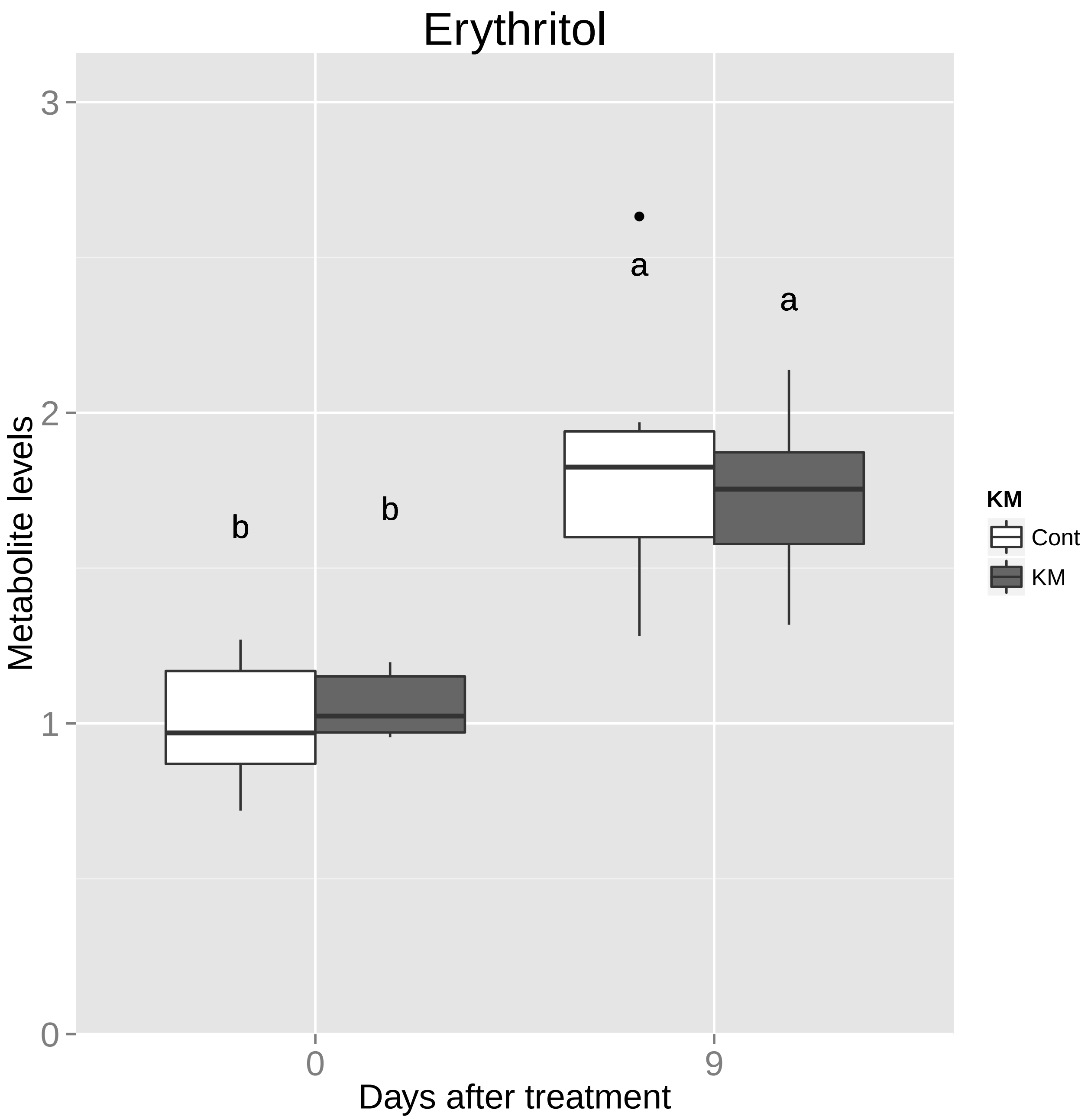

Erythritol
Metabolite levels
3
2
1
0
a
a
b
b
0
9
Days after treatment
KM
Cont
KM

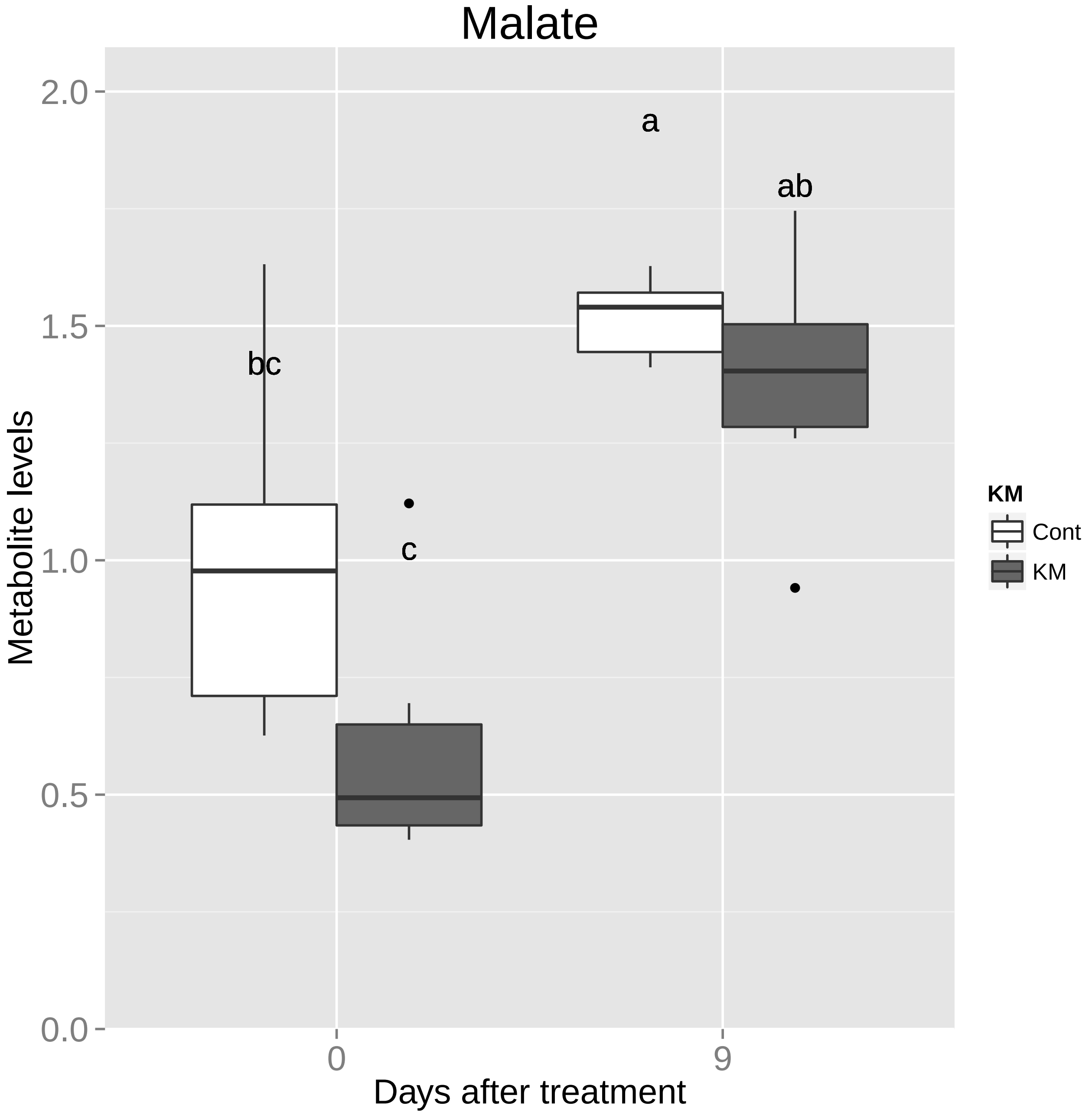
Malate
Metabolite levels
2.0
1.5
1.0
0.5
0.0
a
ab
bc
c
0
9
Days after treatment
KM
Cont
KM

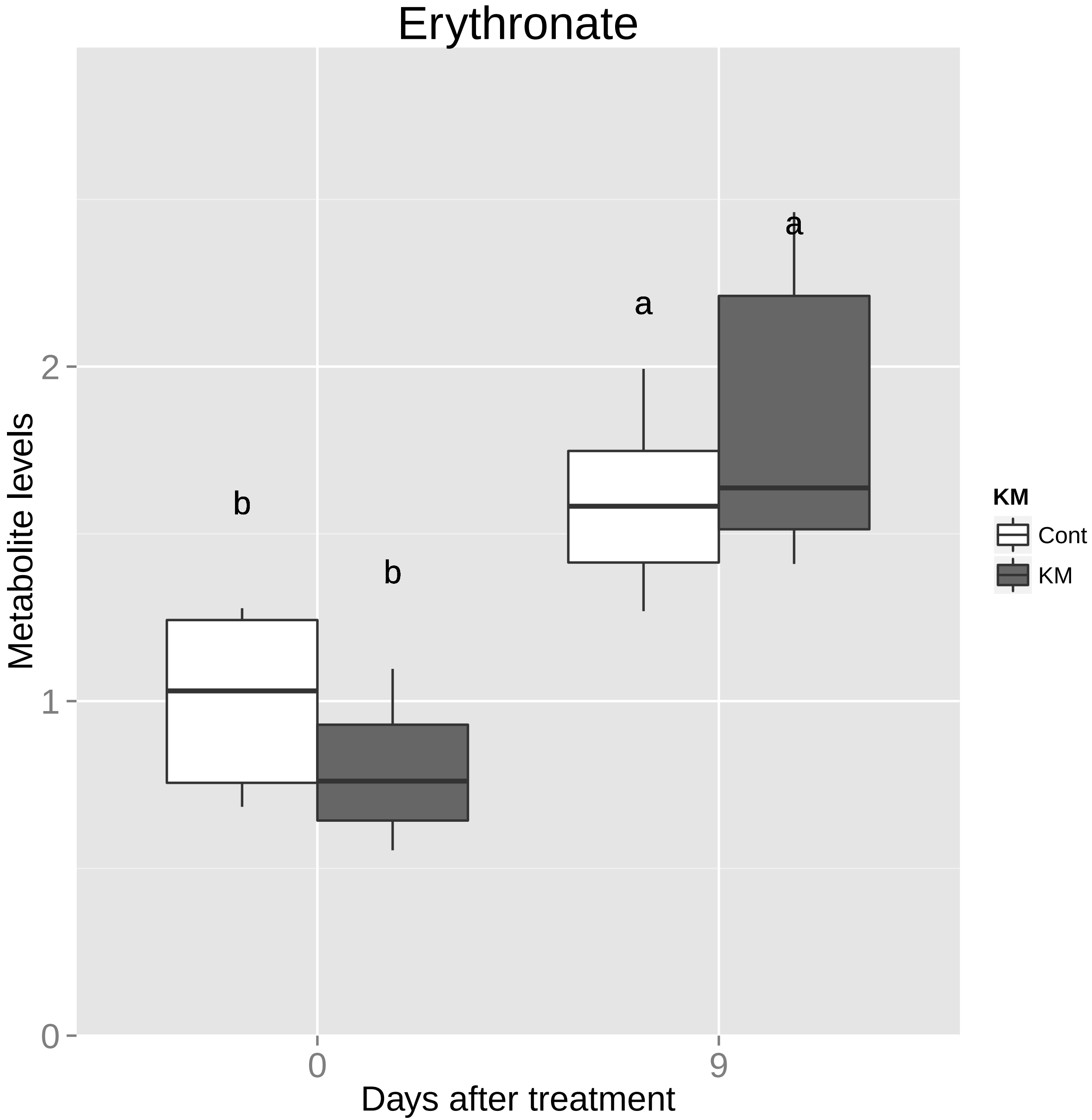
Erythronate
Metabolite levels
0
1
2
b
b
a
a
0
9
Days after treatment
KM
Cont
KM

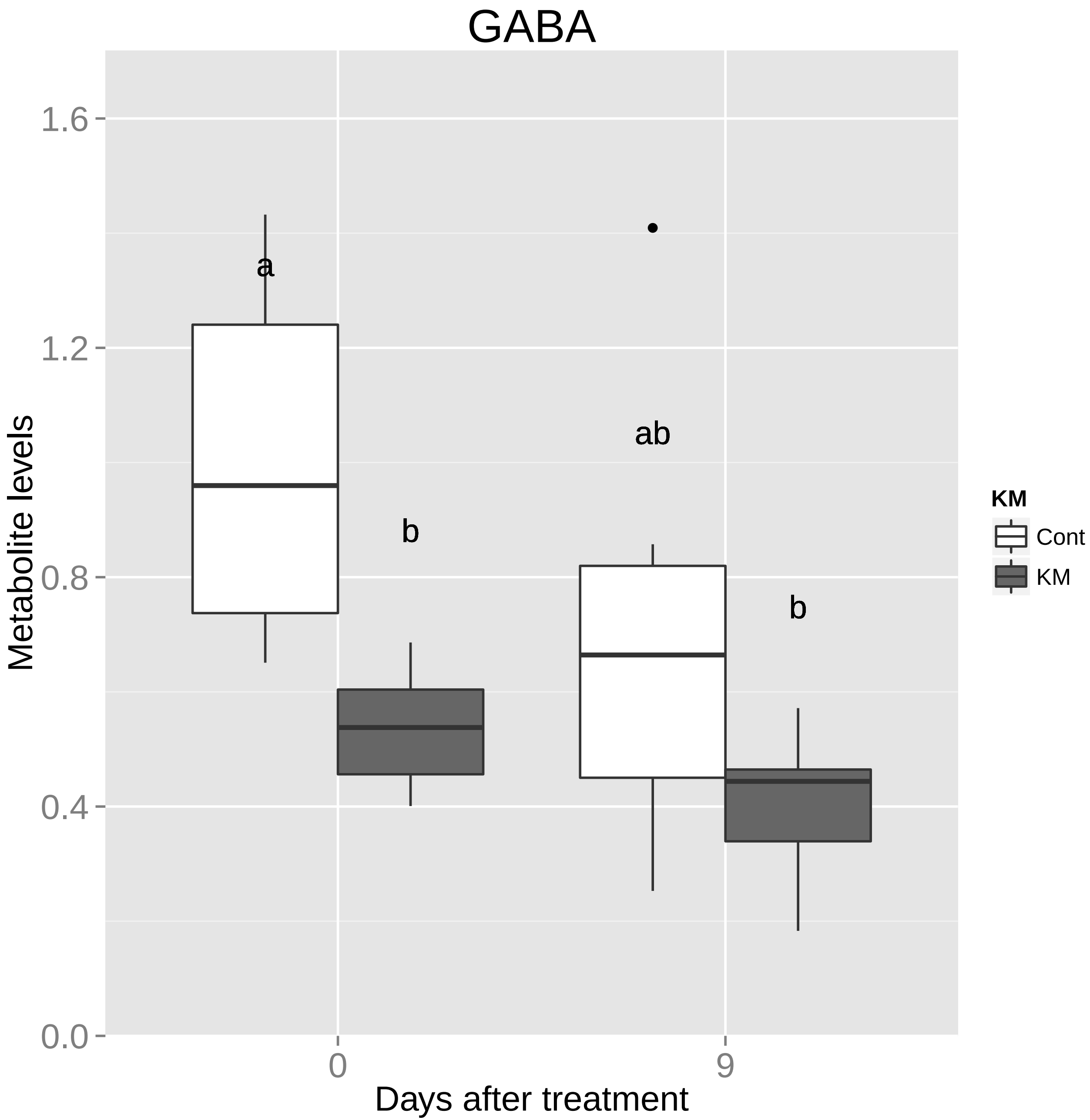
GABA
Metabolite levels
1.6
1.2
0.8
0.4
0.0
a
b
ab
b
0
9
Days after treatment
KM
Cont
KM

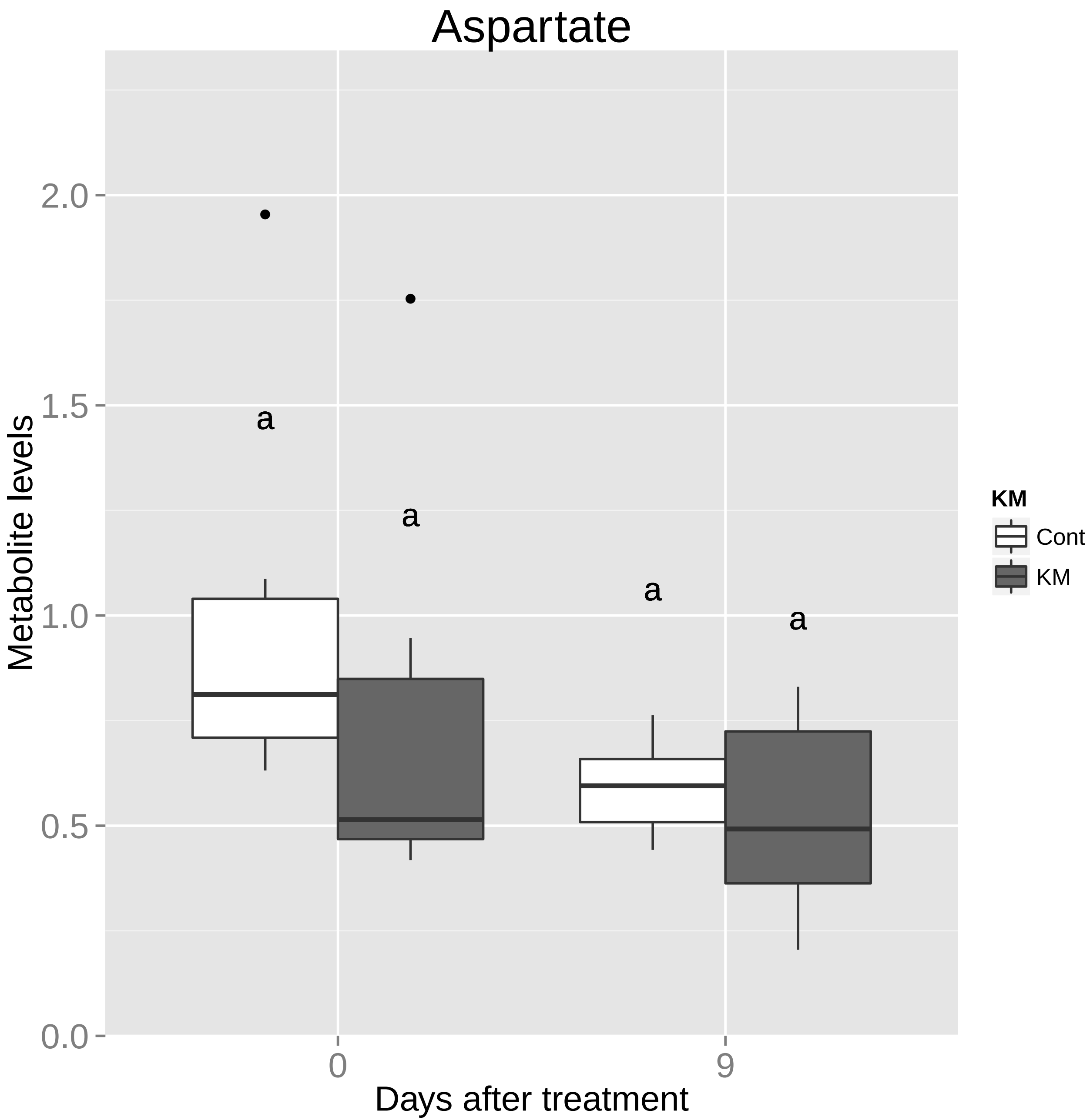

Aspartate
Metabolite levels
0.0
0.5
1.0
1.5
2.0
a
a
a
a
0
9
Days after treatment
KM
Cont
KM

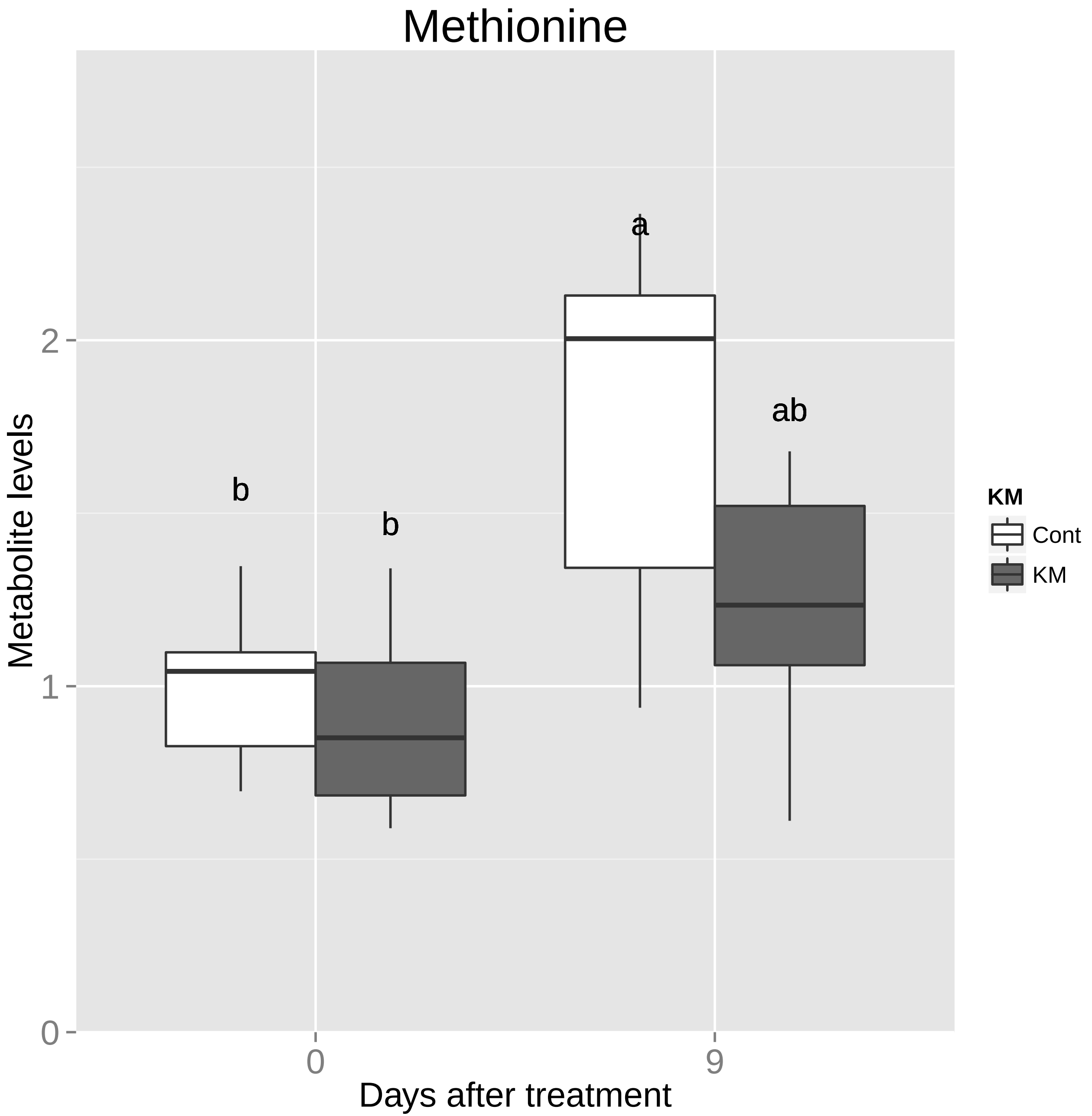
Methionine
Metabolite levels
Days after treatment
0
1
2
0
9
a
ab
b
b
KM
Cont
KM

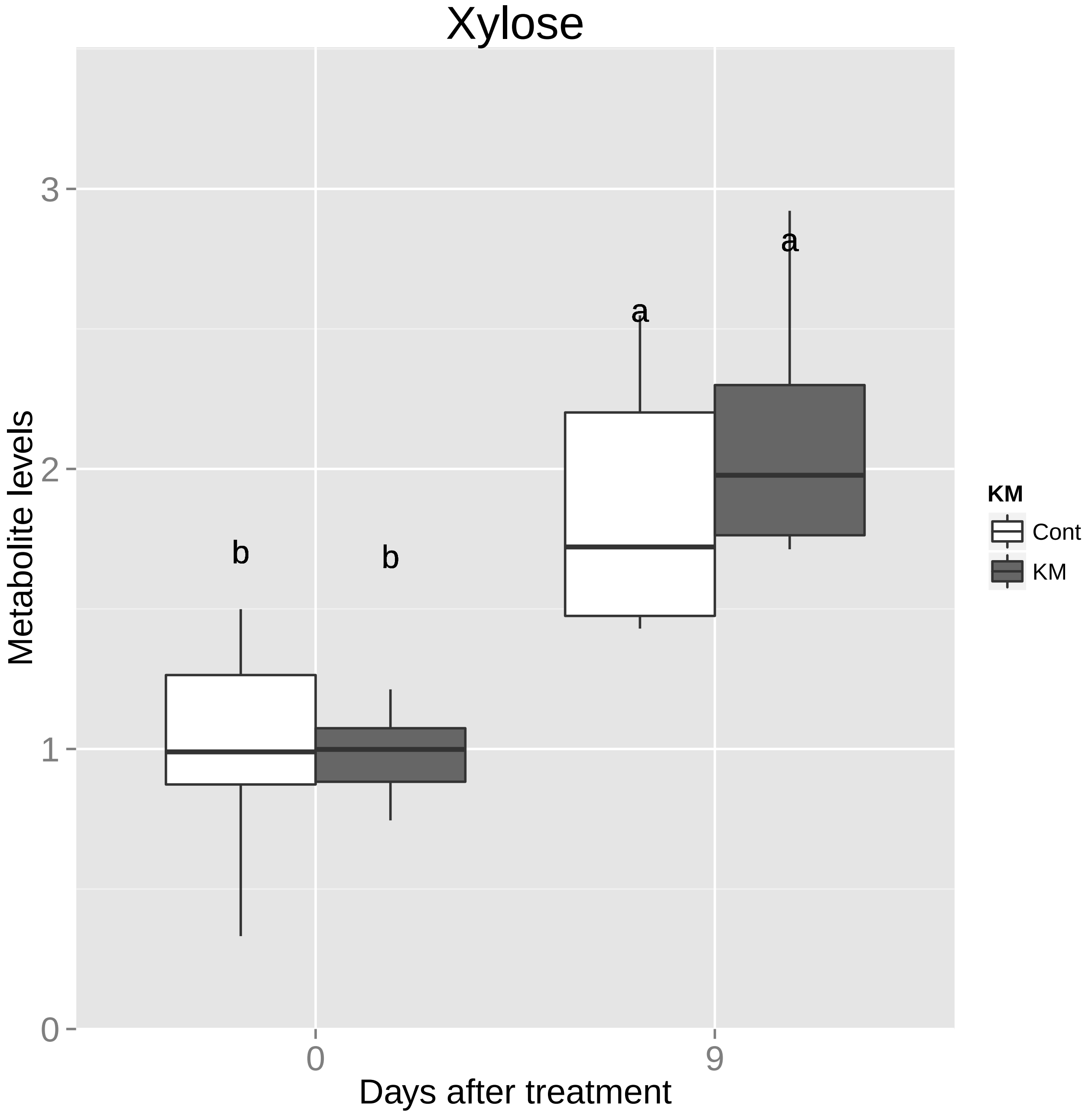
Xylose
Metabolite levels
Days after treatment
0
1
2
3
0
9
b
b
a
a
KM
Cont
KM

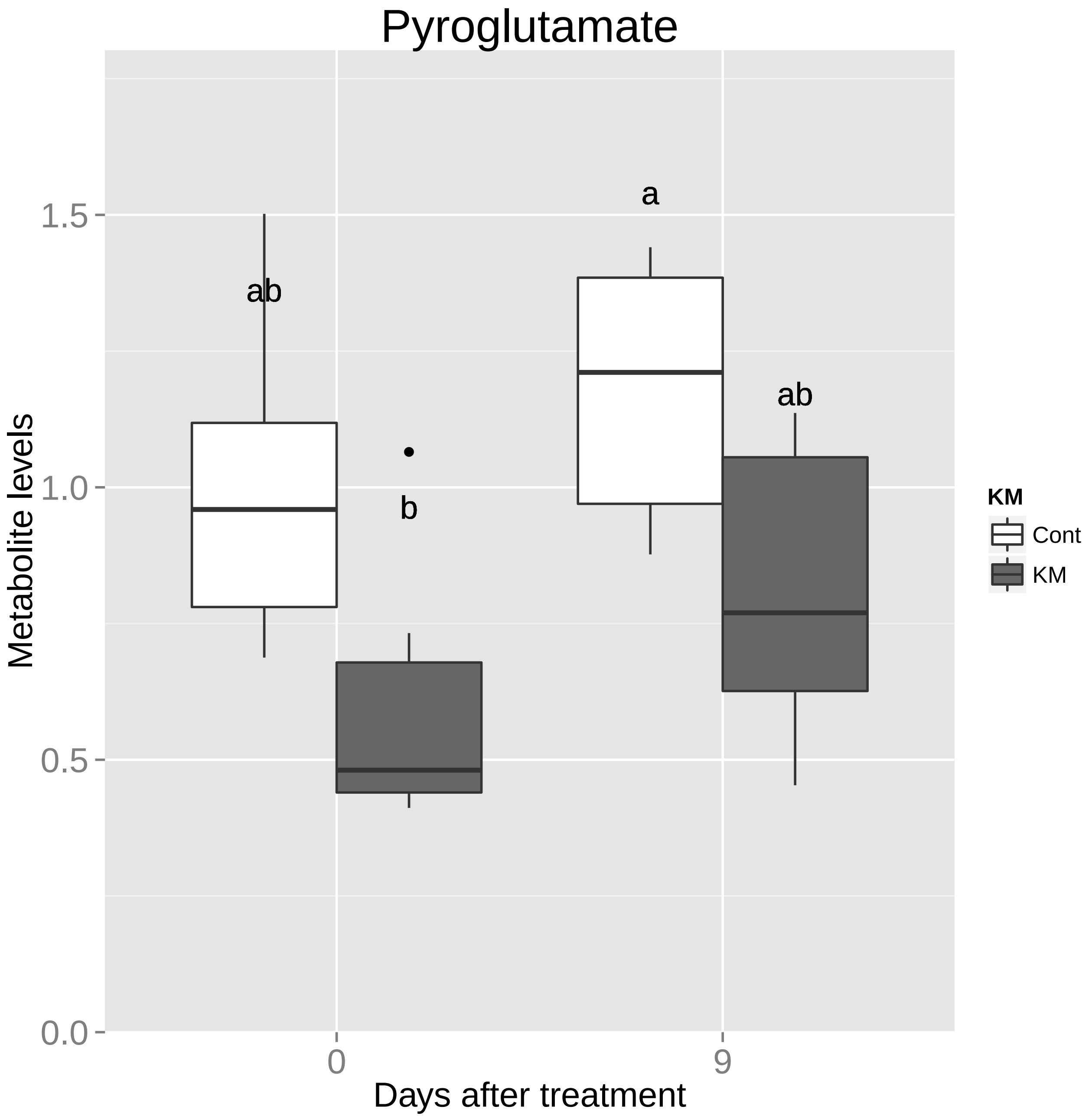

Pyroglutamate
Metabolite levels
Days after treatment
0.0
0.5
1.0
1.5
0
9
ab
b
a
ab
KM
Cont
KM

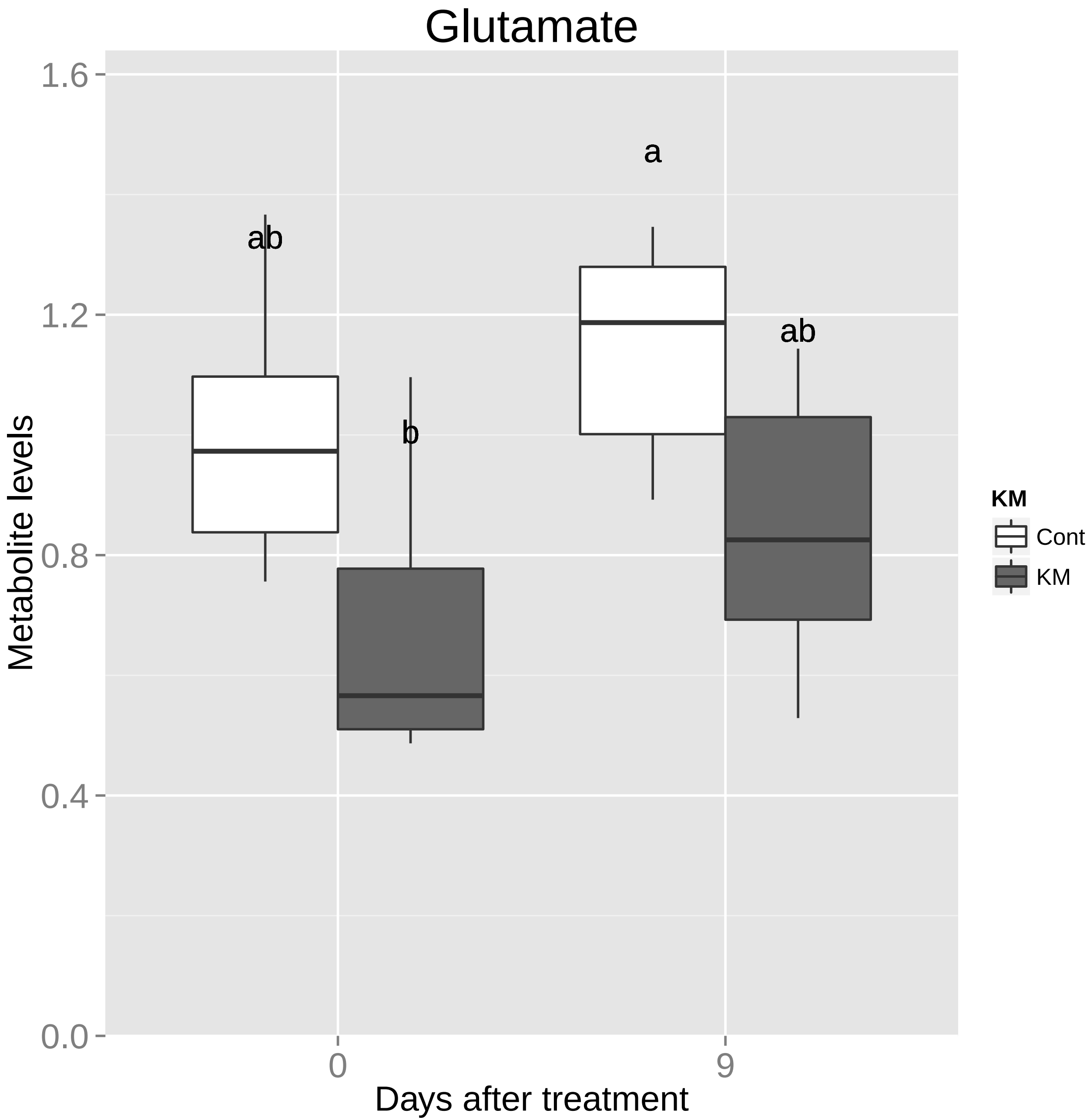

Glutamate
1.6
1.2
0.8
0.4
0.0
Metabolite levels
ab
b
a
ab
0
9
Days after treatment
KM
Cont
KM

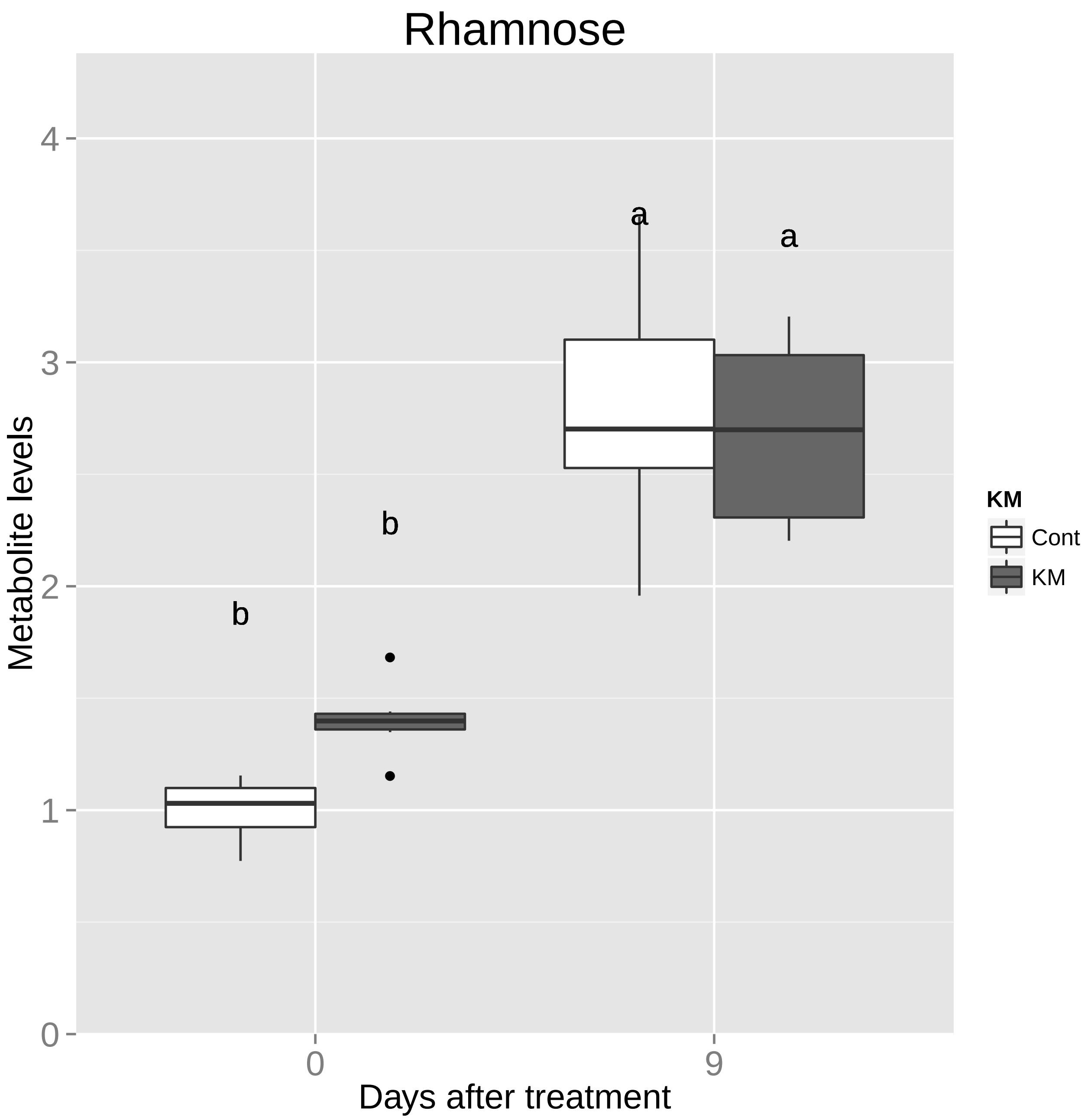
Rhamnose
Metabolite levels
Days after treatment
0
1
2
3
4
0
9
a
a
b
b
KM
Cont
KM

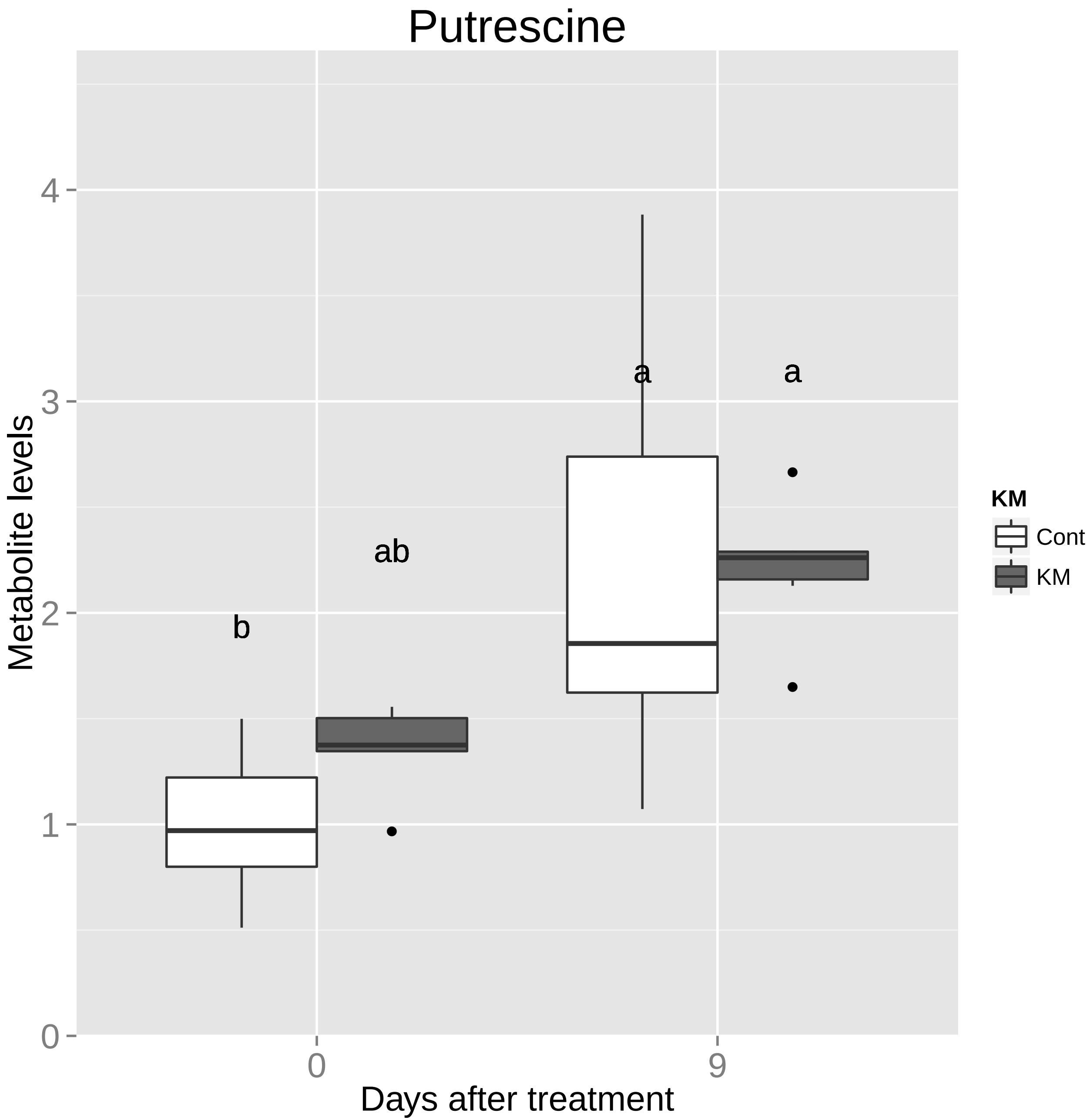

Putrescine
Metabolite levels
Days after treatment
0
1
2
3
4
0
9
b
ab
a
a
KM
Cont
KM

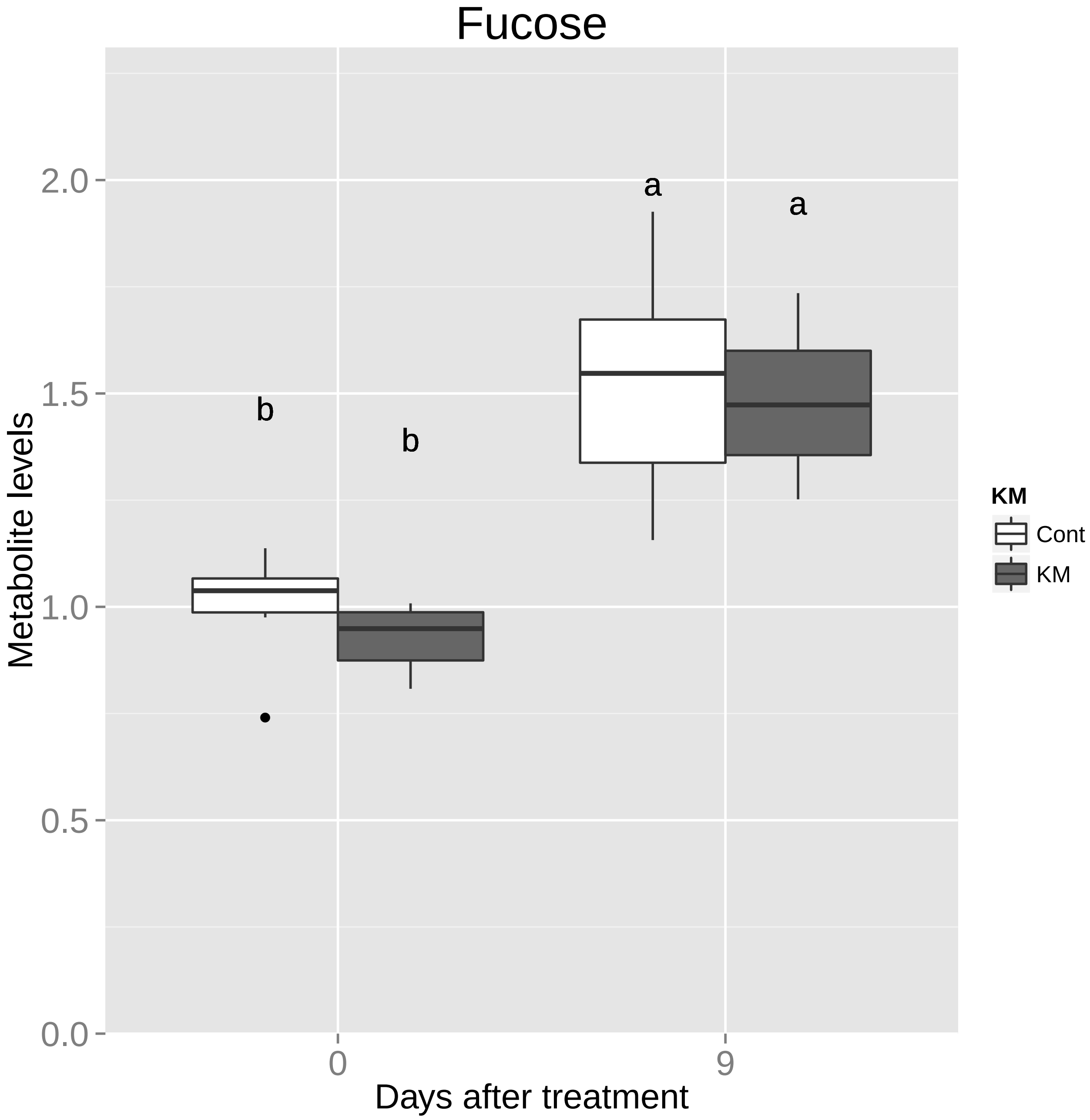
Fucose
Metabolite levels
Days after treatment
2.0
1.5
1.0
0.5
0.0
0
9
b
b
a
a
KM
Cont
KM

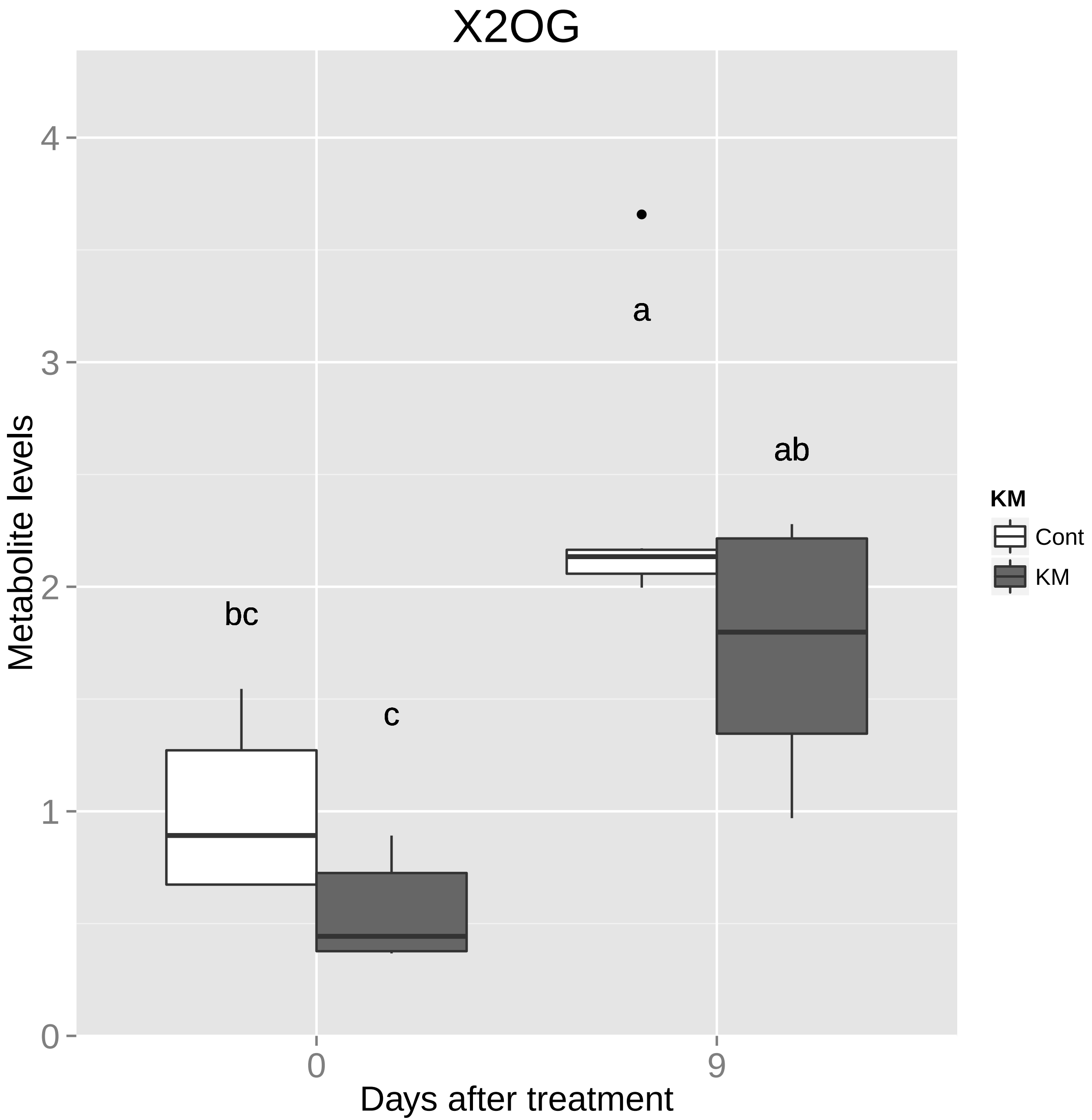

X2OG
Metabolite levels
4
3
2
1
0
a
ab
bc
c
0
9
Days after treatment
KM
Cont
KM

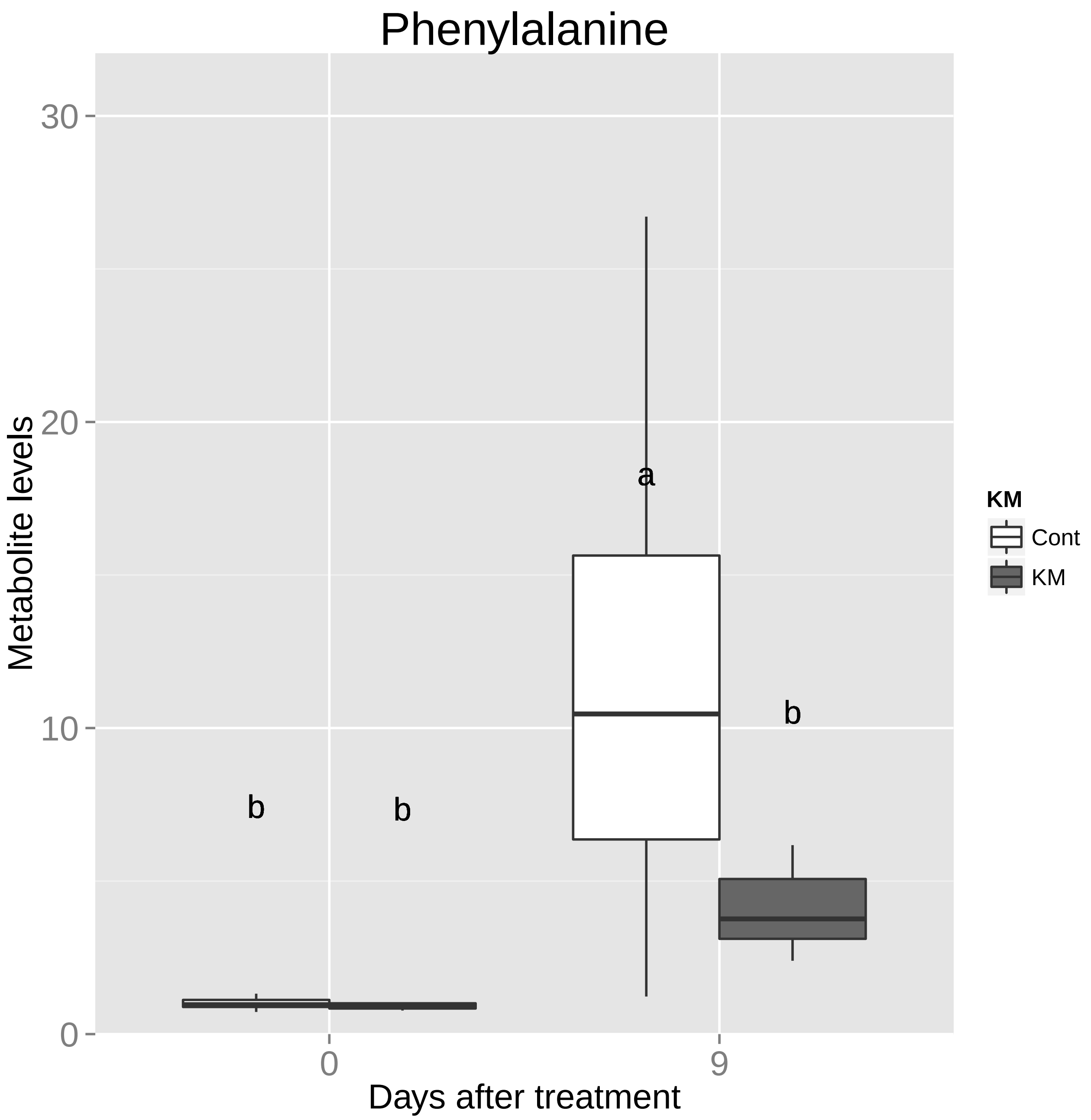

Phenylalanine
Metabolite levels
30
20
10
0
a
b
b
b
0
9
Days after treatment
KM
Cont
KM

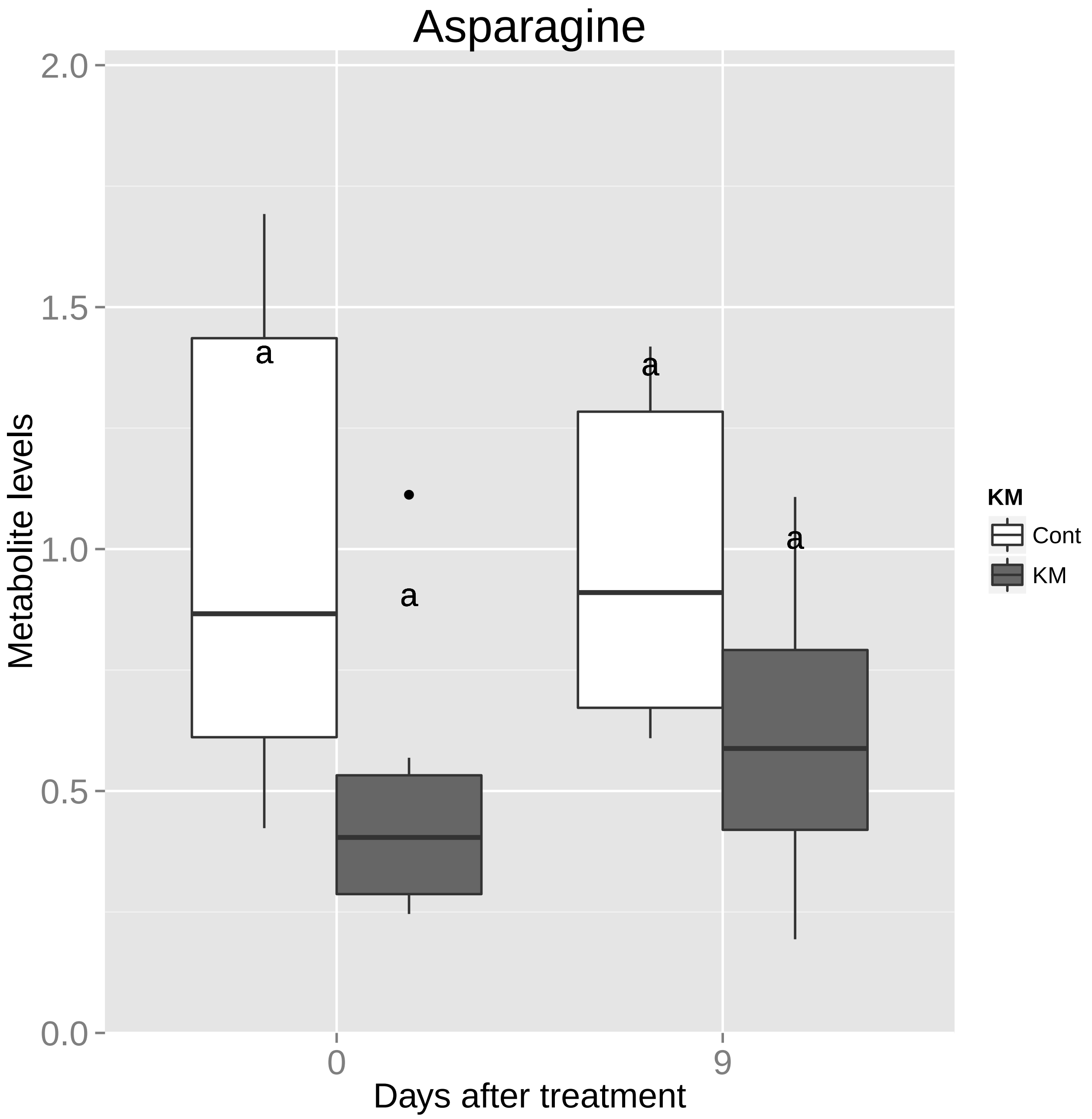
Asparagine
Metabolite levels
2.0
1.5
1.0
0.5
0.0
a
a
a
a
0
9
Days after treatment
KM
Cont
KM

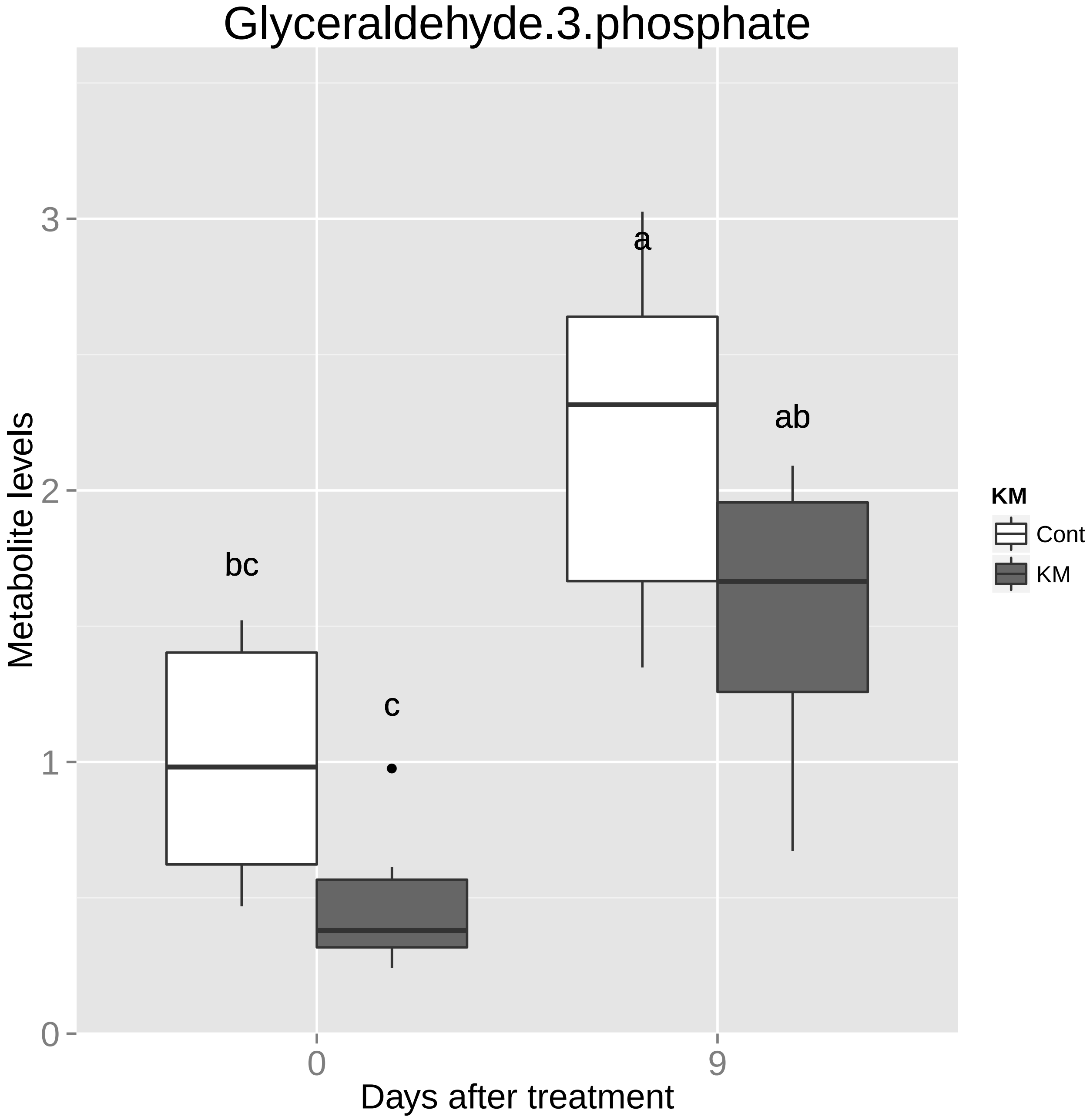
Glyceraldehyde.3.phosphate
Metabolite levels
3
2
1
0
0
9
Days after treatment
a
ab
bc
c
KM
Cont
KM

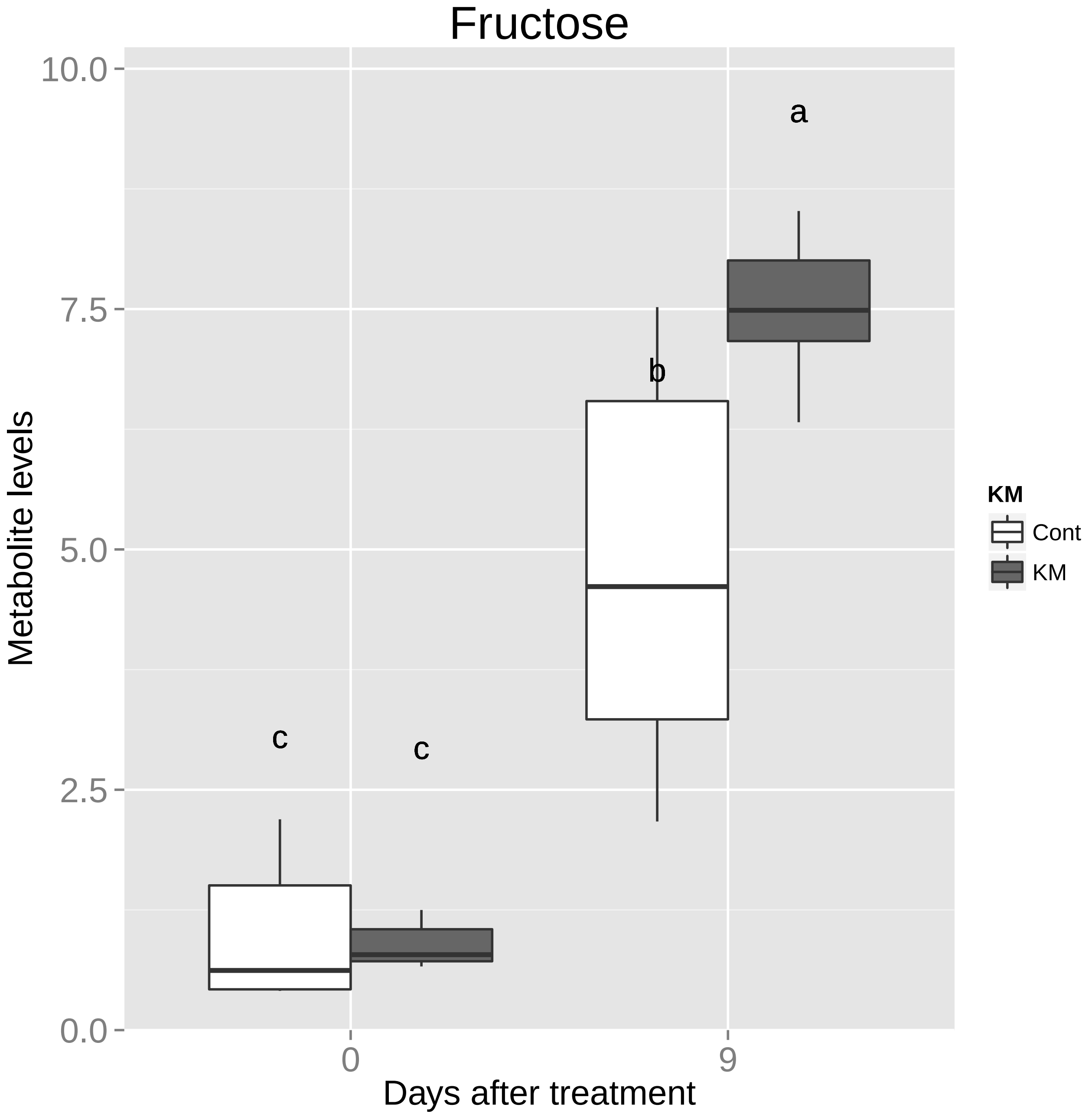
Fructose
Metabolite levels
10.0
7.5
5.0
2.5
0.0
a
b
c
c
0
9
Days after treatment
KM
Cont
KM

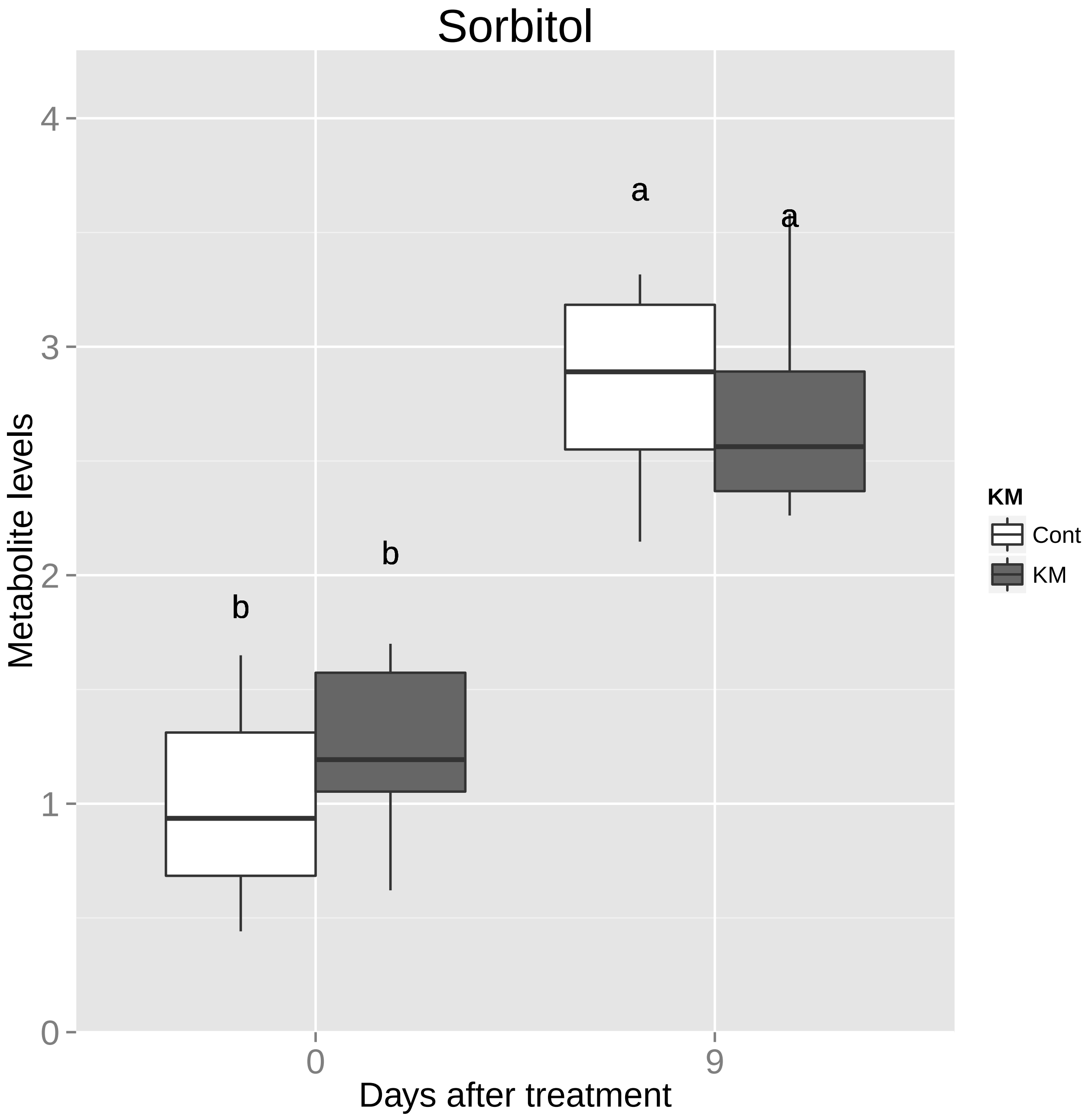

Sorbitol
Metabolite levels
Days after treatment
0
1
2
3
4
0
9
a
a
b
b
KM
Cont
KM

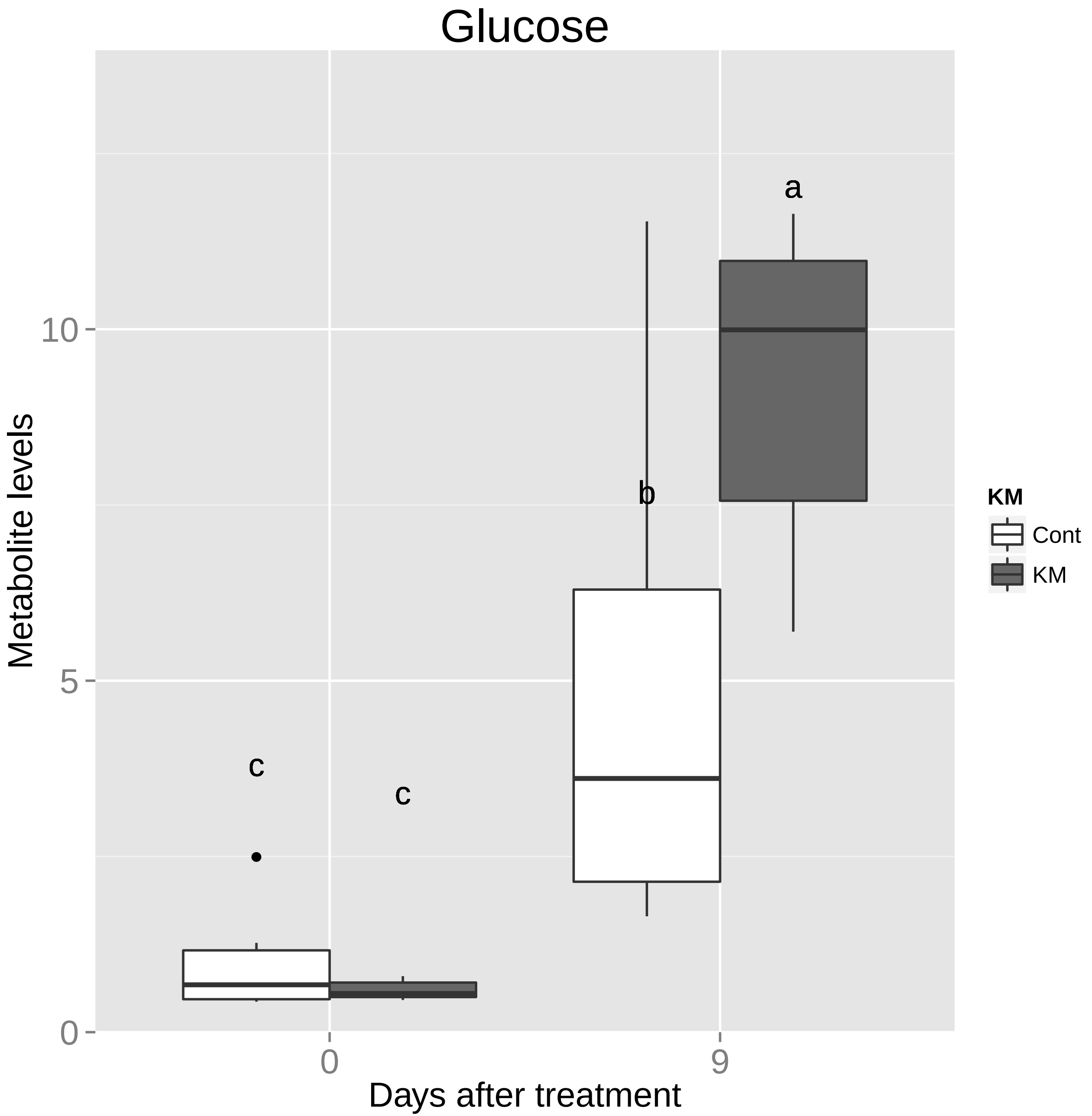
Glucose
Metabolite levels
Days after treatment
0
5
10
0
9
a
b
c
c
KM
Cont
KM

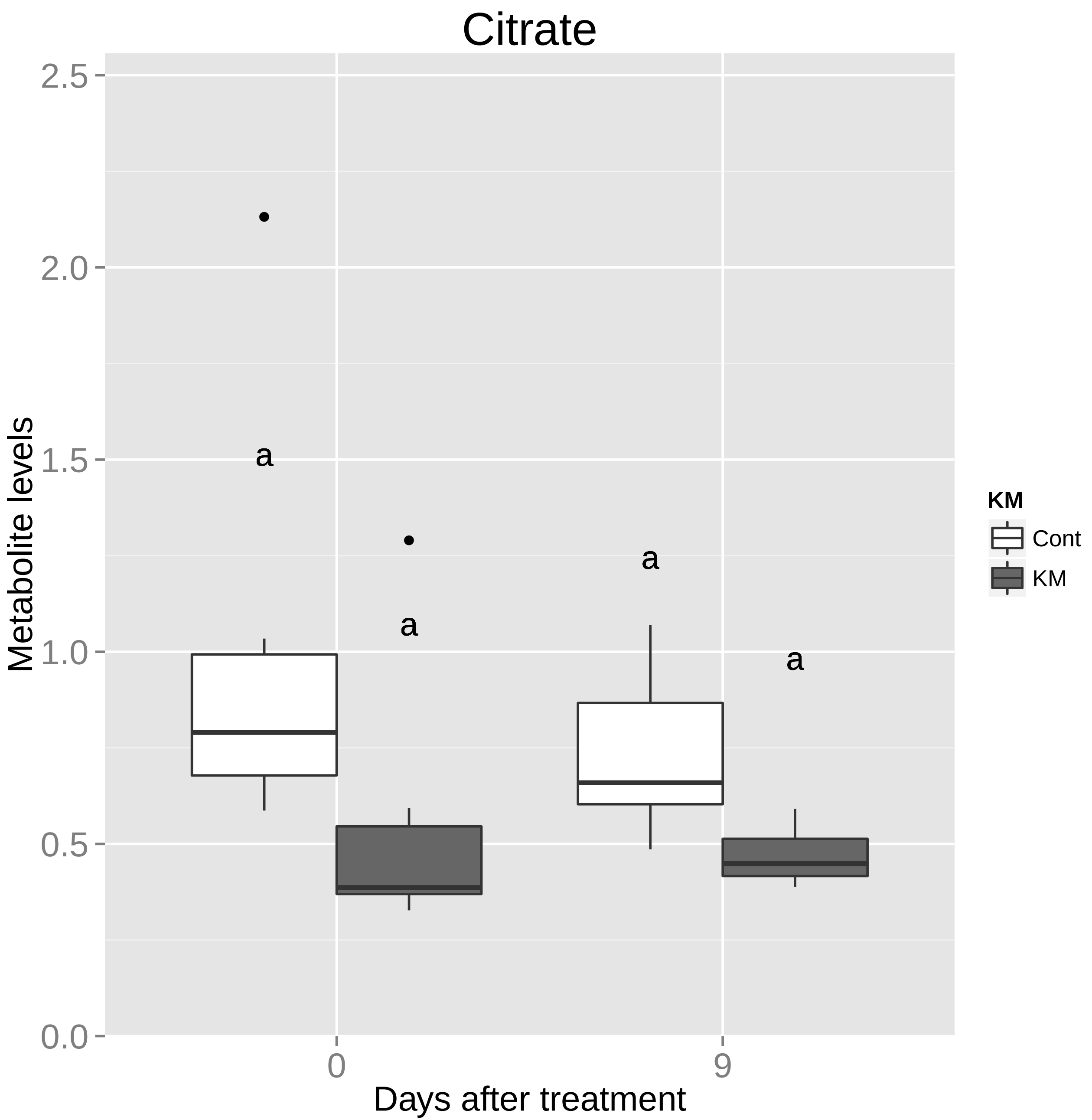
Citrate
Metabolite levels
2.5
2.0
1.5
1.0
0.5
0.0
a
a
a
a
0
9
Days after treatment
KM
Cont
KM

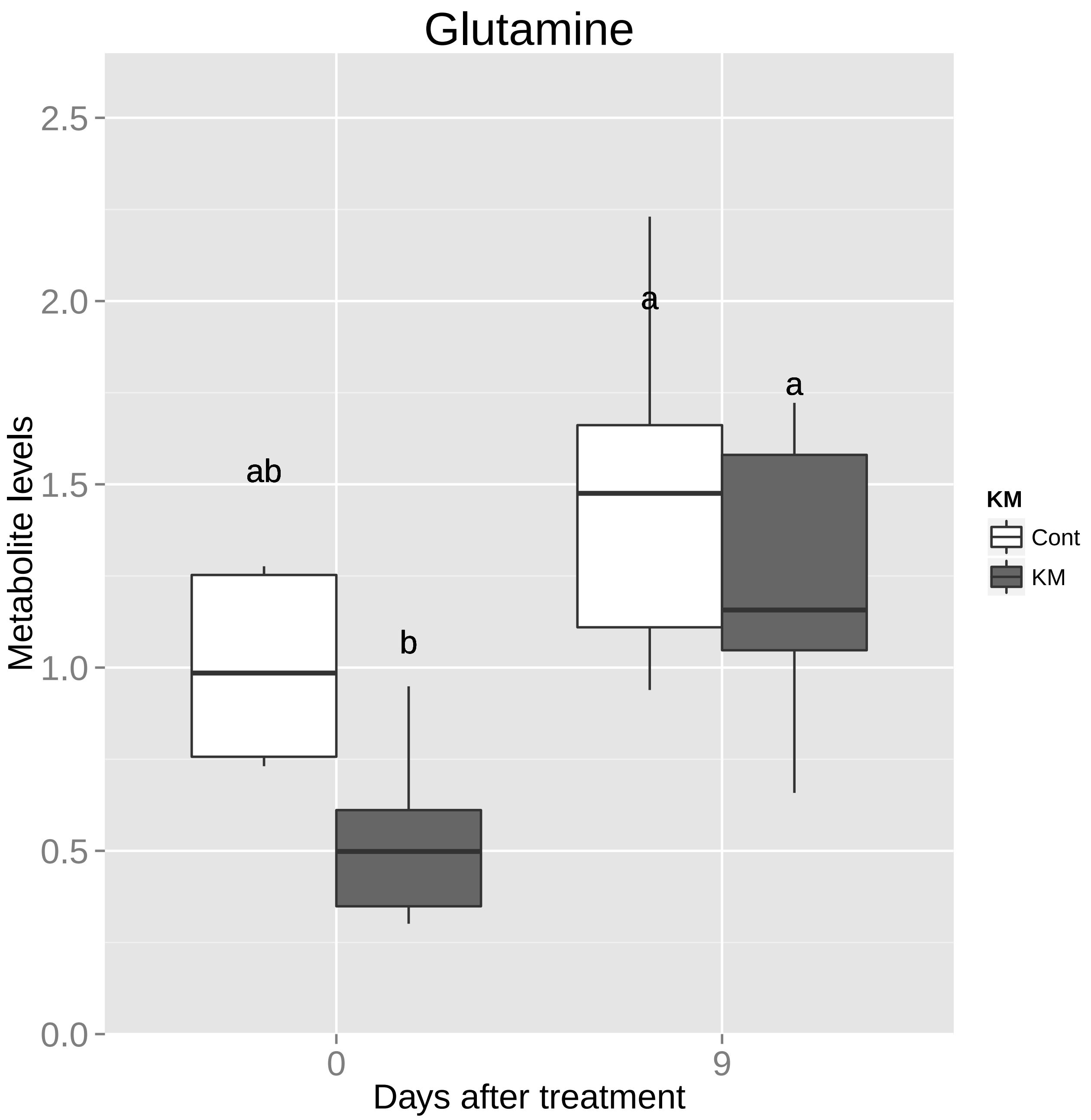

Glutamine
Metabolite levels
2.5
2.0
1.5
1.0
0.5
0.0
ab
b
a
a
0
9
Days after treatment
KM
Cont
KM

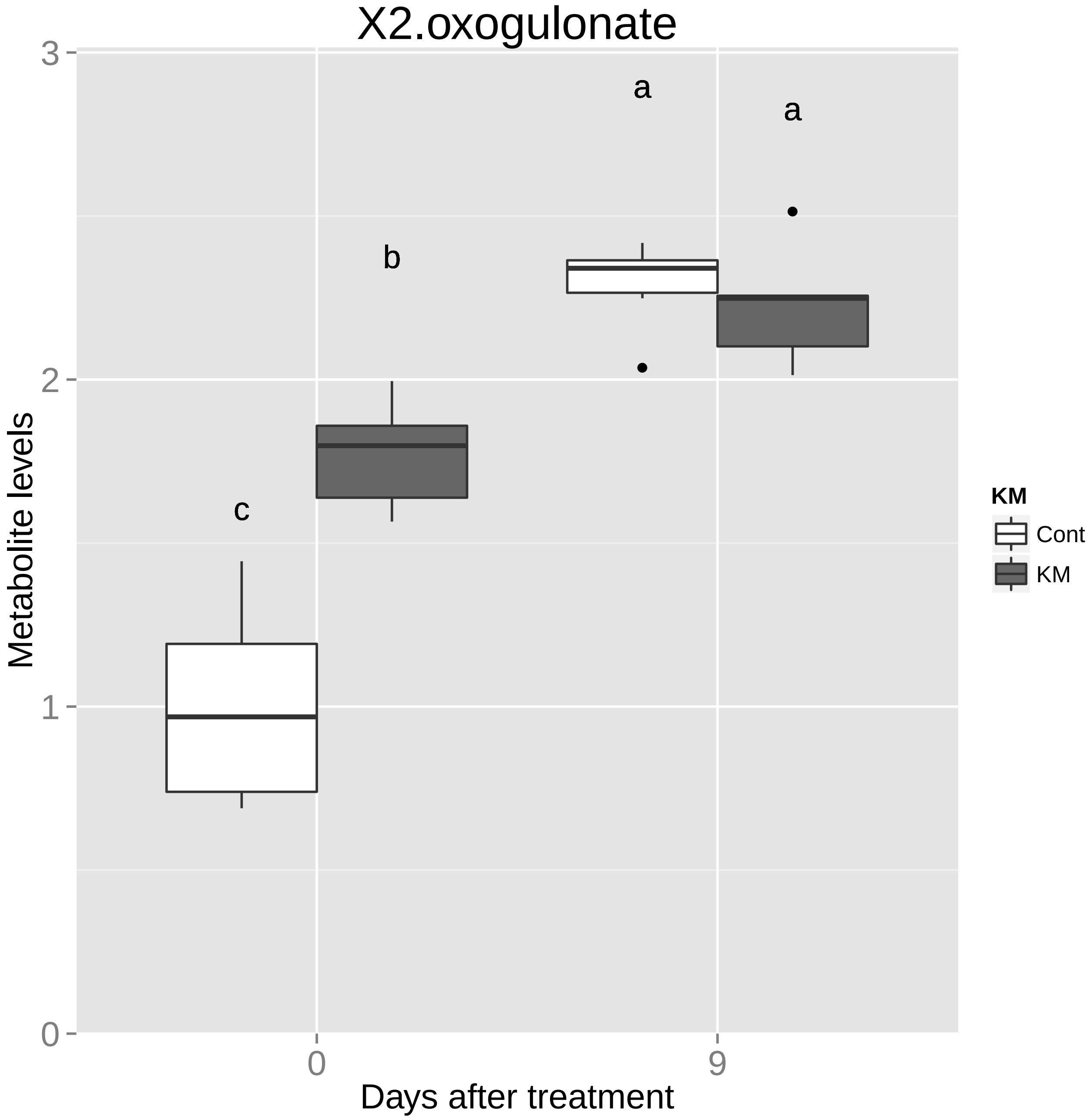
X2.oxogulonate
Metabolite levels
Days after treatment
0
1
2
3
0
9
a
a
b
c
KM
Cont
KM

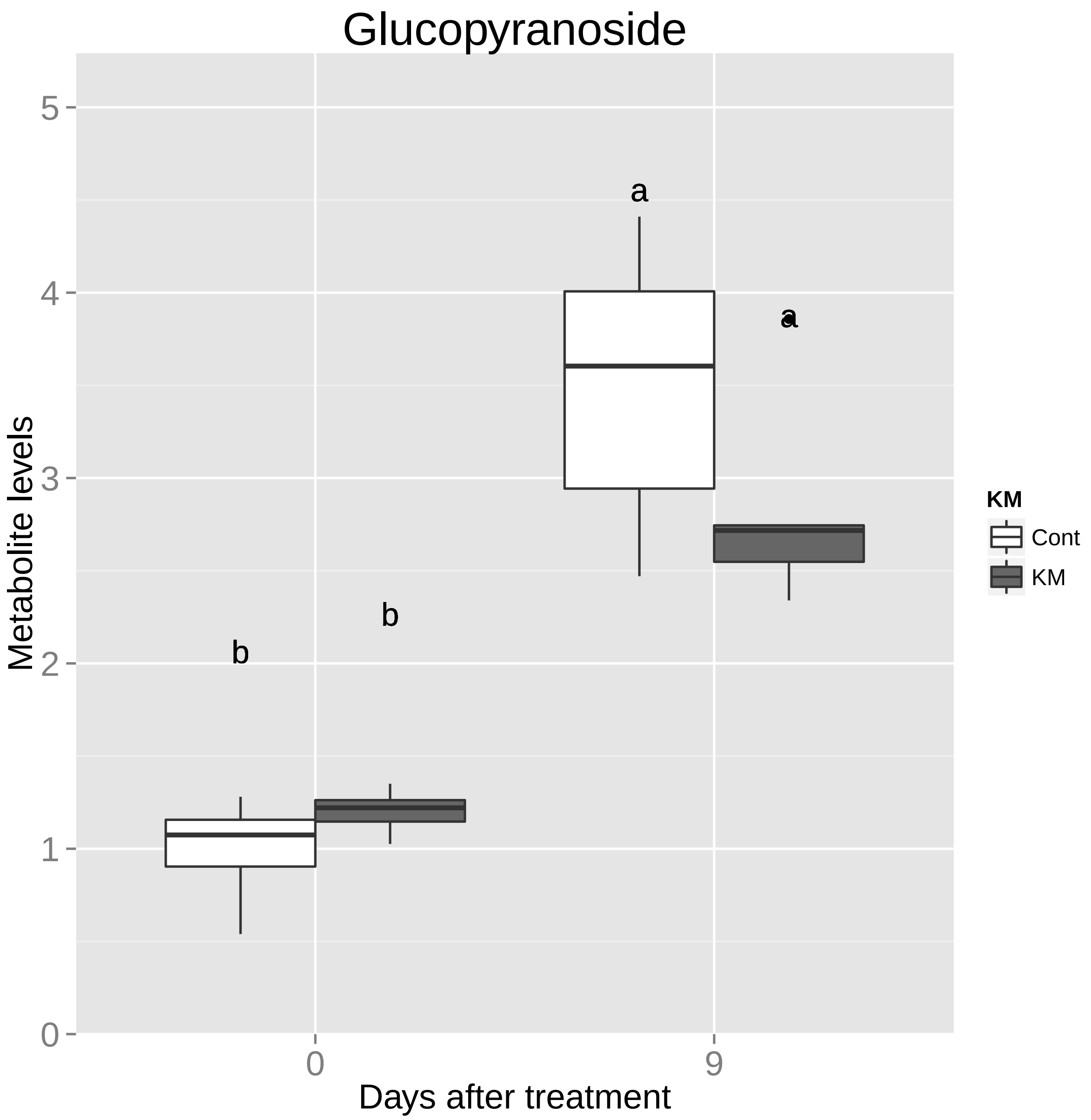
Glucopyranoside
Metabolite levels
0
1
2
3
4
5
b
b
a
a
0
9
Days after treatment
KM
Cont
KM

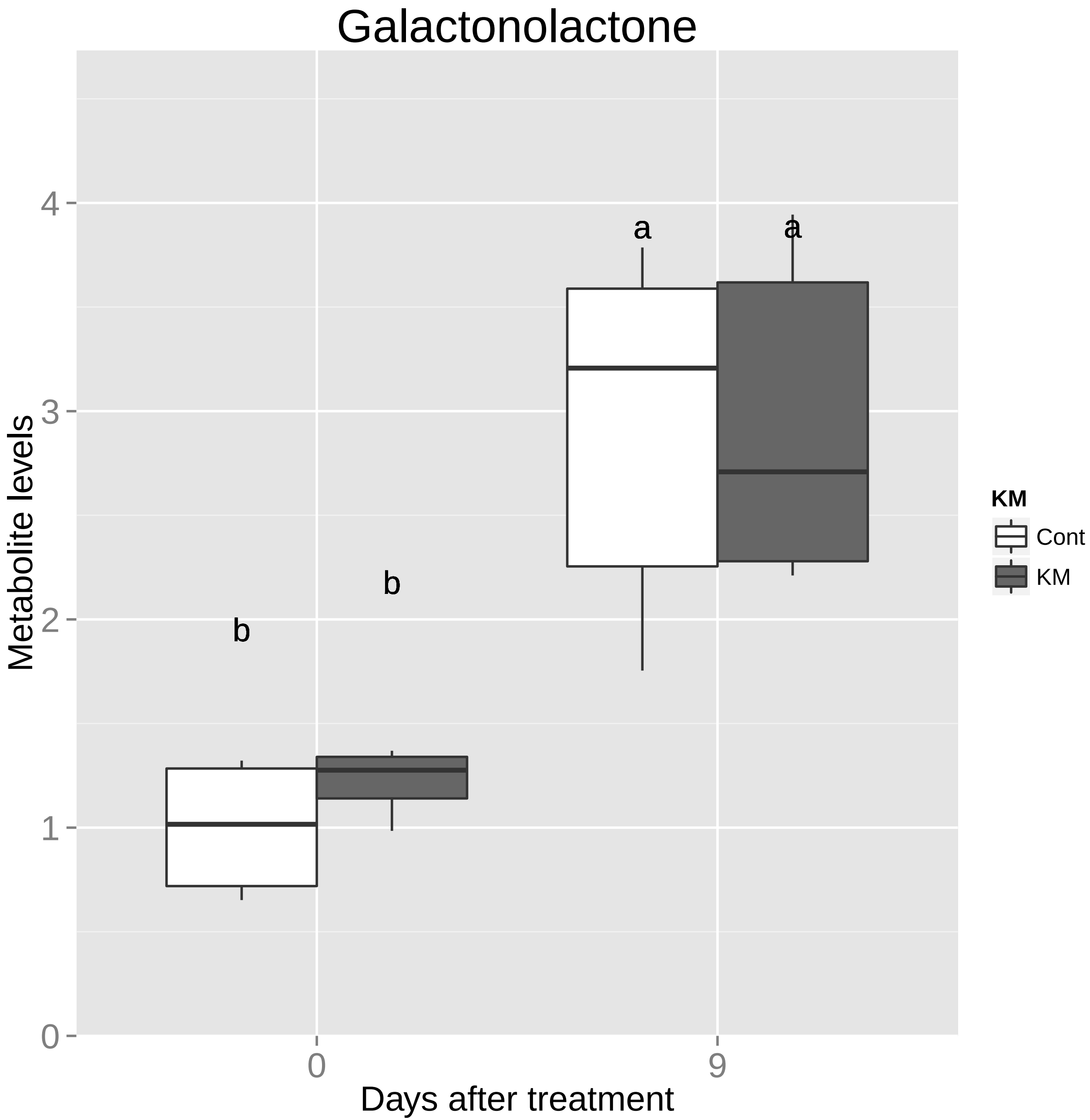
Galactonolactone
Metabolite levels
Days after treatment
0
1
2
3
4
0
9
a
a
b
b
KM
Cont
KM

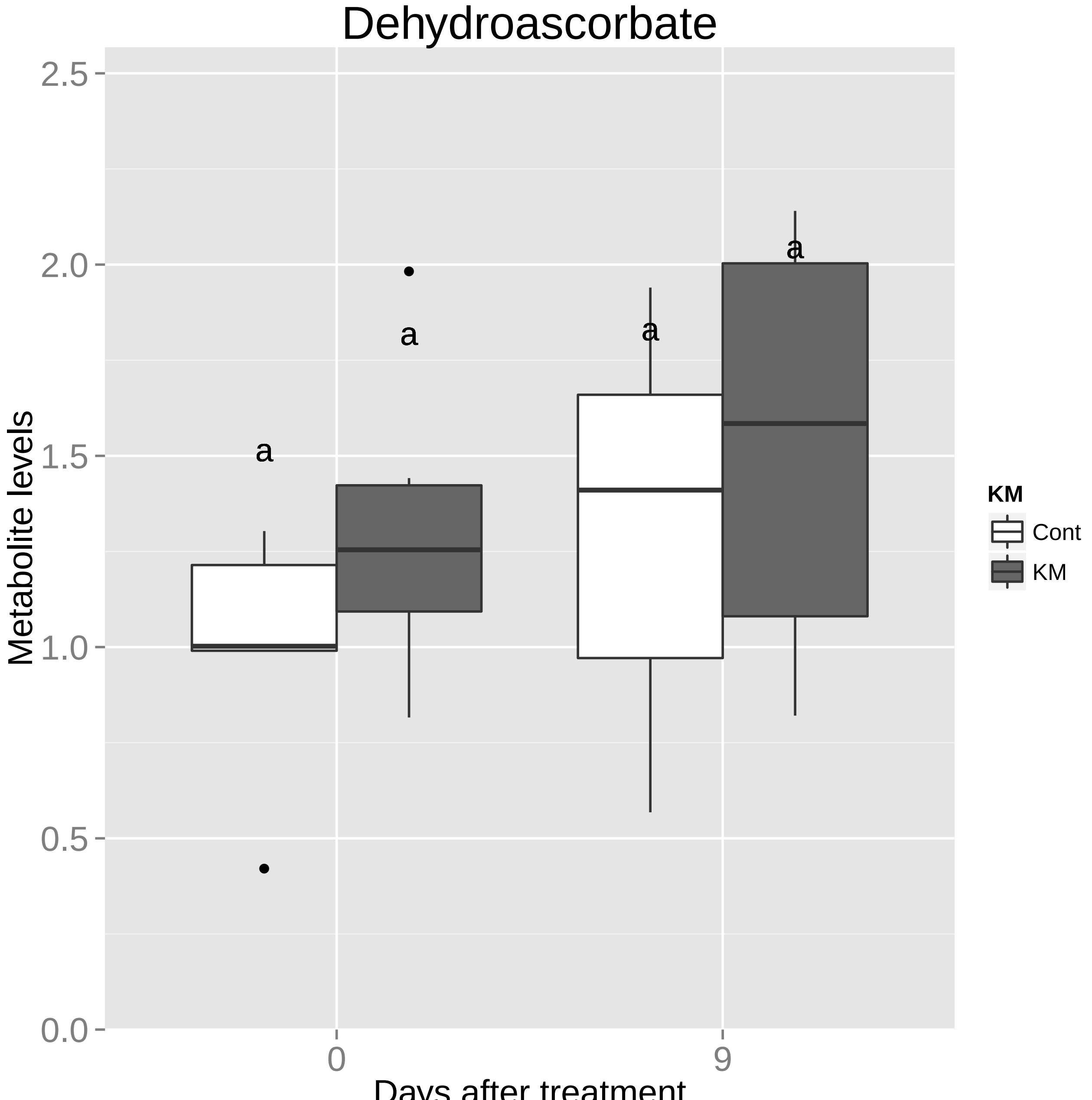
Dehydroascorbate
Metabolite levels
2.5
2.0
1.5
1.0
0.5
0.0
a
a
a
a
0
9
Days after treatment
KM
Cont
KM

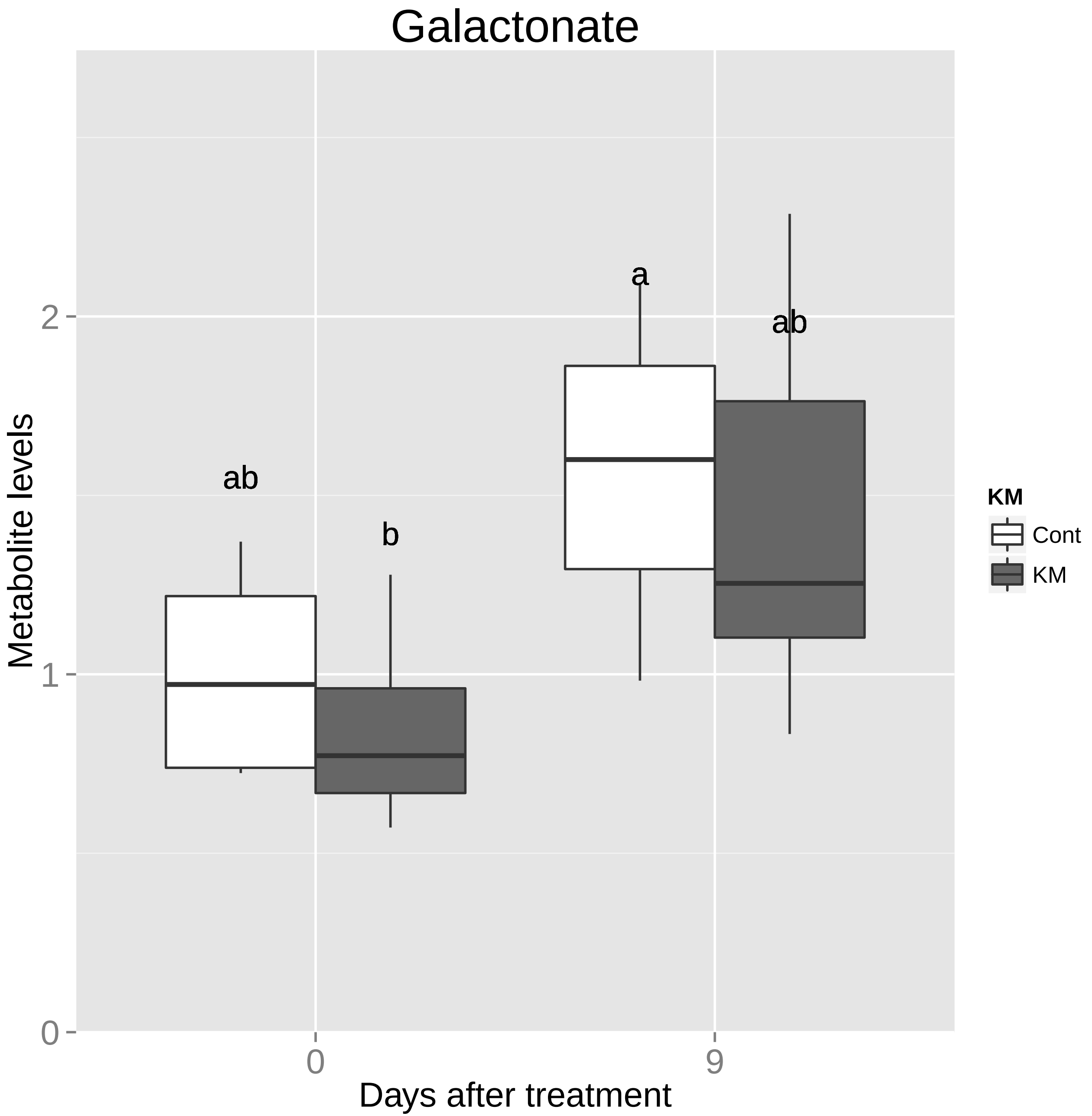
Galactonate
Metabolite levels
Days after treatment
0
1
2
0
9
ab
b
a
ab
KM
Cont
KM

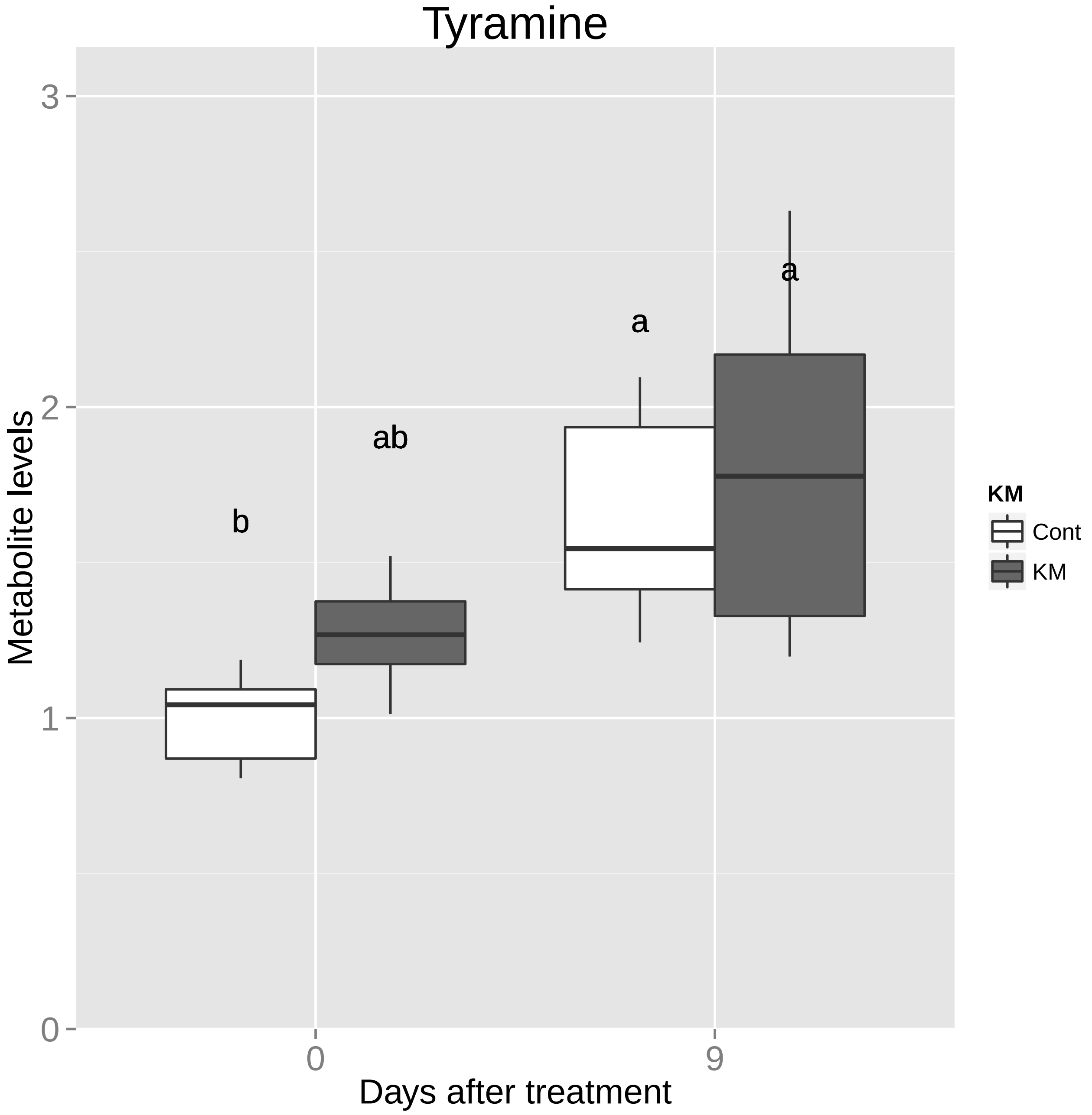
Tyramine
Metabolite levels
Days after treatment
0
1
2
3
0
9
b
ab
a
a
KM
Cont
KM

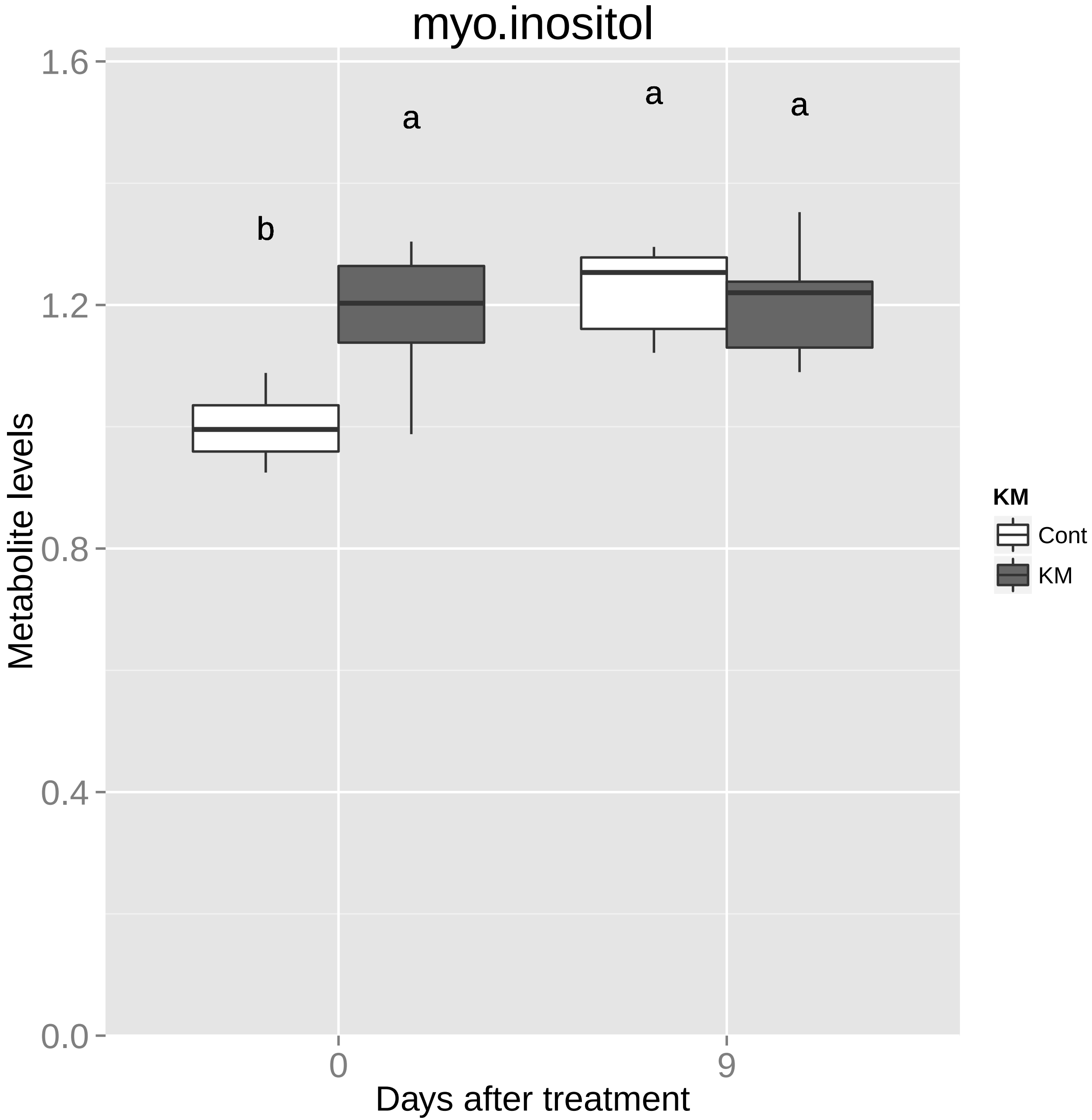
myo.inositol
1.6
1.2
0.8
0.4
0.0
Metabolite levels
b
a
a
a
0
9
Days after treatment
KM
Cont
KM

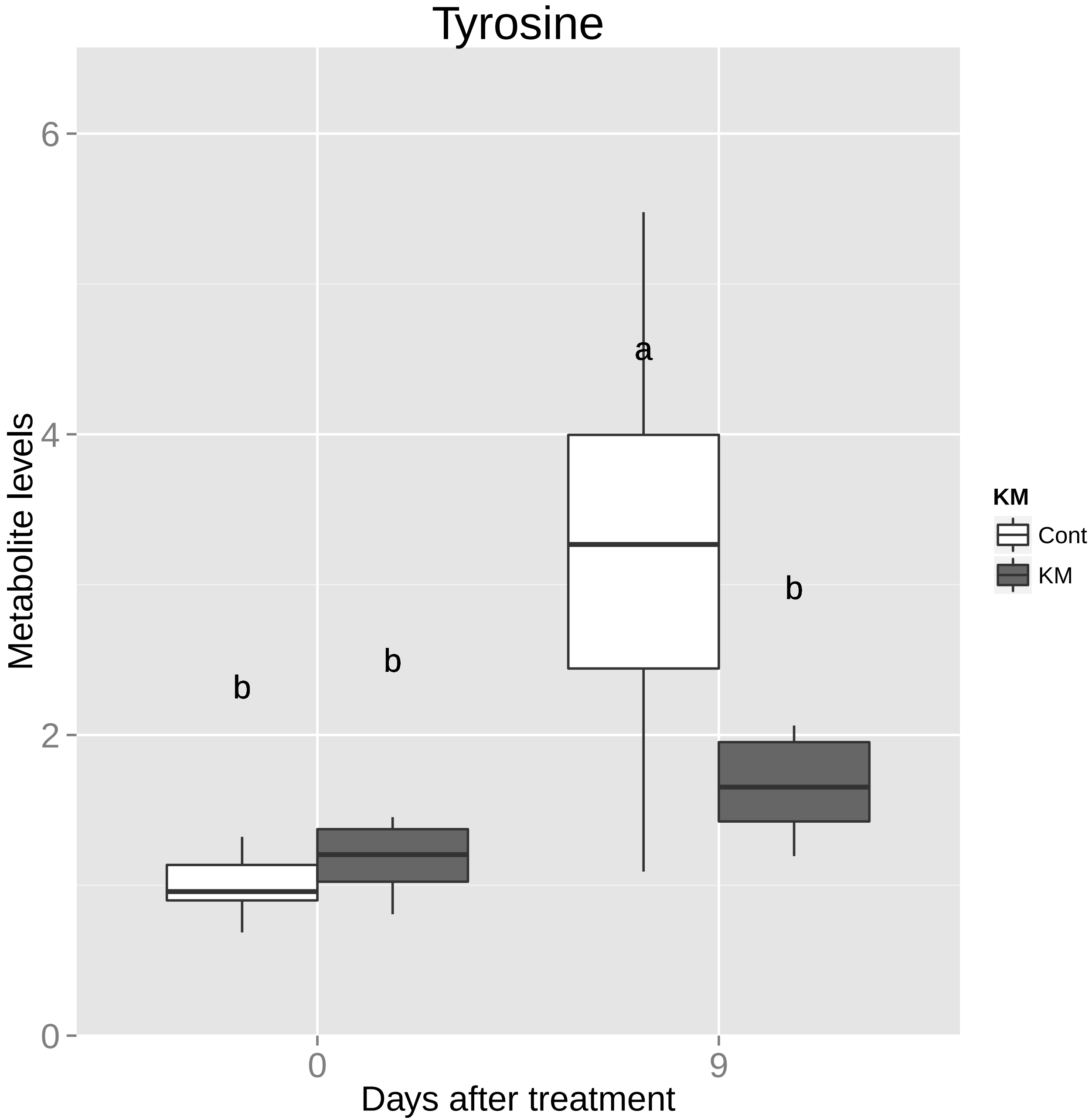

Tyrosine
Metabolite levels
0
2
4
6
a
b
b
b
b
0
9
Days after treatment
KM
Cont
KM

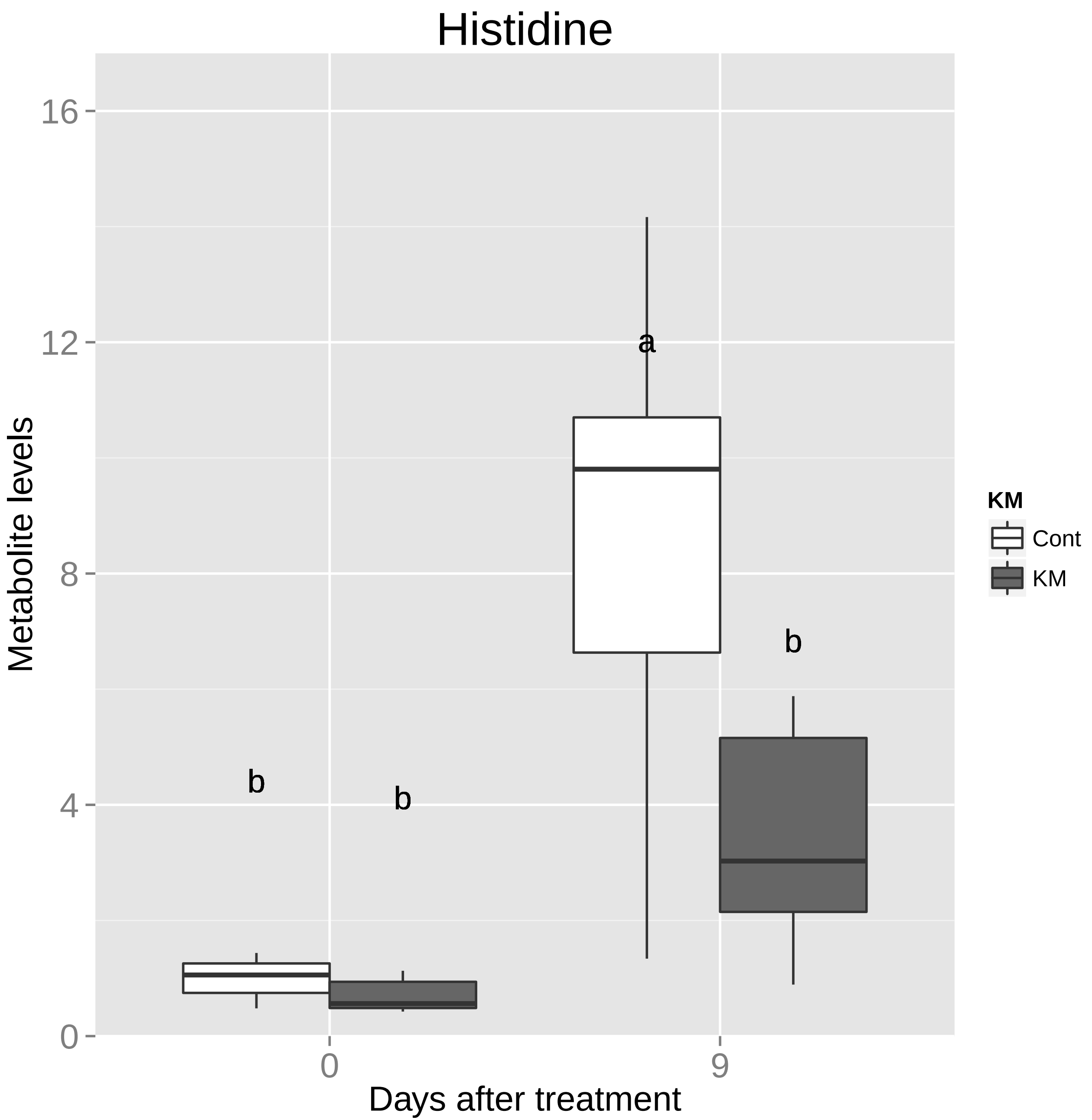

Histidine
16
12
8
4
0
a
b
b
b
0
9
Days after treatment
Metabolite levels
KM
Cont
KM

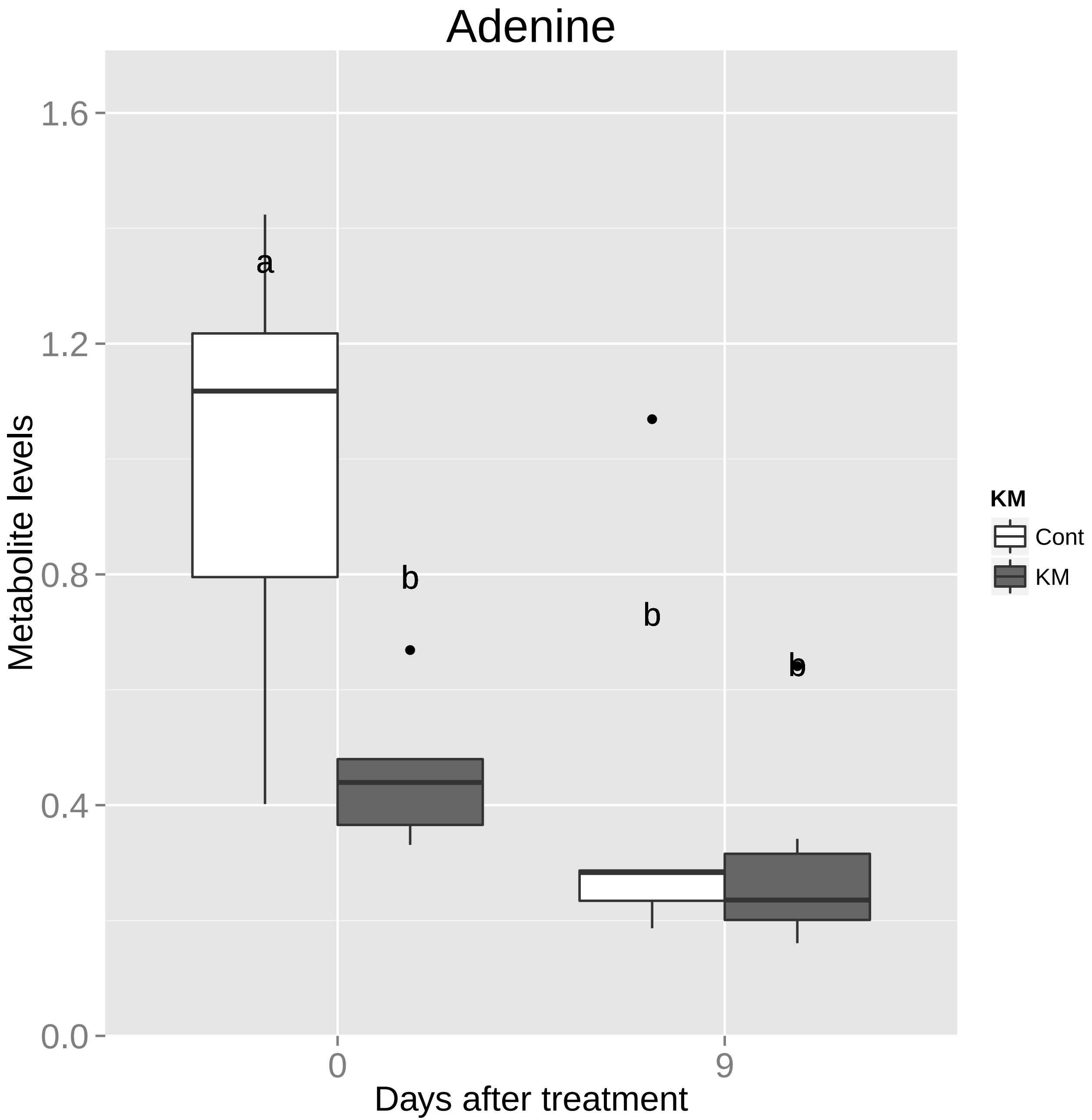
Adenine
Metabolite levels
Days after treatment
0.0
0.4
0.8
1.2
1.6
0
9
a
b
b
b
KM
Cont
KM

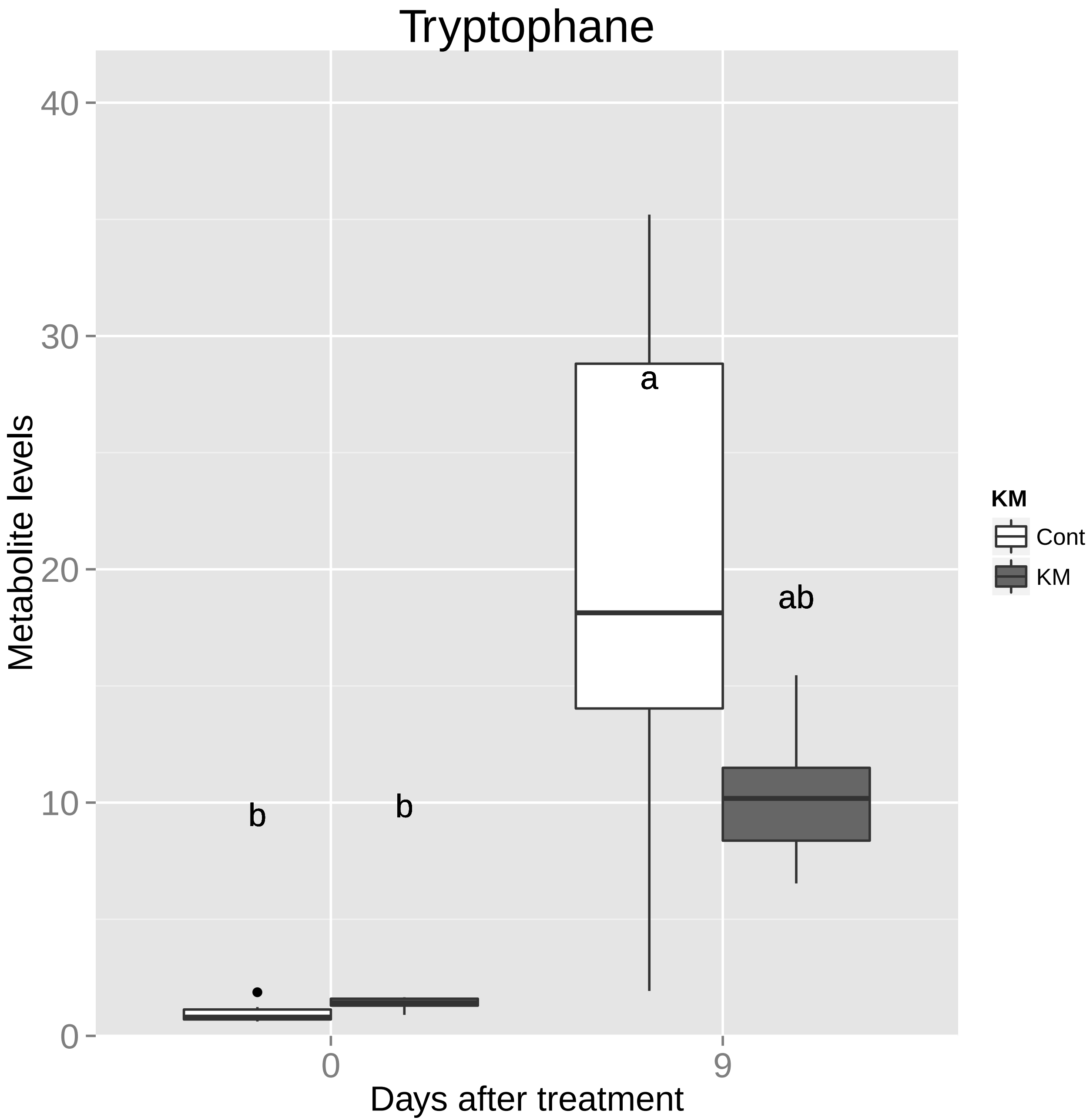
Tryptophane
Metabolite levels
40
30
20
10
0
a
ab
b
b
0
9
Days after treatment
KM
Cont
KM

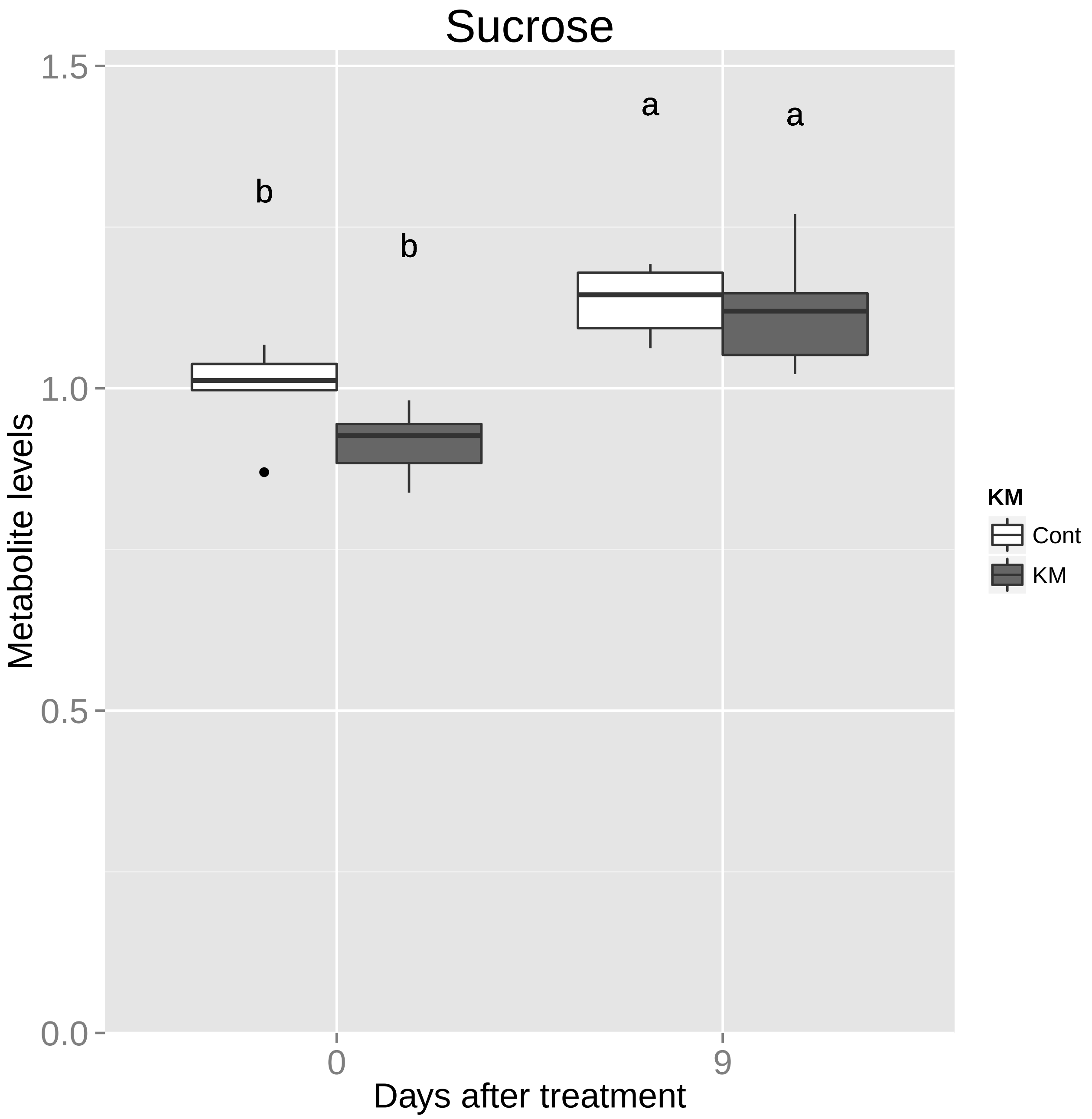

Sucrose
1.5
1.0
0.5
0.0
Metabolite levels
b
b
a
a
0
9
Days after treatment
KM
Cont
KM

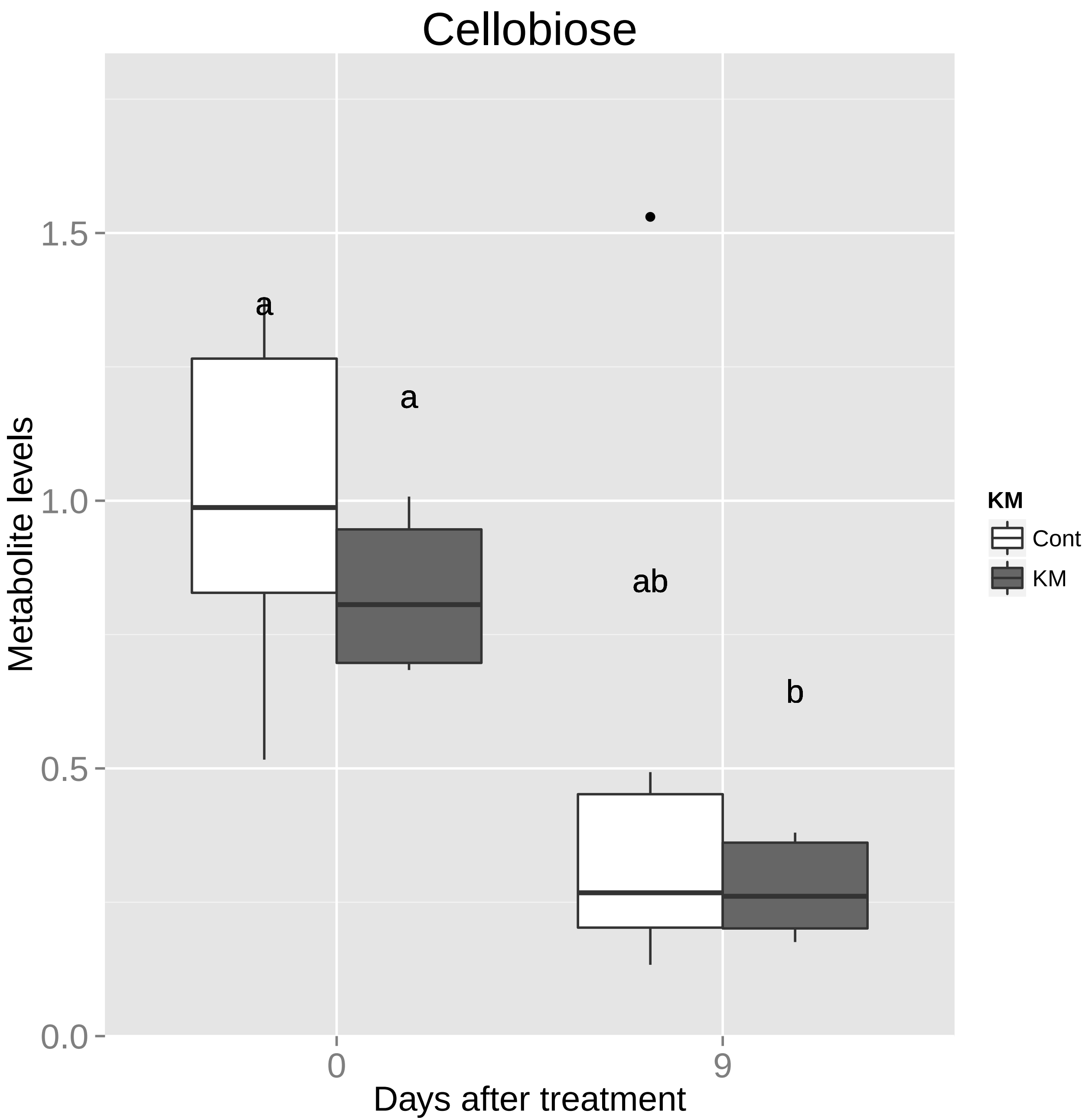
Cellobiose
Metabolite levels
0.0
0.5
1.0
1.5
a
a
ab
b
0
9
Days after treatment
KM
Cont
KM

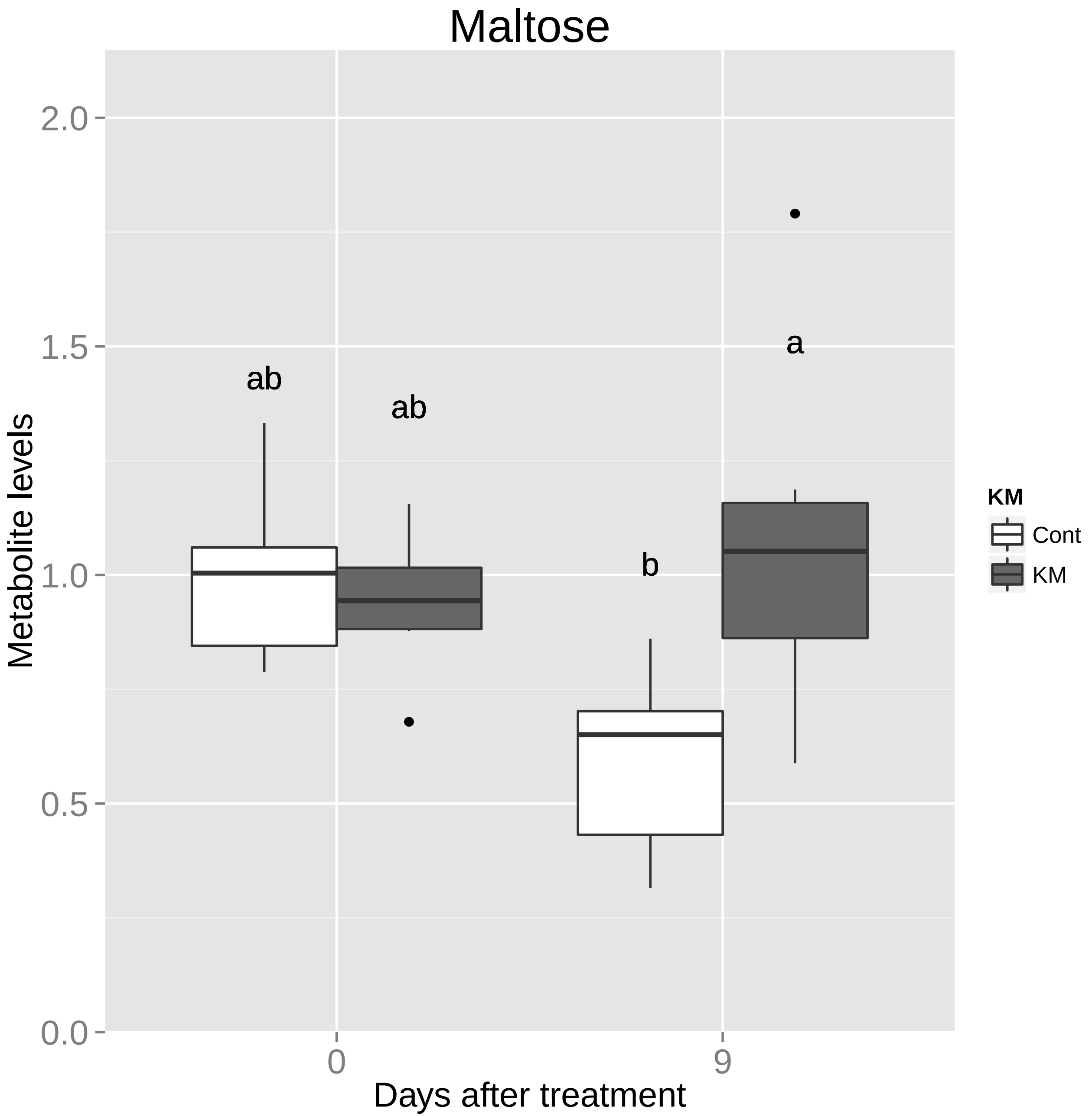

Maltose
Metabolite levels
0.0
0.5
1.0
1.5
2.0
ab
ab
b
a
0
9
Days after treatment
KM
Cont
KM

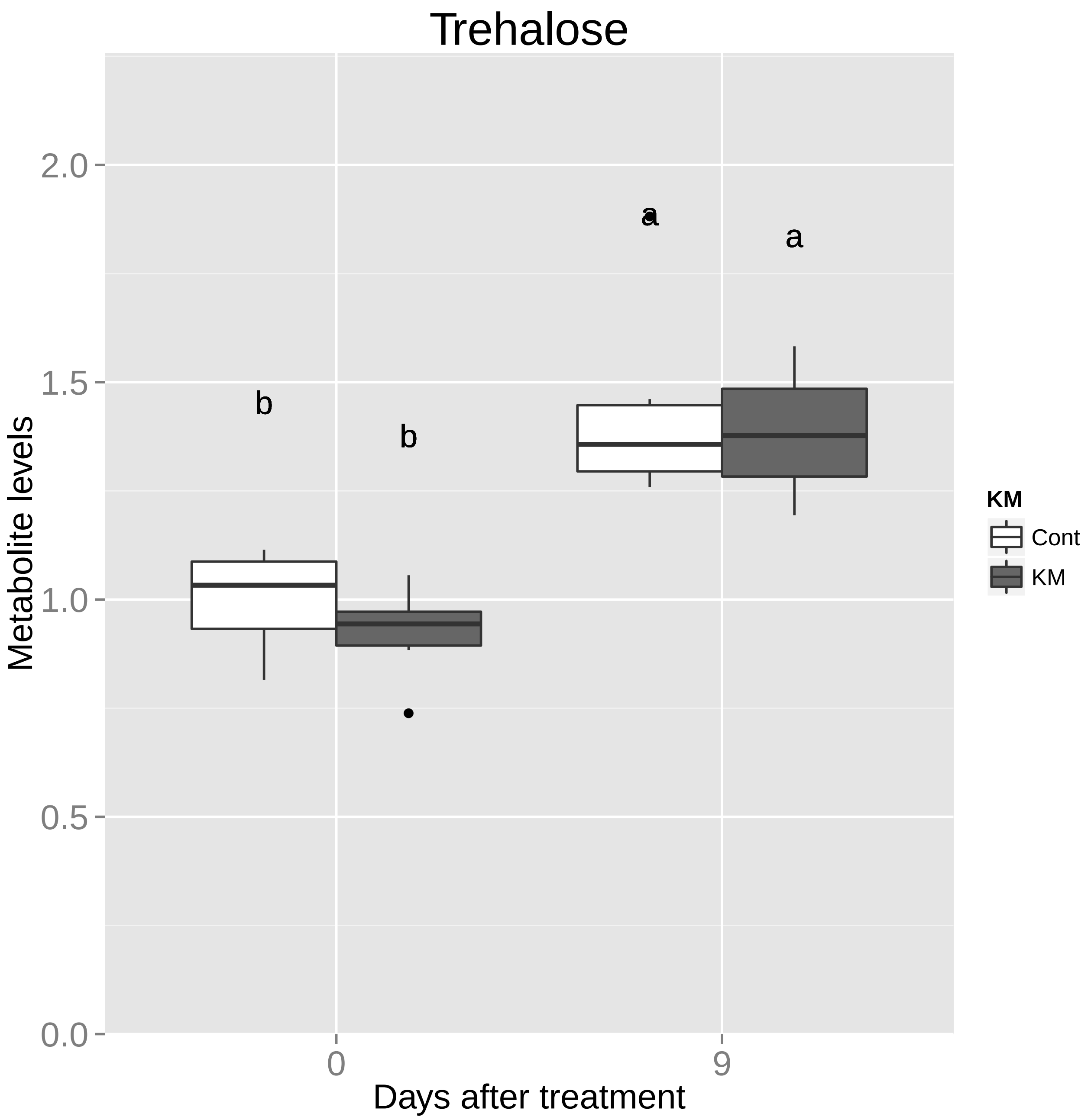
Trehalose
2.0
1.5
1.0
0.5
0.0
a
a
b
b
KM
Cont
KM
0
9
Metabolite levels
Days after treatment

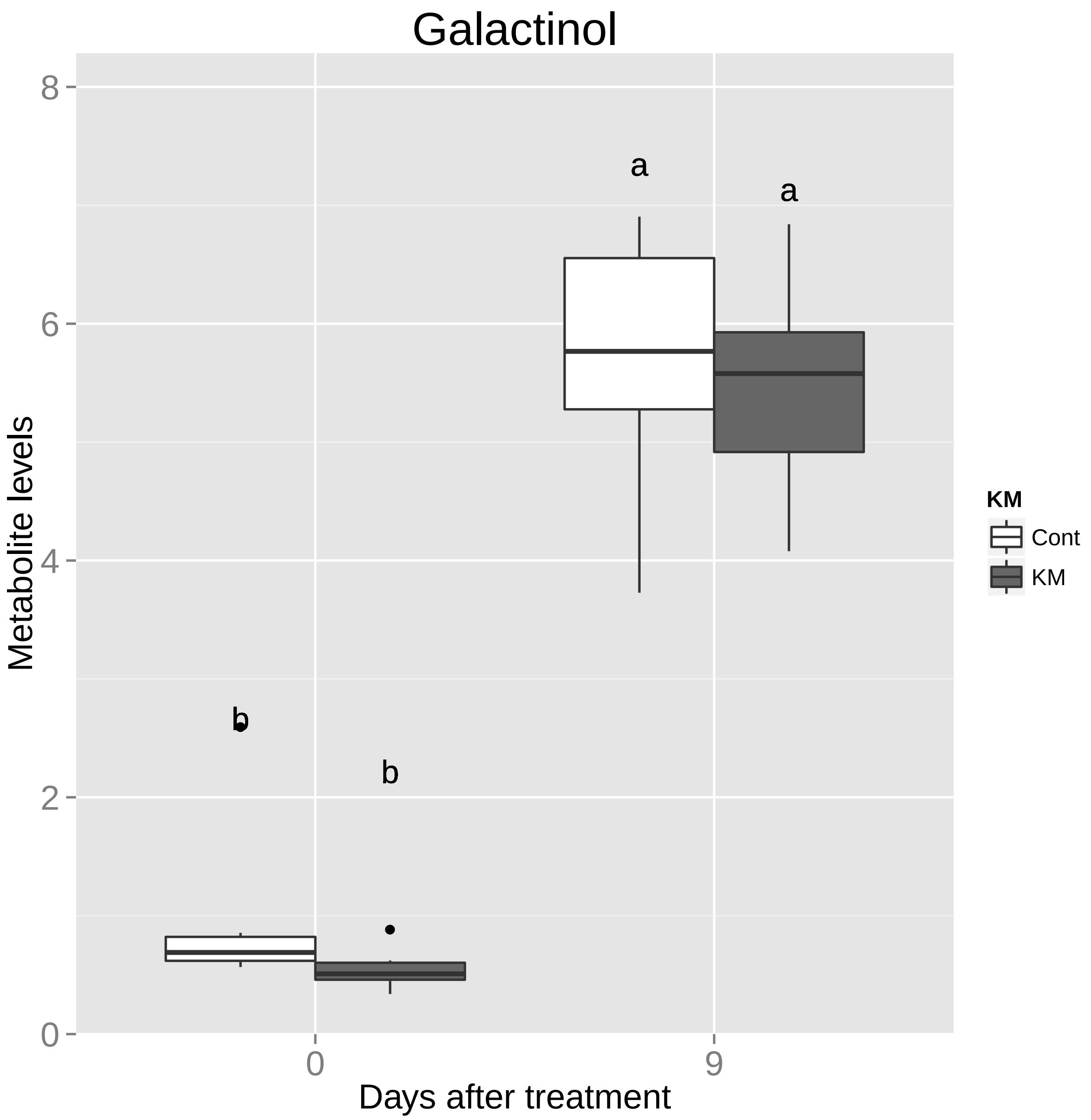

Galactinol
Metabolite levels
8
6
4
2
0
a
a
b
b
0
9
Days after treatment
KM
Cont
KM

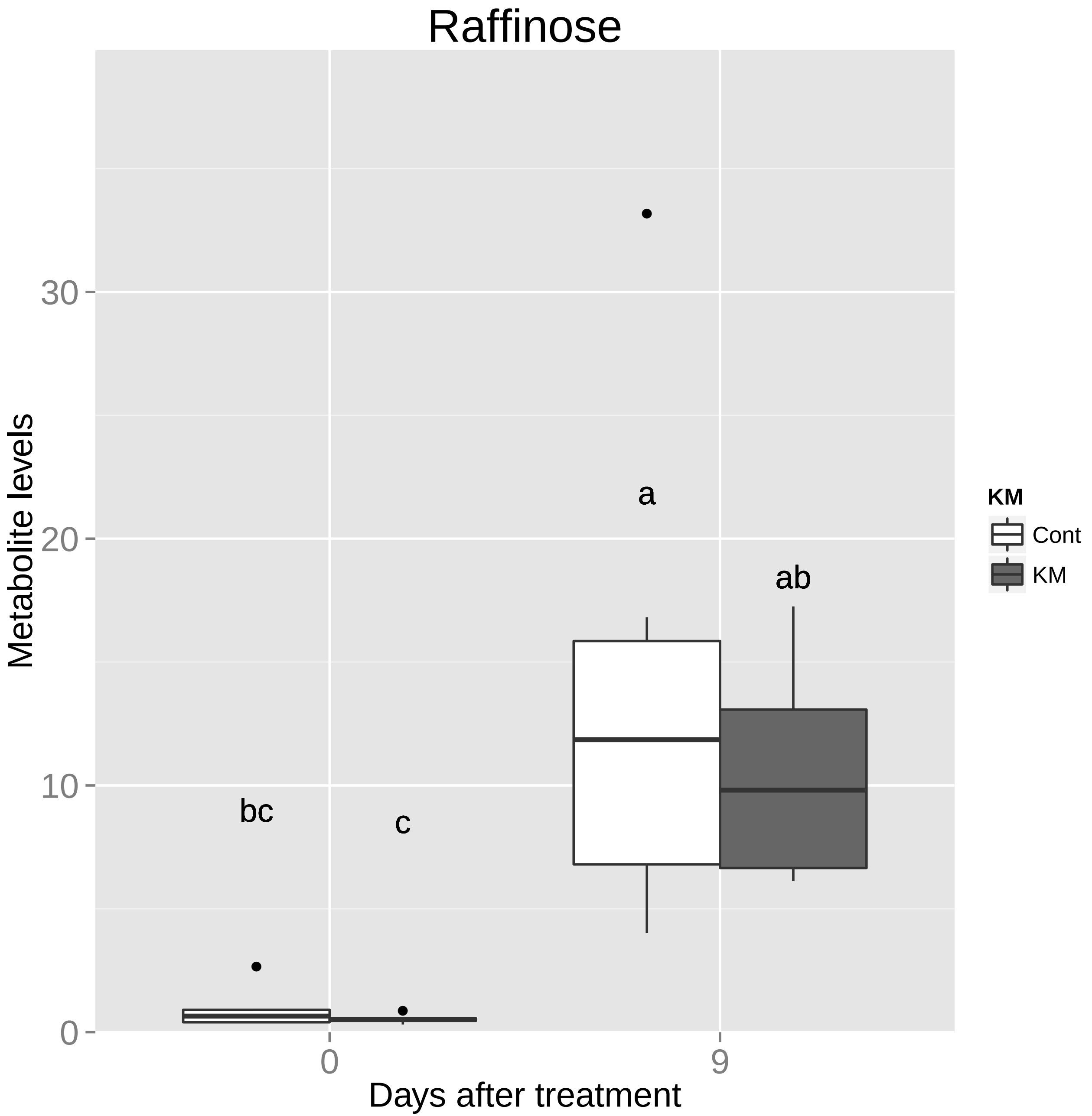
Raffinose
Metabolite levels
30
20
10
0
a
ab
bc
c
0
9
Days after treatment
KM
Cont
KM

Supplementary Figure S4B

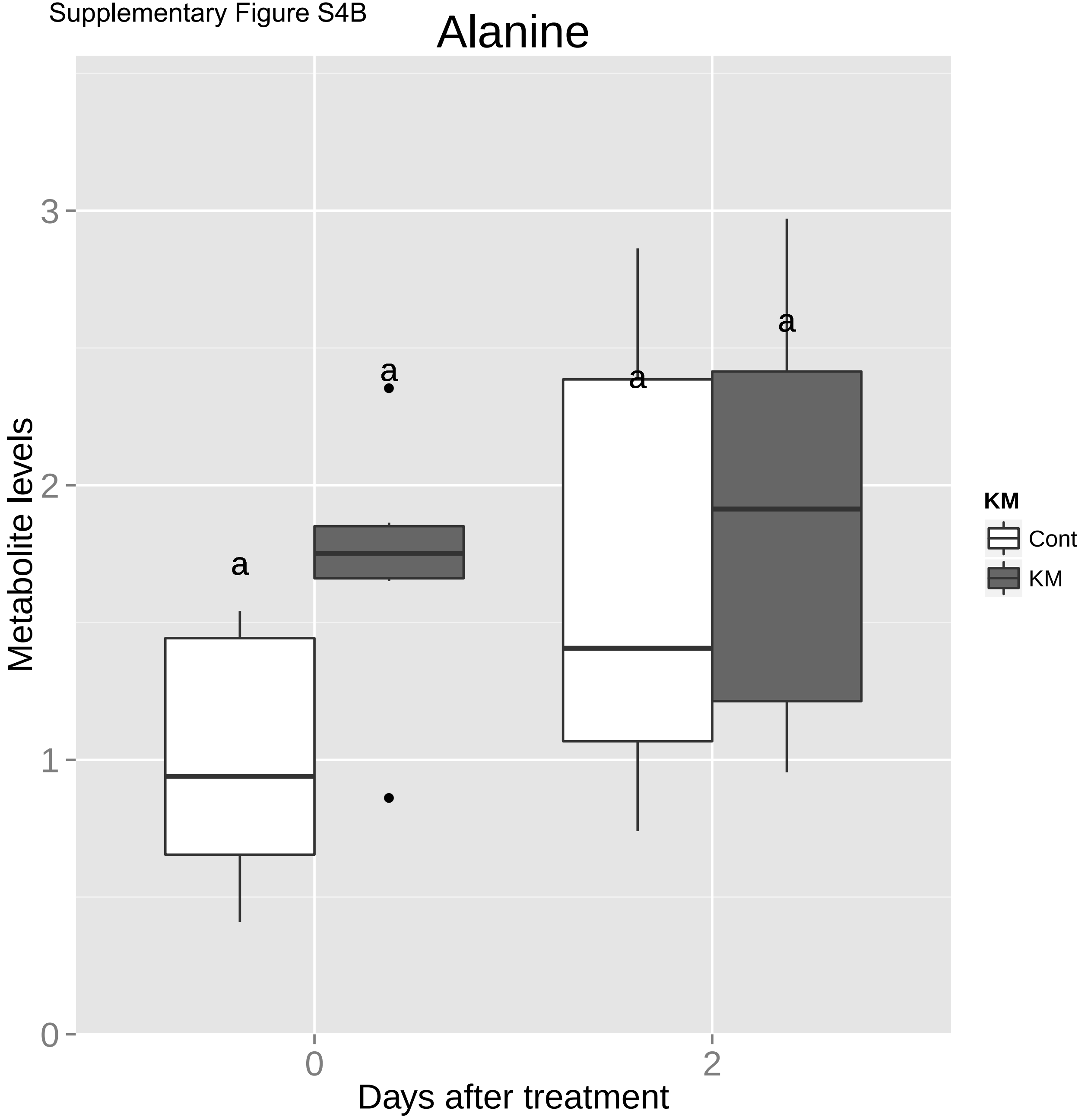

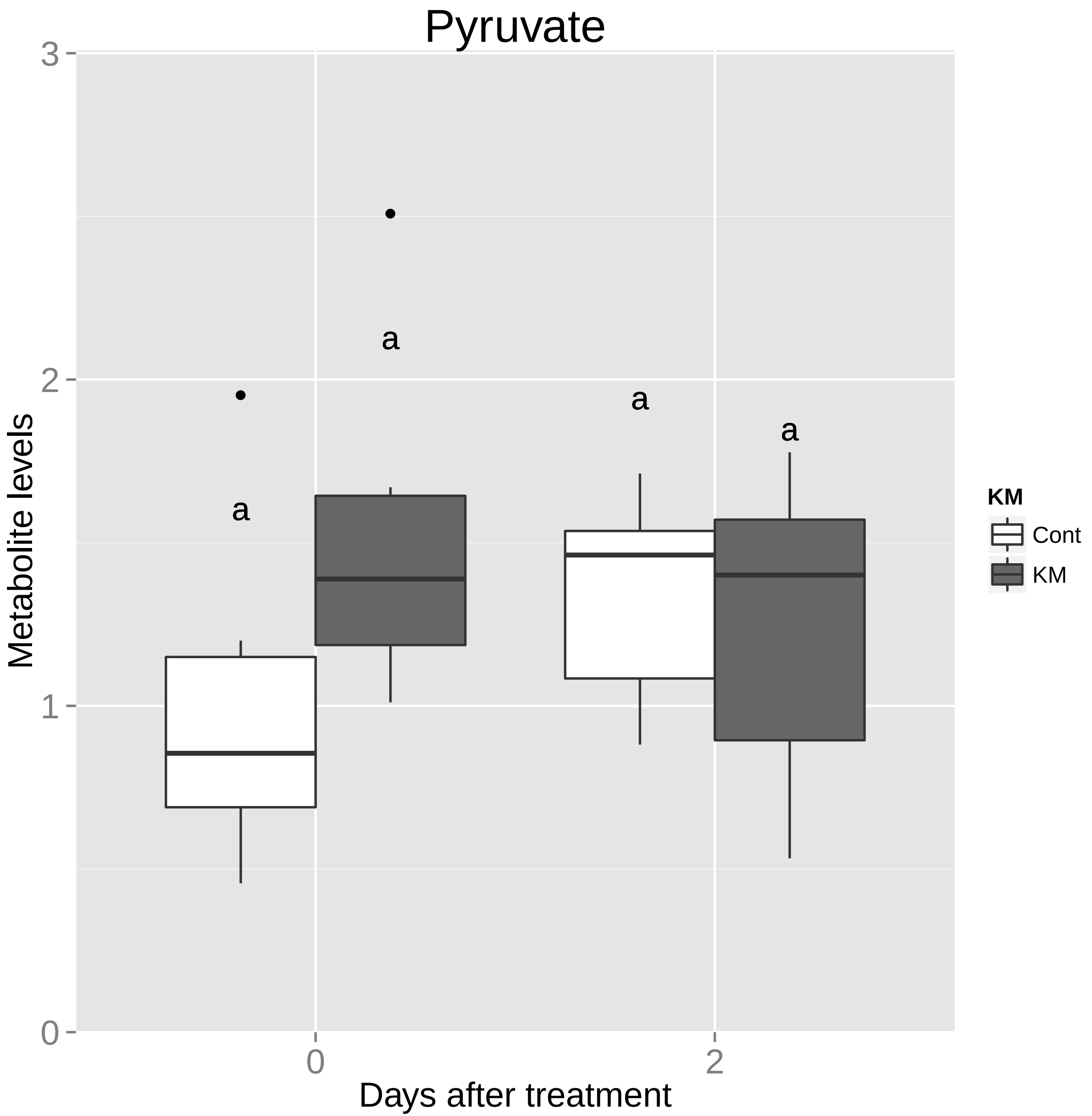

Pyruvate
3
2
1
0
a
a
a
a
Metabolite levels
0
2
Days after treatment
KM
Cont
KM

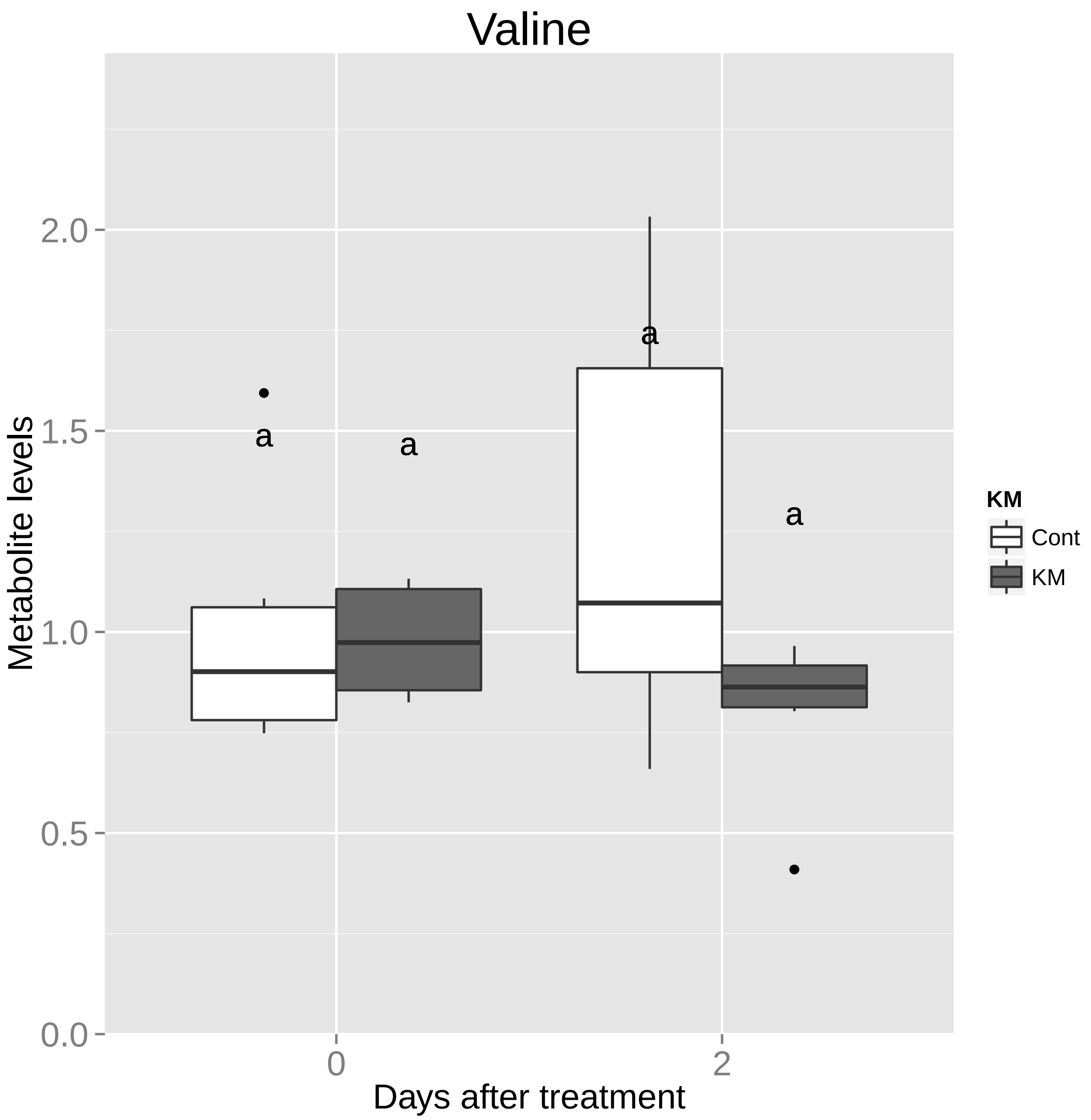

Valine
Metabolite levels
0.0
0.5
1.0
1.5
2.0
a
a
a
a
0
2
Days after treatment
KM
Cont
KM

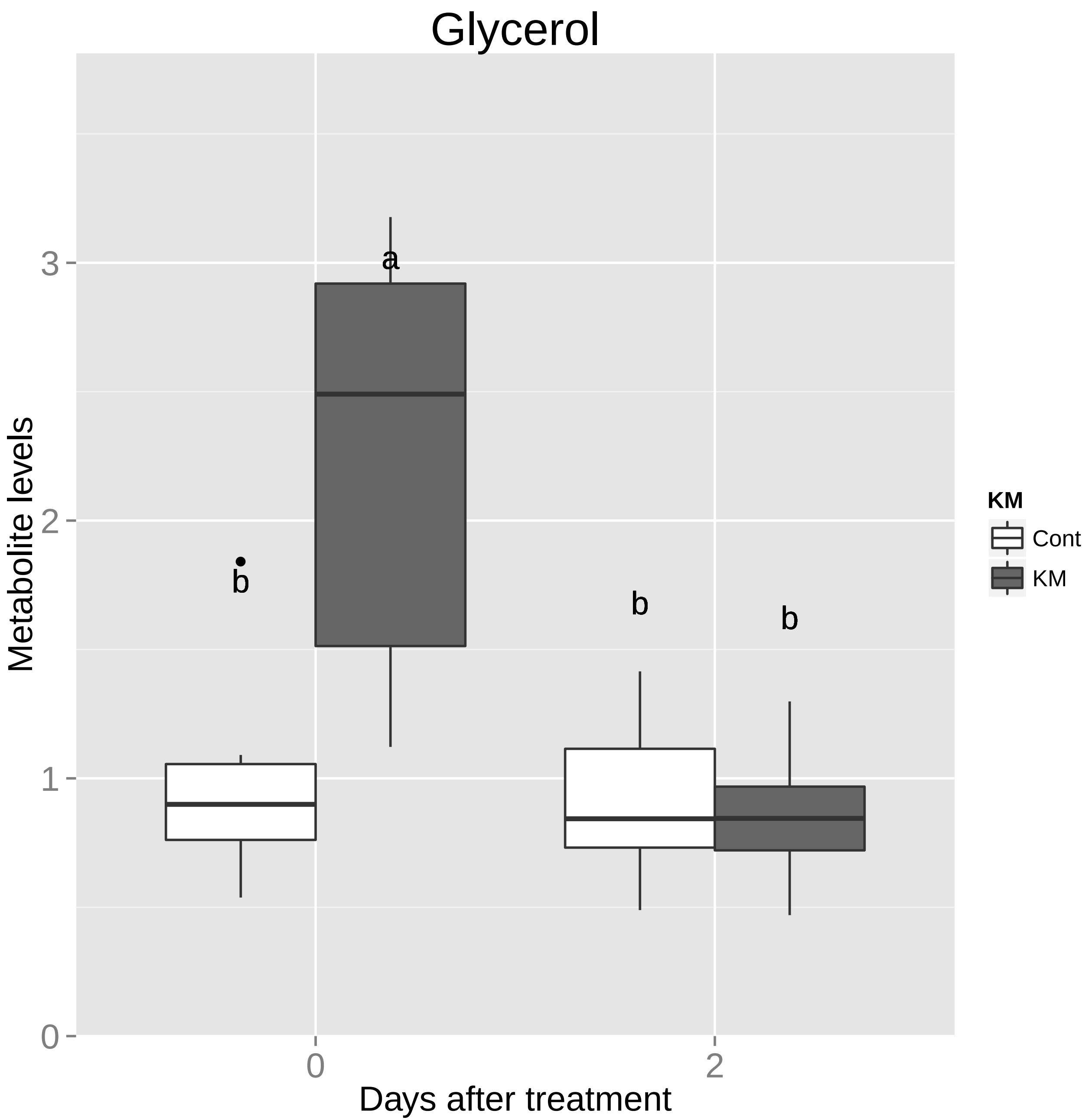
Glycerol
Metabolite levels
0
1
2
3
a
b
b
b
0
2
Days after treatment
KM
Cont
KM

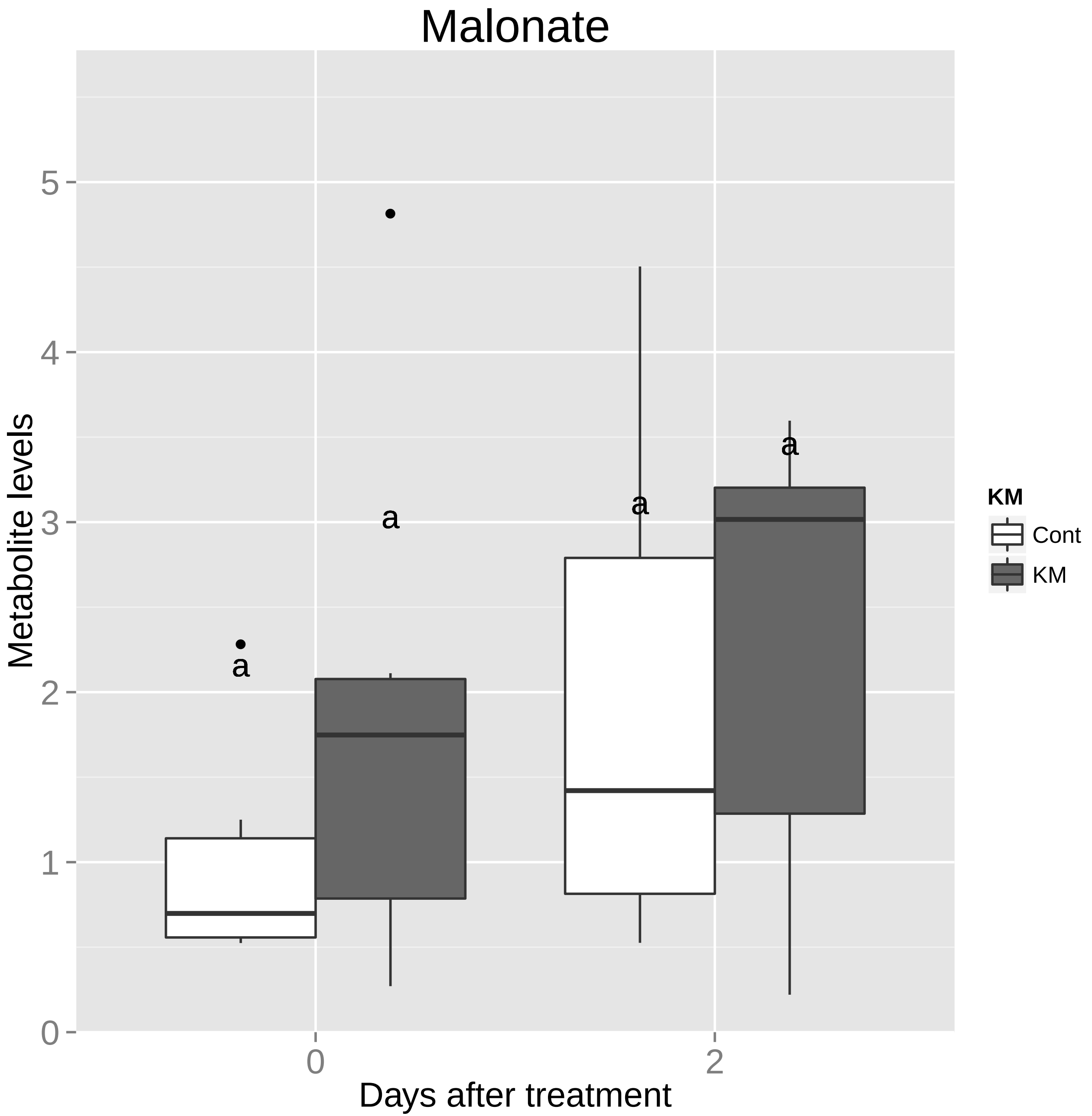
Malonate
Metabolite levels
0
1
2
3
4
5
a
a
a
a
0
2
Days after treatment
KM
Cont
KM

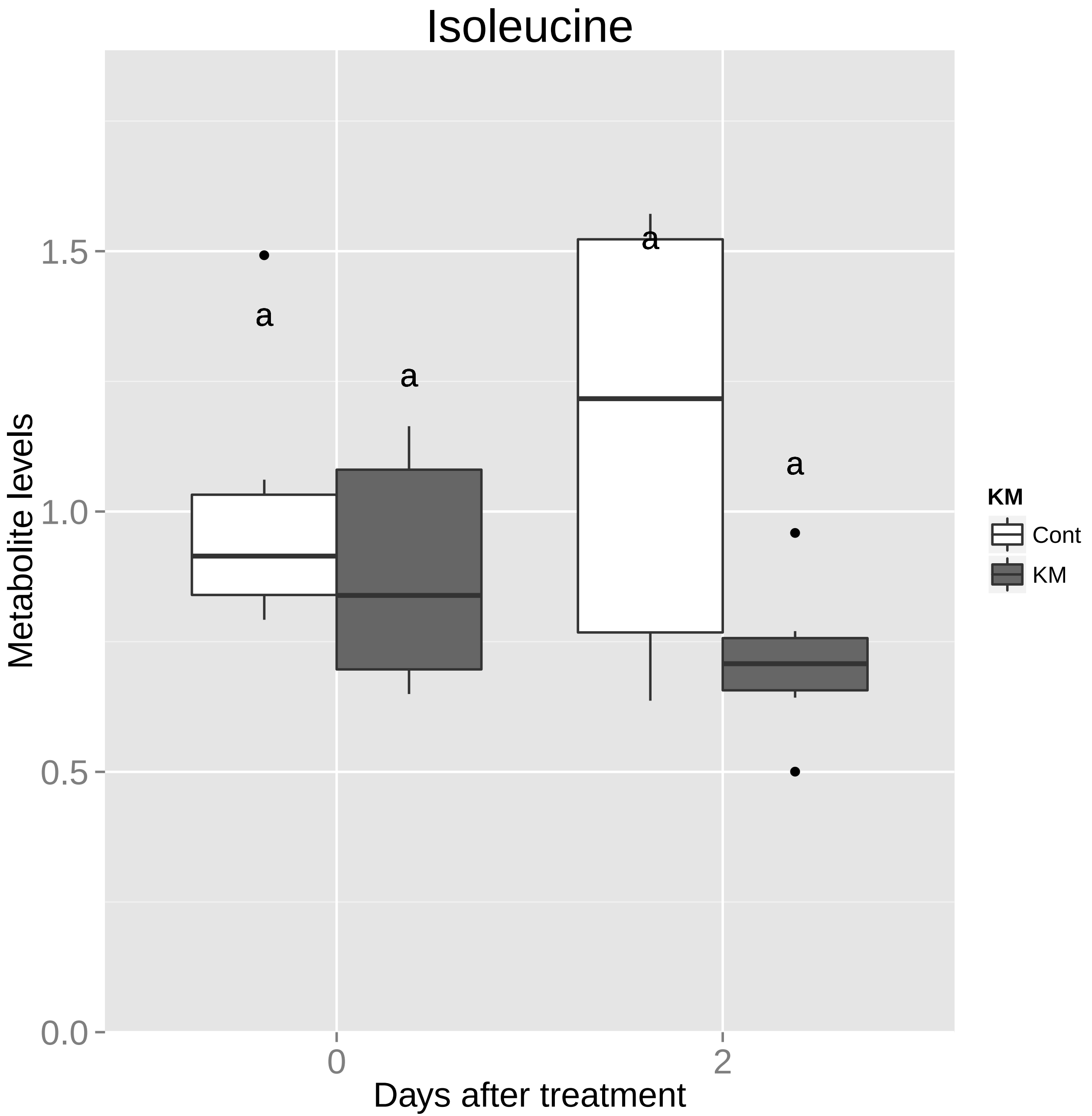

Isoleucine
Metabolite levels
Days after treatment
0.0
0.5
1.0
1.5
0
2
a
a
a
a
KM
Cont
KM

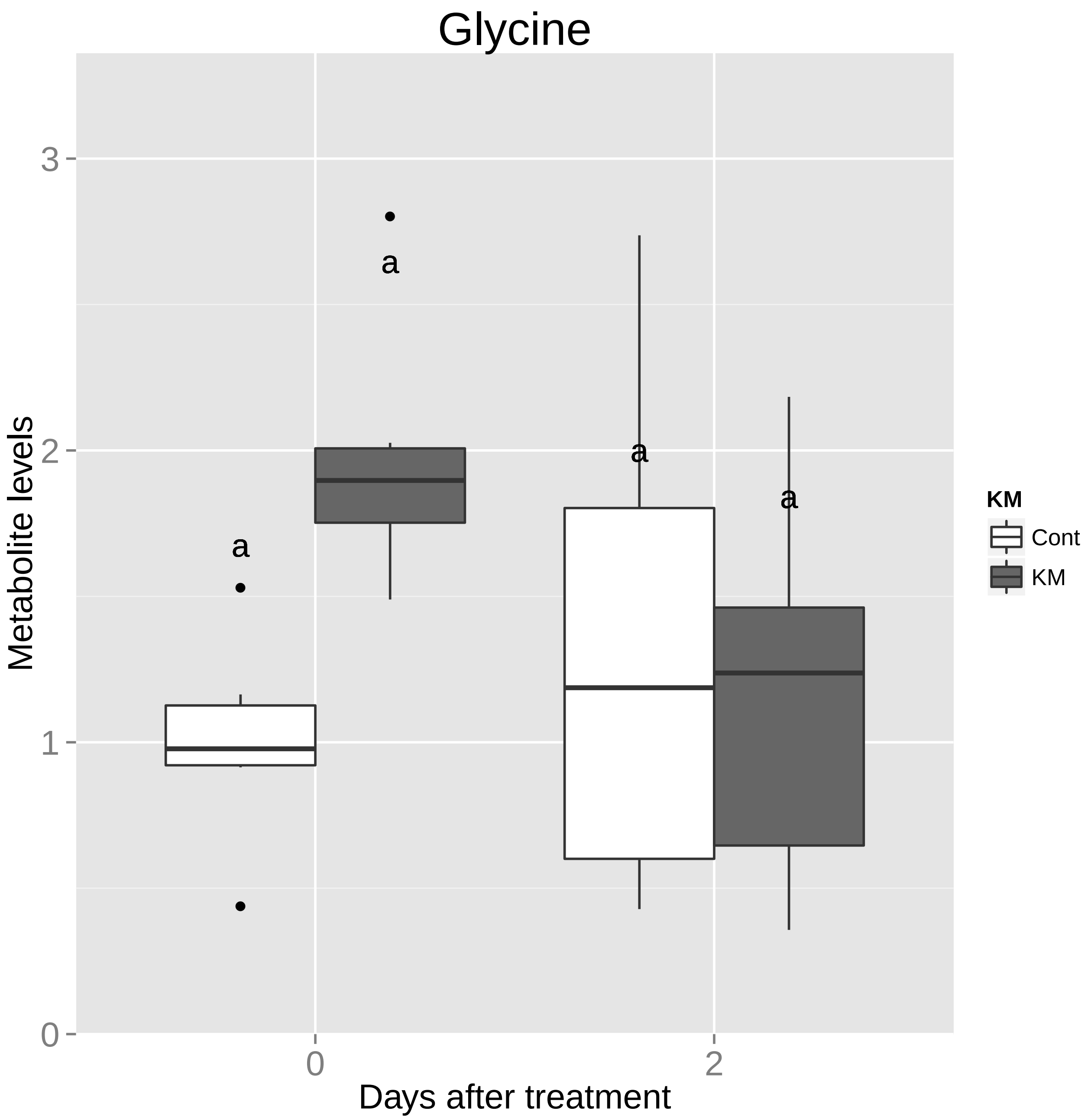

Glycine
Metabolite levels
Days after treatment
0
1
2
3
0
2
a
a
a
a
KM
Cont
KM

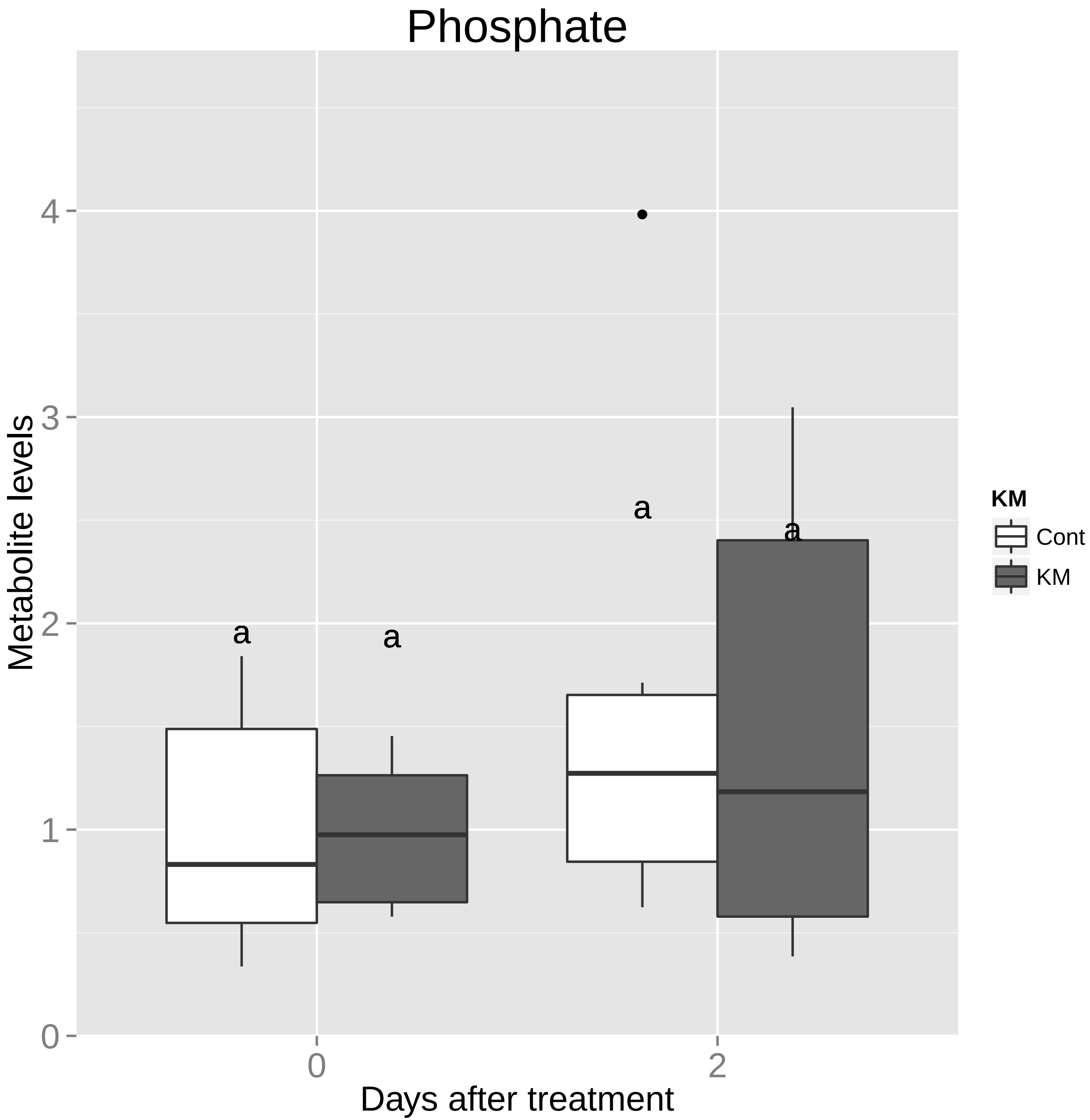
Phosphate
Metabolite levels
Days after treatment
0
1
2
3
4
0
2
a
a
a
a
KM
Cont
KM

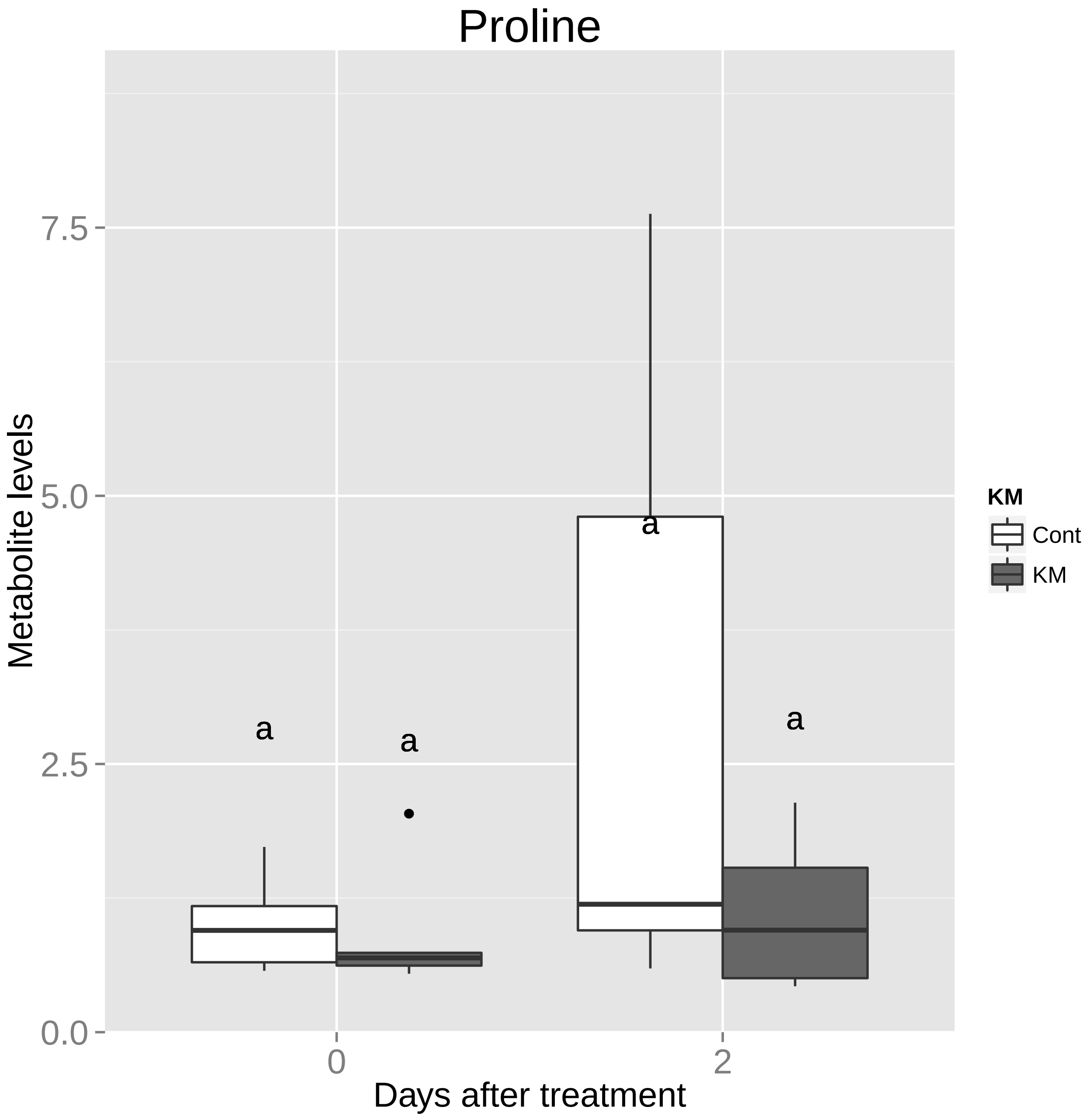
Proline
Metabolite levels
7.5
5.0
2.5
0.0
0
2
Days after treatment
a
a
a
a
KM
Cont
KM

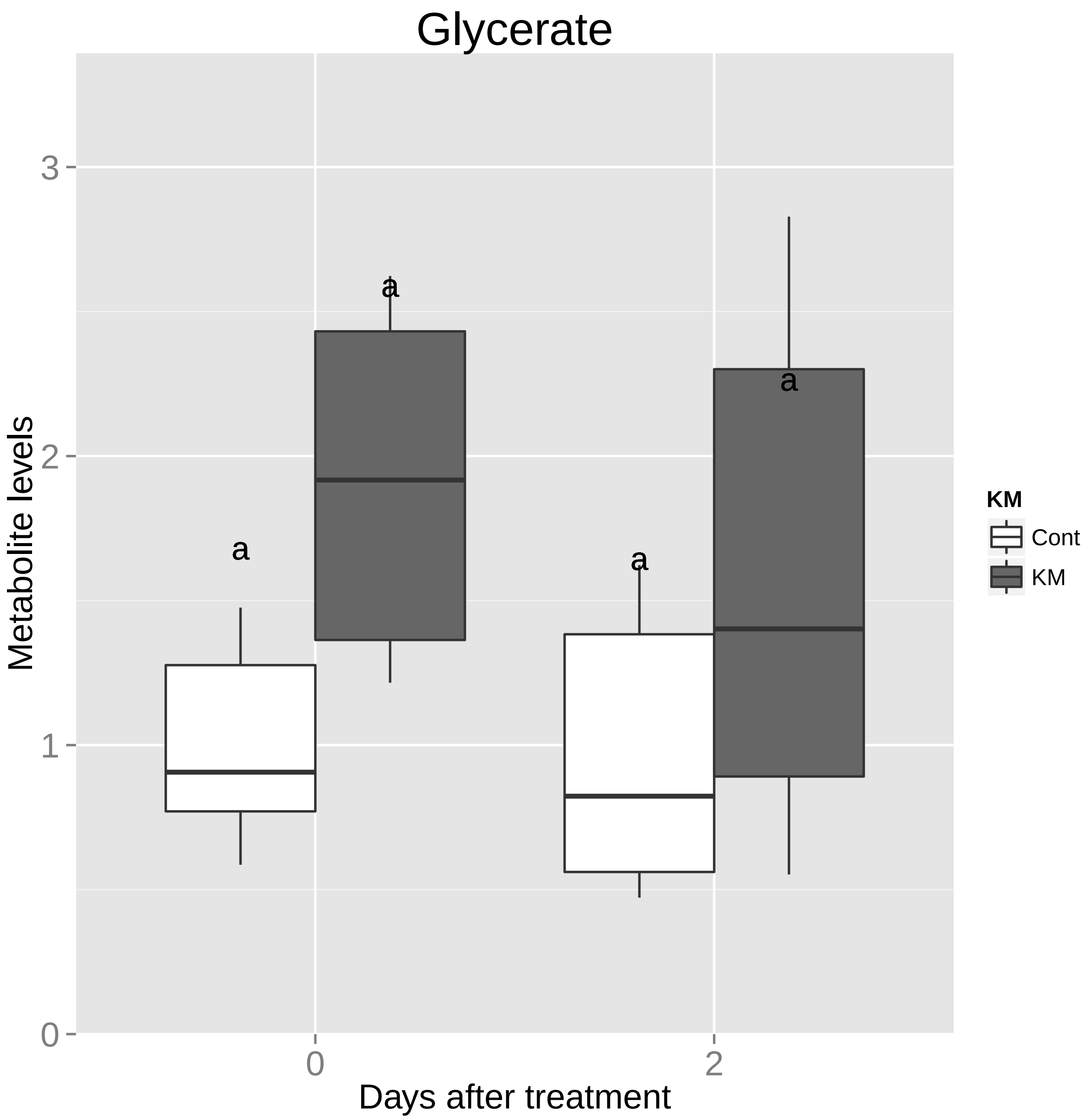
Glycerate
Metabolite levels
Days after treatment
0
1
2
3
0
2
a
a
a
a
KM
Cont
KM

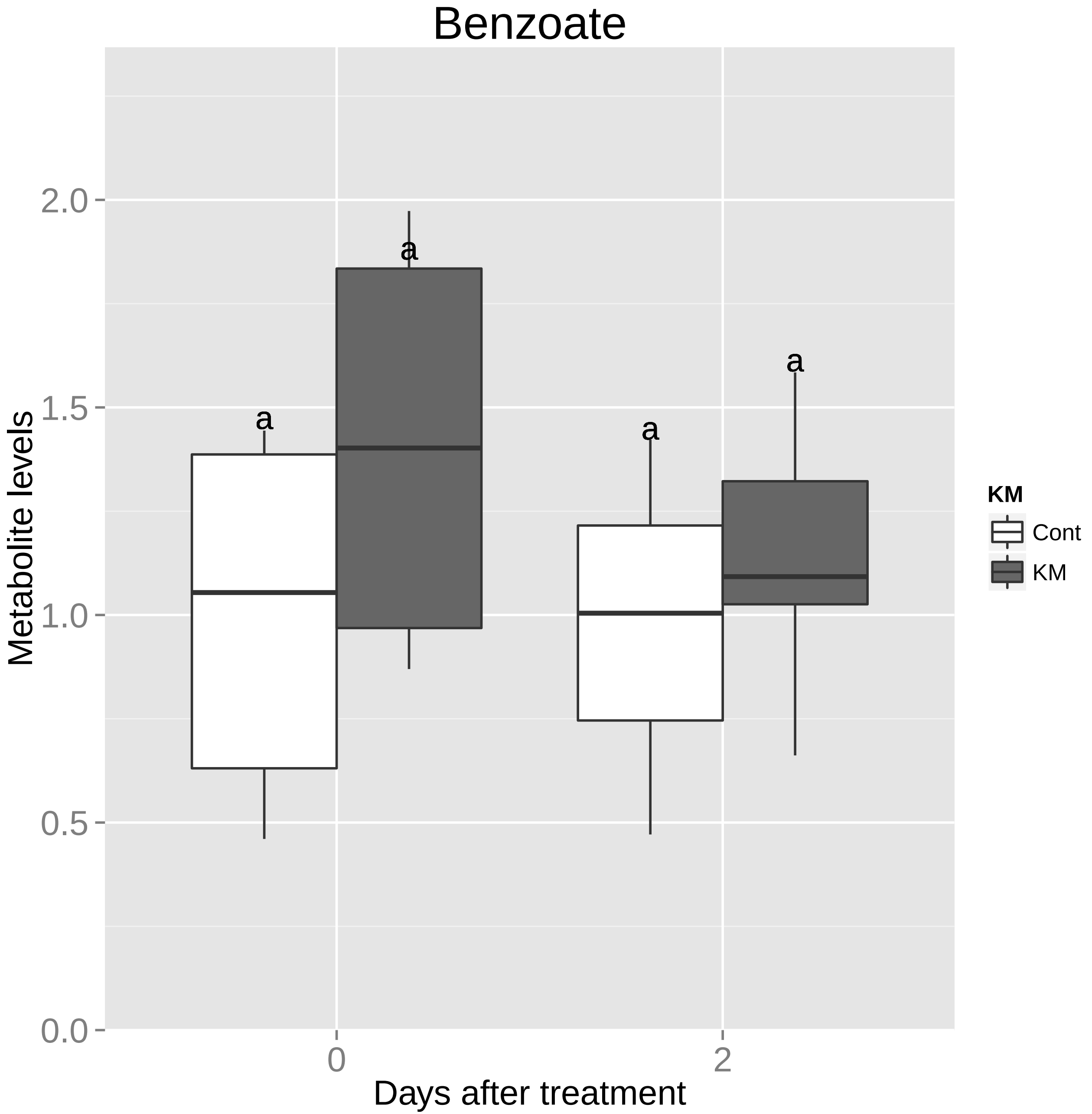
Benzoate
Metabolite levels
0.0
0.5
1.0
1.5
2.0
a
a
a
a
0
2
Days after treatment
KM
Cont
KM

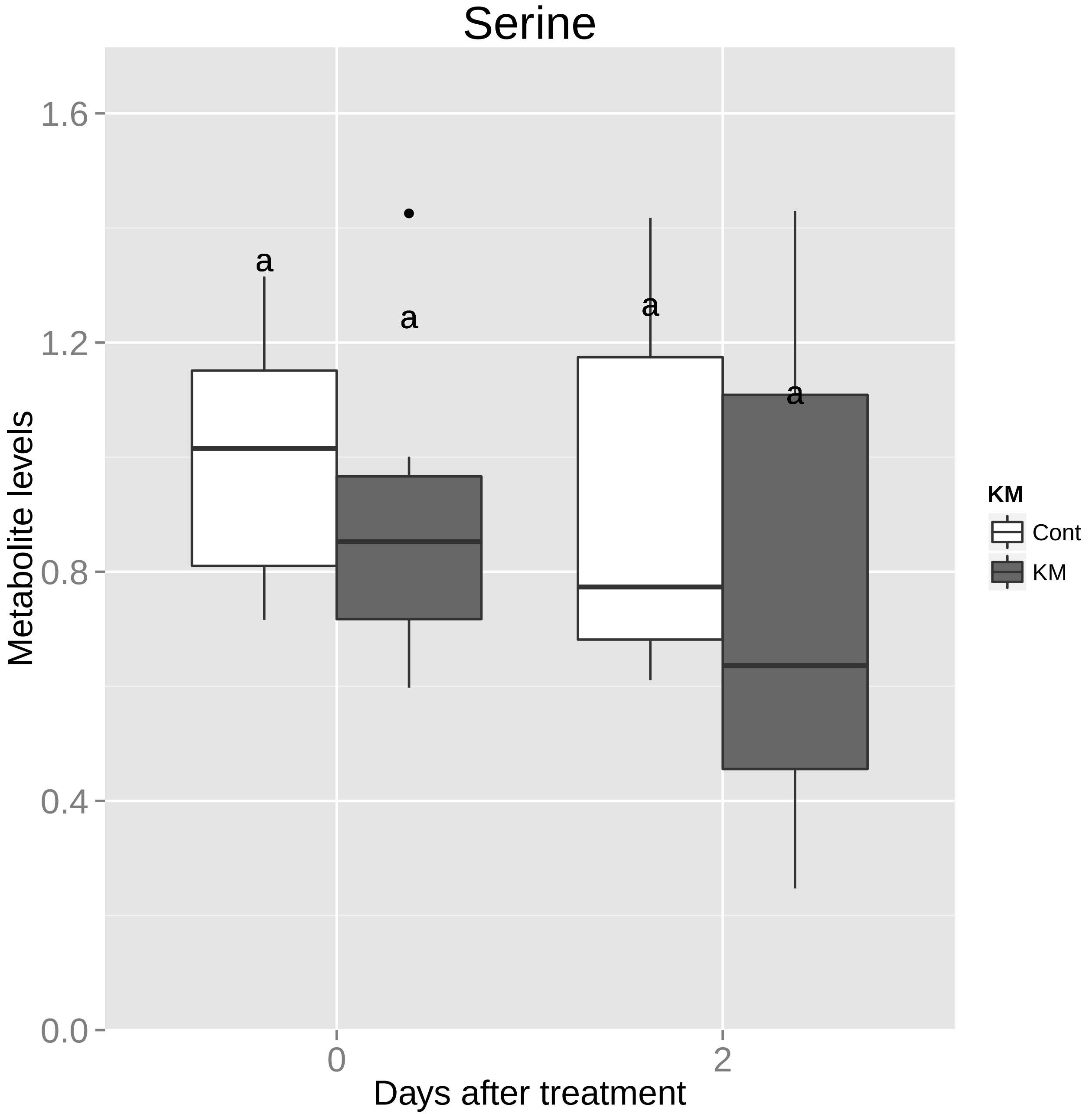

Serine
1.6
1.2
0.8
0.4
0.0
Metabolite levels
a
a
a
a
0
2
Days after treatment
KM
Cont
KM

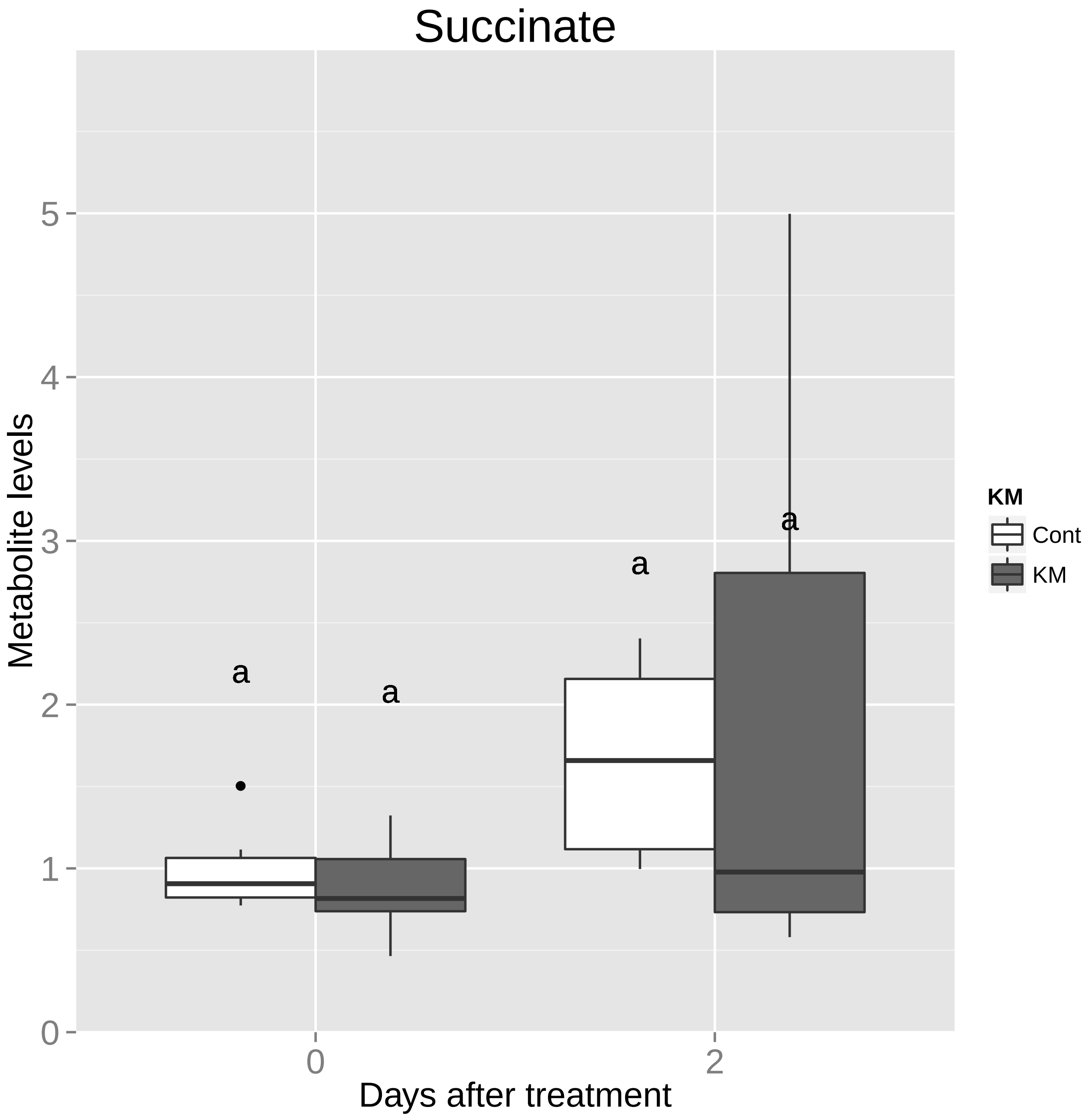
Succinate
Metabolite levels
0
1
2
3
4
5
a
a
a
a
0
2
Days after treatment
KM
Cont
KM

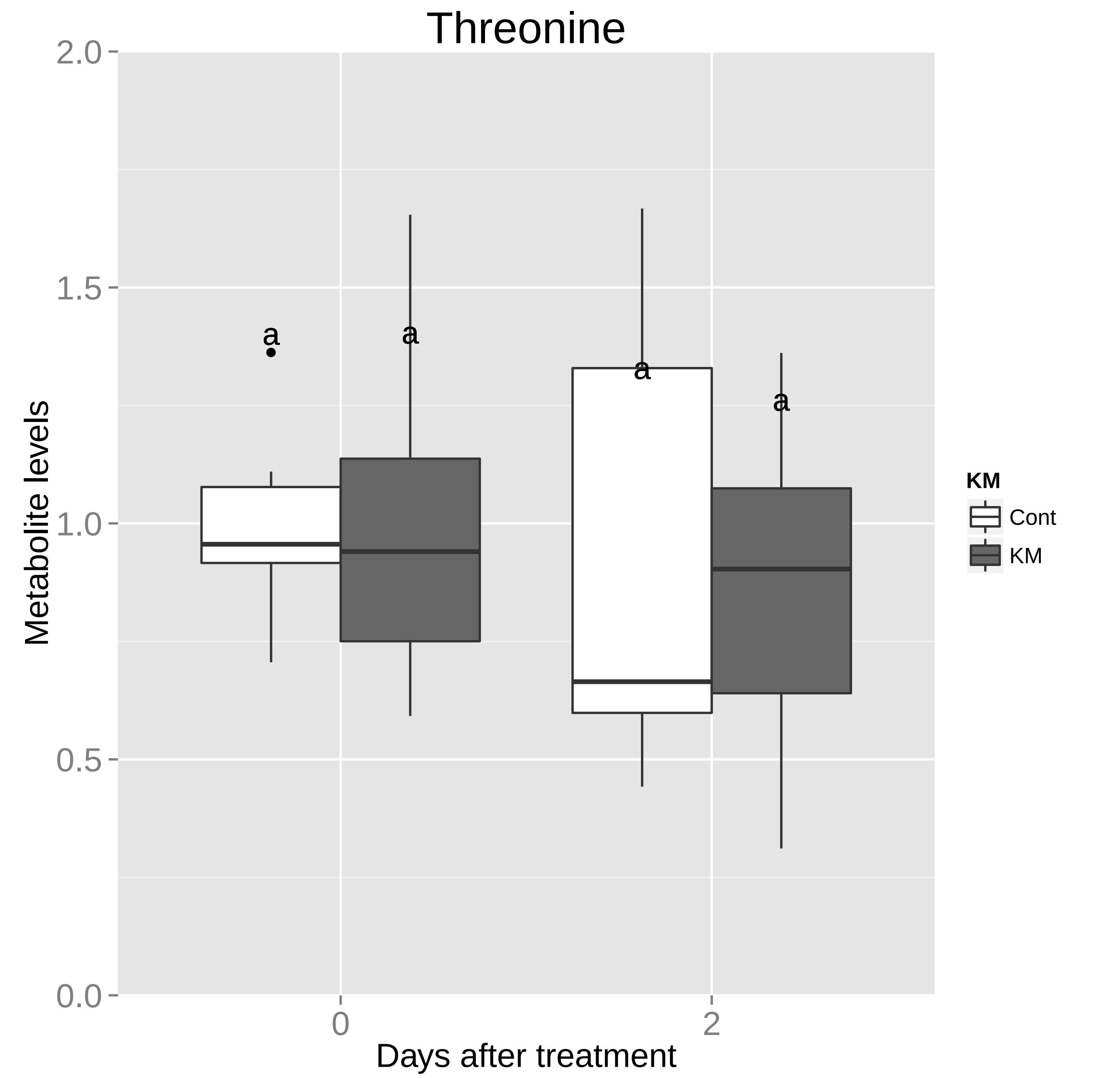

Threonine
2.0
1.5
1.0
0.5
0.0
Metabolite levels
a
a
a
a
0
2
Days after treatment
KM
Cont
KM

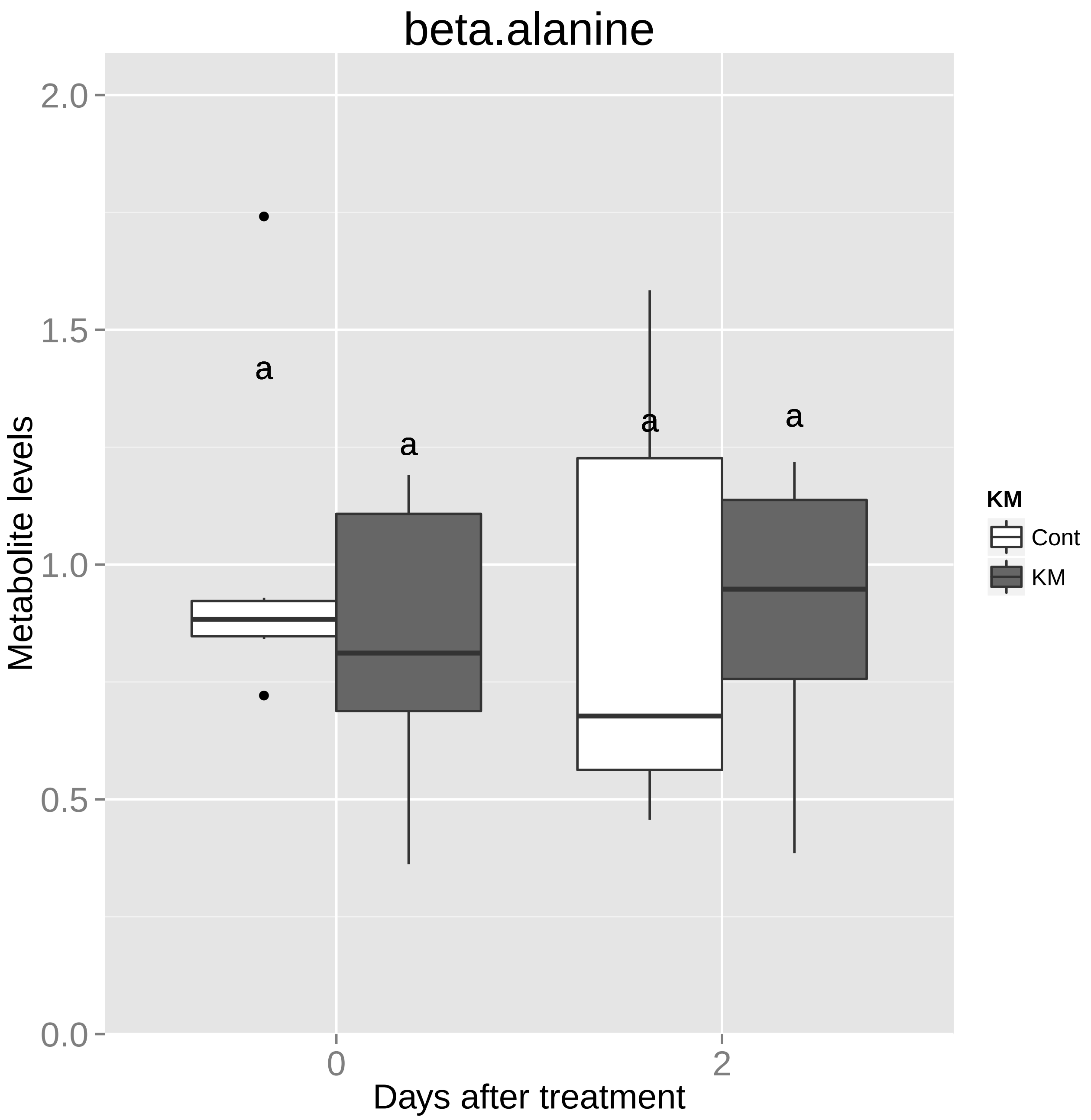

beta.alanine
2.0
1.5
1.0
0.5
0.0
Metabolite levels
a
a
a
a
0
2
Days after treatment
KM
Cont
KM

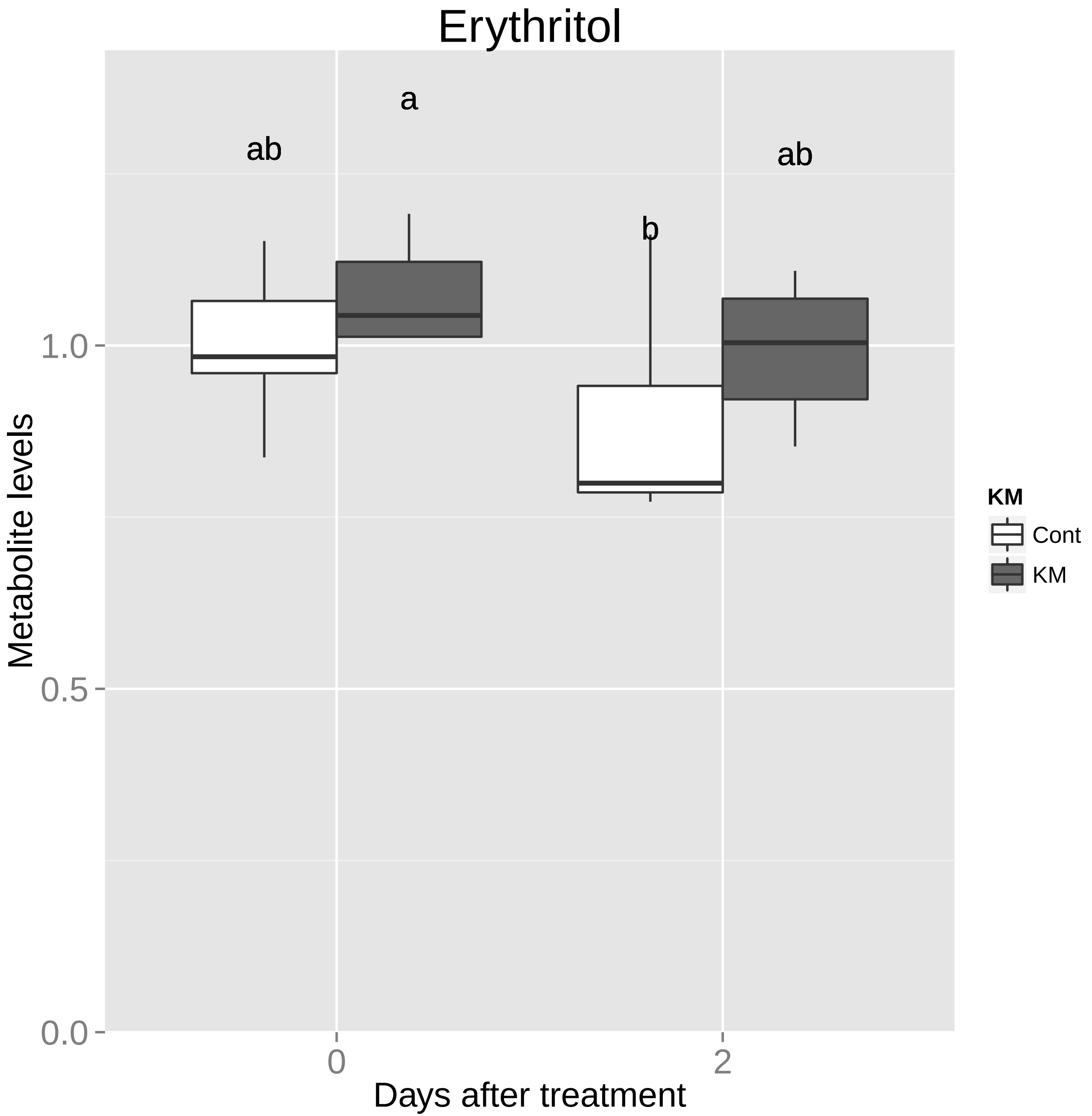

Erythritol
ab
a
ab
b
1.0
0.5
0.0
0
2
Metabolite levels
Days after treatment
KM
Cont
KM

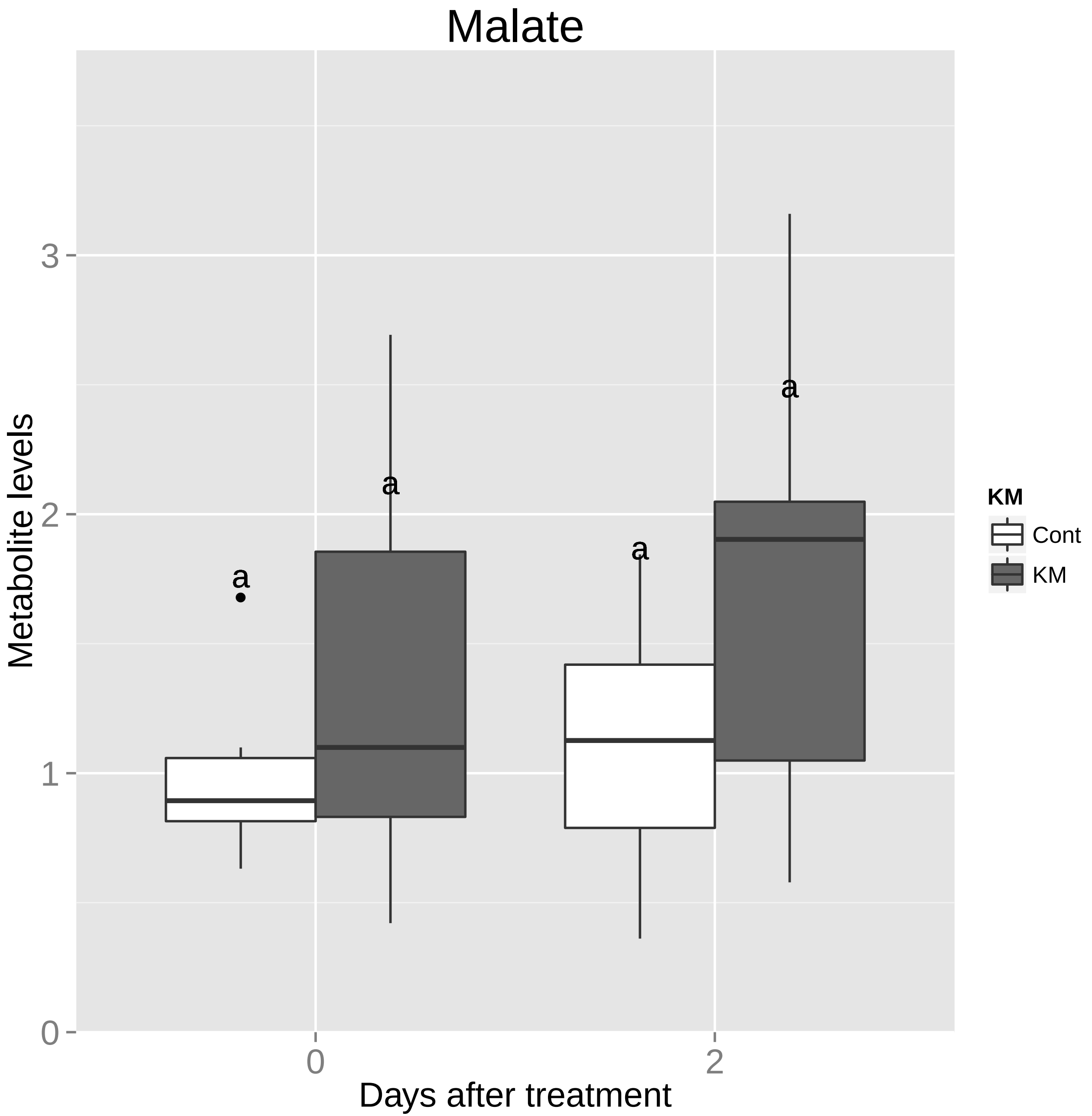
Malate
Metabolite levels
Days after treatment
0
1
2
3
0
2
a
a
a
a
KM
Cont
KM

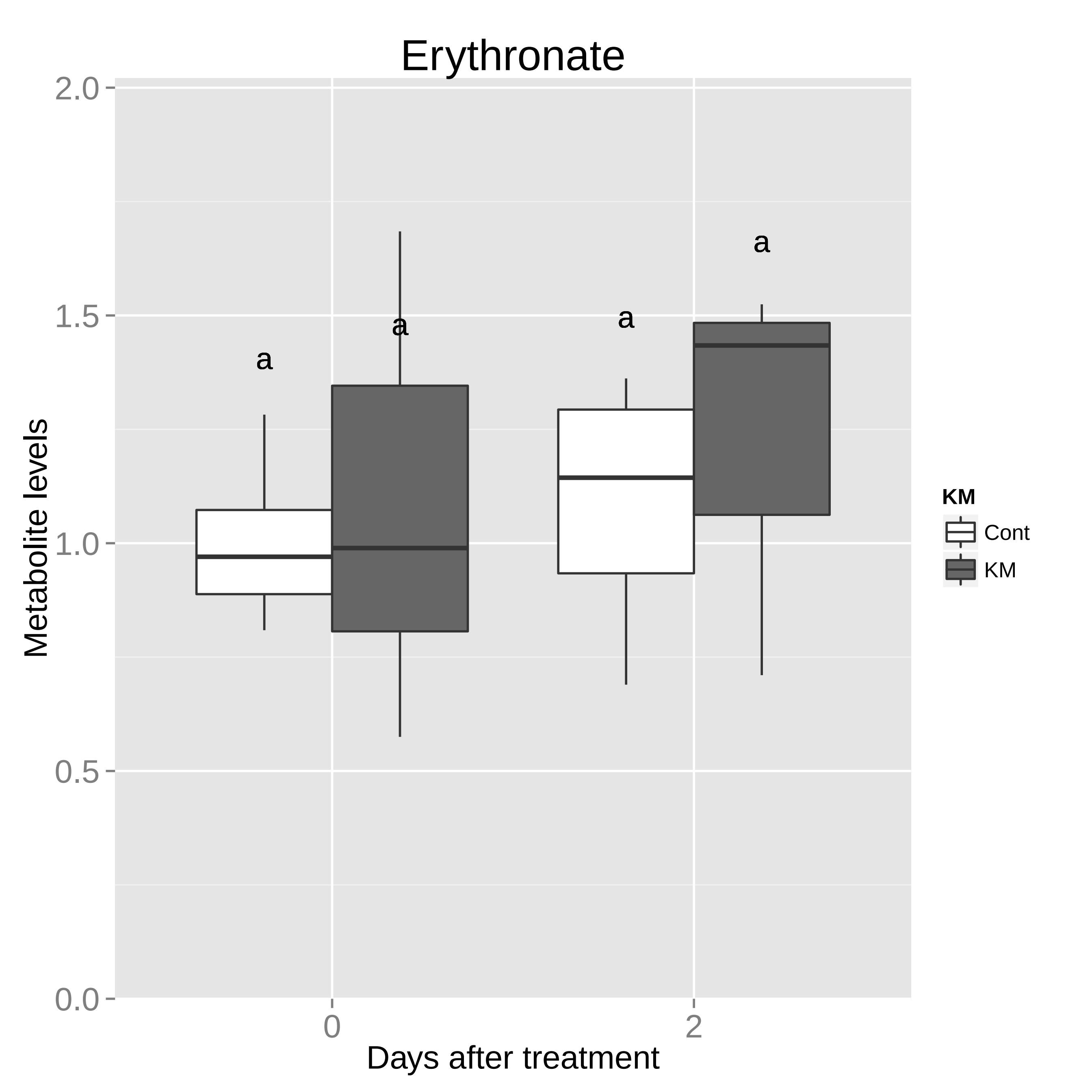

Erythronate
Metabolite levels
2.0
1.5
1.0
0.5
0.0
a
a
a
a
0
2
Days after treatment
KM
Cont
KM

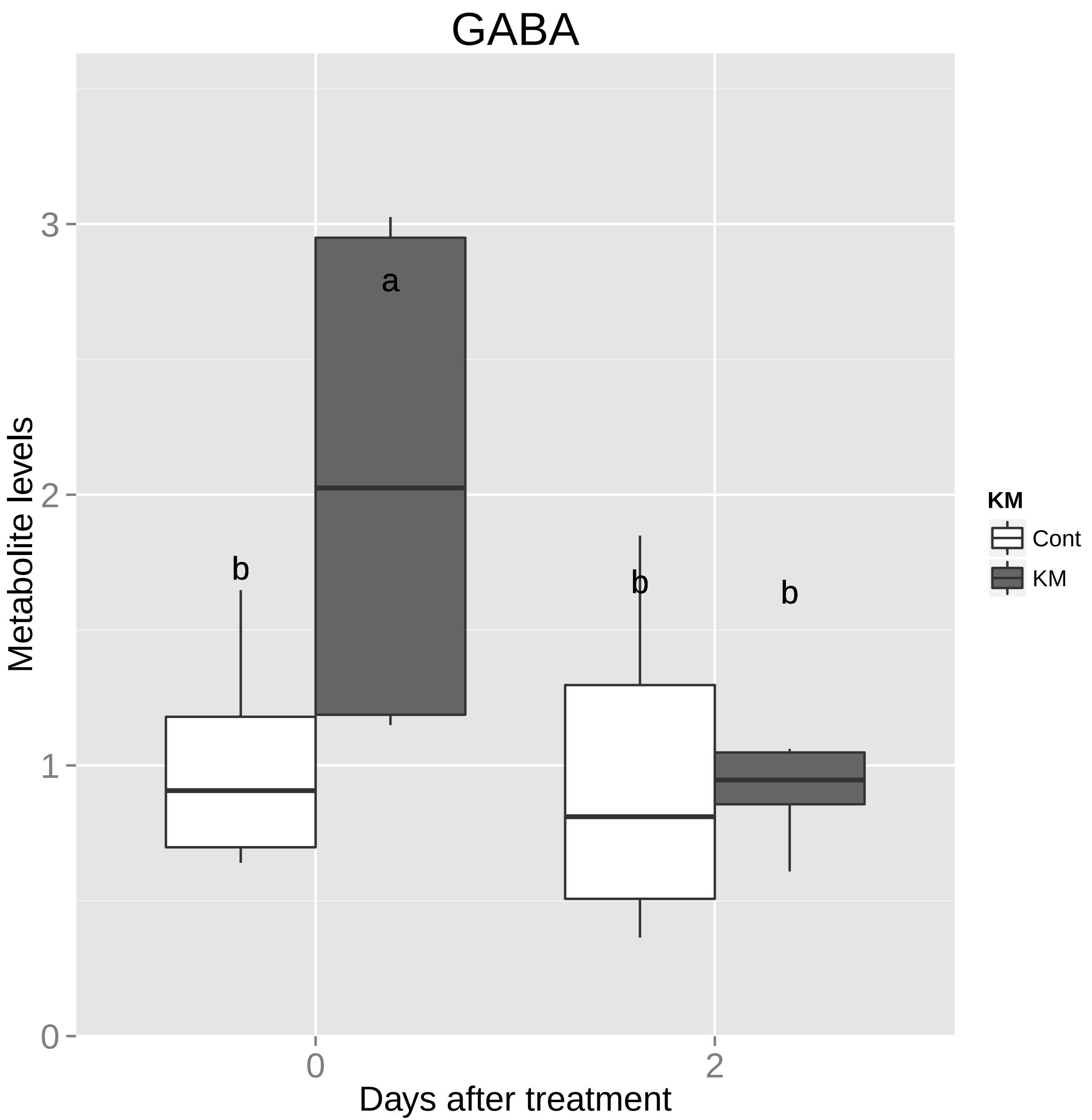

GABA
Metabolite levels
Days after treatment
0
1
2
3
0
2
a
b
b
b
KM
Cont
KM

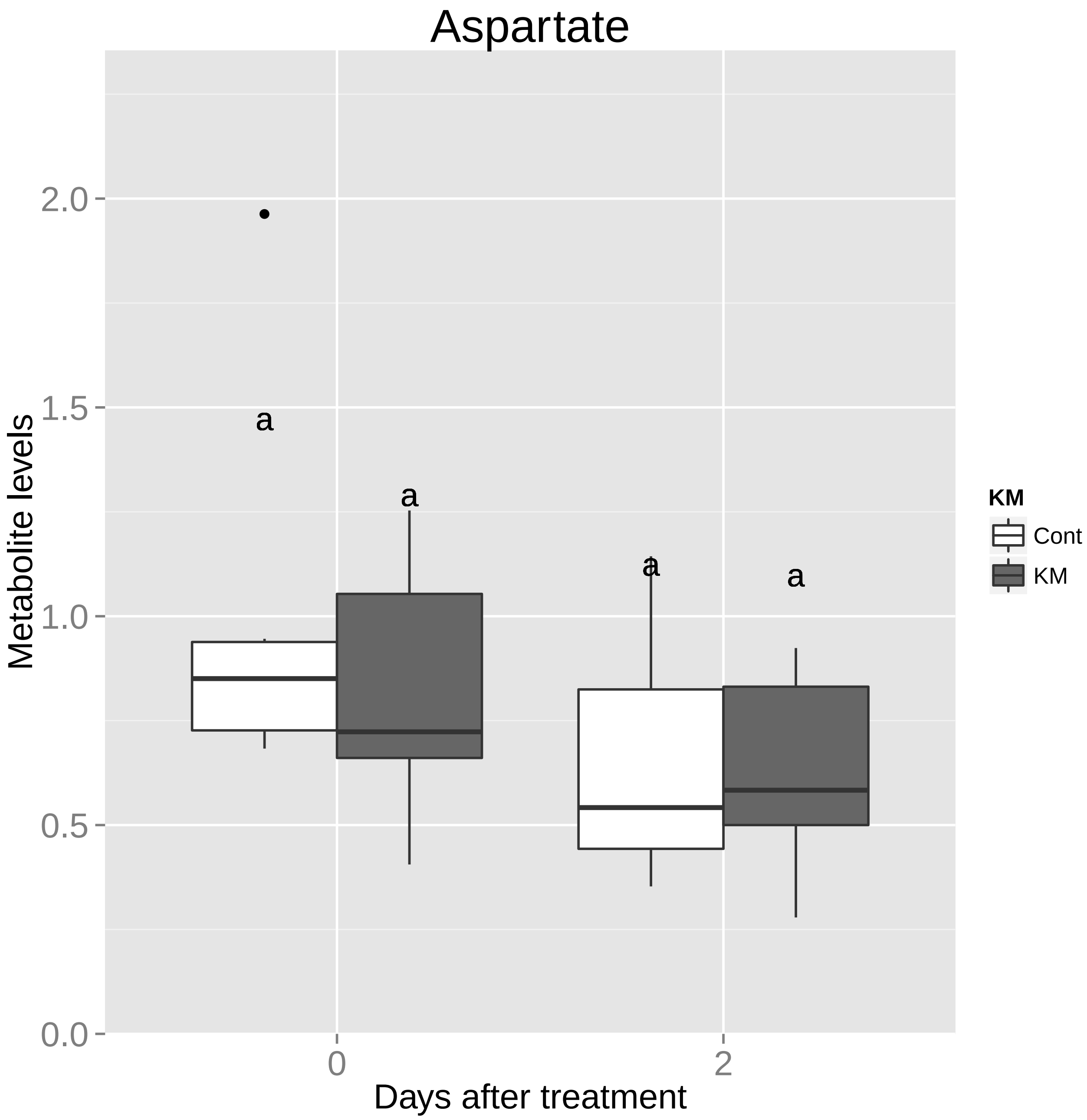
Aspartate
Metabolite levels
2.0
1.5
1.0
0.5
0.0
a
a
a
a
0
2
Days after treatment
KM
Cont
KM

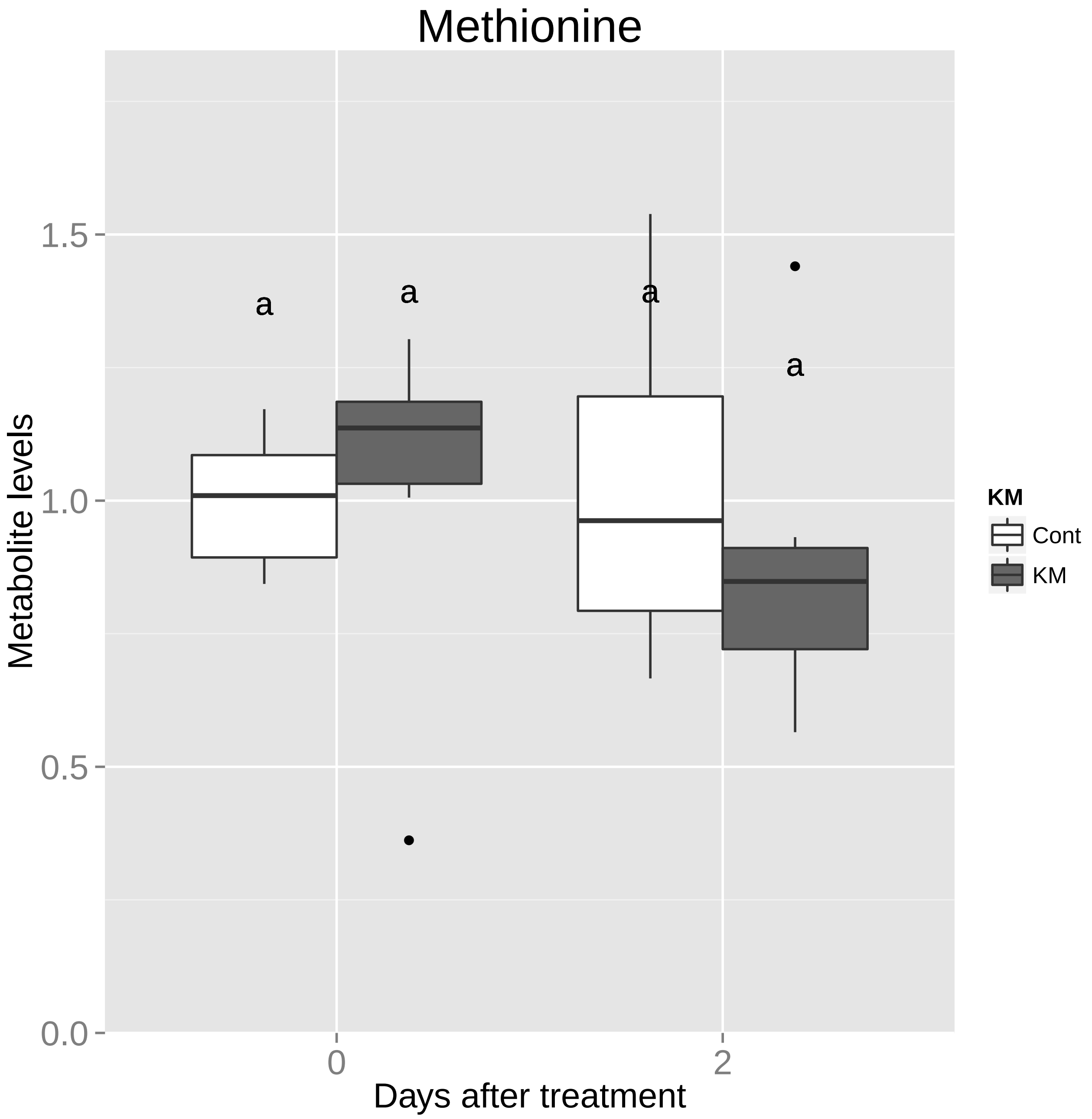
Methionine
Metabolite levels
1.5
1.0
0.5
0.0
a
a
a
a
0
2
Days after treatment
KM
Cont
KM

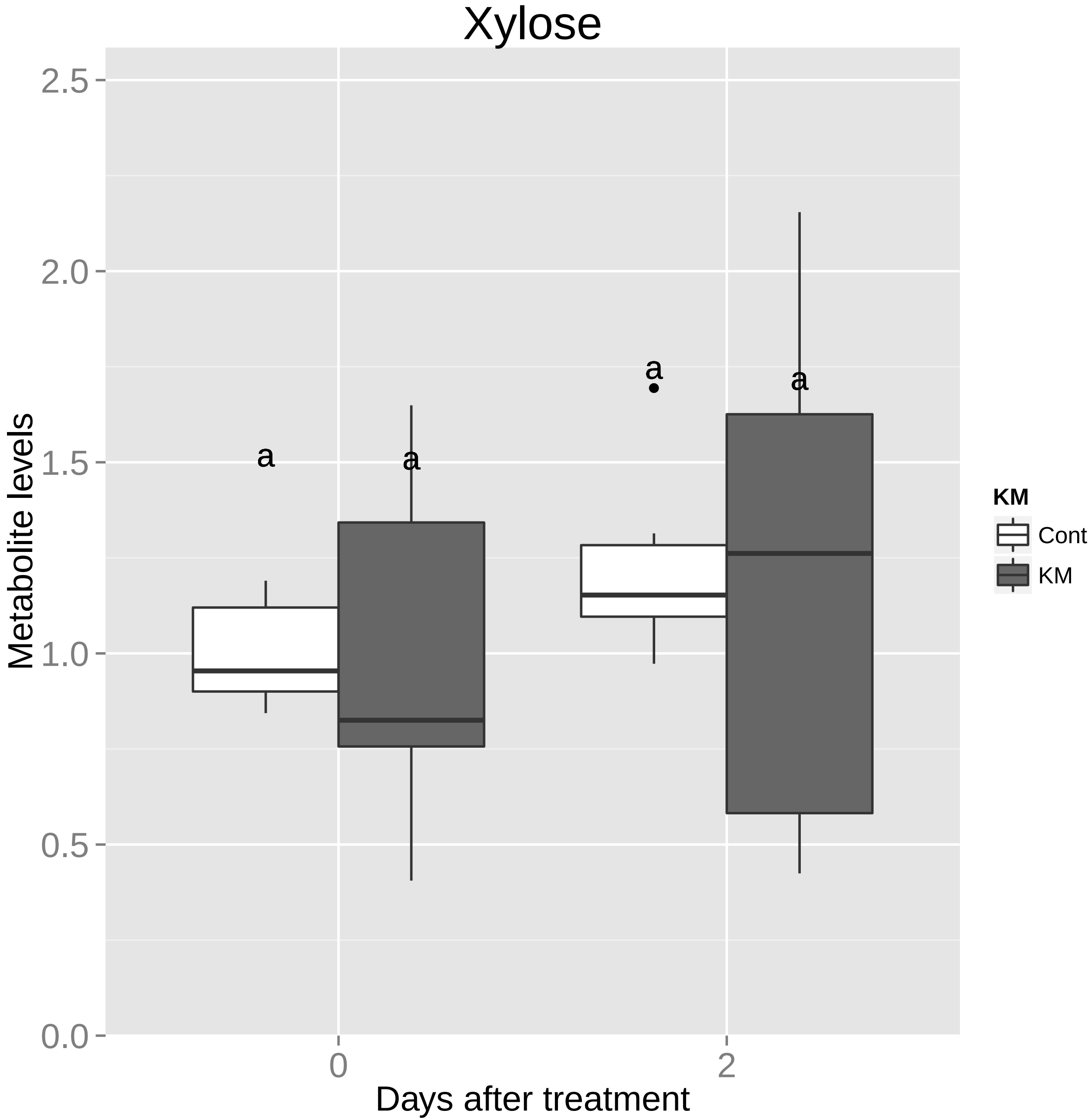

Xylose
Metabolite levels
2.5
2.0
1.5
1.0
0.5
0.0
a
a
a
a
0
2
Days after treatment
KM
Cont
KM

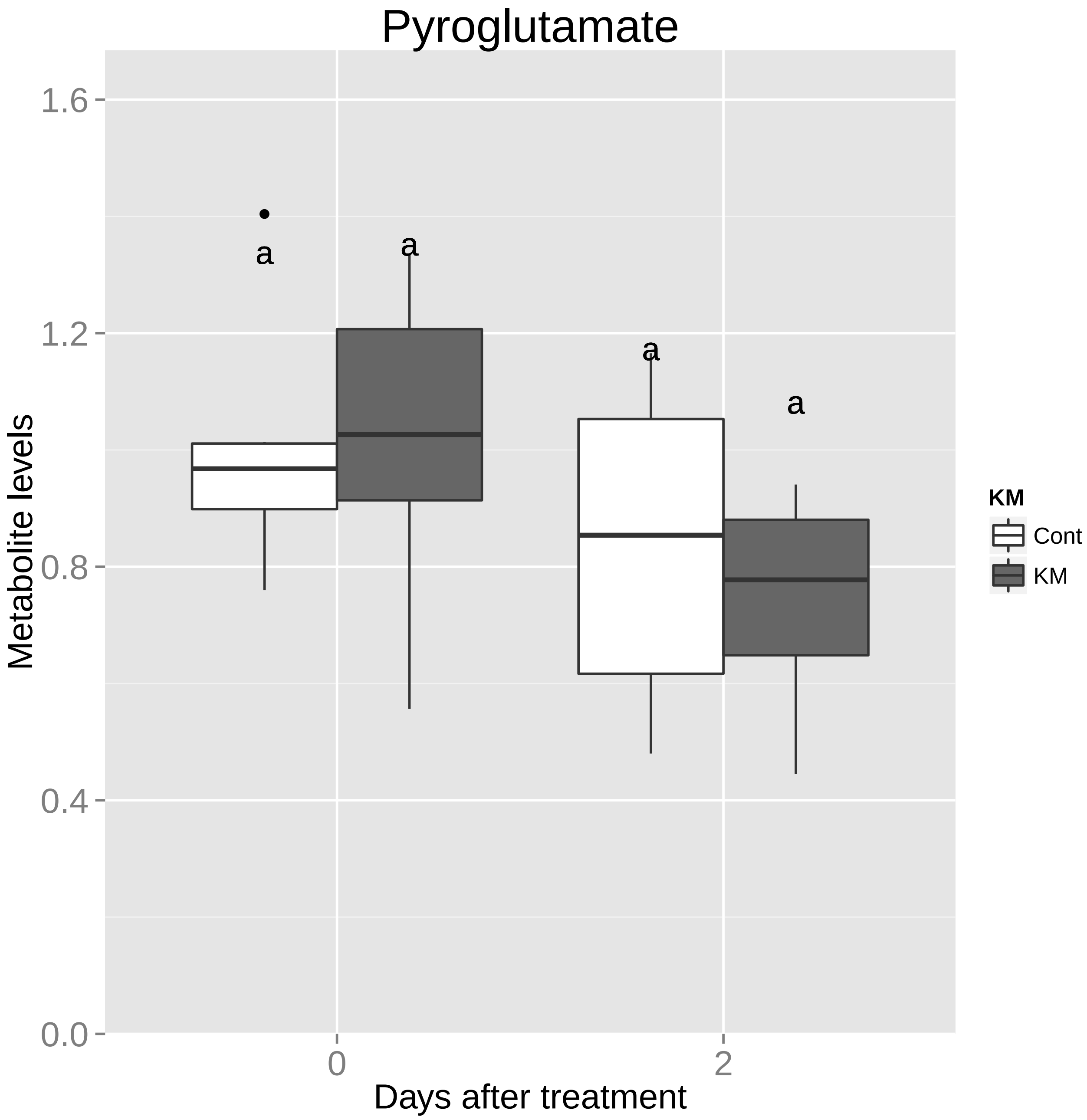
Pyroglutamate
1.6
1.2
0.8
0.4
0.0
Metabolite levels
a
a
a
a
0
2
Days after treatment
KM
Cont
KM

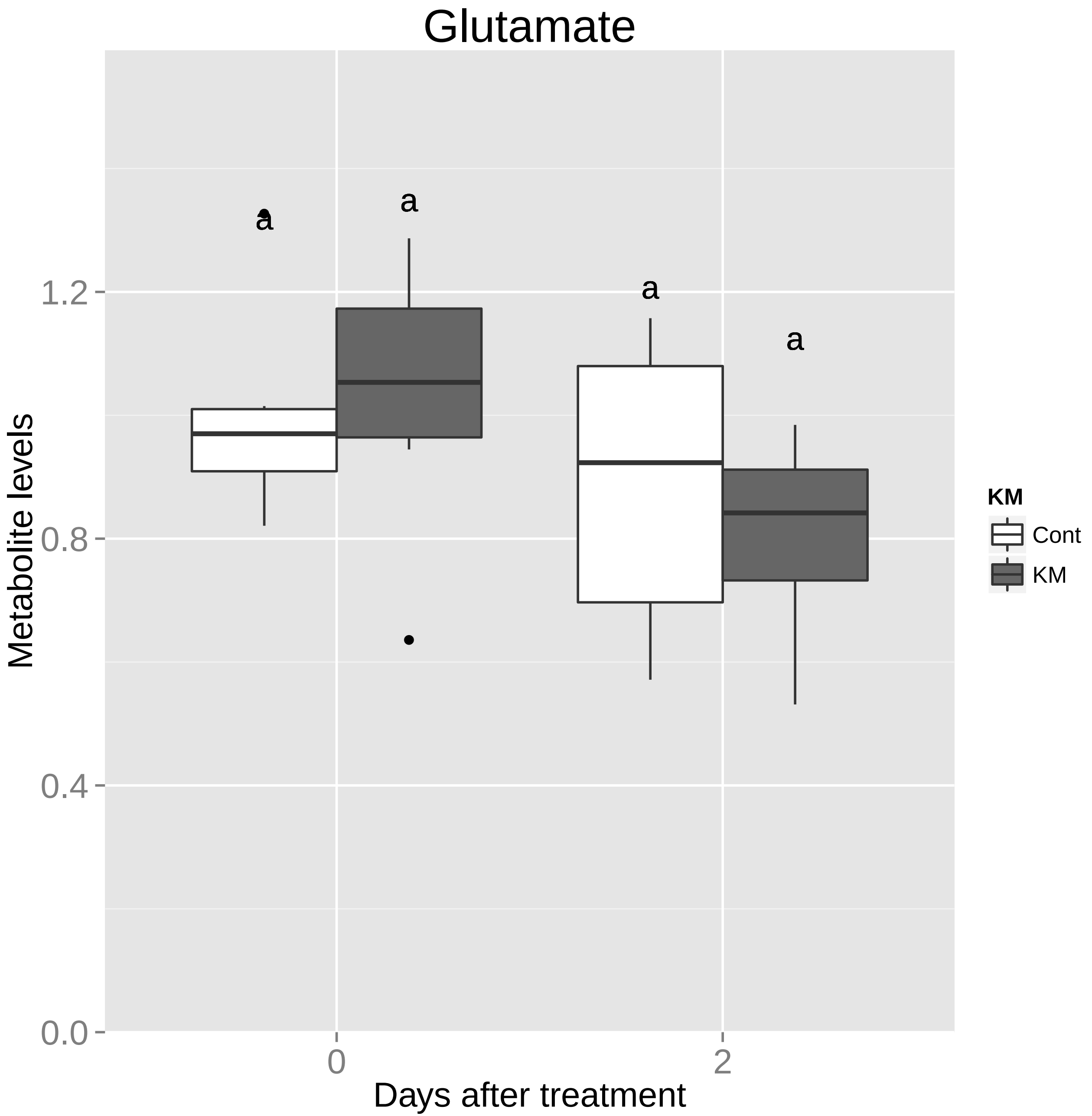

Glutamate
Metabolite levels
Days after treatment
0.0
0.4
0.8
1.2
0
2
a
a
a
a
KM
Cont
KM

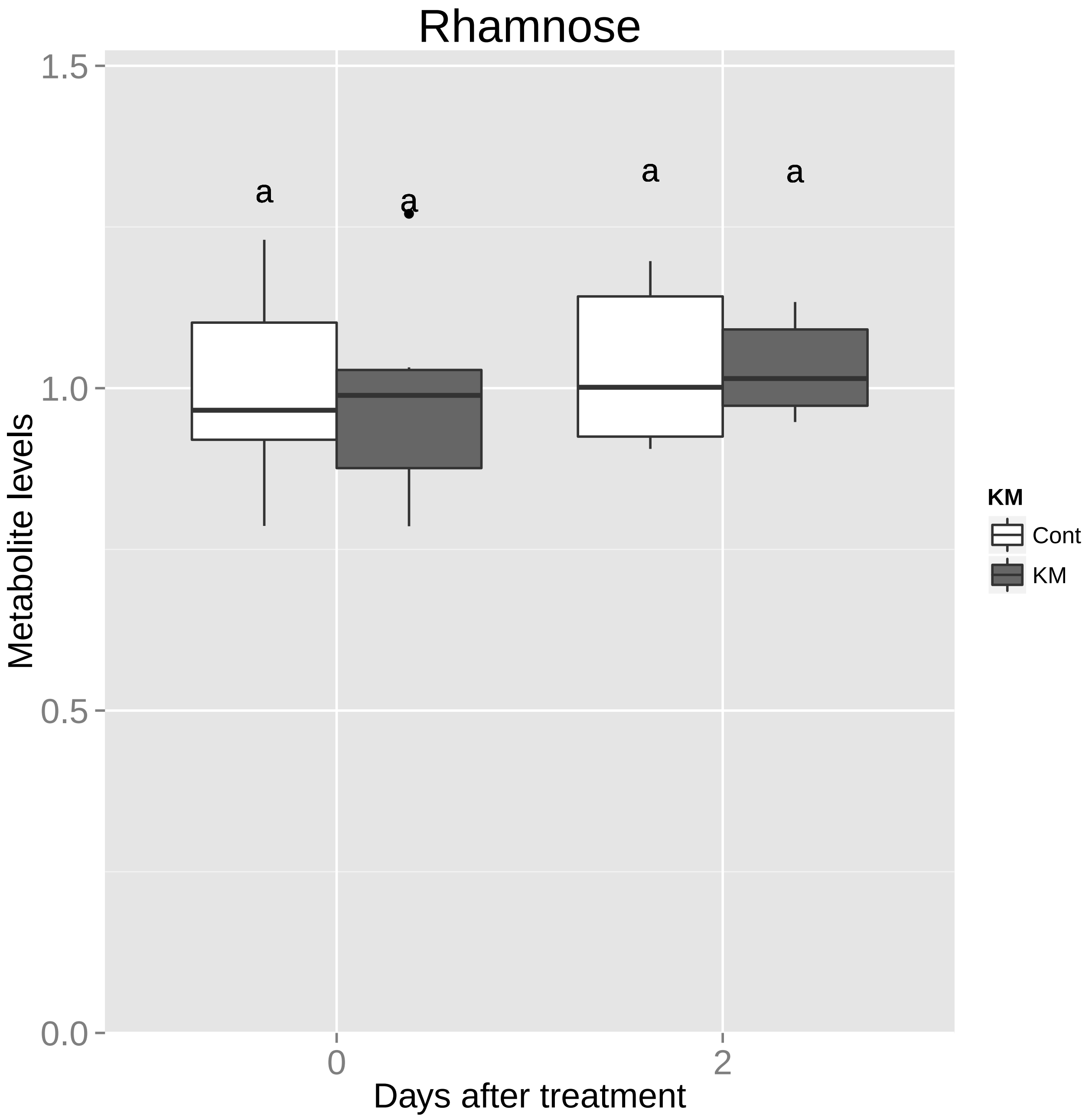

Rhamnose
Metabolite levels
1.5
1.0
0.5
0.0
a
a
a
a
0
2
Days after treatment
KM
Cont
KM

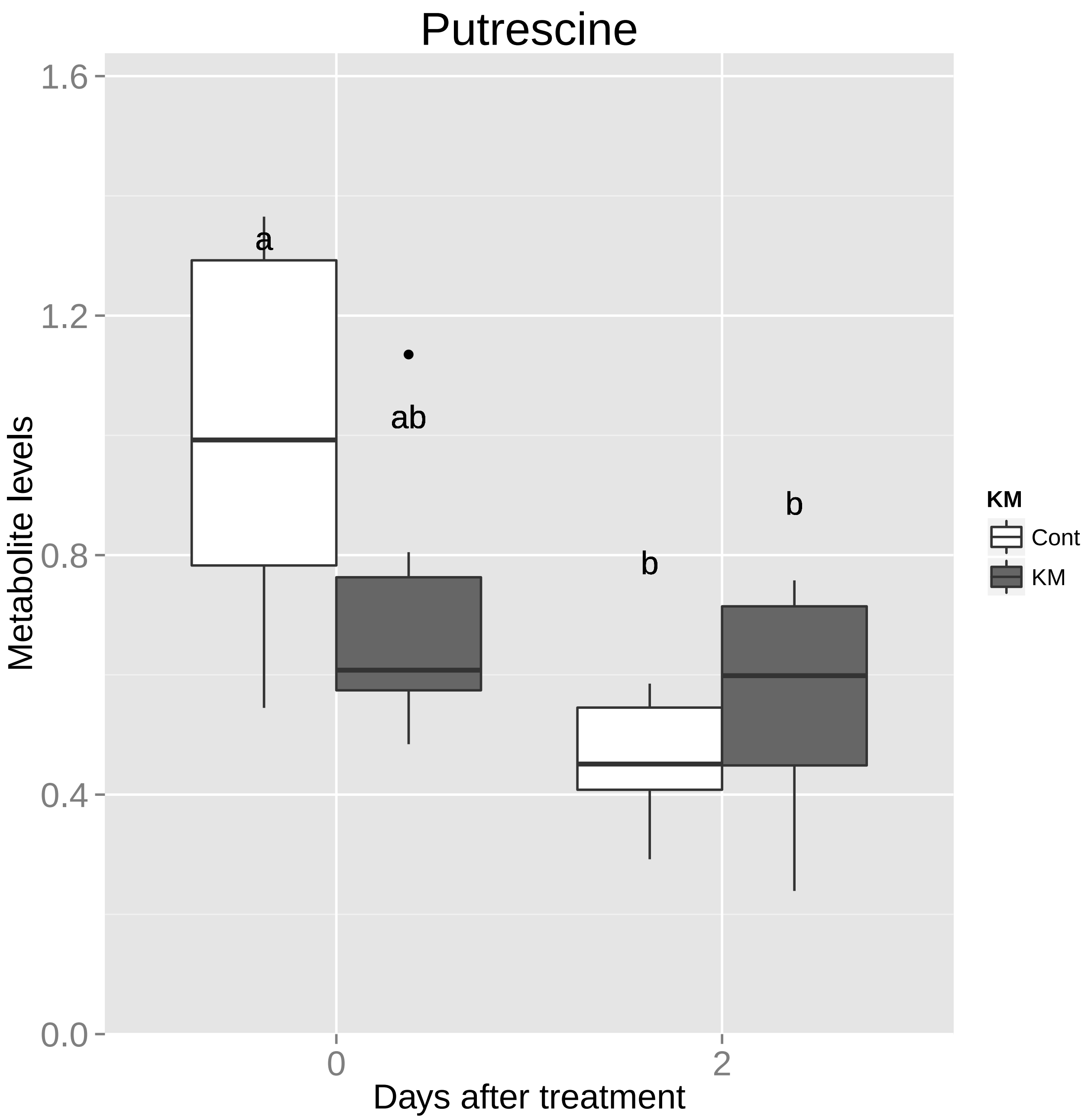
Putrescine
Metabolite levels
Days after treatment
1.6
1.2
0.8
0.4
0.0
0
2
a
ab
b
b
KM
Cont
KM

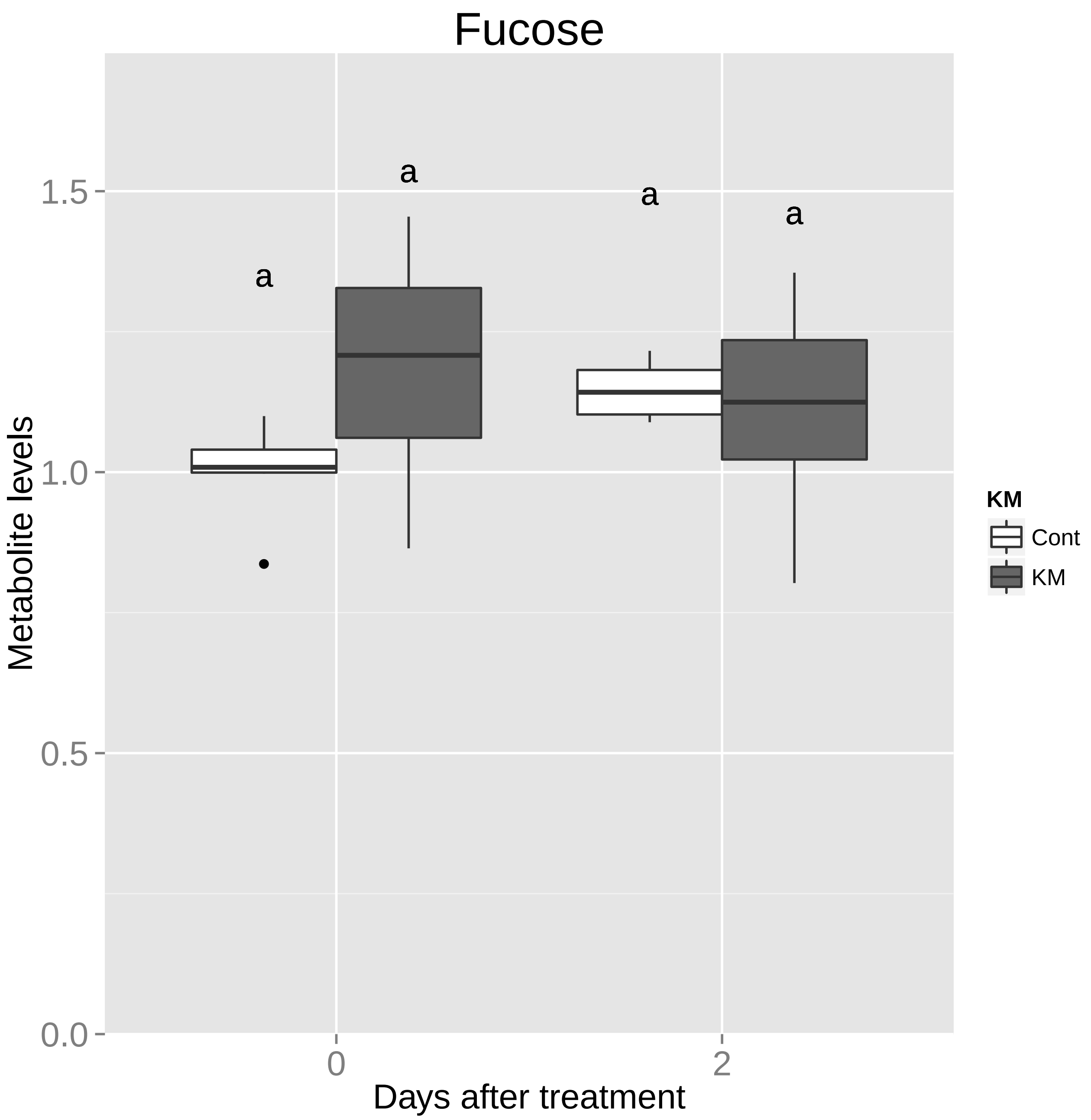

Fucose
a
a
a
a
1.5
1.0
0.5
0.0
Metabolite levels
0
2
Days after treatment
KM
Cont
KM

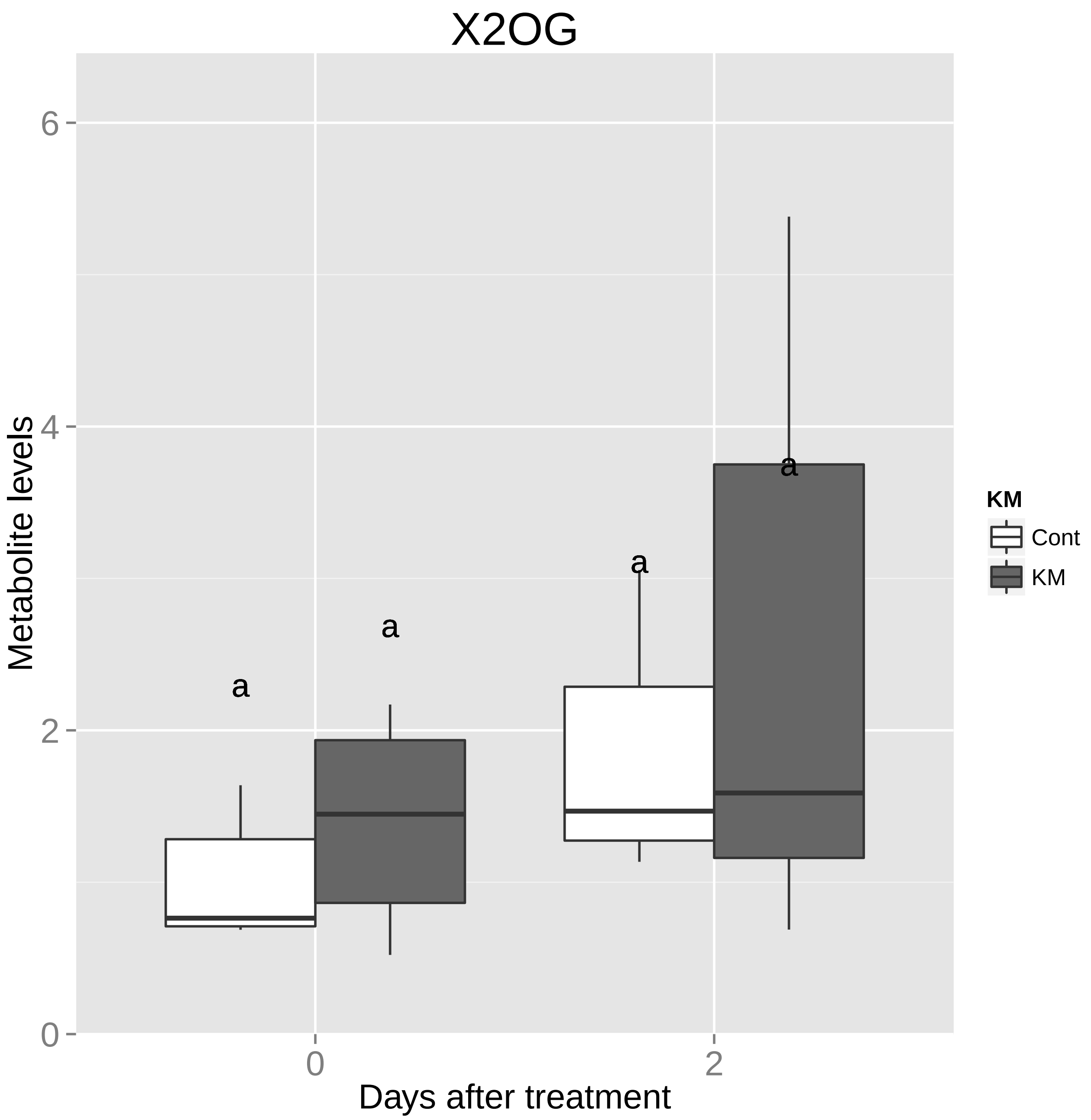
X2OG
Metabolite levels
Days after treatment
0
2
4
6
0
2
a
a
a
a
KM
Cont
KM

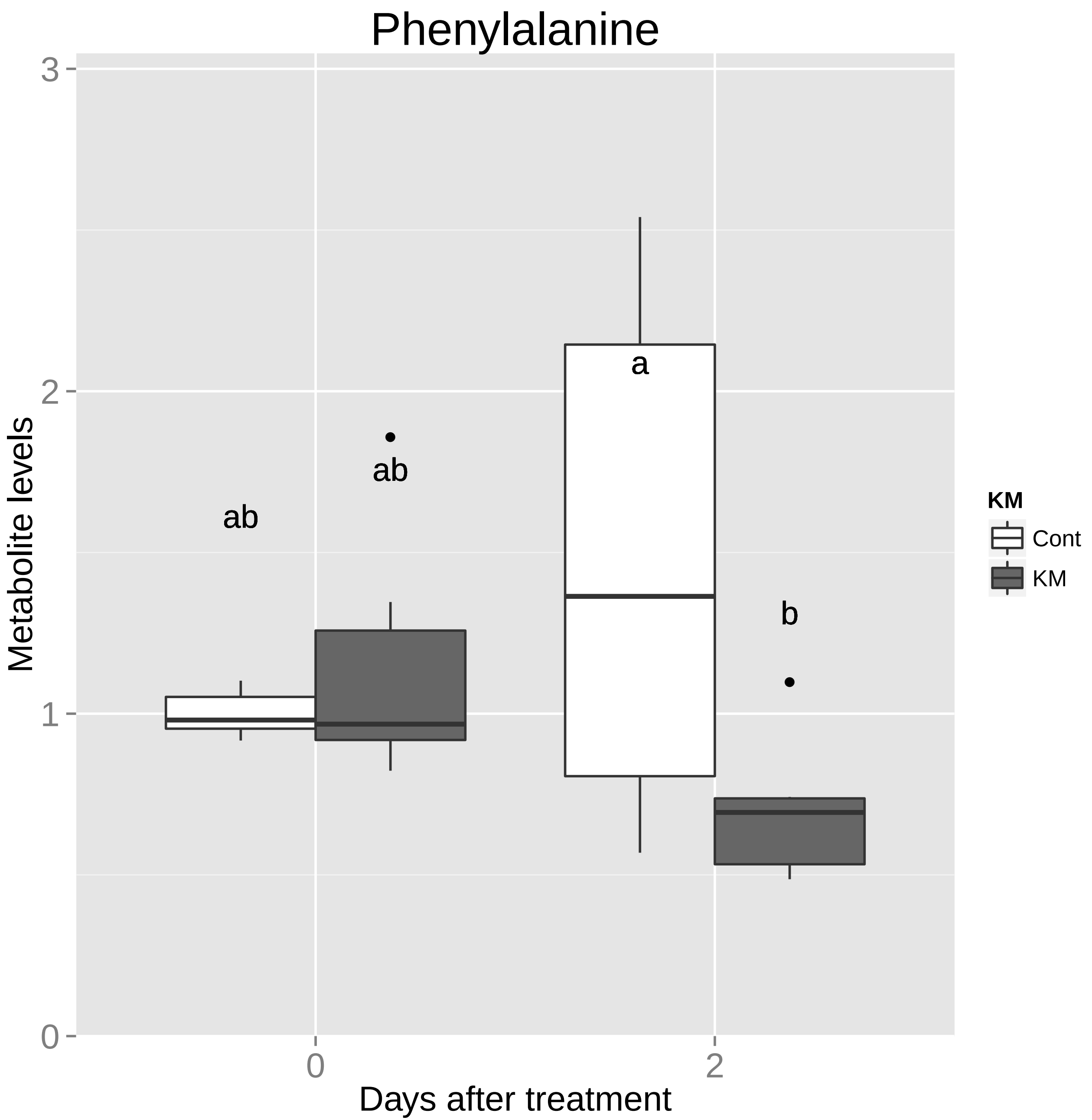

Phenylalanine
3
2
1
0
Metabolite levels
ab
ab
a
b
0
2
Days after treatment
KM
Cont
KM

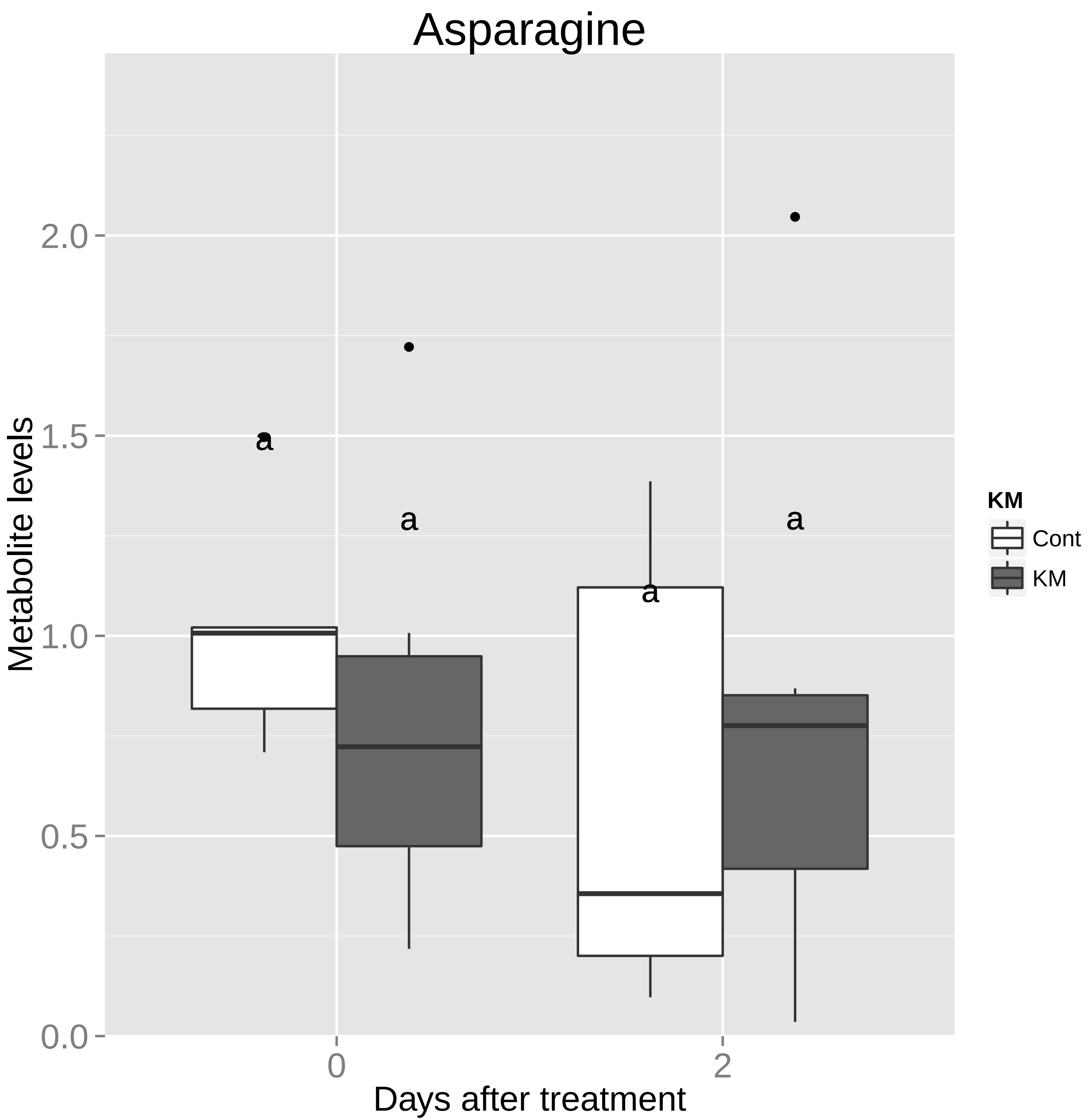

Asparagine
Metabolite levels
2.0
1.5
1.0
0.5
0.0
0
2
Days after treatment
a
a
a
a
KM
Cont
KM

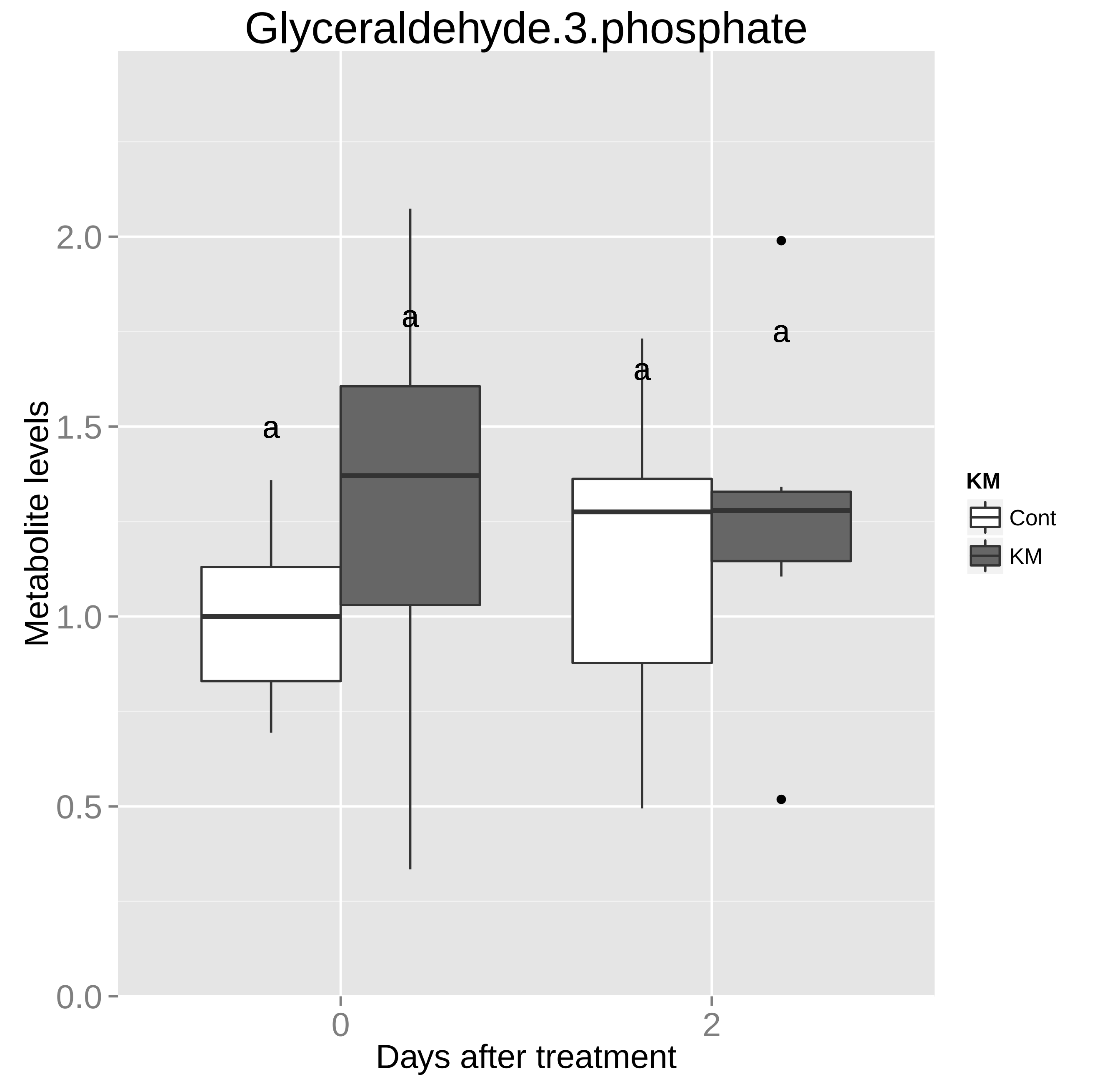

Glyceraldehyde.3.phosphate
Metabolite levels
0.0
0.5
1.0
1.5
2.0
a
a
a
a
0
2
Days after treatment
KM
Cont
KM

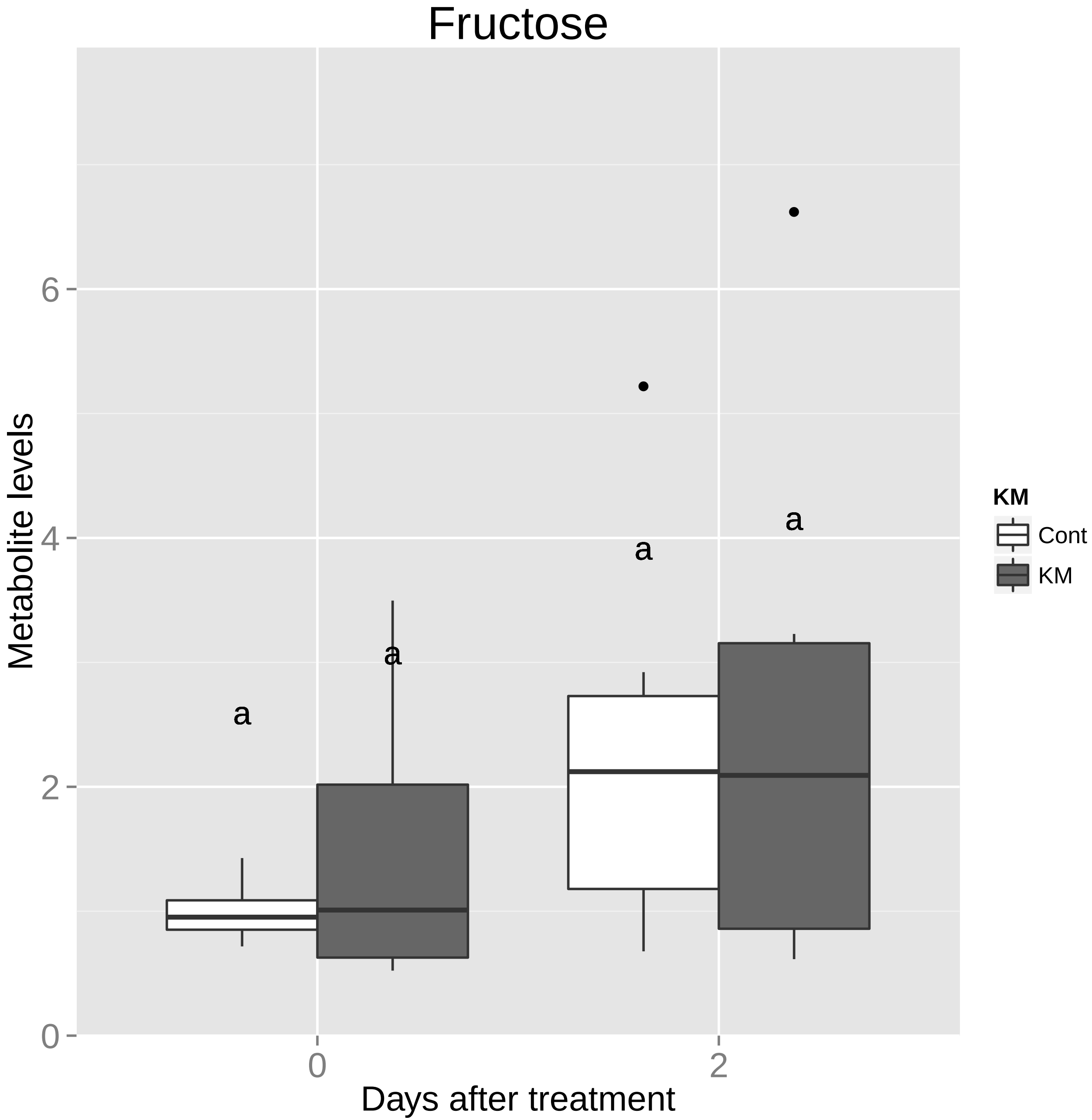

Fructose
Metabolite levels
Days after treatment
0
2
4
6
0
2
a
a
a
a
KM
Cont
KM

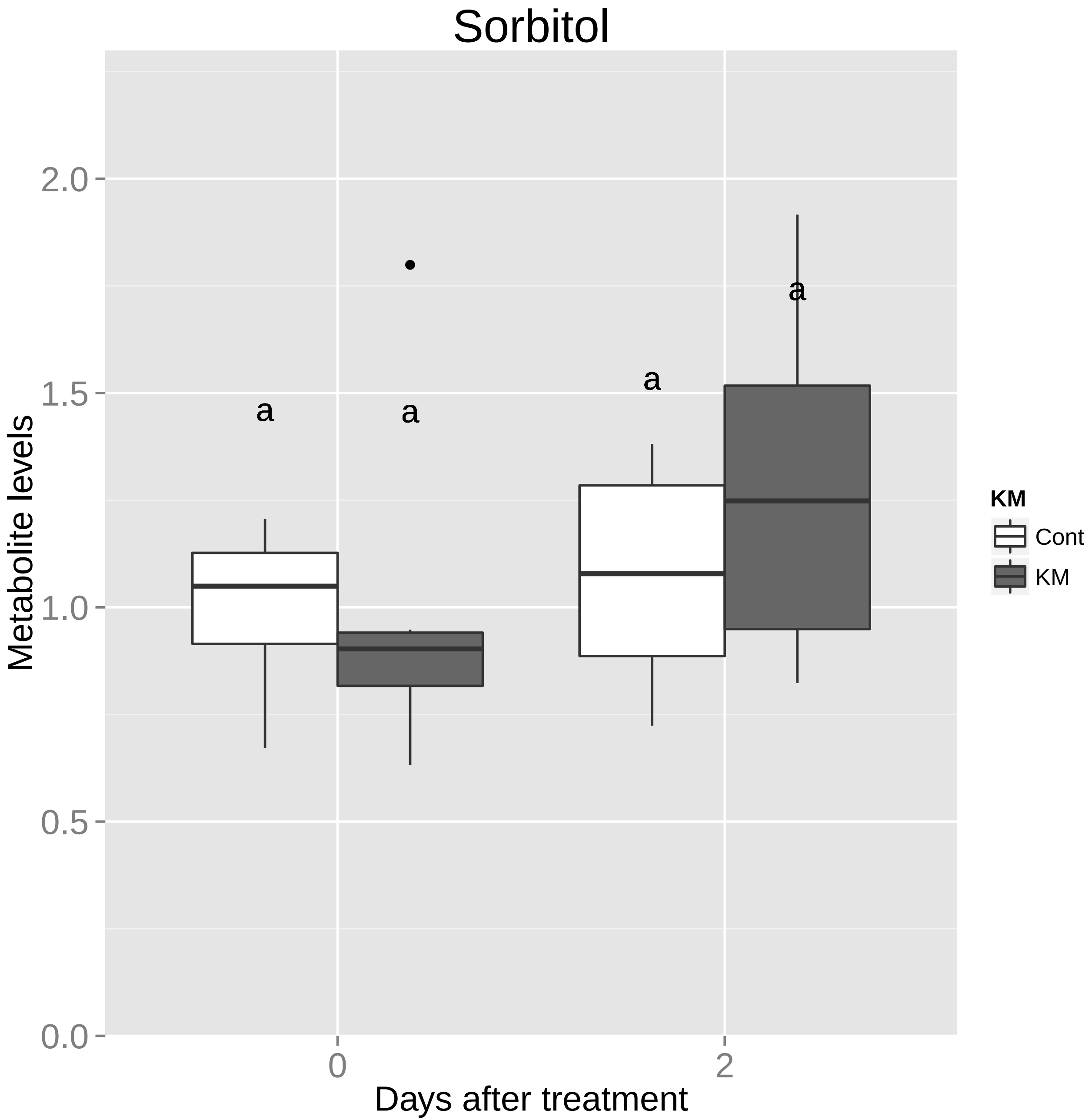

Sorbitol
2.0
1.5
1.0
0.5
0.0
Metabolite levels
a
a
a
a
0
2
Days after treatment
KM
Cont
KM

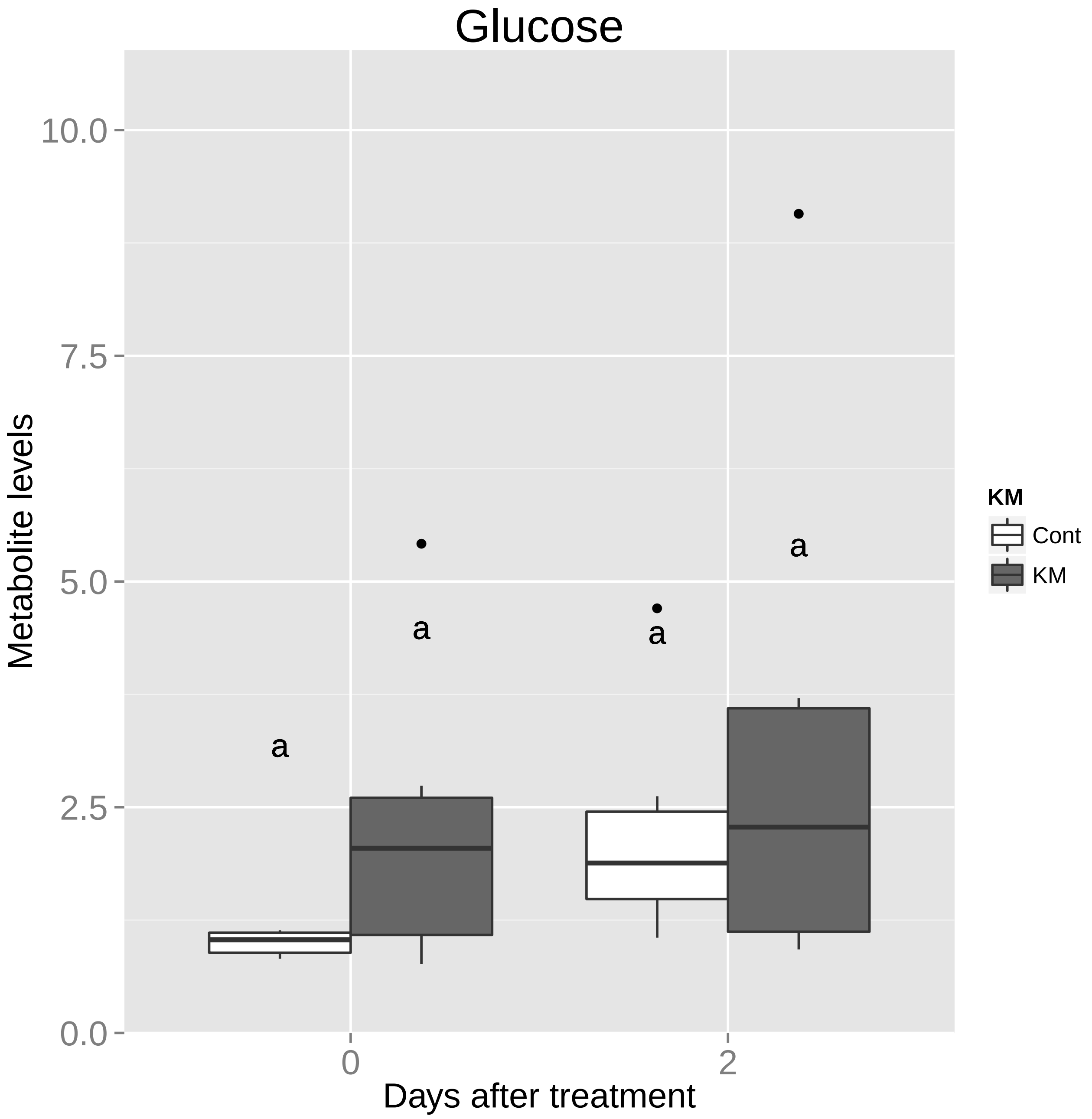

Glucose
10.0
7.5
5.0
2.5
0.0
Metabolite levels
a
a
a
a
0
2
Days after treatment
KM
Cont
KM

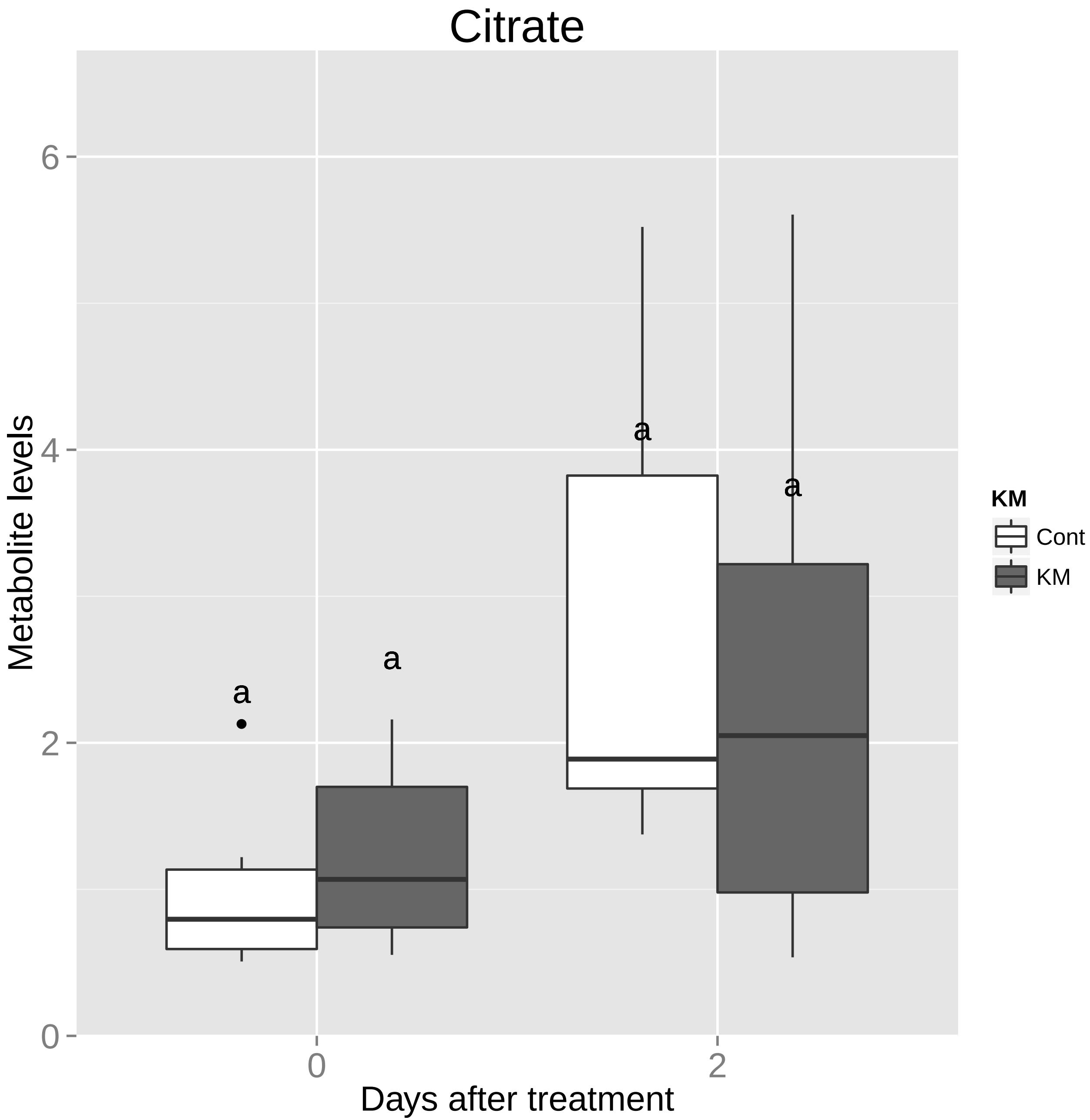

Citrate
Metabolite levels
Days after treatment
0
2
4
6
0
2
a
a
a
a
KM
Cont
KM

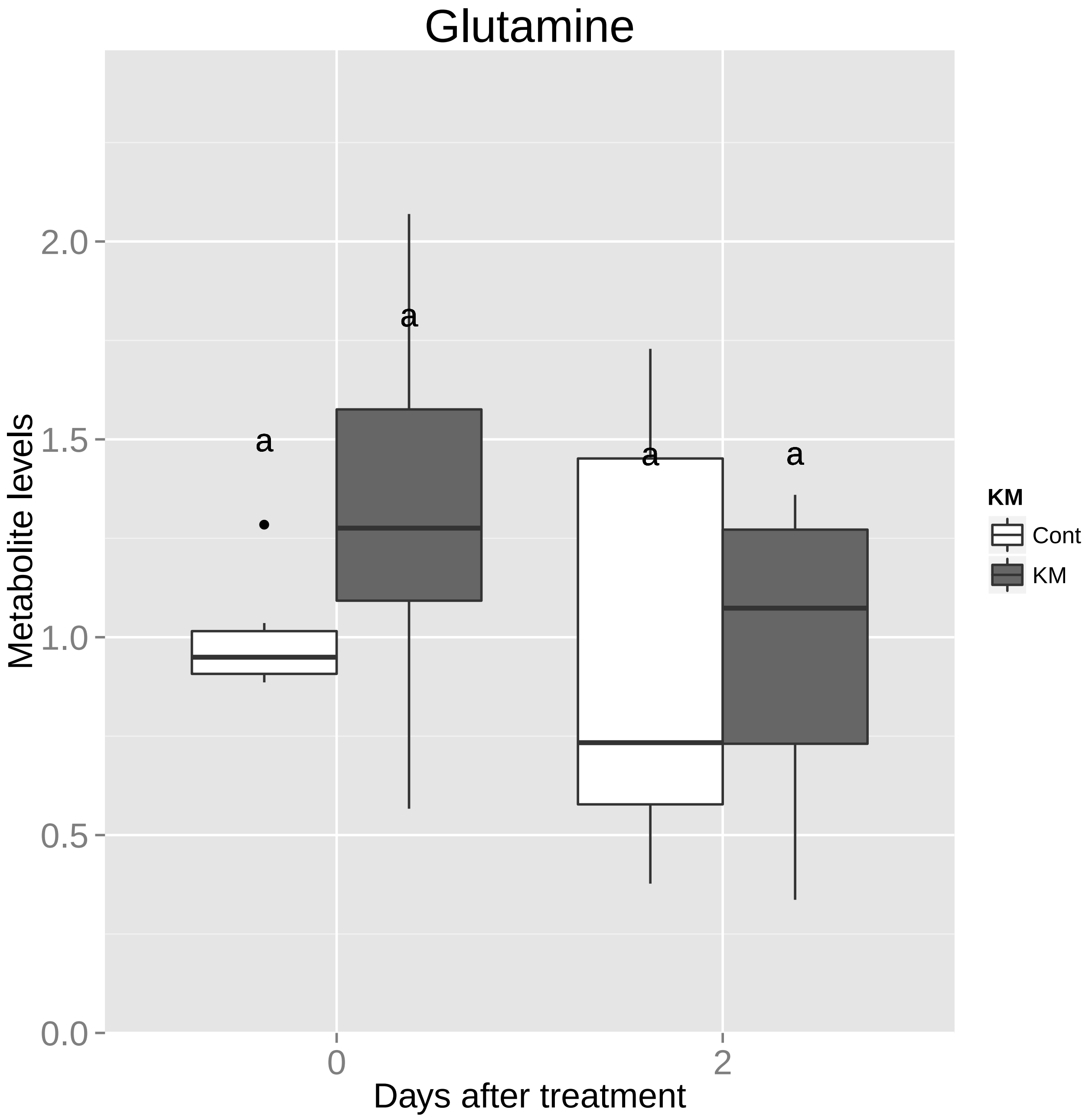

Glutamine
Metabolite levels
Days after treatment
0.0
0.5
1.0
1.5
2.0
0
2
a
a
a
a
KM
Cont
KM

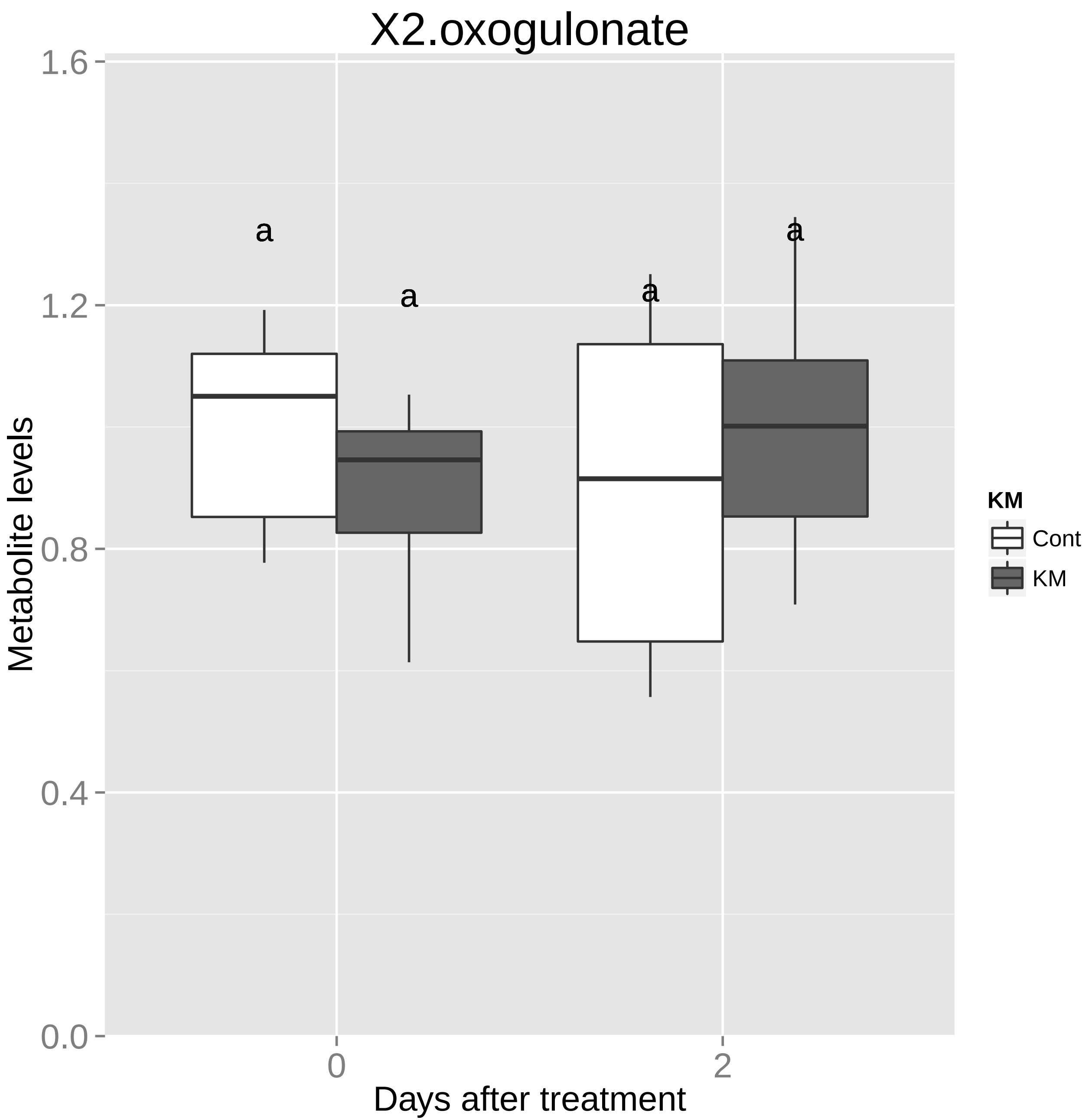

X2.oxogulonate
Metabolite levels
Days after treatment
0.0
0.4
0.8
1.2
1.6
0
2
a
a
a
a
KM
Cont
KM

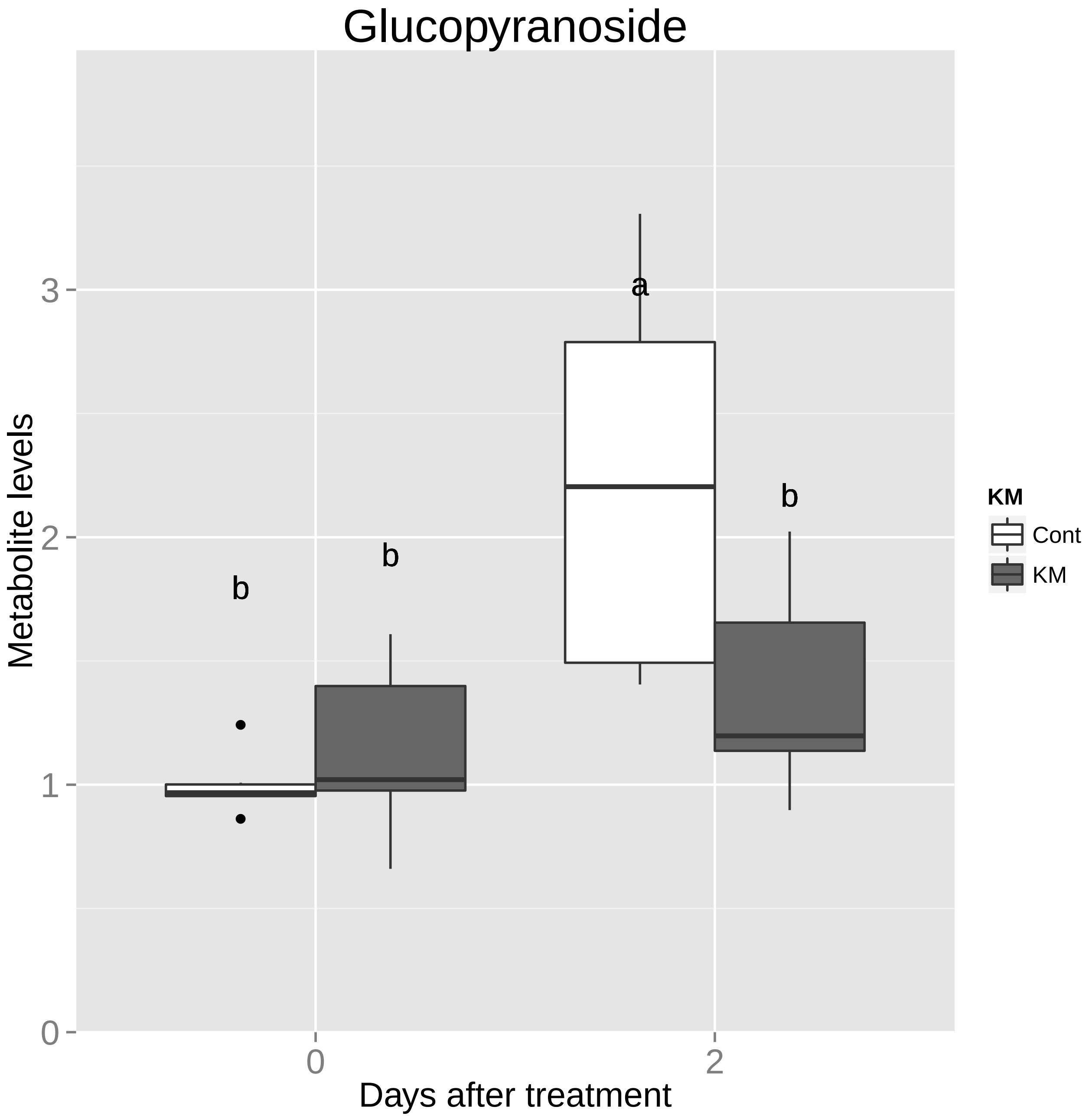

Glucopyranoside
Metabolite levels
Days after treatment
0
1
2
3
0
2
a
b
b
b
KM
Cont
KM

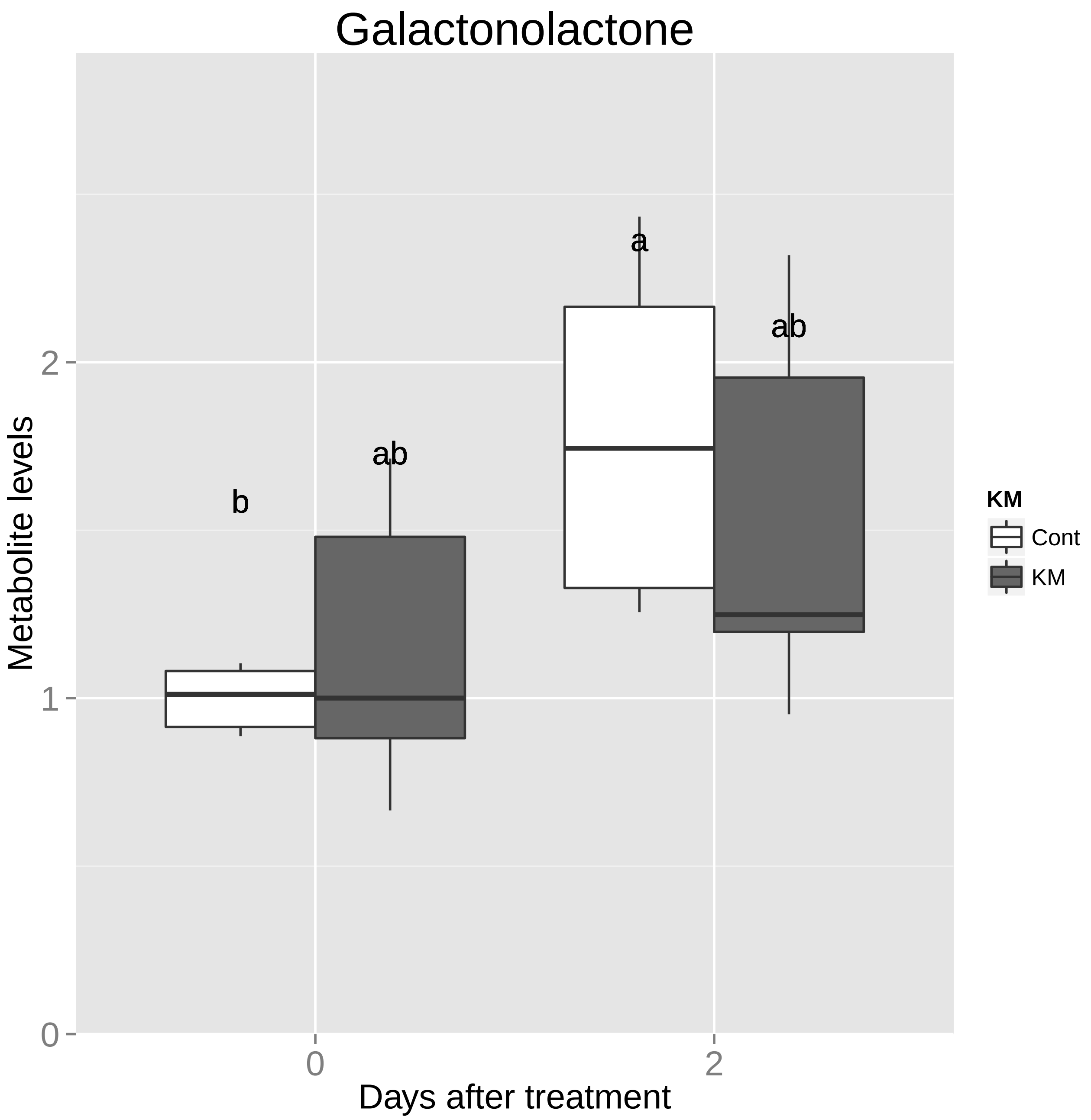

Galactonolactone
Metabolite levels
Days after treatment
0
1
2
0
2
b
ab
a
ab
KM
Cont
KM

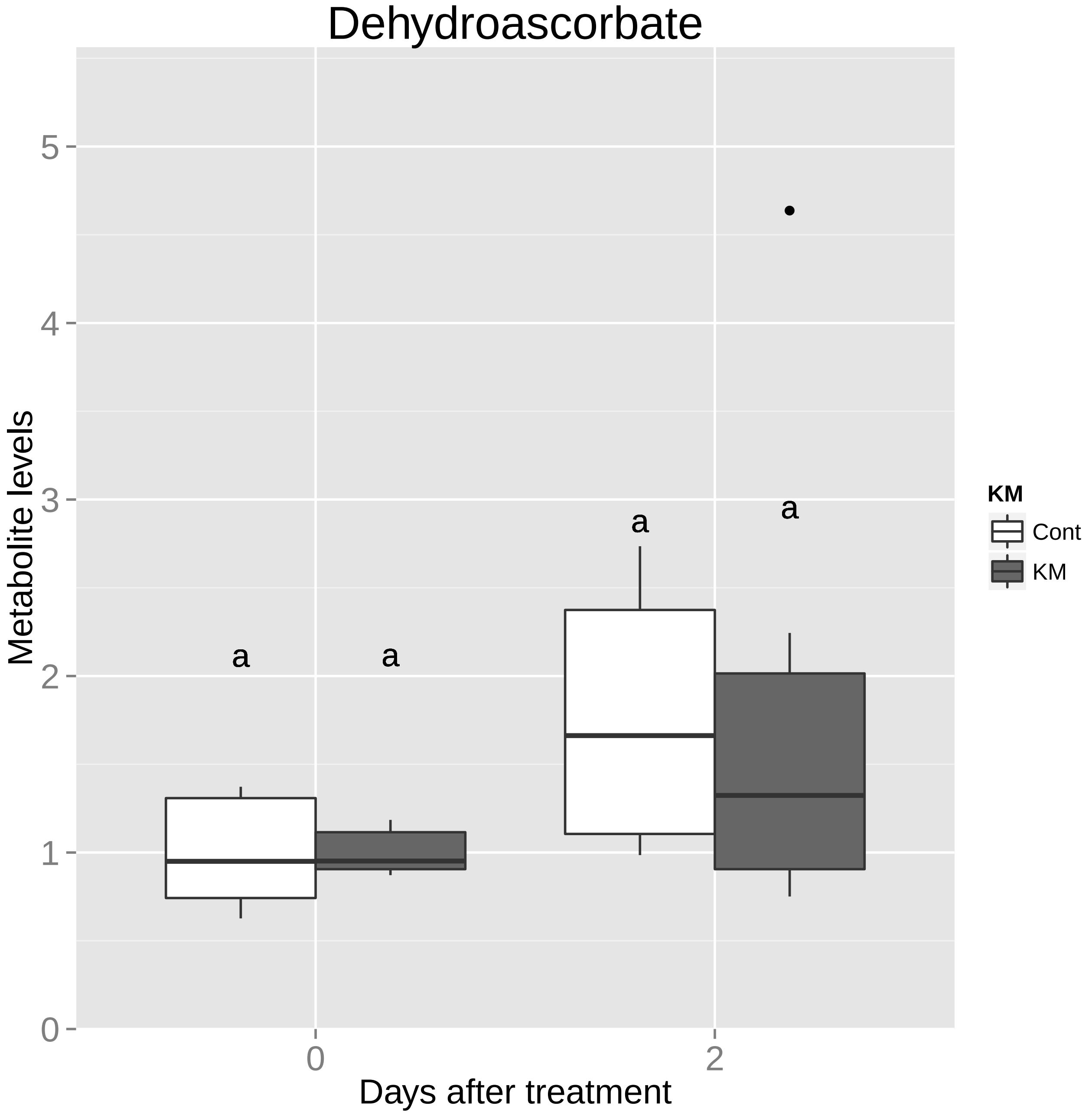

Dehydroascorbate
Metabolite levels
Days after treatment
0
1
2
3
4
5
0
2
a
a
a
a
KM
Cont
KM

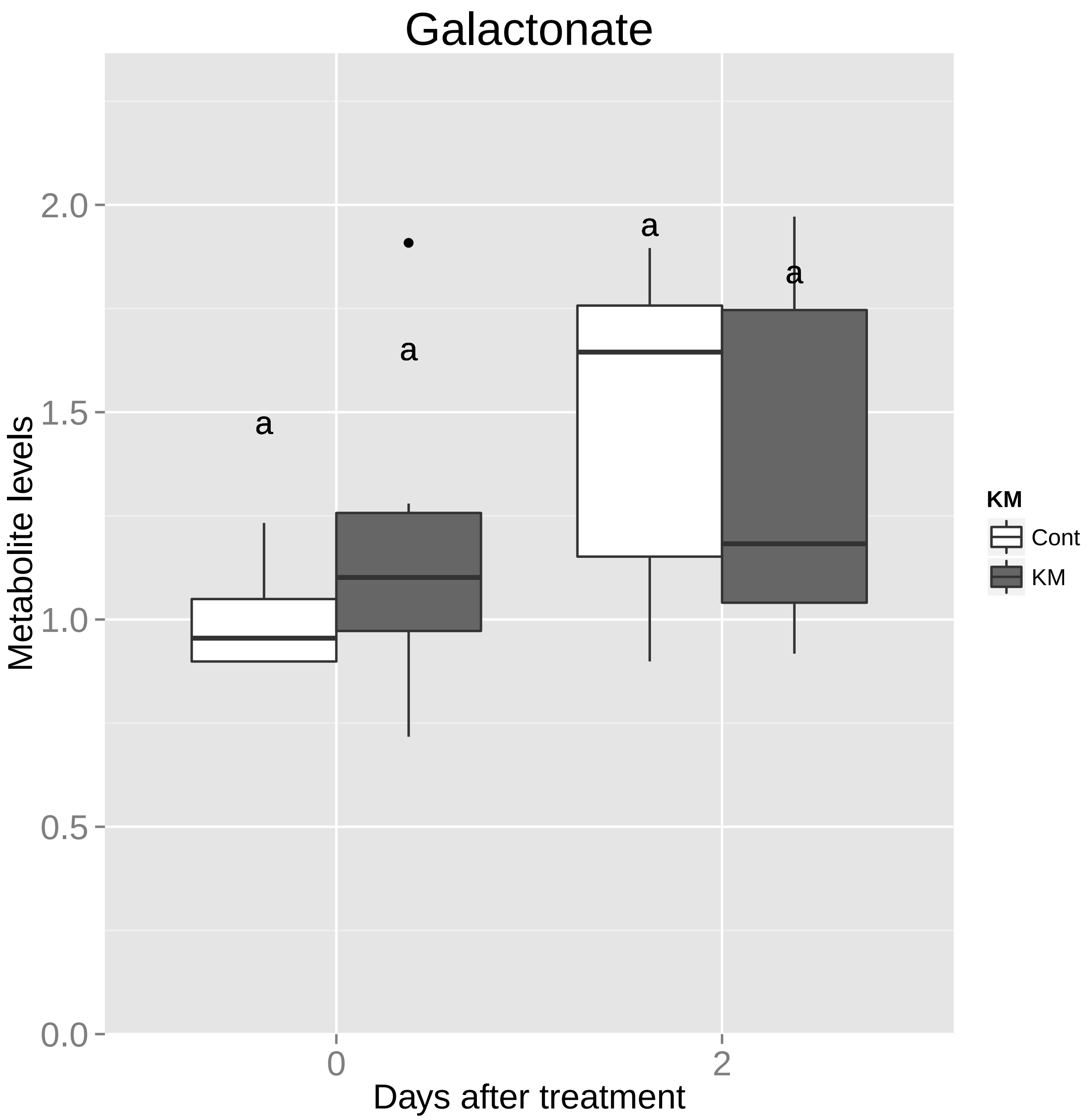
Galactonate
Metabolite levels
2.0
1.5
1.0
0.5
0.0
a
a
a
a
0
2
Days after treatment
KM
Cont
KM

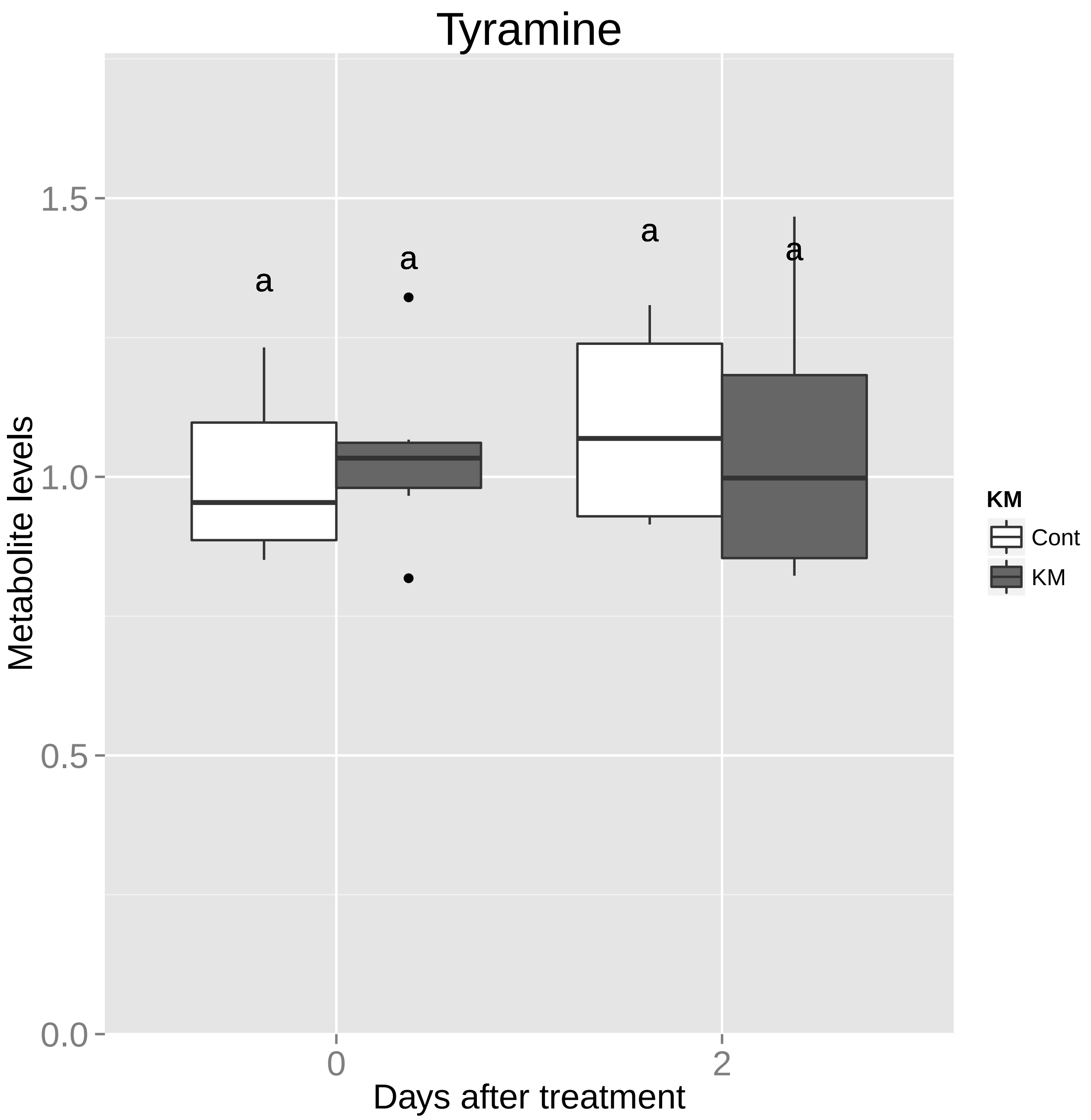

Tyramine
Metabolite levels
0.0
0.5
1.0
1.5
a
a
a
a
0
2
Days after treatment
KM
Cont
KM

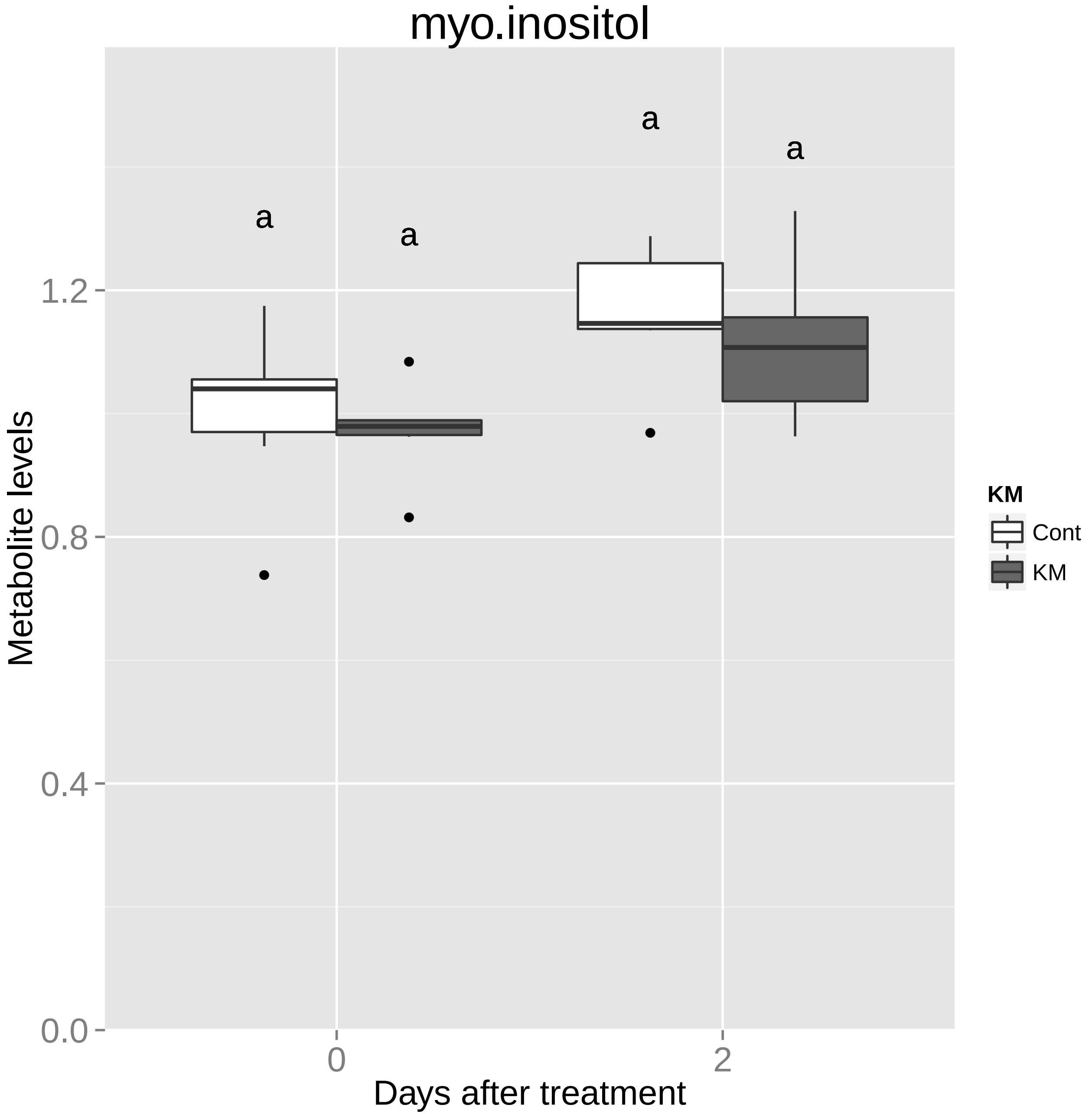

myo.inositol
a
a
a
a
Metabolite levels
1.2
0.8
0.4
0.0
0
2
Days after treatment
KM
Cont
KM

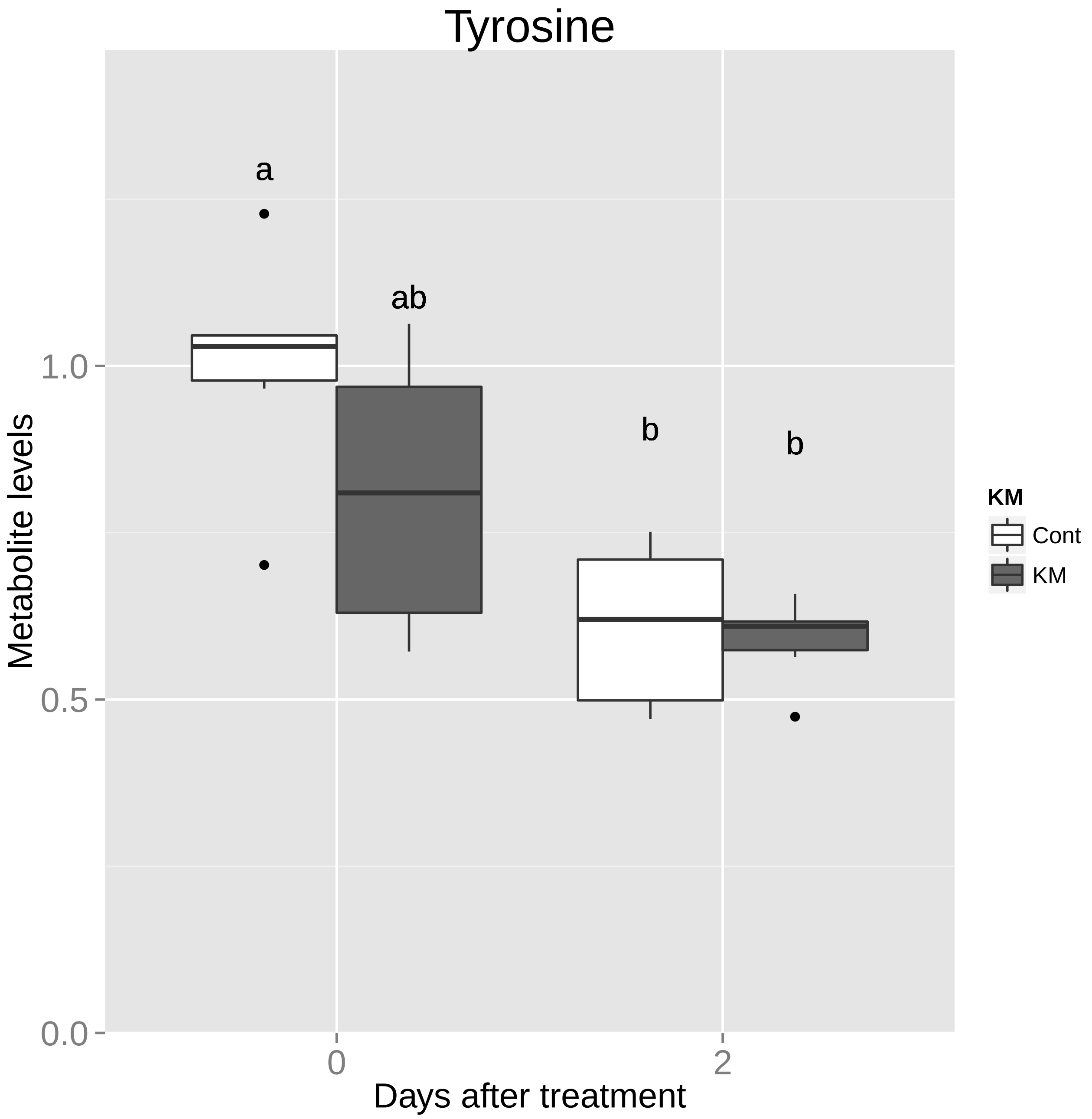
Tyrosine
a
ab
b
b
1.0
0.5
0.0
0
2
Metabolite levels
Days after treatment
KM
Cont
KM

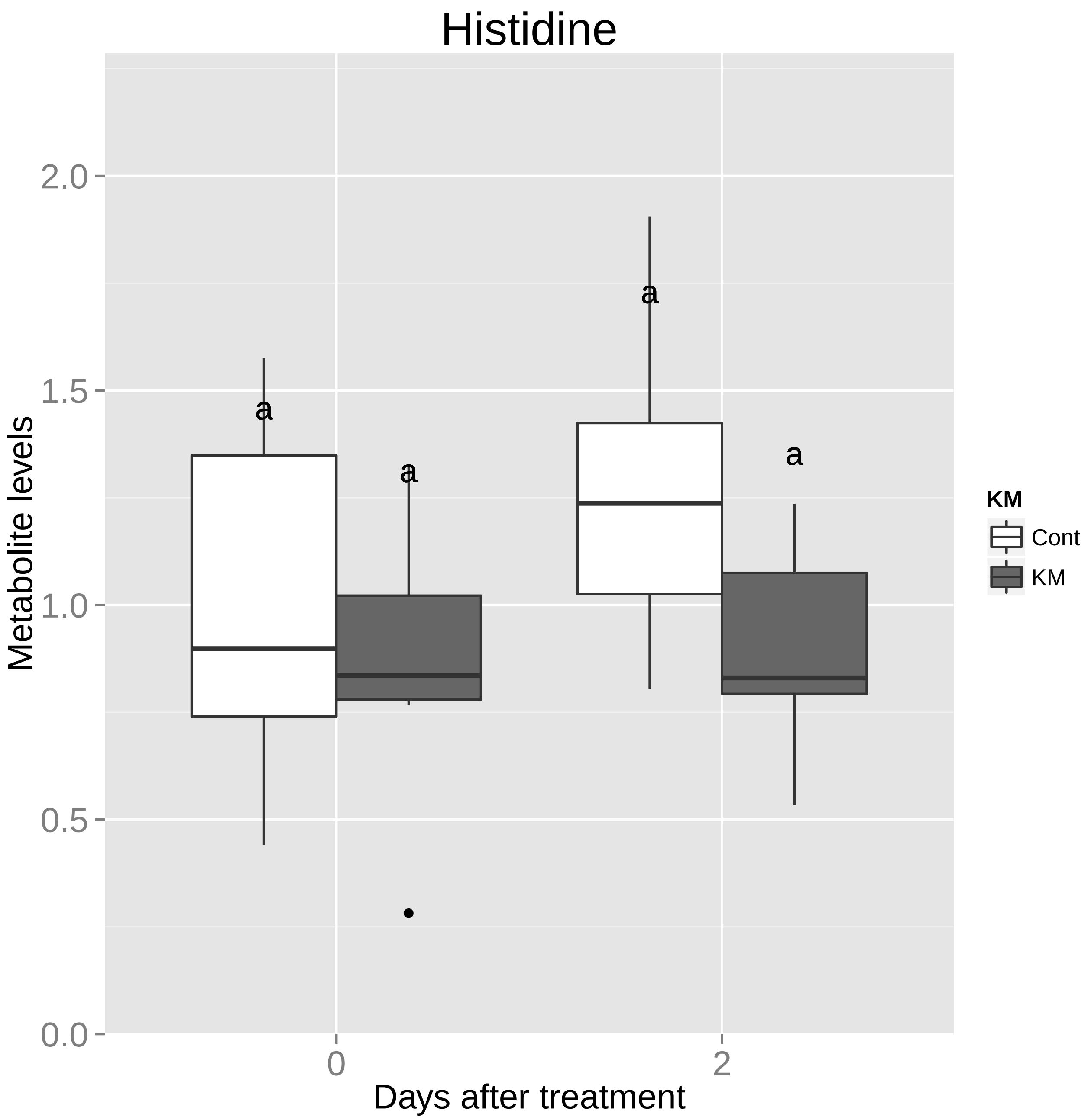

Histidine
Metabolite levels
2.0
1.5
1.0
0.5
0.0
0
2
Days after treatment
a
a
a
a
KM
Cont
KM

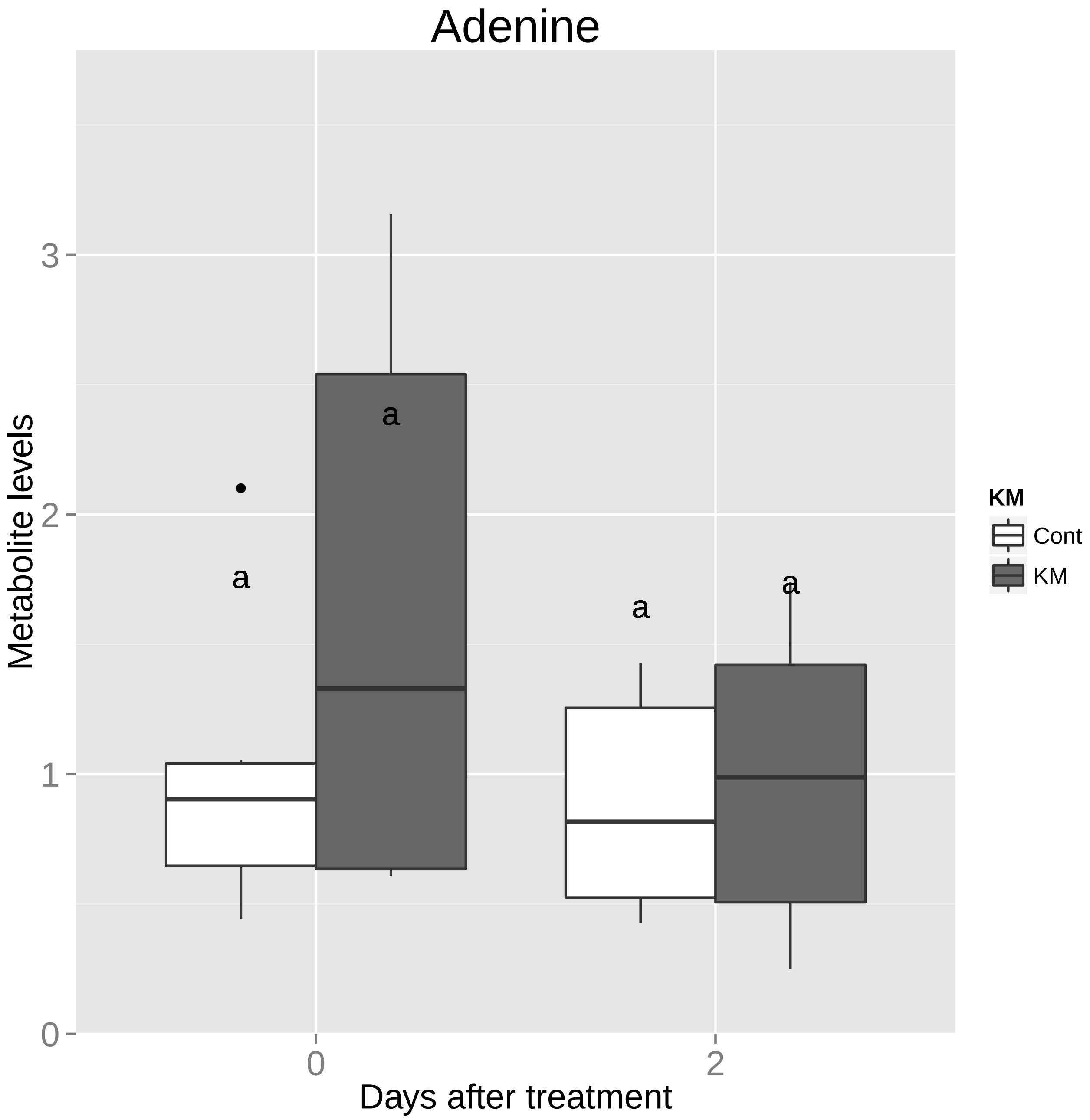

Adenine
Metabolite levels
Days after treatment
0
1
2
3
0
2
a
a
a
a
KM
Cont
KM

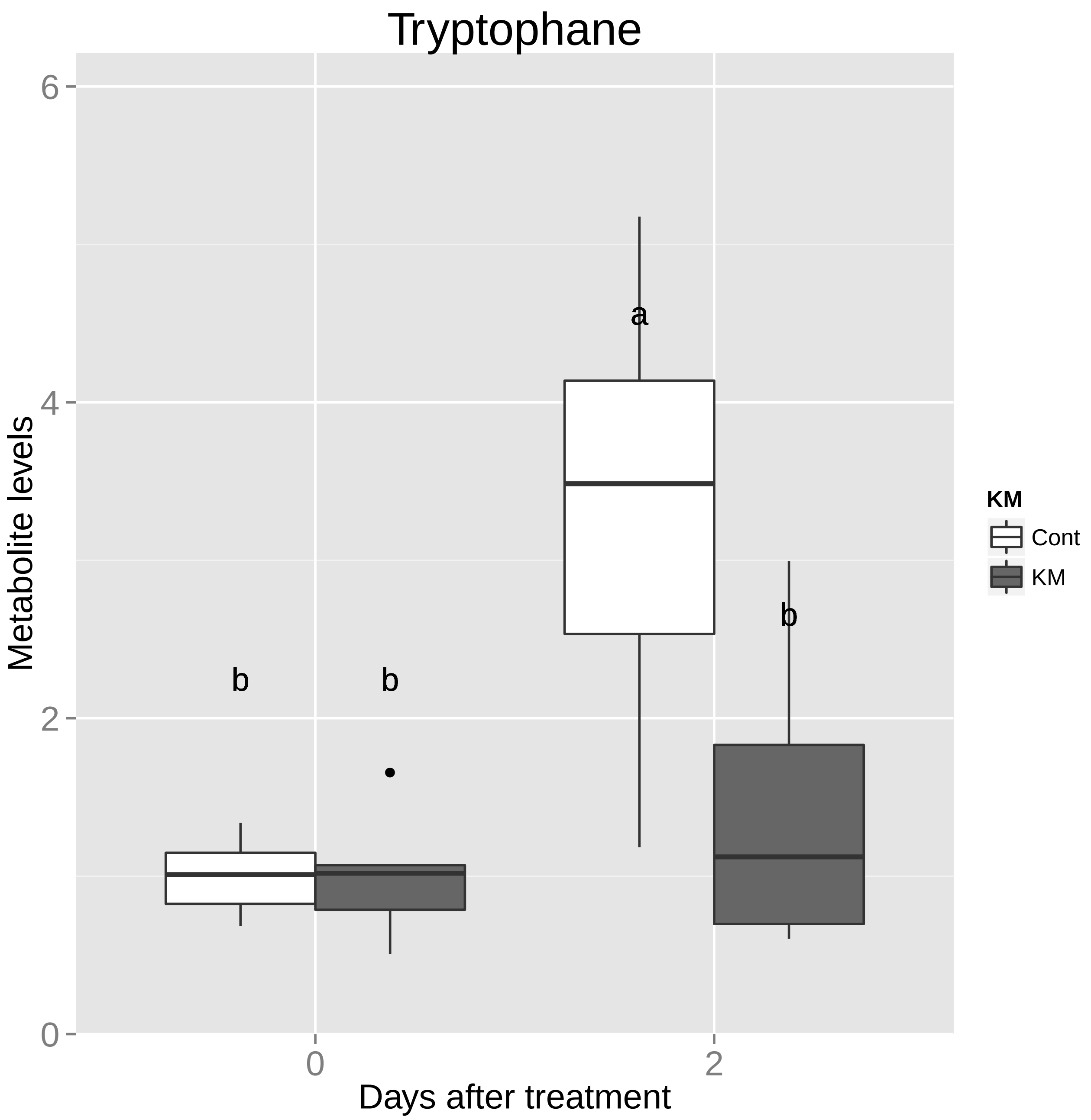
Tryptophane
Metabolite levels
Days after treatment
0
2
4
6
0
2
a
b
b
b
KM
Cont
KM

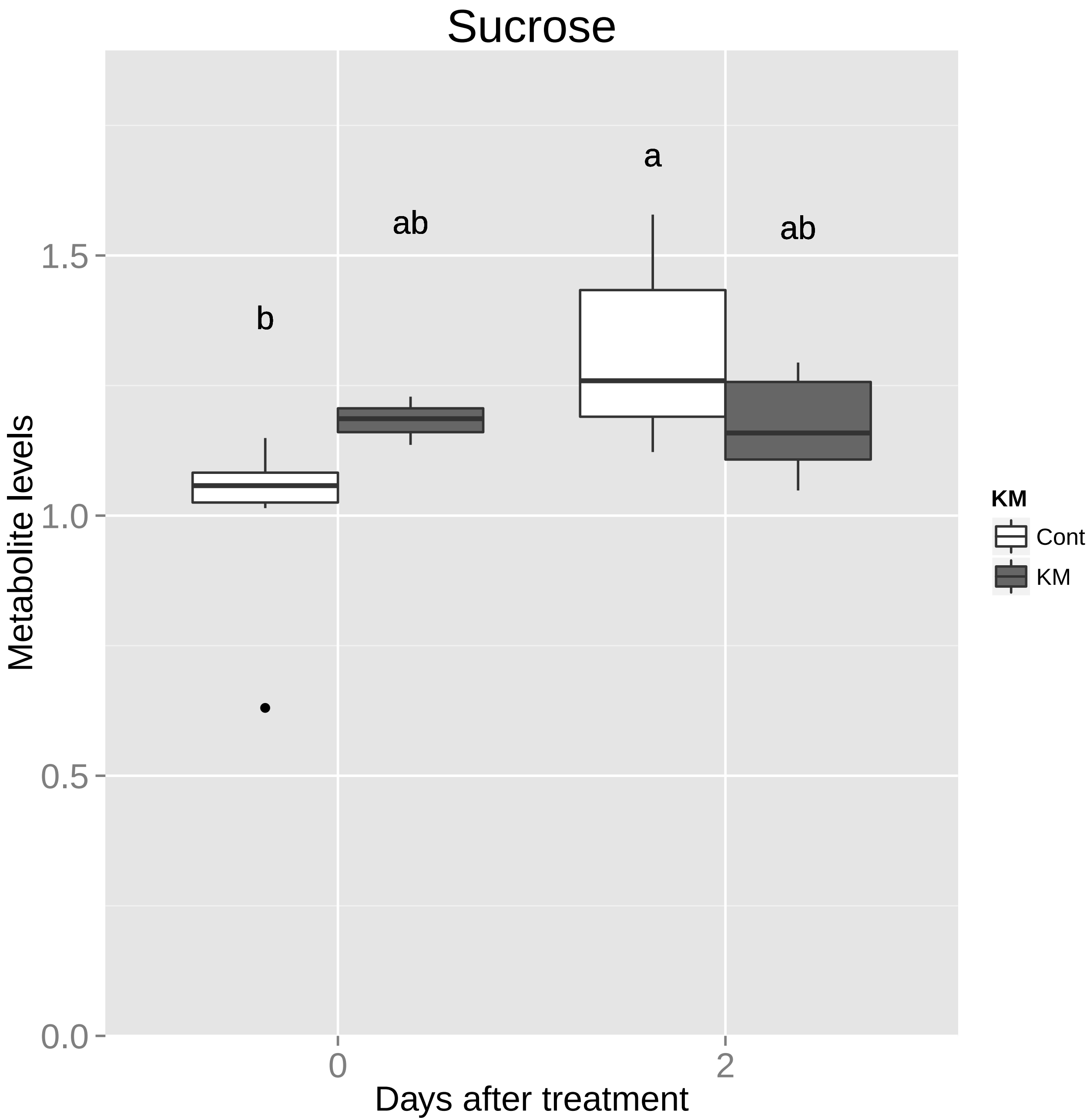
Sucrose
Metabolite levels
1.5
1.0
0.5
0.0
b
ab
a
ab
0
2
Days after treatment
KM
Cont
KM

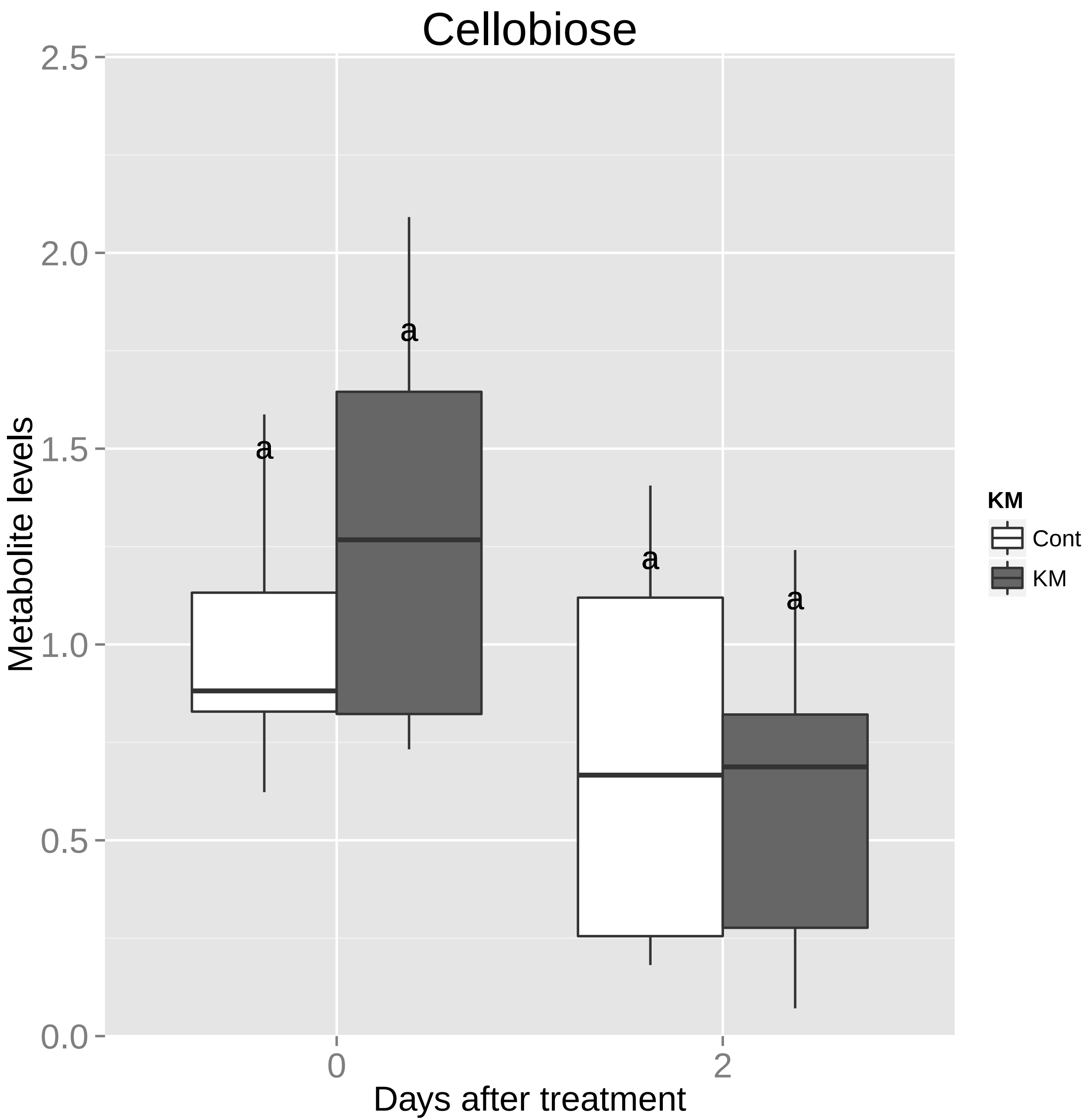

Cellobiose
2.5
2.0
1.5
1.0
0.5
0.0
Metabolite levels
a
a
a
a
0
2
Days after treatment
KM
Cont
KM

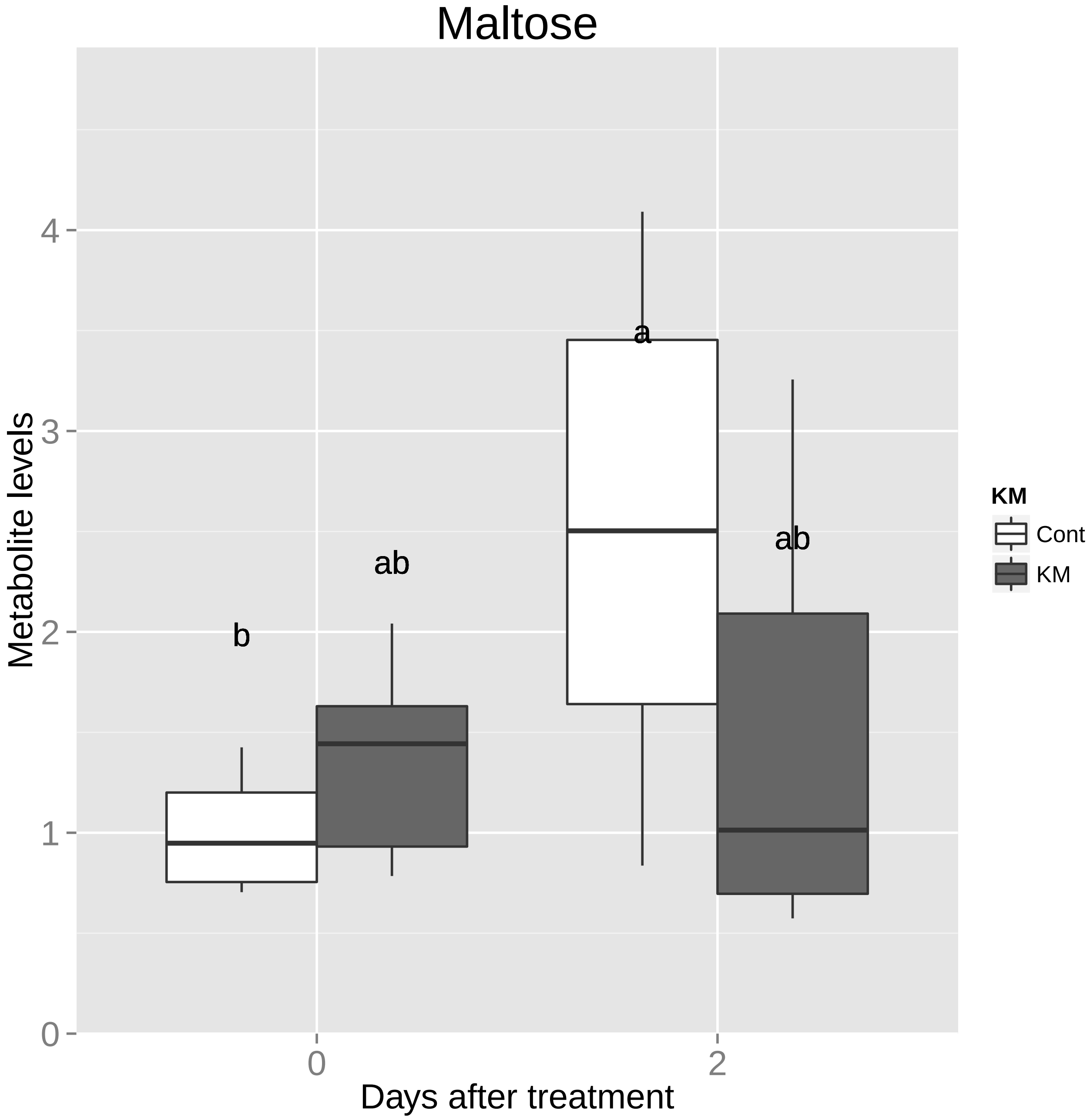
Maltose
Metabolite levels
Days after treatment
0
1
2
3
4
0
2
b
ab
a
ab
KM
Cont
KM

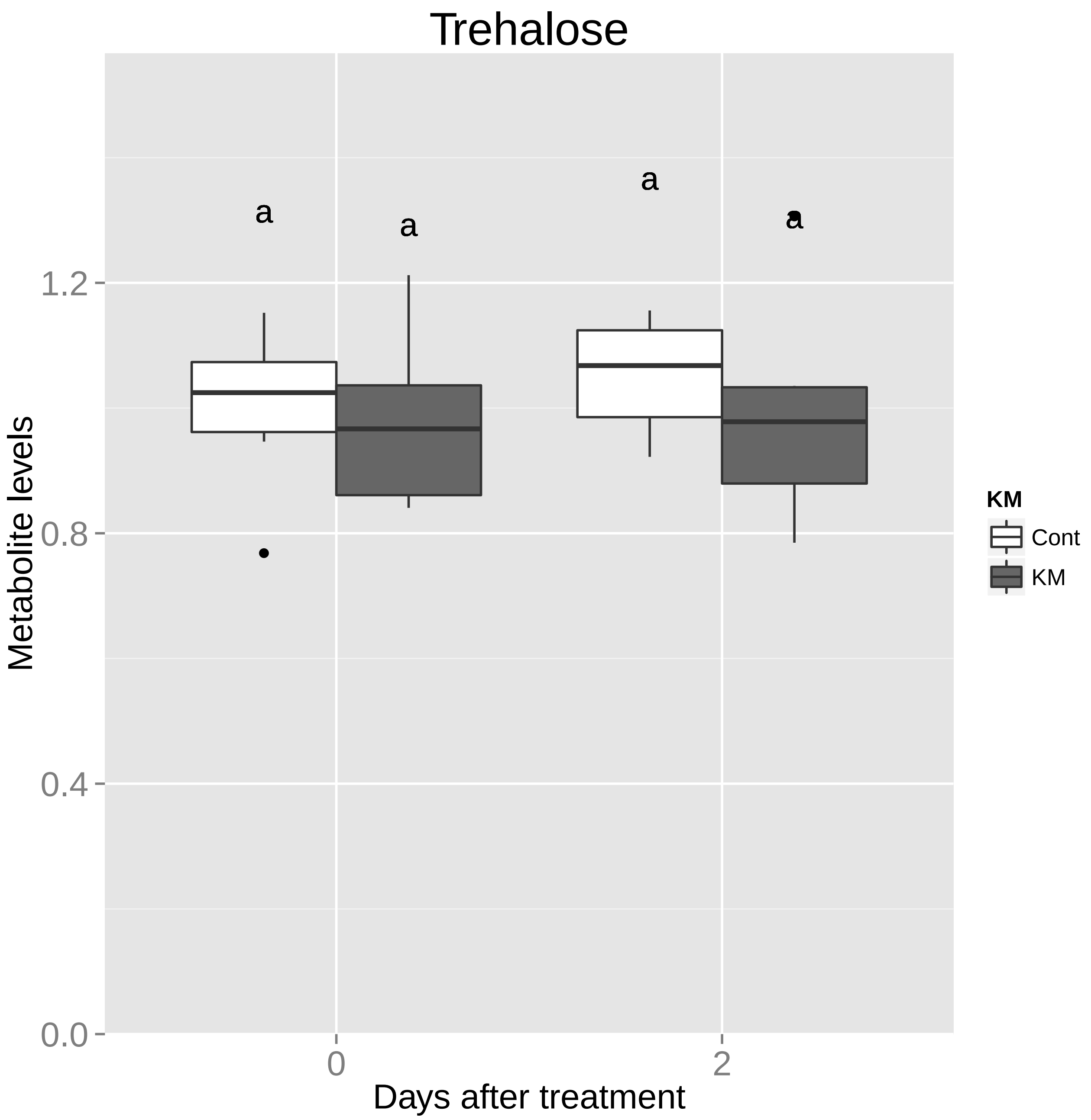

Trehalose
a
a
a
a
1.2
0.8
0.4
0.0
Metabolite levels
0
2
Days after treatment
KM
Cont
KM

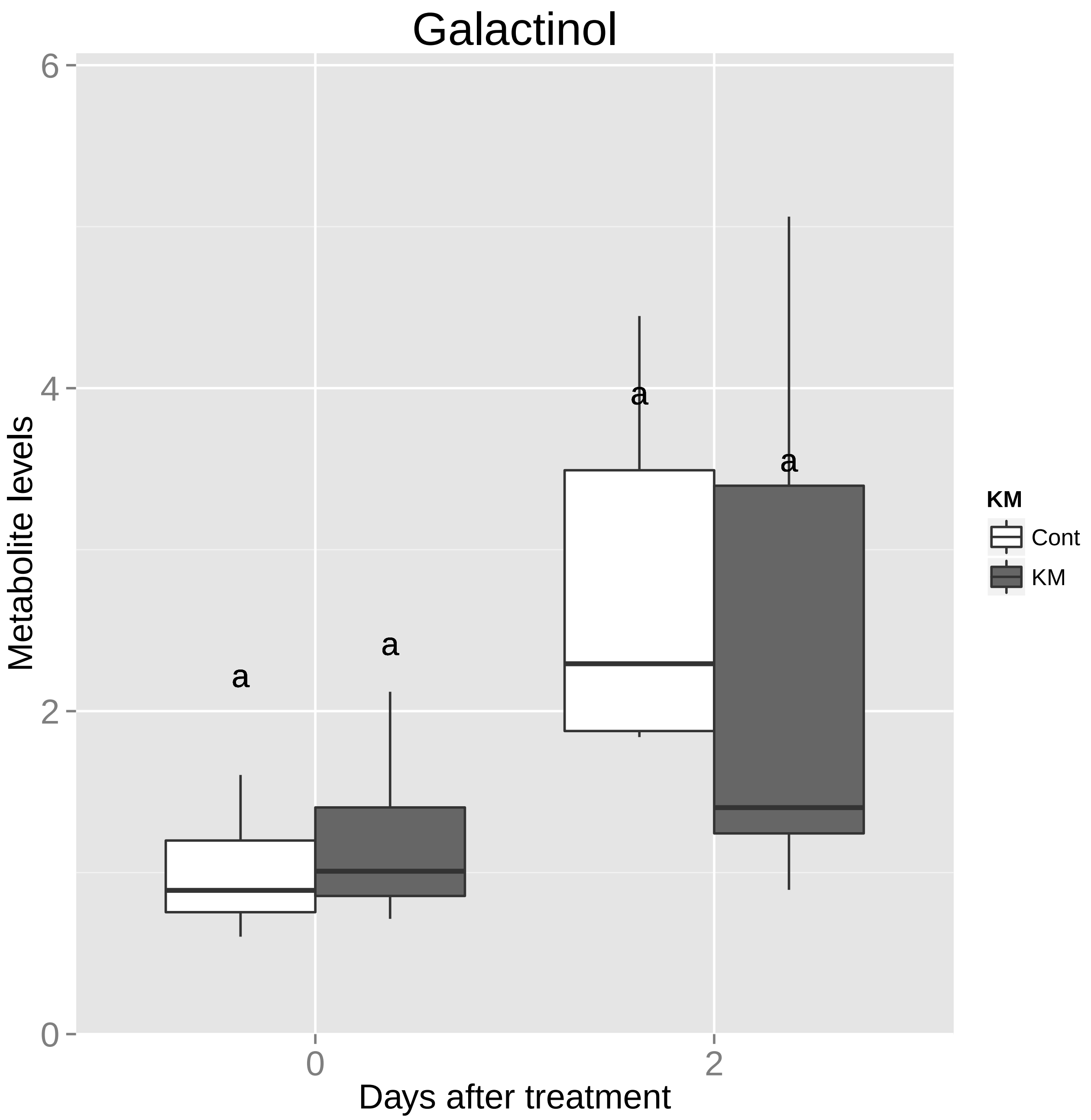
Galactinol
Metabolite levels
Days after treatment
0
2
4
6
0
2
a
a
a
a
KM
Cont
KM

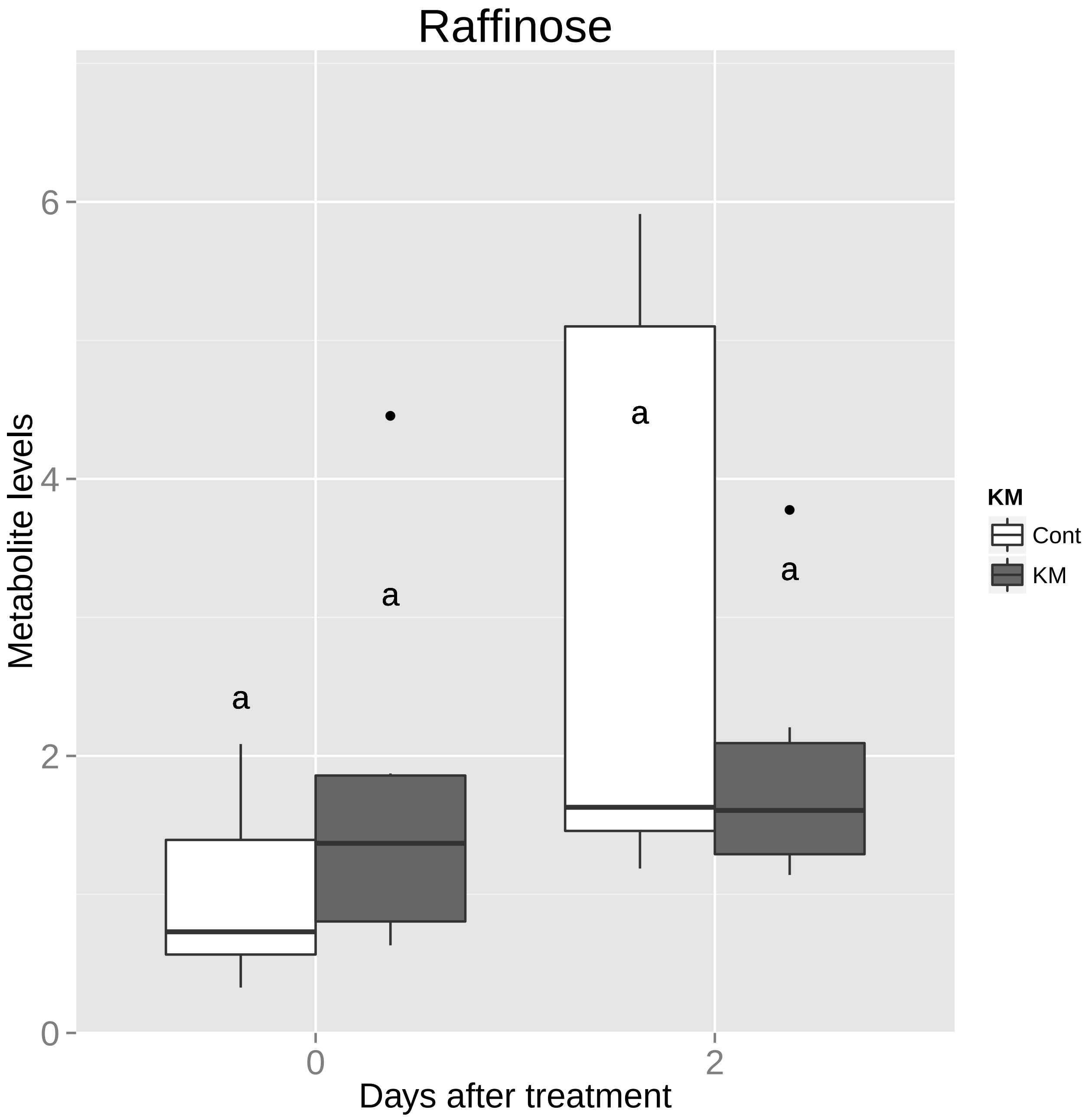
Raffinose
Metabolite levels
0
2
4
6
a
a
a
a
Days after treatment
KM
Cont
KM

Supplementary Figure S4C

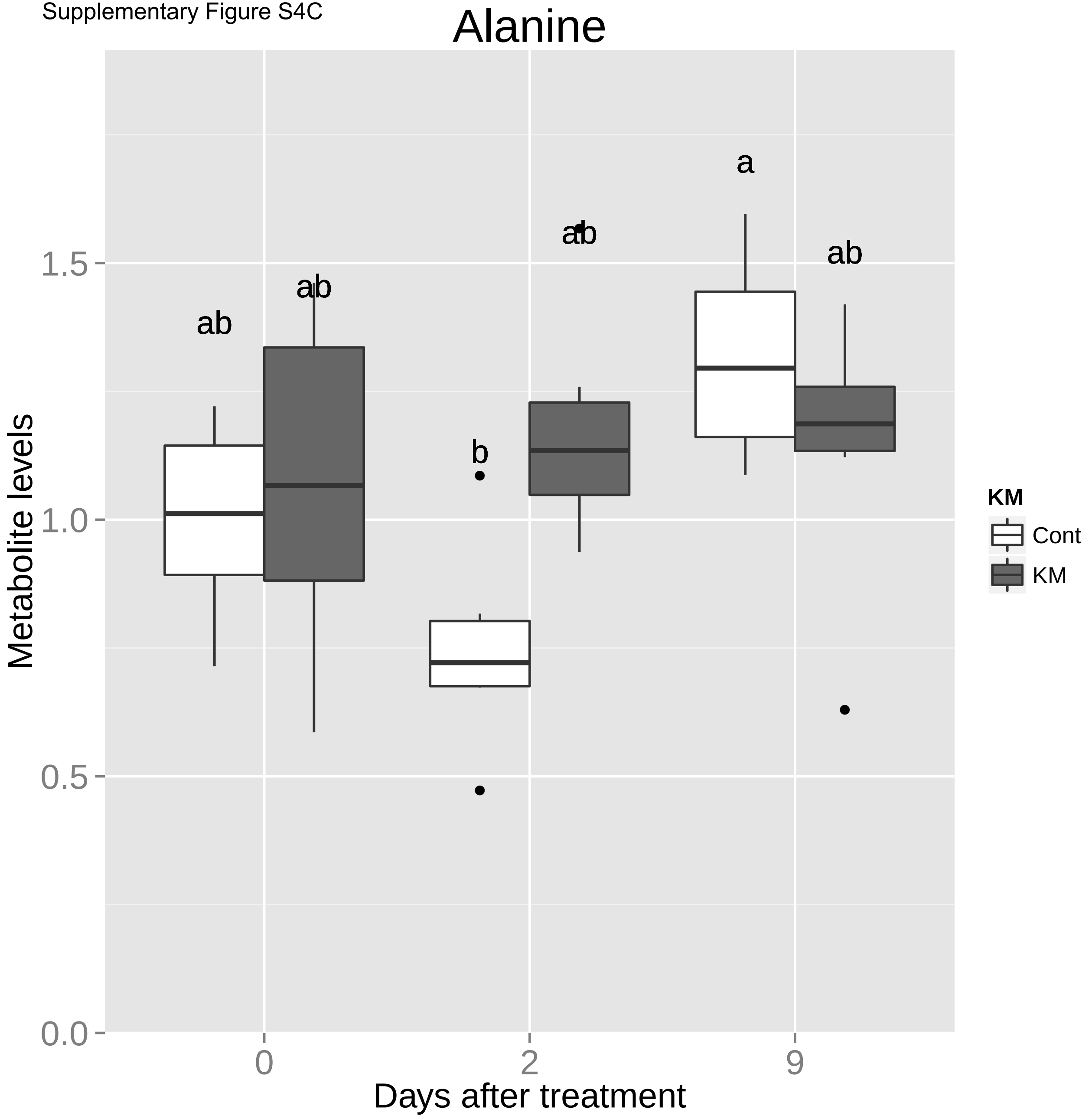

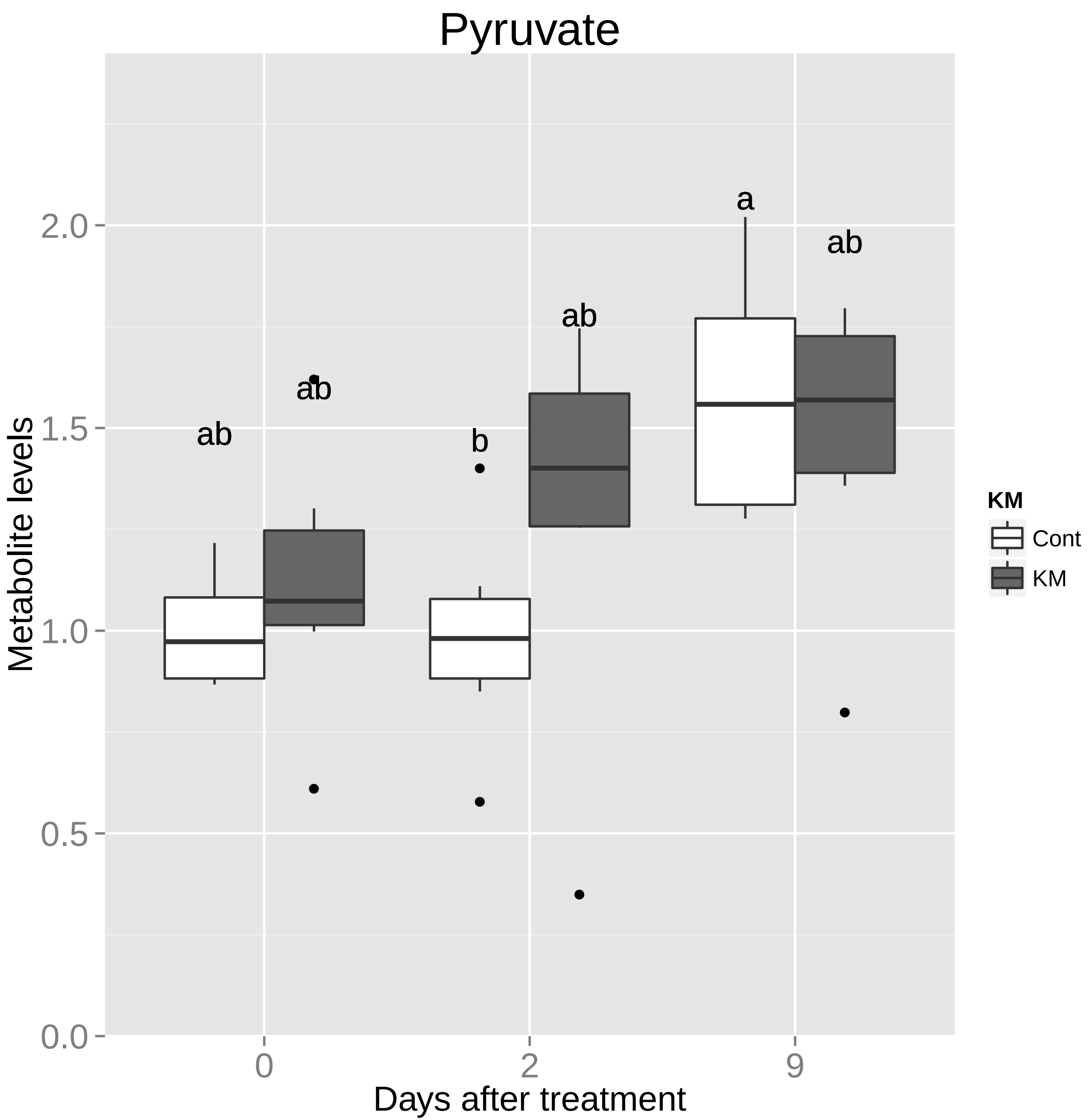
Pyruvate
Metabolite levels
2.0
1.5
1.0
0.5
0.0
ab
ab
b
ab
a
ab
0
2
9
Days after treatment
KM
Cont
KM

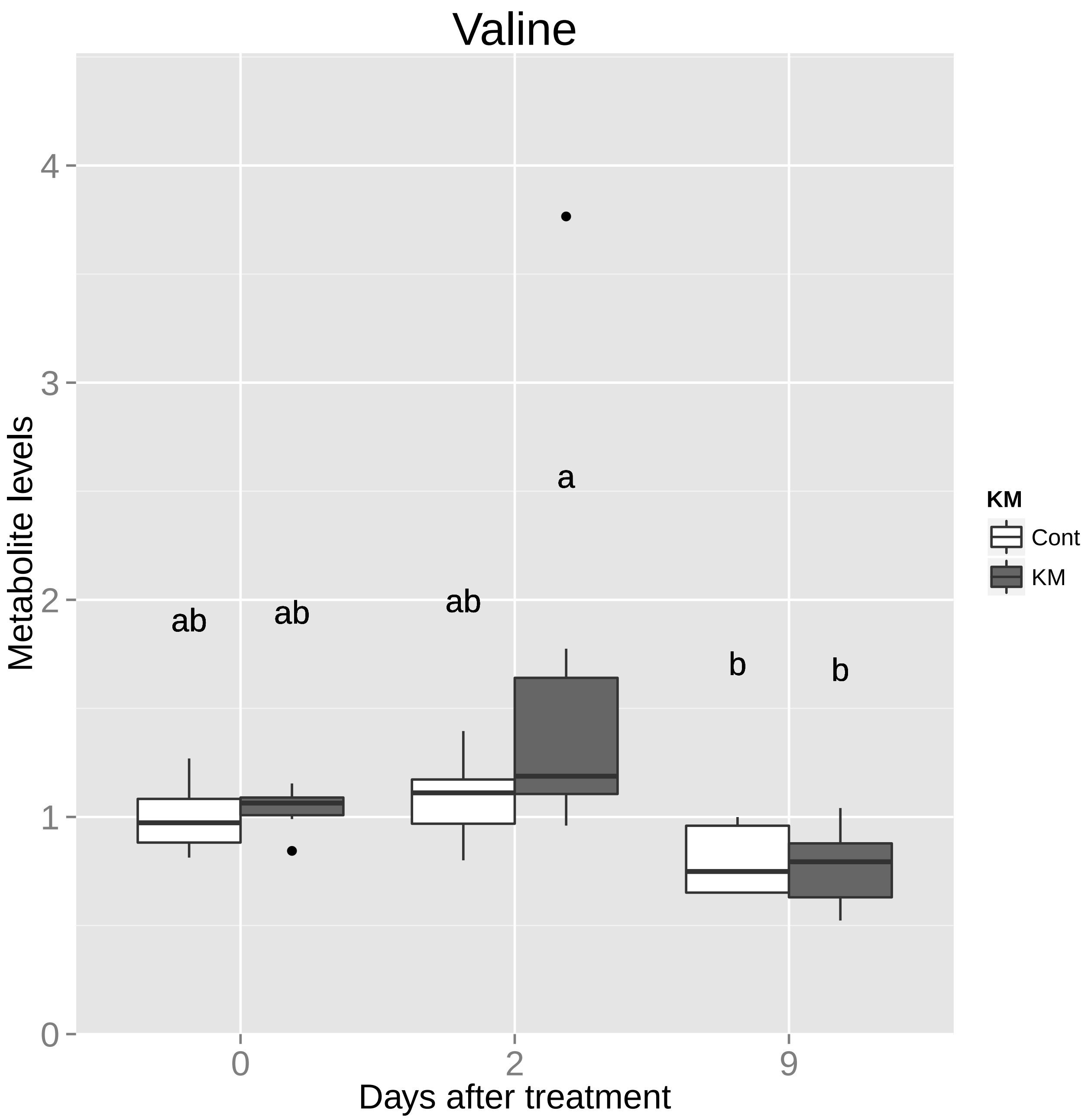
Valine
Metabolite levels
0
1
2
3
4
ab
ab
ab
a
b
b
0
2
9
Days after treatment
KM
Cont
KM

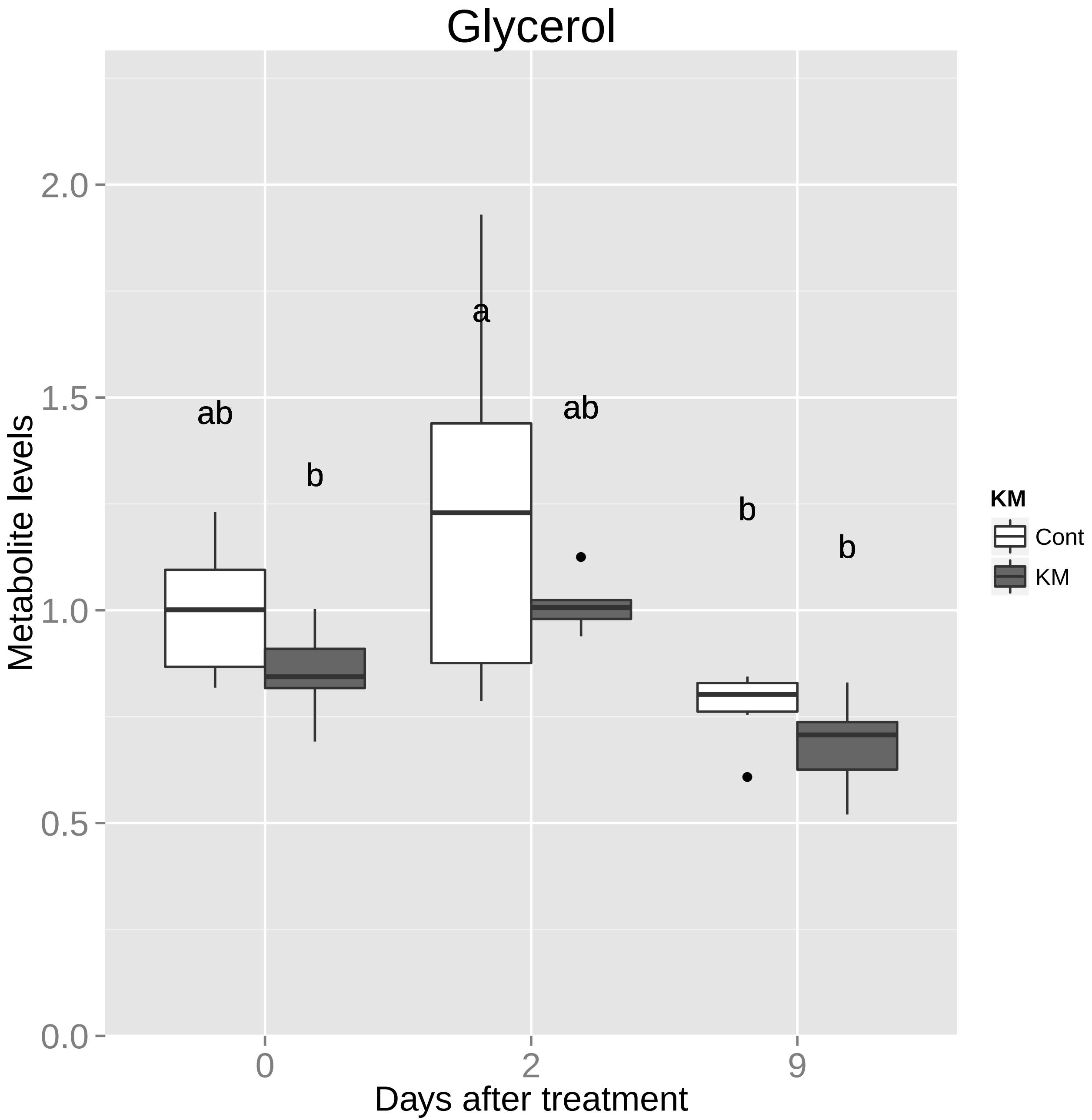

Glycerol
Metabolite levels
Days after treatment
0.0
0.5
1.0
1.5
2.0
0
2
9
ab
b
a
ab
b
b
KM
Cont
KM

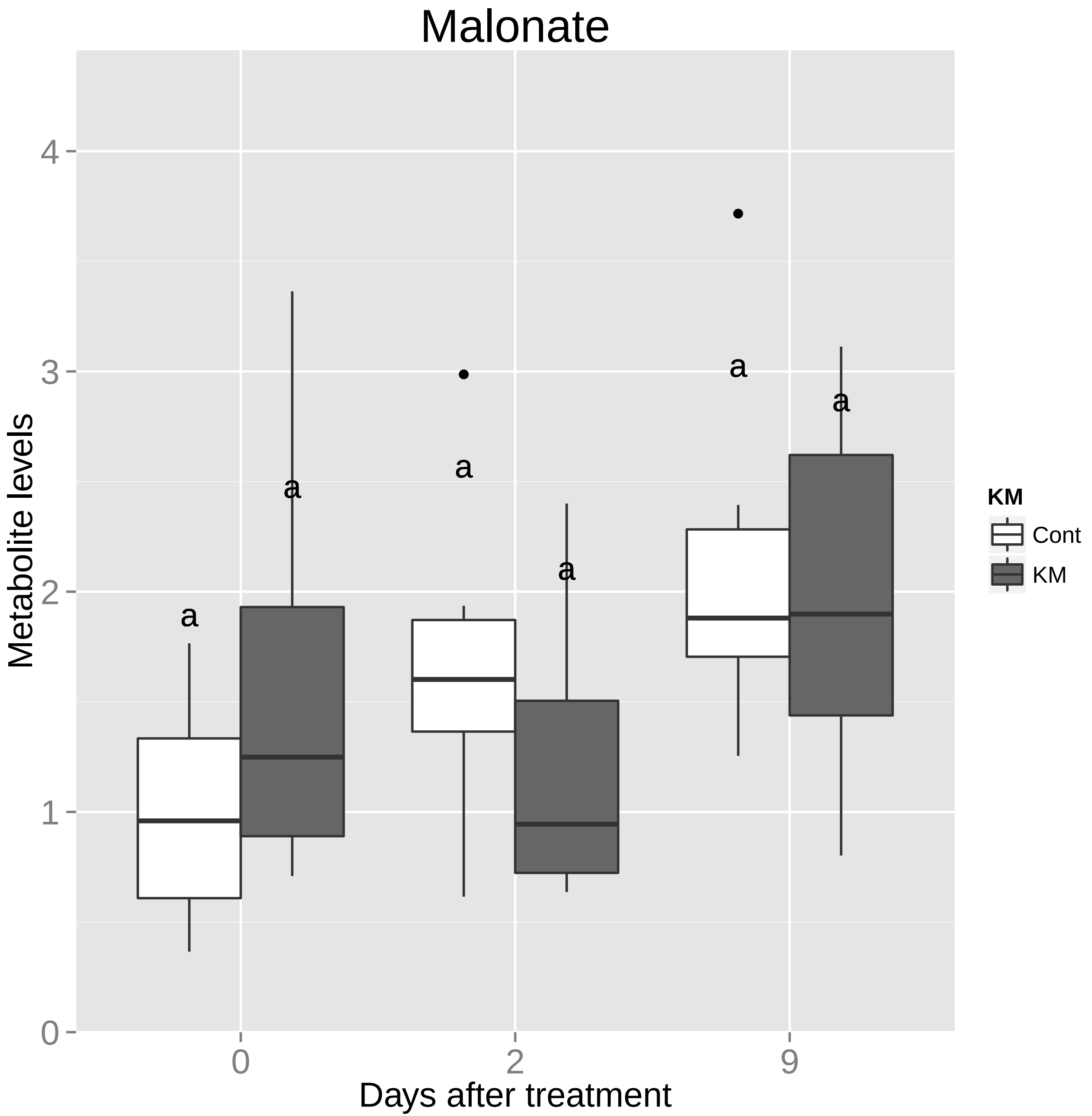

Malonate
Metabolite levels
Days after treatment
0
1
2
3
4
0
2
9
a
a
a
a
a
a
KM
Cont
KM

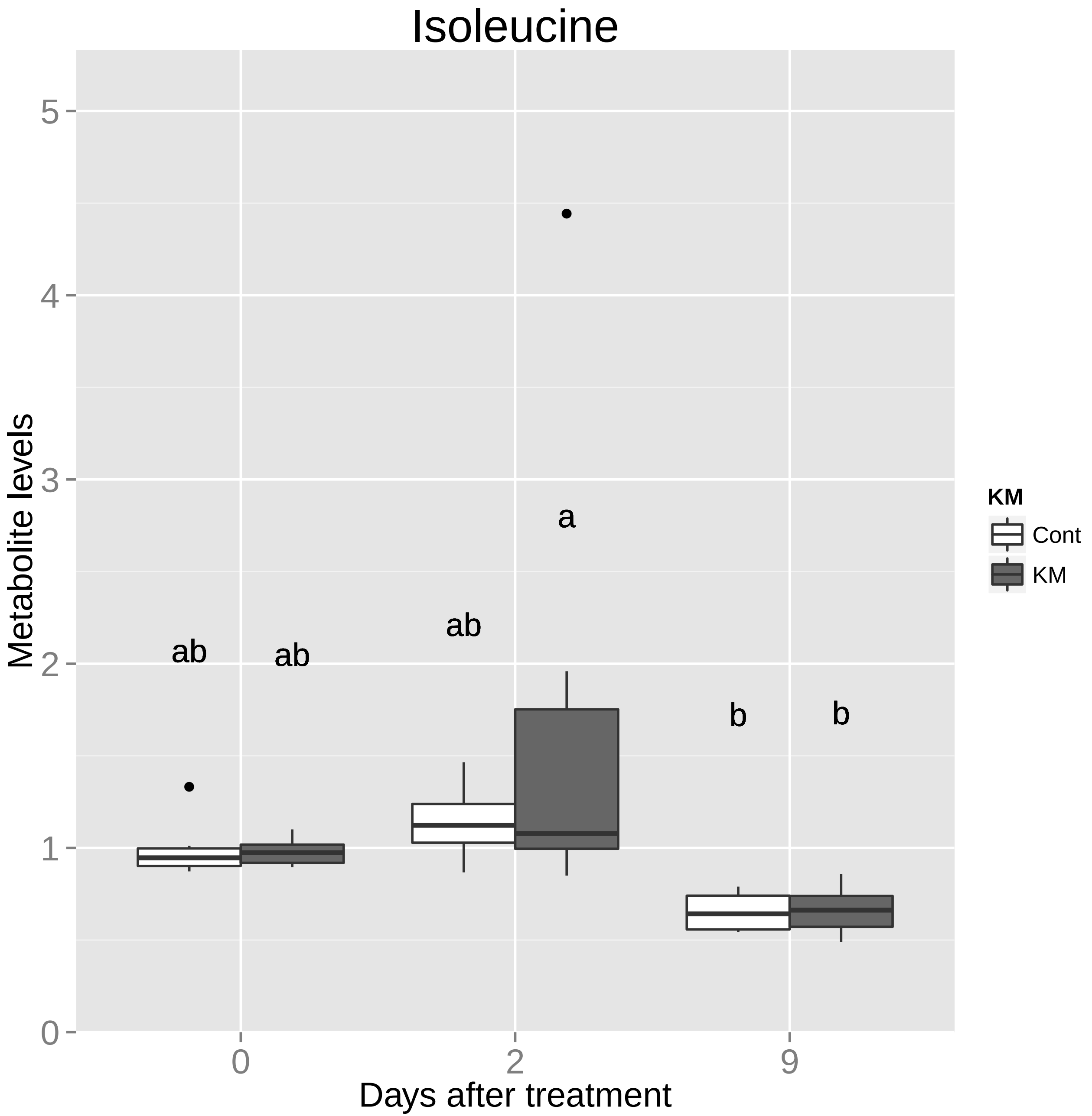

Isoleucine
Metabolite levels
Days after treatment
0
1
2
3
4
5
0
2
9
ab
ab
ab
a
b
b
KM
Cont
KM

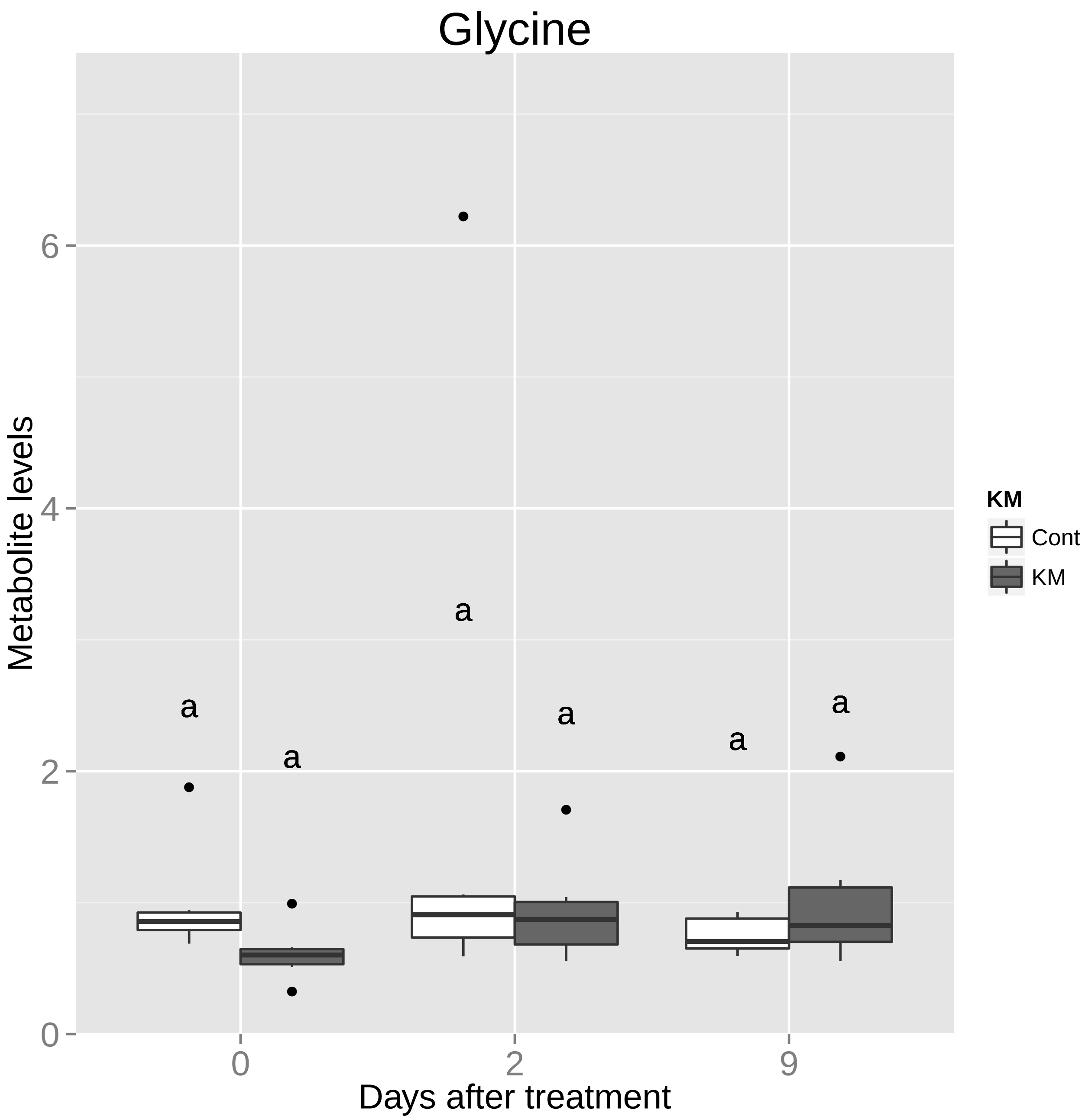

Glycine
Metabolite levels
Days after treatment
0
2
4
6
0
2
9
a
a
a
a
a
a
KM
Cont
KM

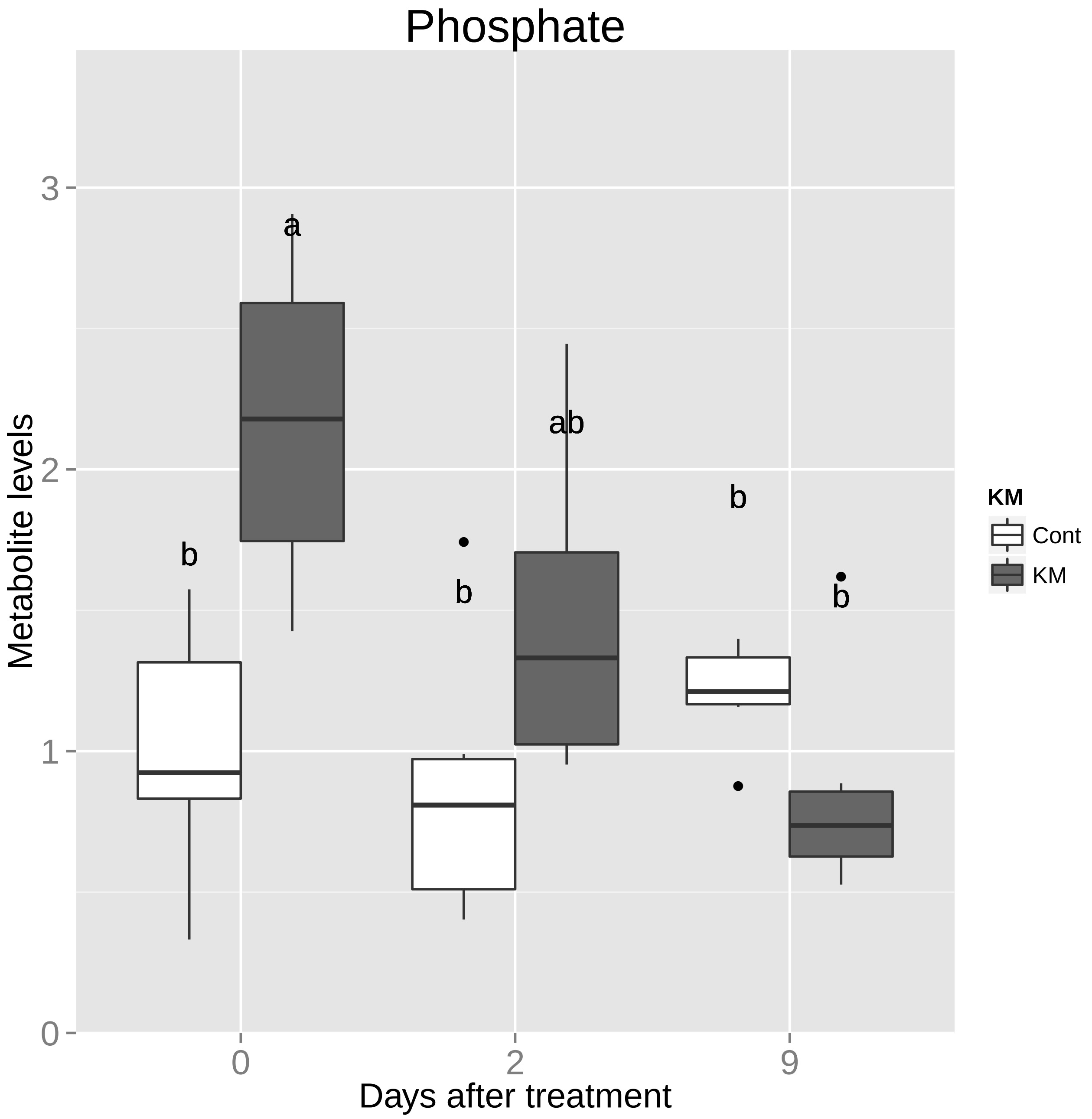
Phosphate
Metabolite levels
Days after treatment
0
1
2
3
0
2
9
a
b
ab
b
b
b
KM
Cont
KM

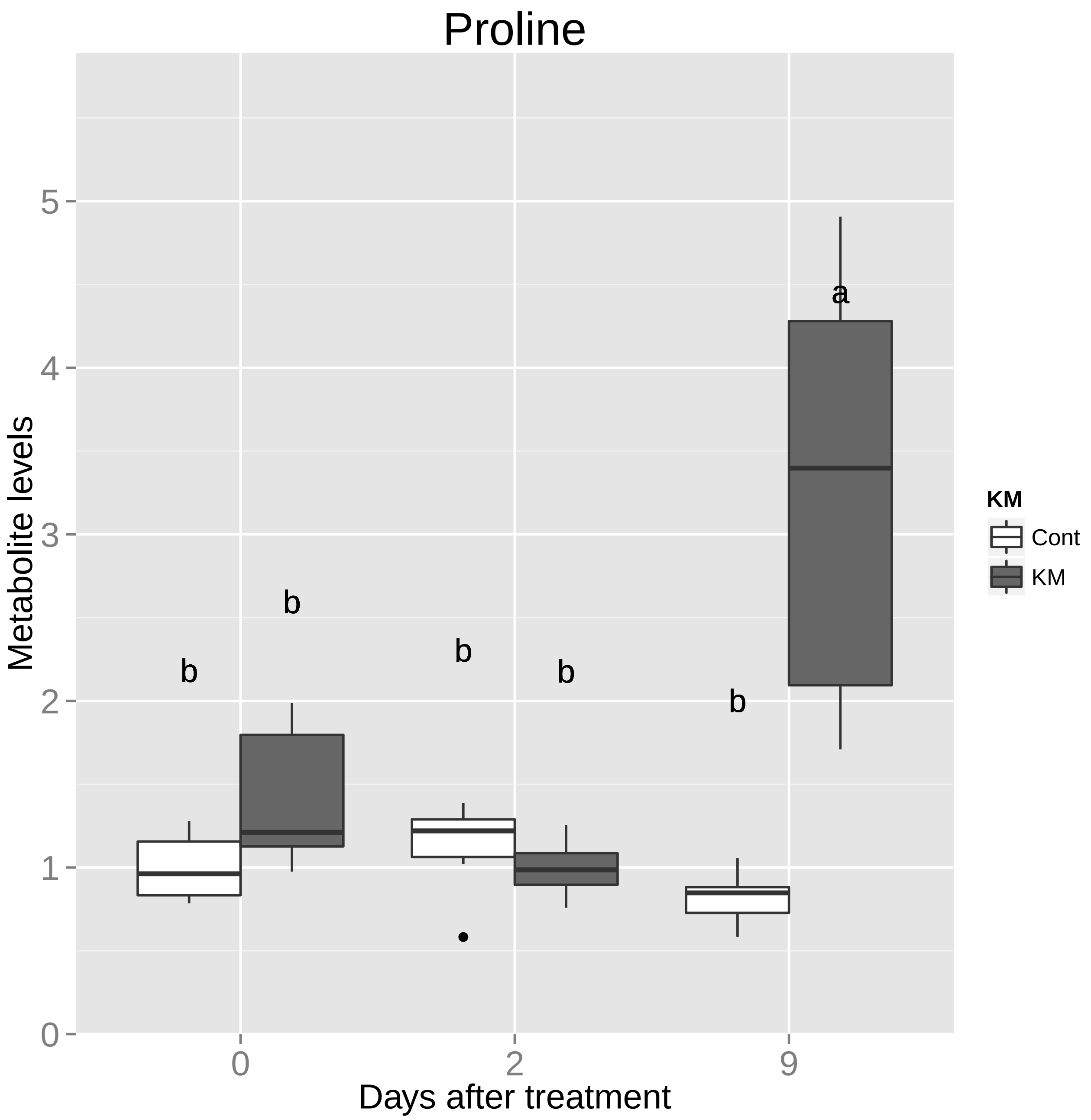

Proline
Metabolite levels
0
1
2
3
4
5
b
b
b
b
b
a
0
2
9
Days after treatment
KM
Cont
KM

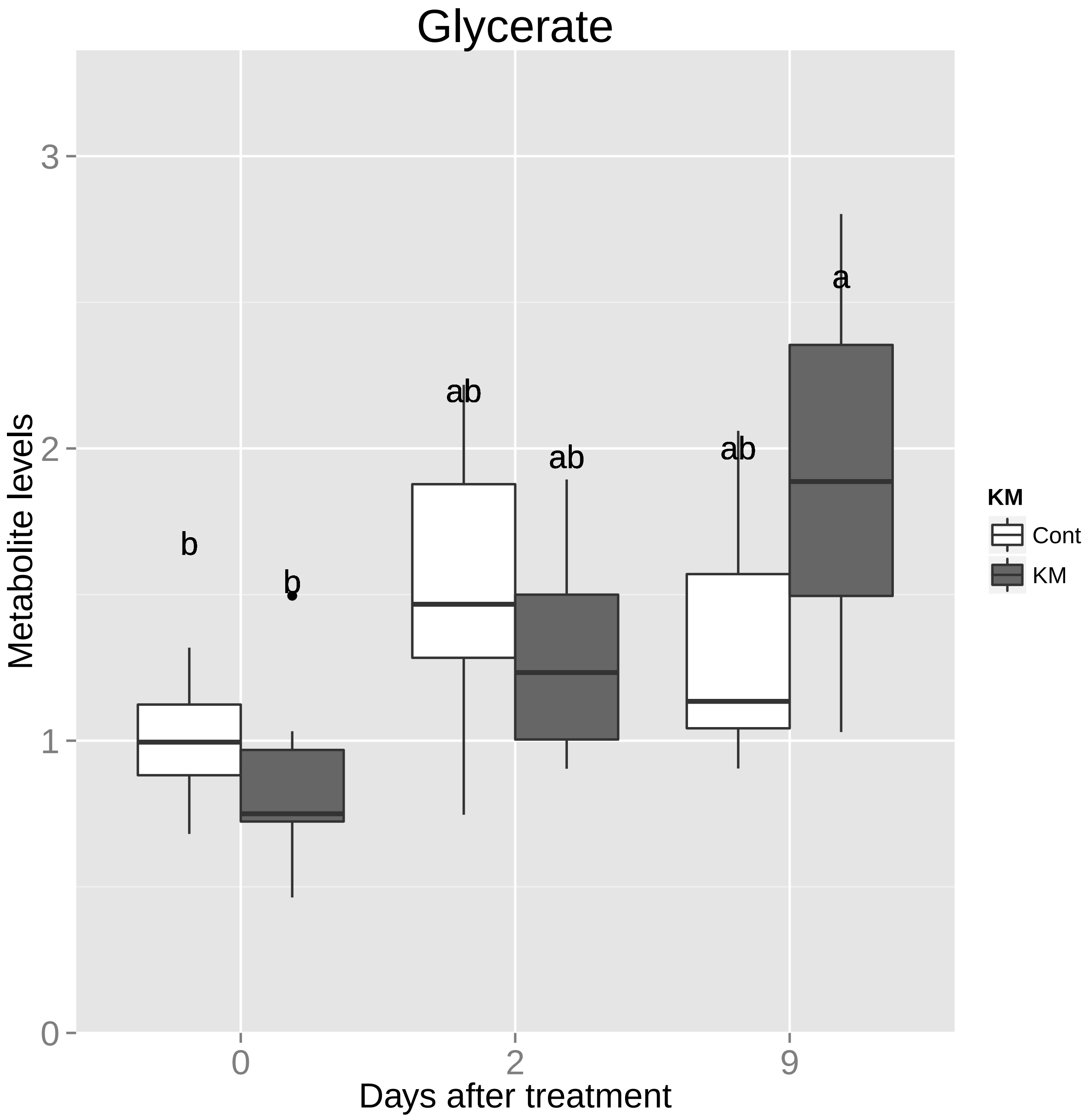
Glycerate
Metabolite levels
Days after treatment
0
1
2
3
0
2
9
b
b
ab
ab
ab
a
KM
Cont
KM

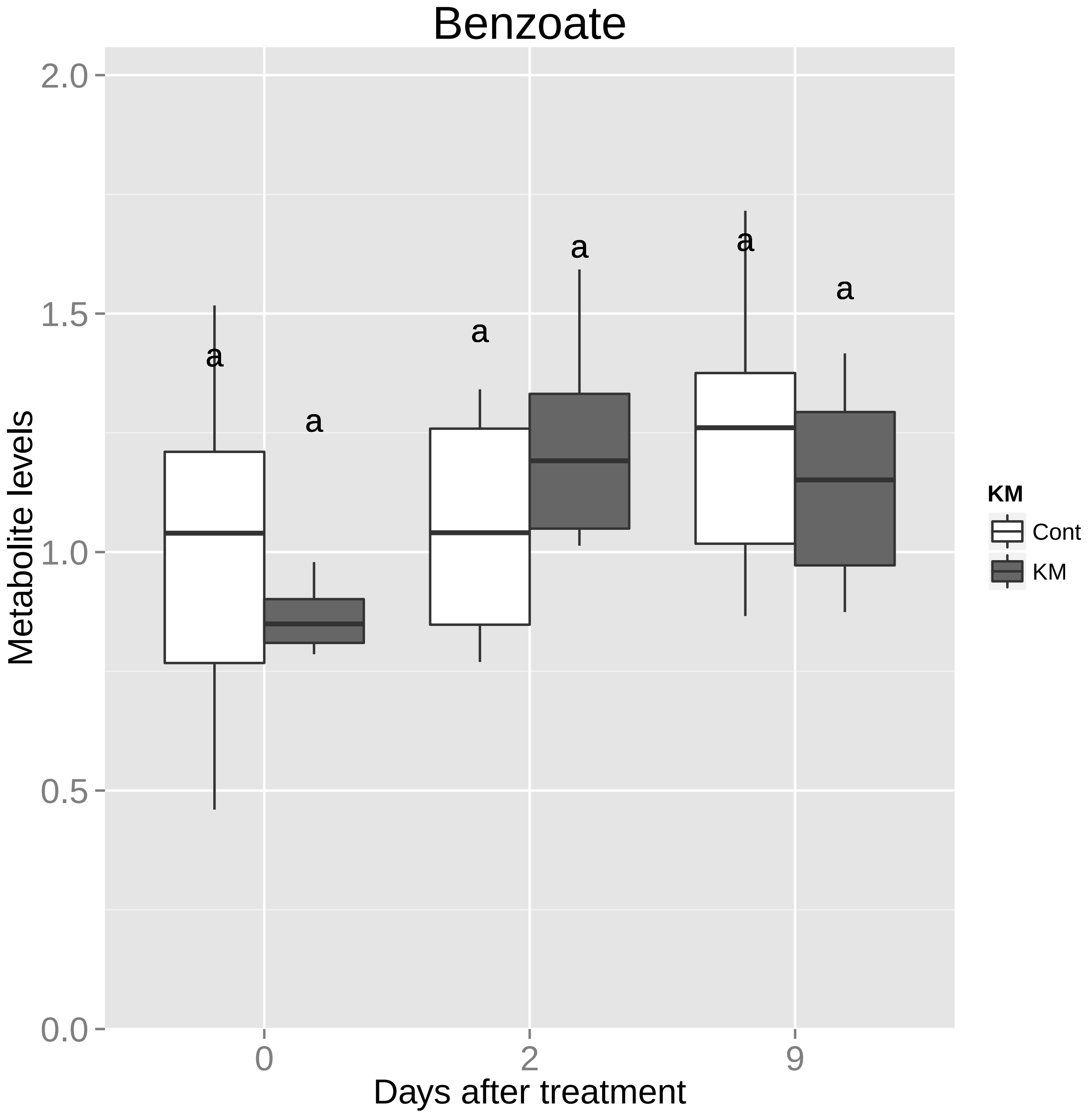

Benzoate
Metabolite levels
Days after treatment
0.0
0.5
1.0
1.5
2.0
0
2
9
a
a
a
a
a
a
KM
Cont
KM

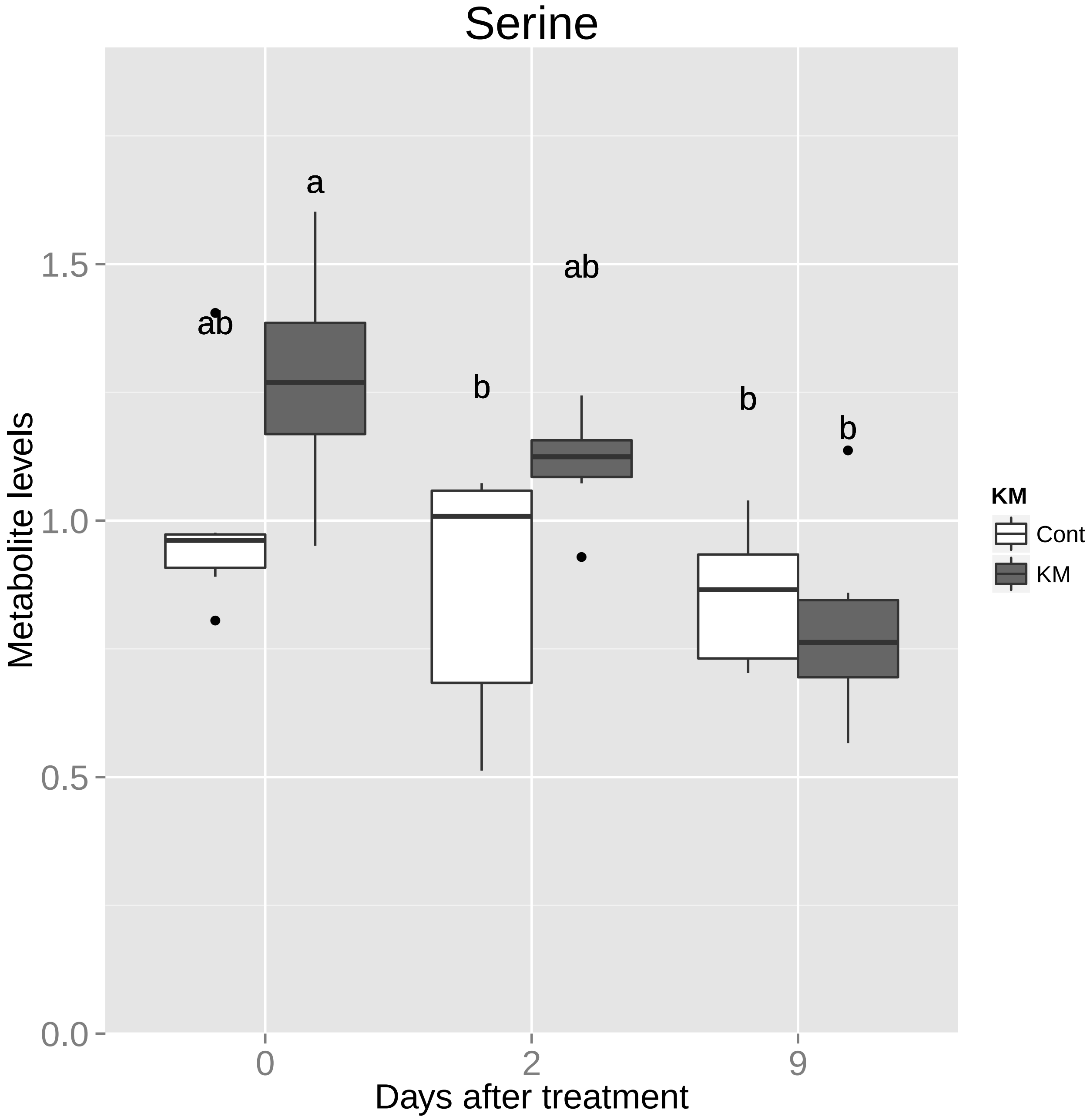

Serine
Metabolite levels
Days after treatment
0.0
0.5
1.0
1.5
0
2
9
a
ab
ab
b
b
b
KM
Cont
KM

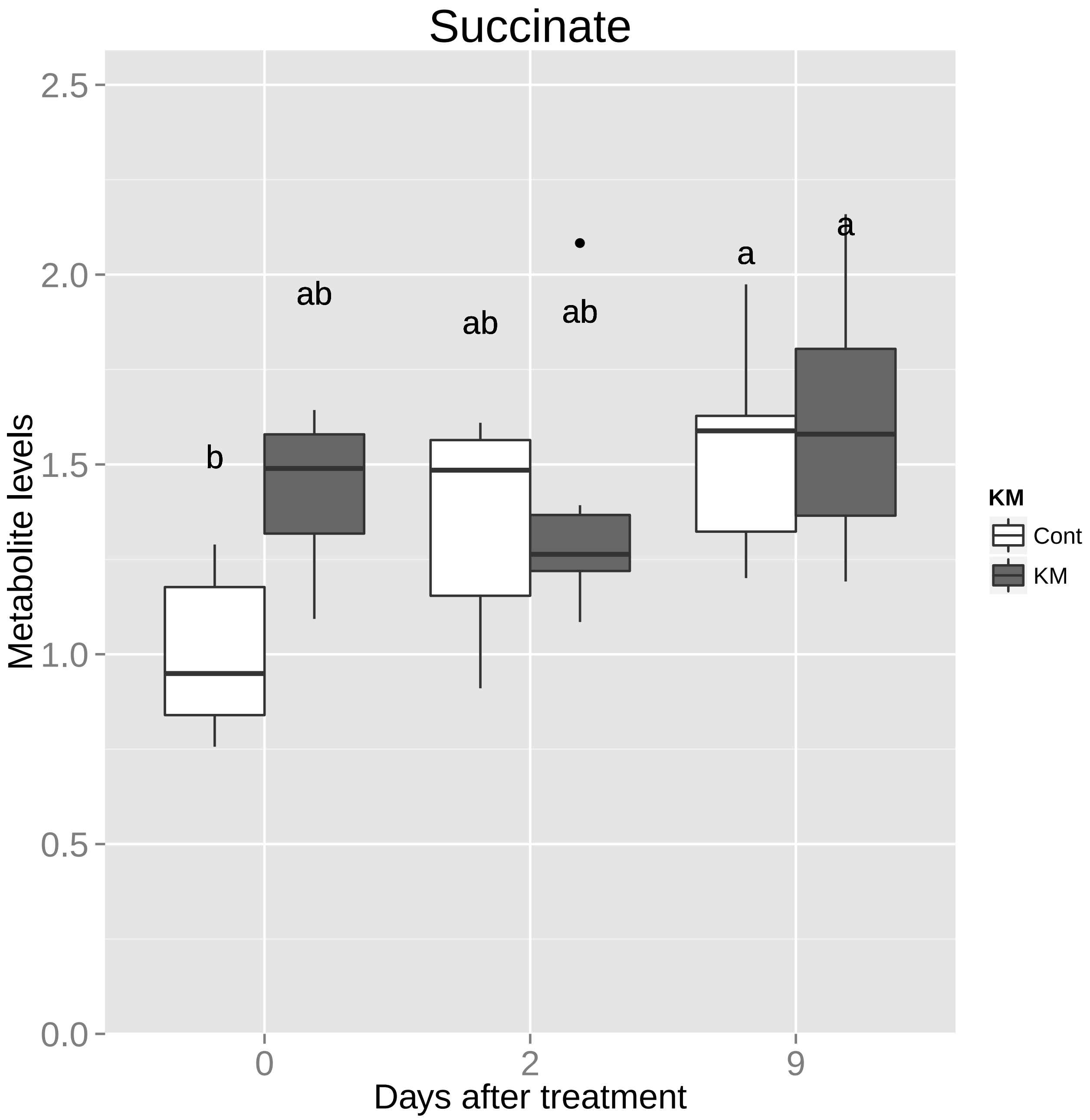

Succinate
Metabolite levels
0.0
0.5
1.0
1.5
2.0
2.5
b
ab
ab
ab
a
a
0
2
9
Days after treatment
KM
Cont
KM

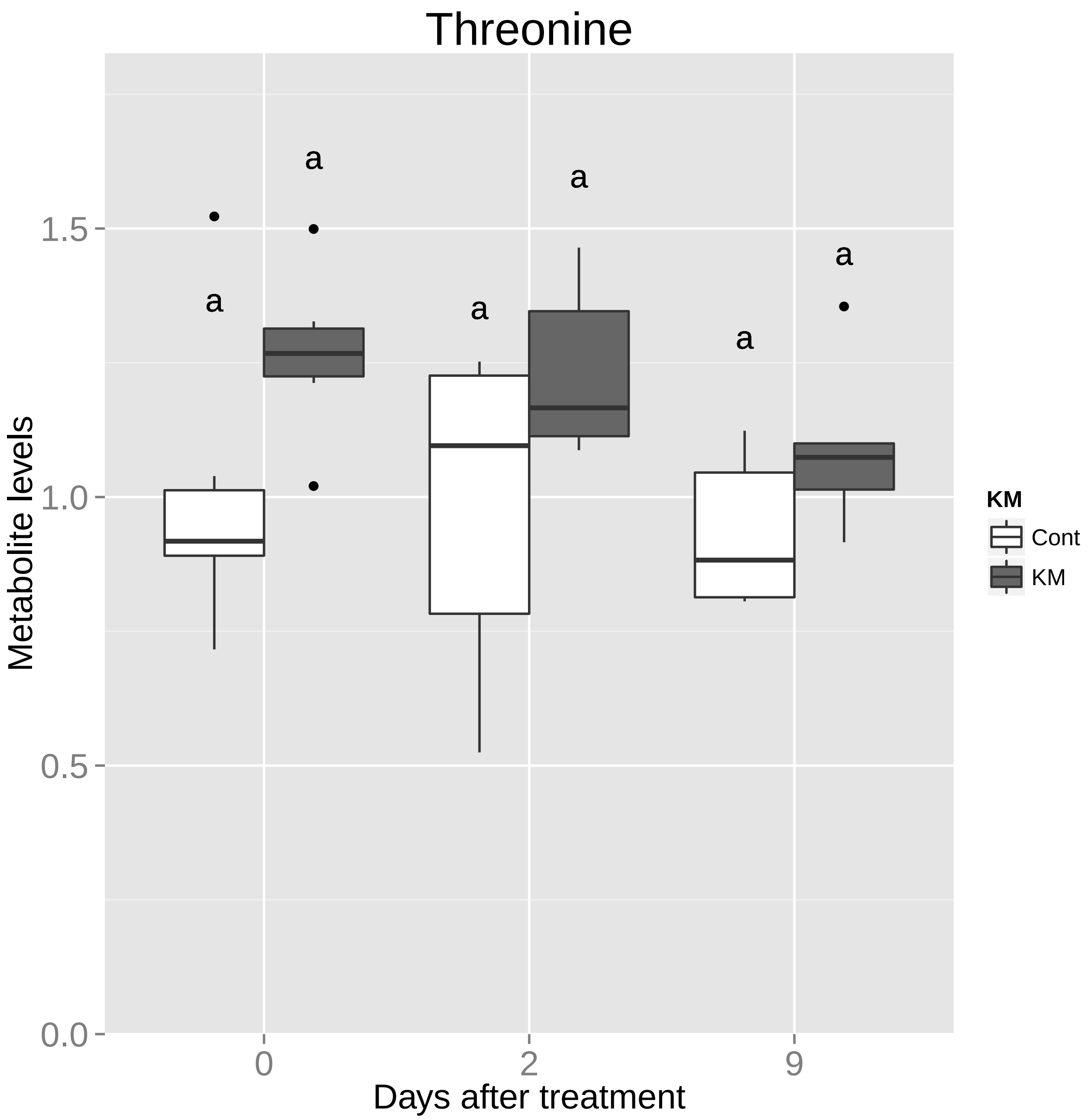

Threonine
Metabolite levels
1.5
1.0
0.5
0.0
0
2
9
Days after treatment
a
a
a
a
a
a
KM
Cont
KM

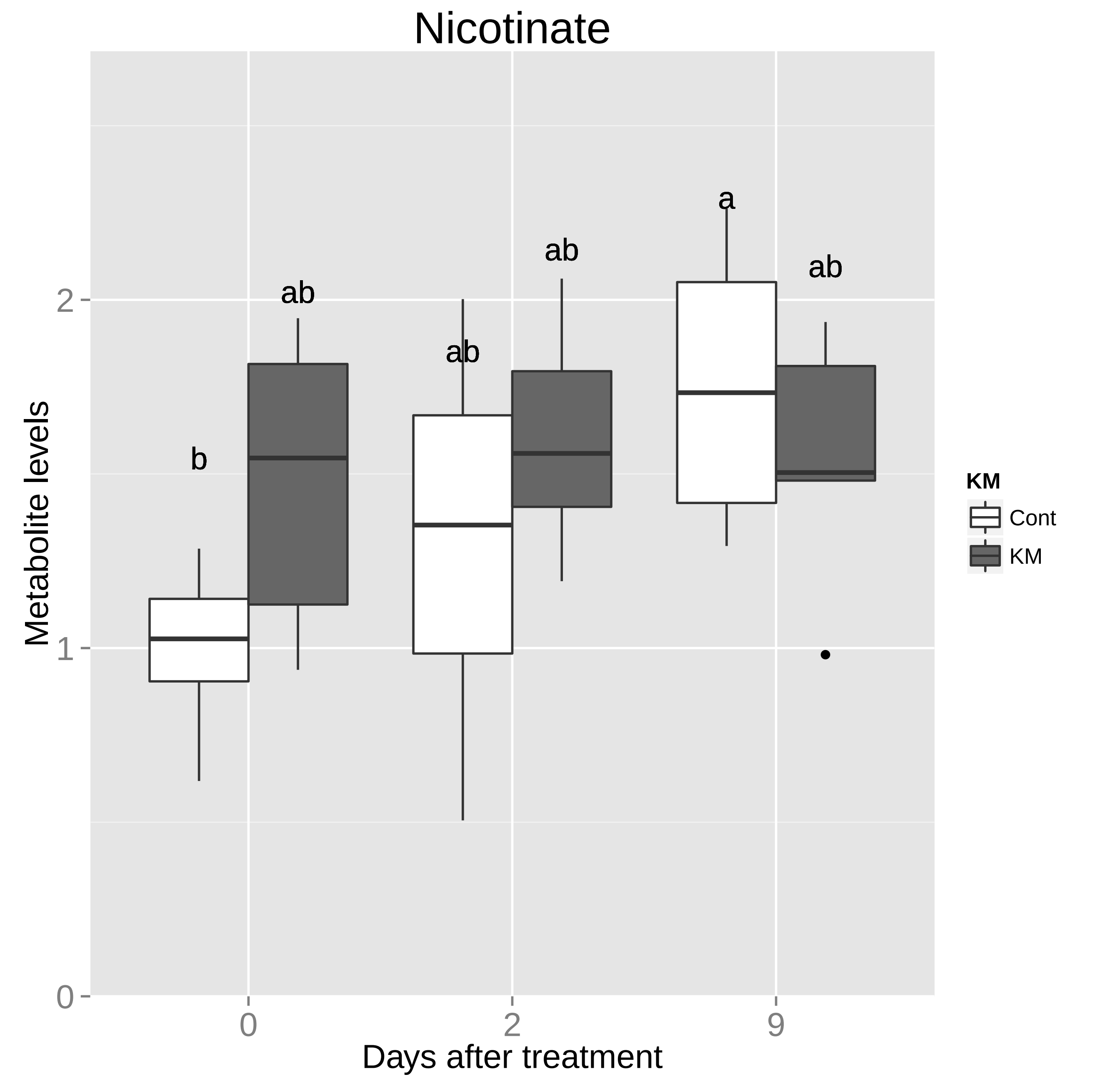

Nicotinate
Metabolite levels
0
1
2
b
ab
ab
ab
a
ab
0
2
9
Days after treatment
KM
Cont
KM

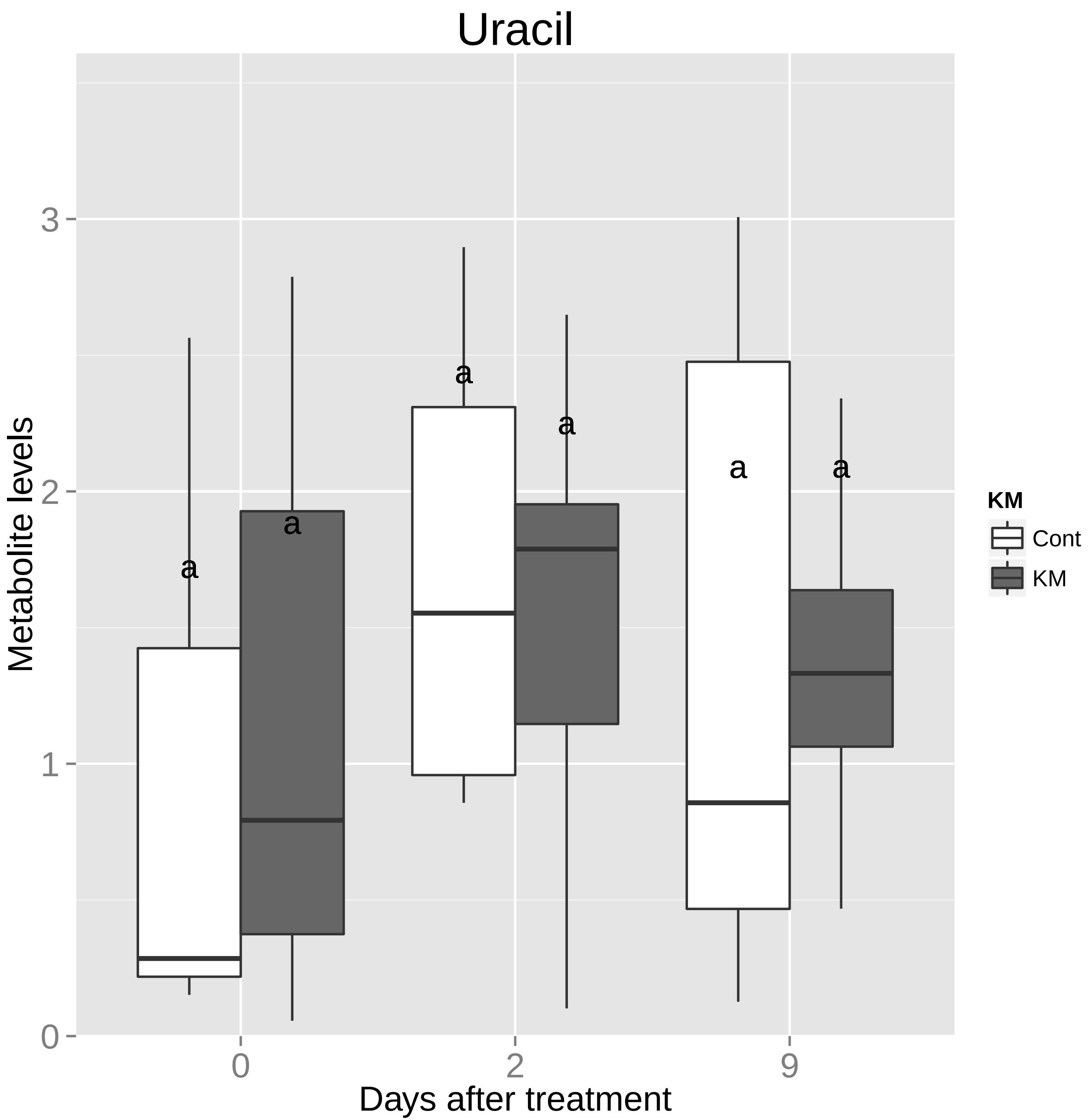

Uracil
Metabolite levels
3
2
1
0
a
a
a
a
a
a
0
2
9
Days after treatment
KM
Cont
KM

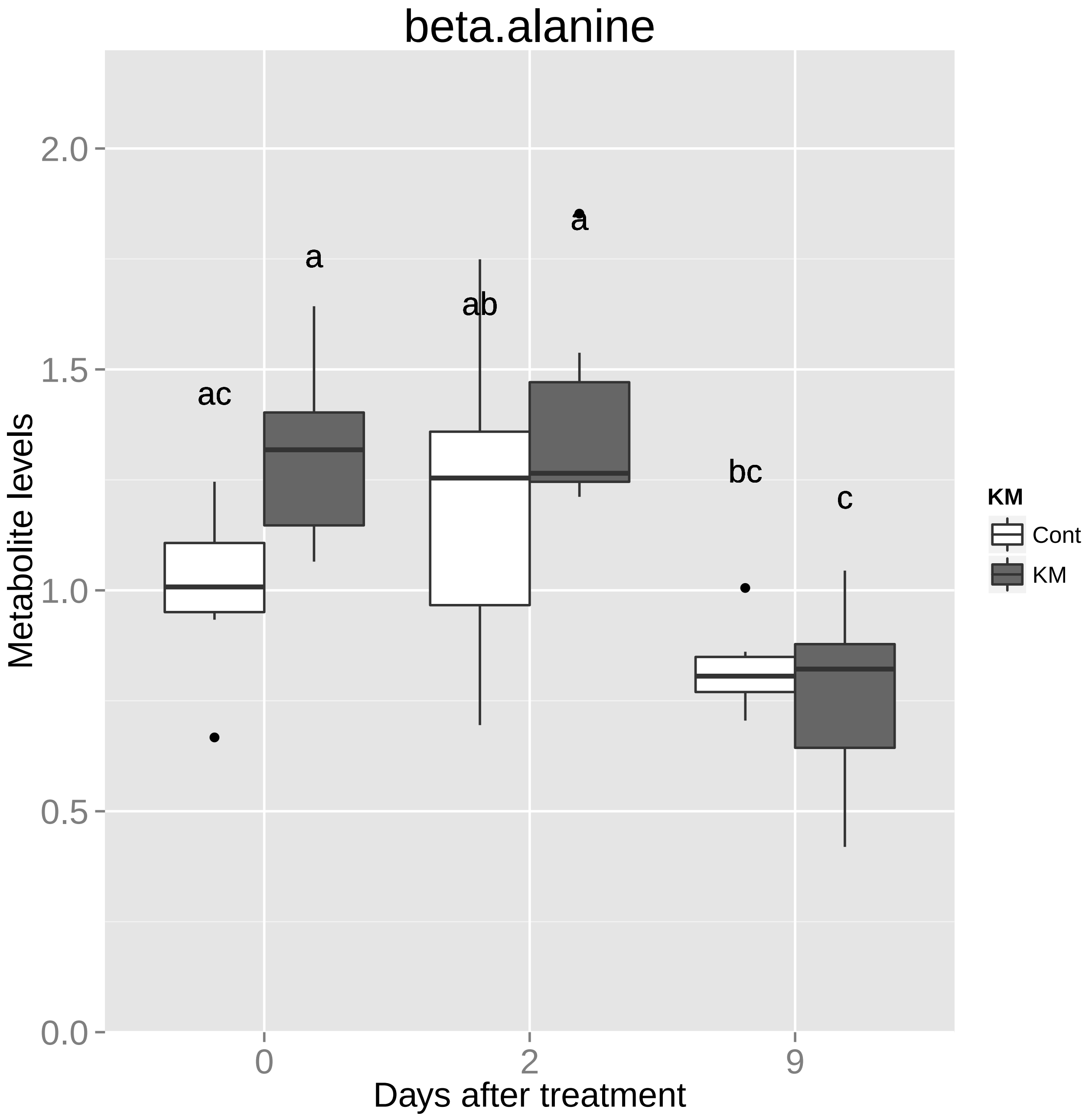

beta.alanine
Metabolite levels
Days after treatment
0.0
0.5
1.0
1.5
2.0
0
2
9
ac
a
ab
a
bc
c
KM
Cont
KM

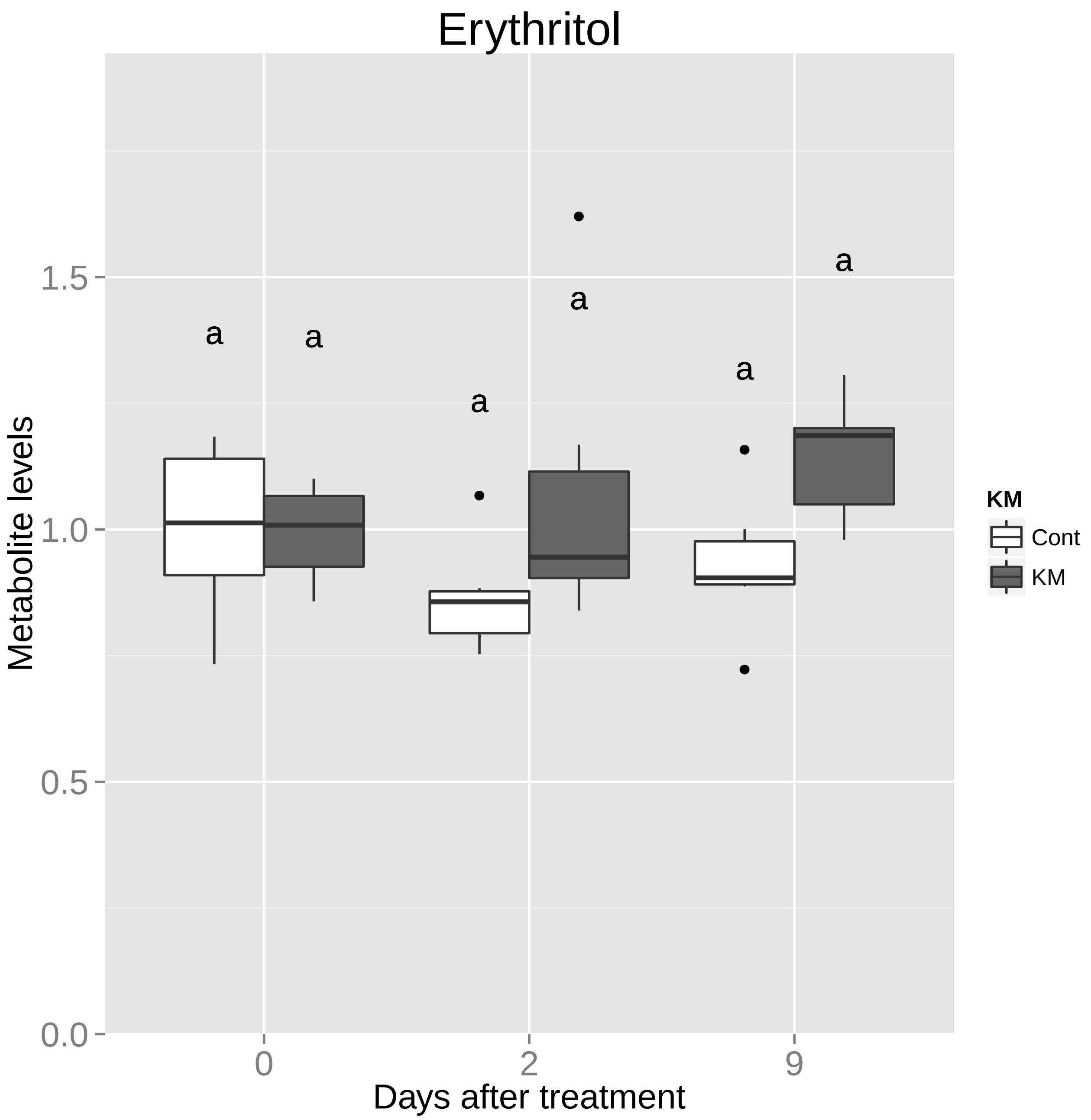

Erythritol
Metabolite levels
Days after treatment
0.0
0.5
1.0
1.5
0
2
9
a
a
a
a
a
a
KM
Cont
KM

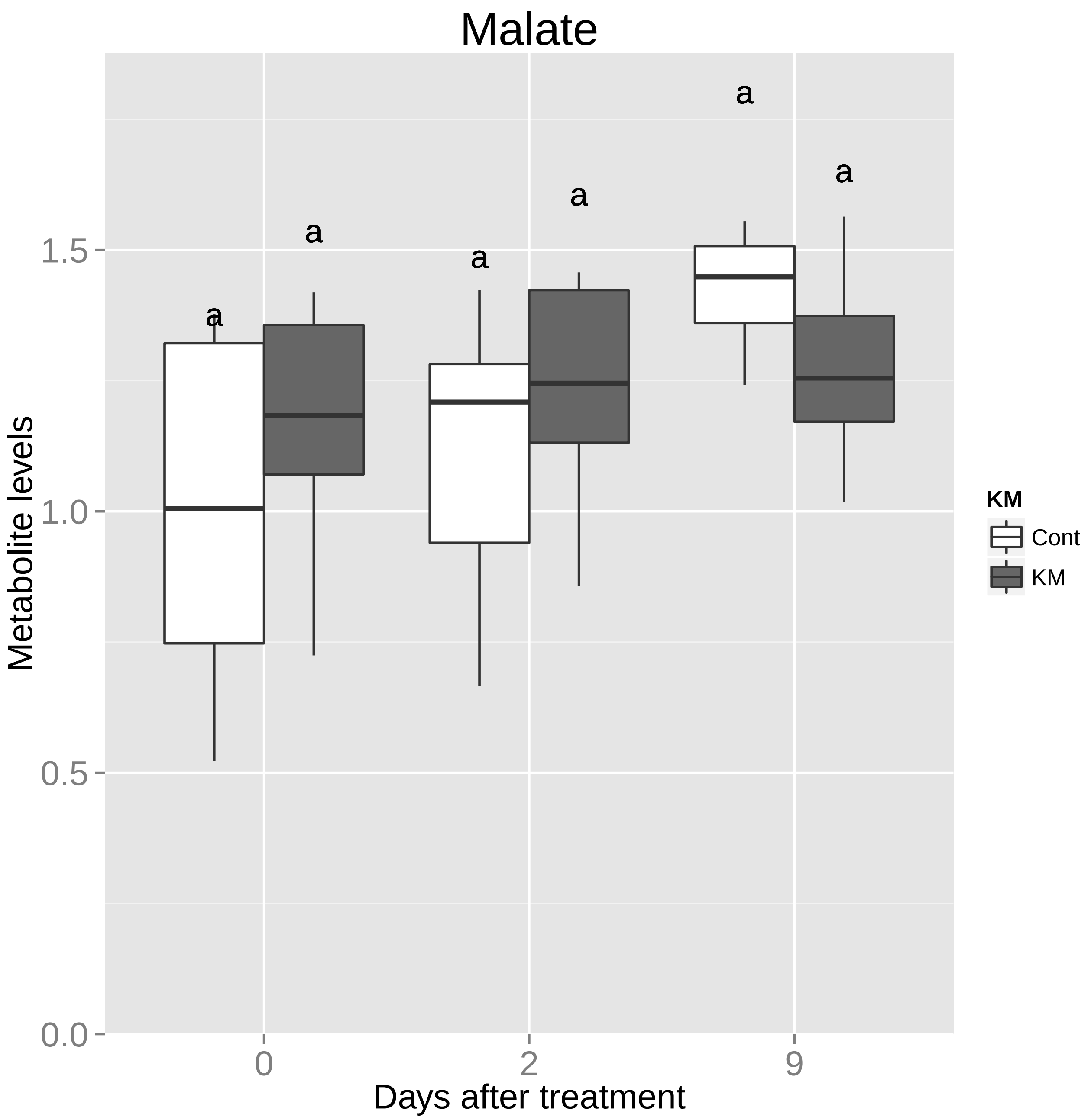

Malate
a
a
a
a
a
a
1.5
1.0
0.5
0.0
Metabolite levels
0
2
9
Days after treatment
KM
Cont
KM

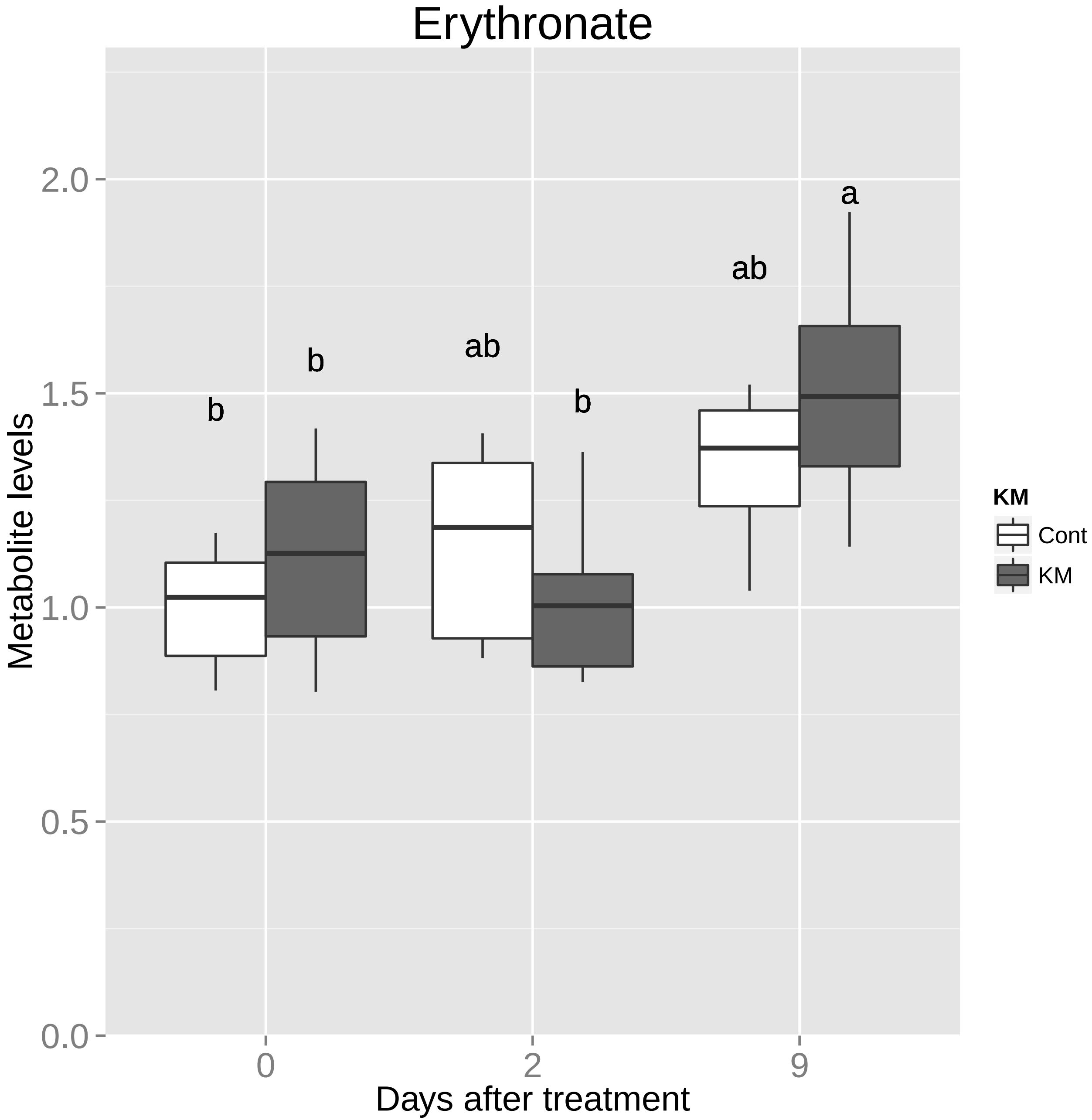
Erythronate
Metabolite levels
2.0
1.5
1.0
0.5
0.0
b
b
ab
b
ab
a
0
2
9
Days after treatment
KM
Cont
KM

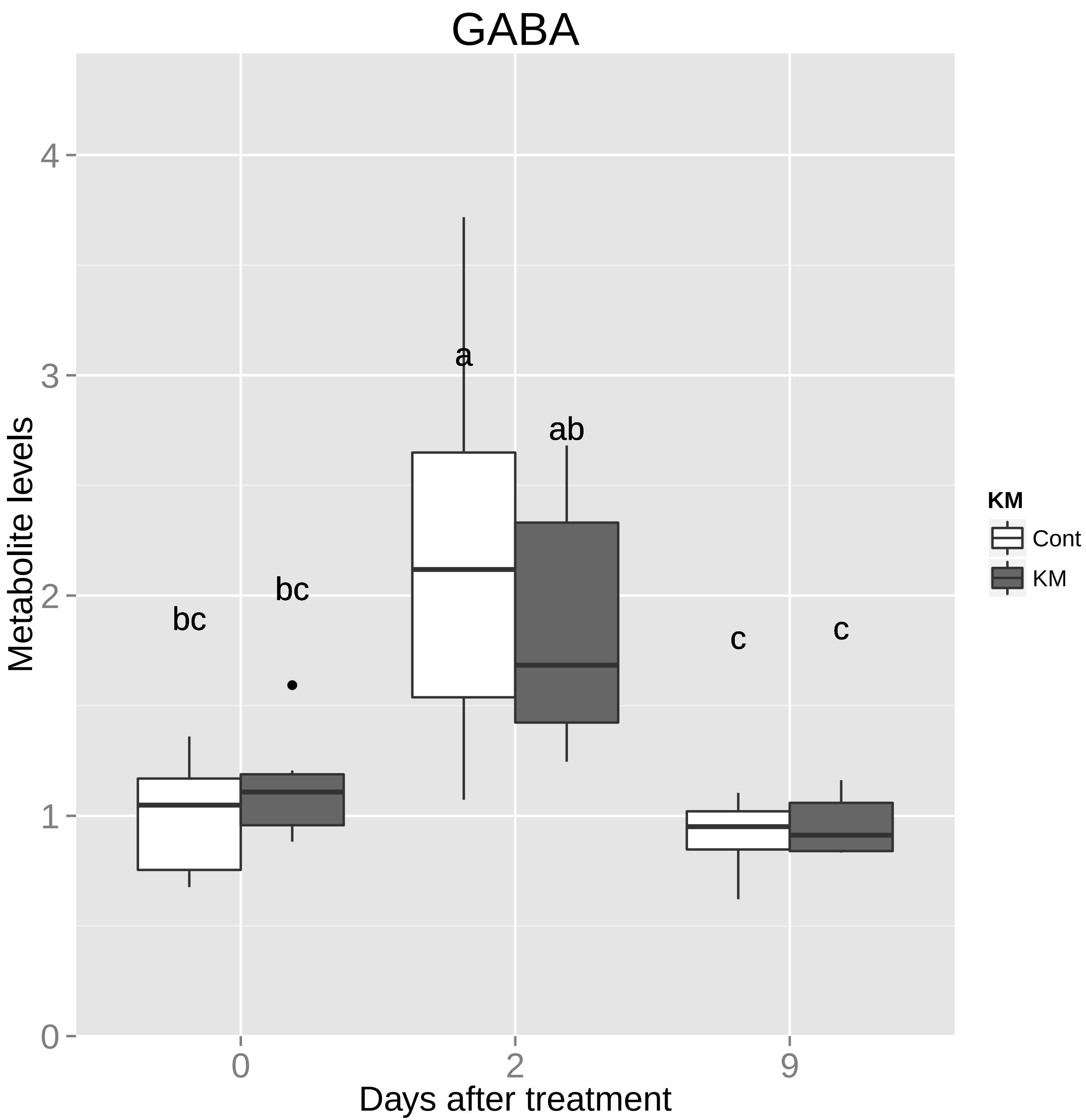
GABA
Metabolite levels
Days after treatment
0
1
2
3
4
0
2
9
bc
bc
a
ab
c
c
KM
Cont
KM

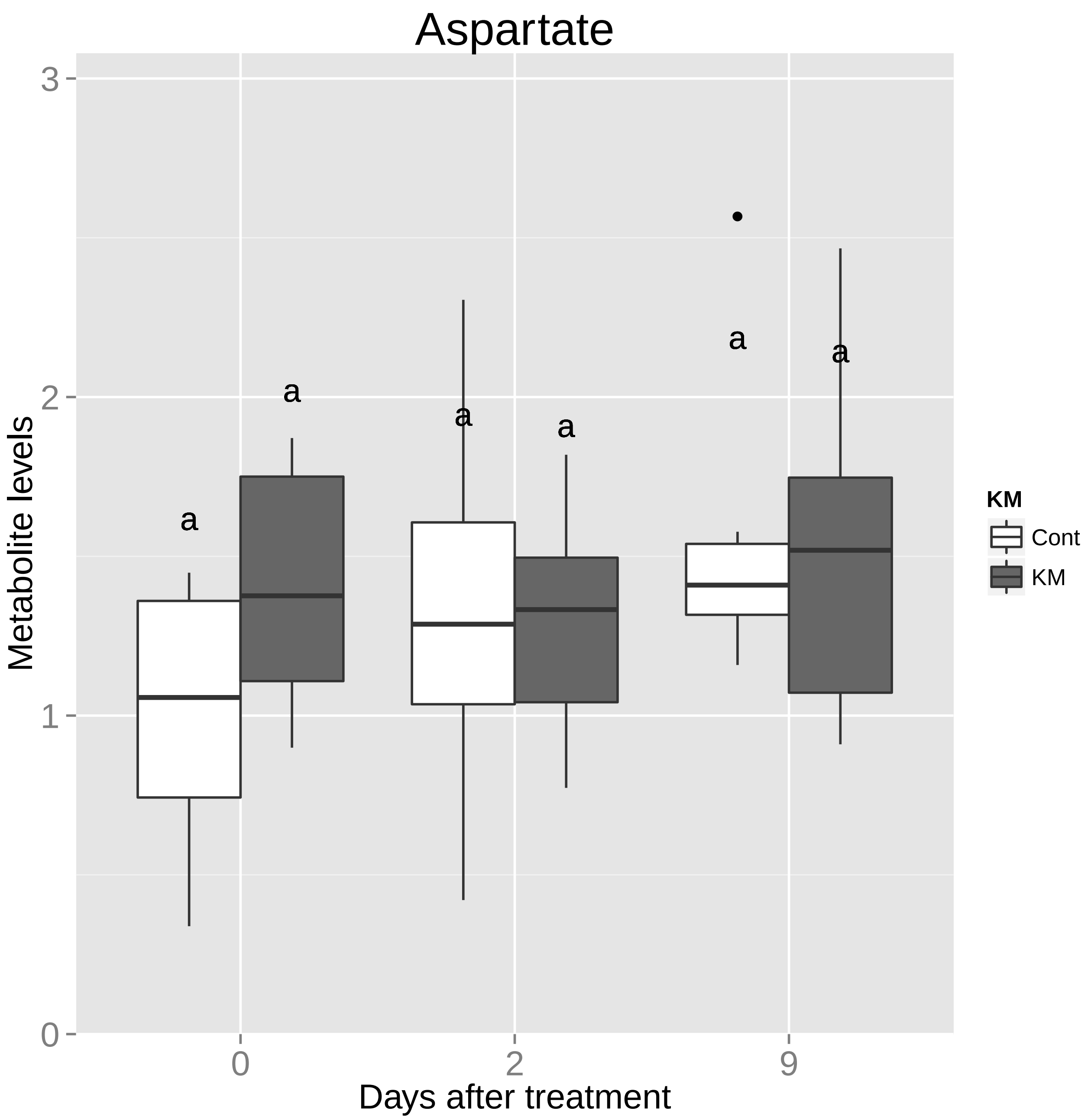
Aspartate
Metabolite levels
Days after treatment
0
1
2
3
0
2
9
a
a
a
a
a
a
KM
Cont
KM

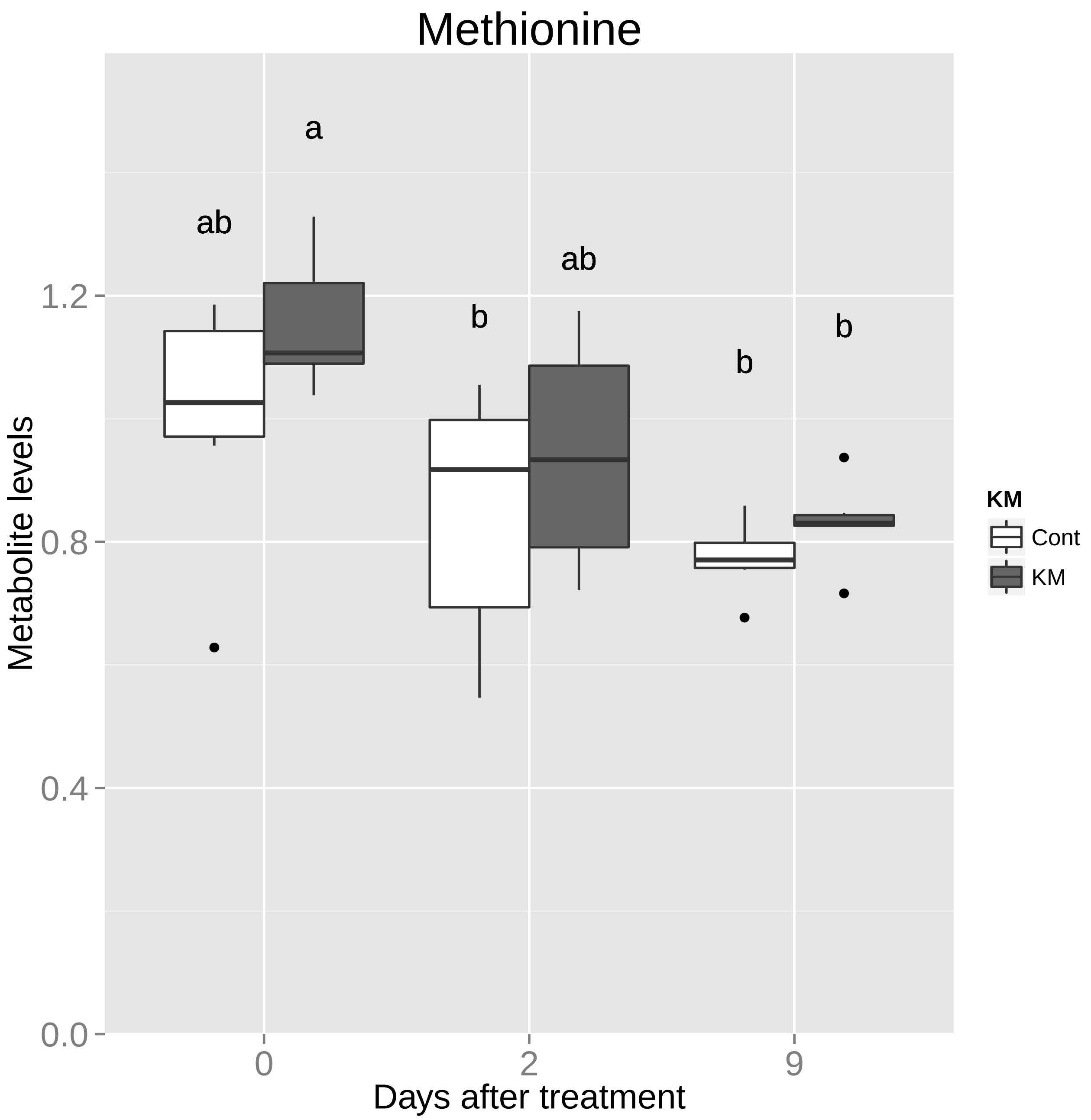

Methionine
a
ab
ab
b
b
b
1.2
0.8
0.4
0.0
Metabolite levels
0
2
9
Days after treatment
KM
Cont
KM

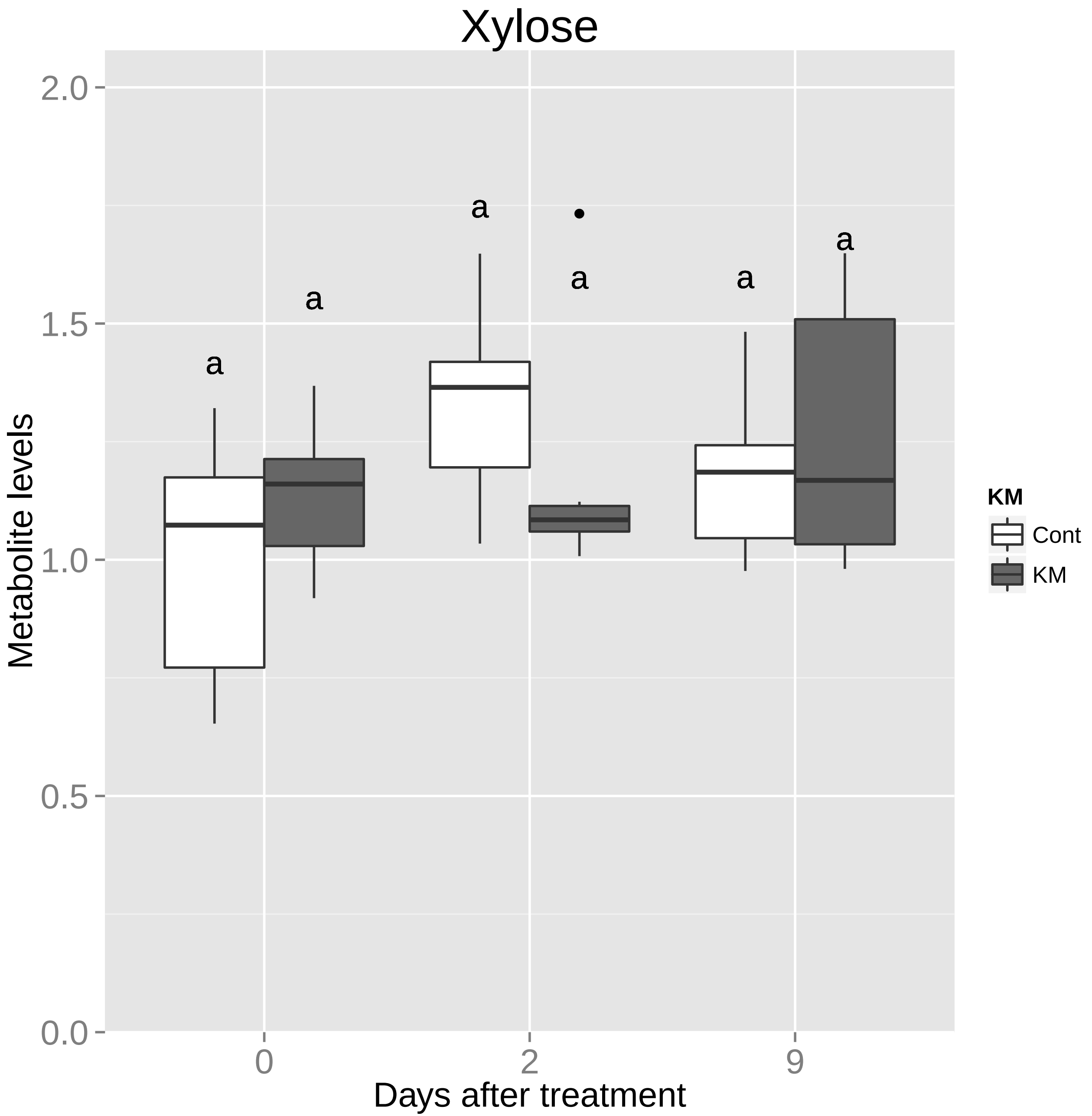
Xylose
Metabolite levels
2.0
1.5
1.0
0.5
0.0
a
a
a
a
a
a
0
2
9
Days after treatment
KM
Cont
KM

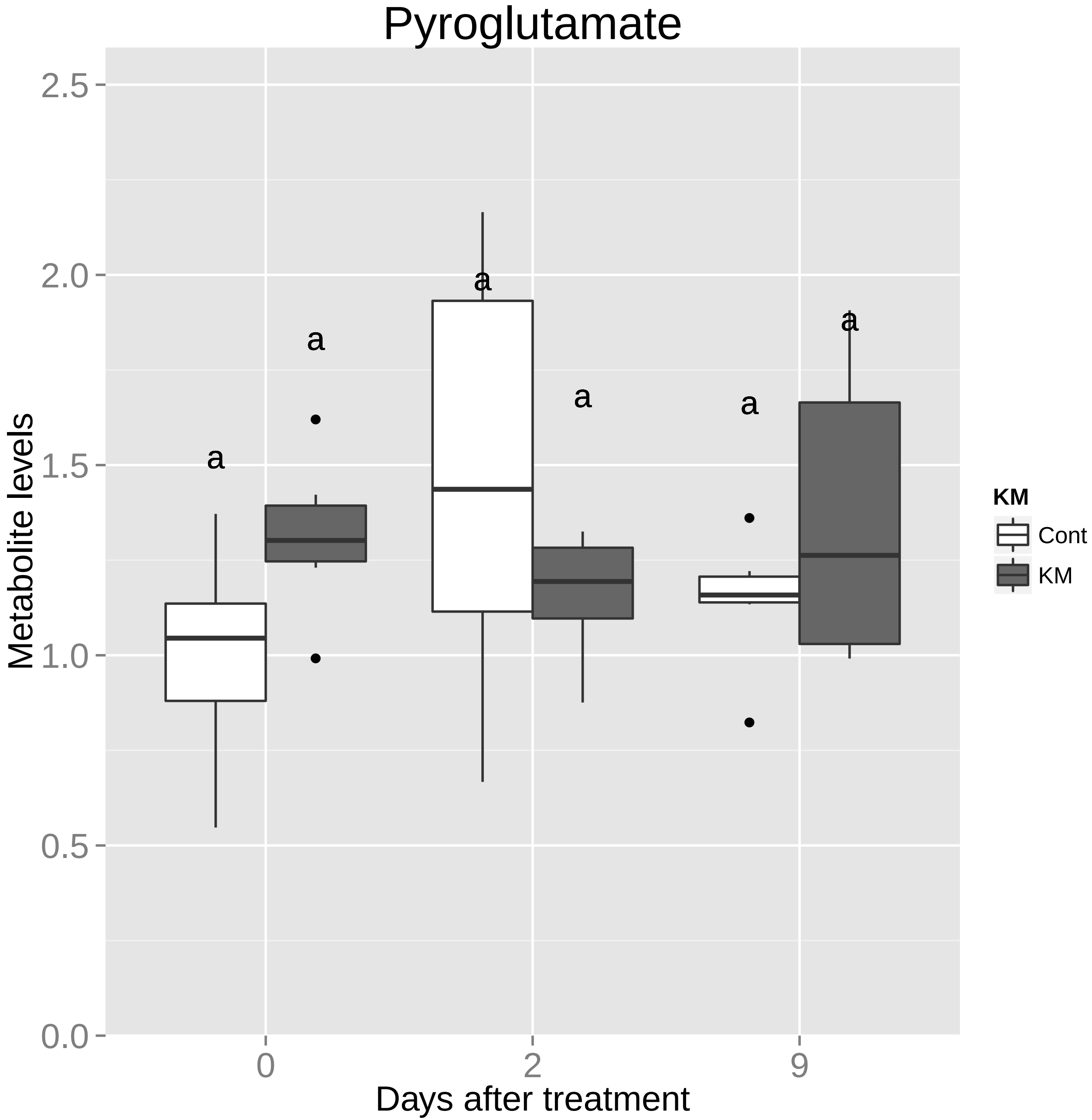

Pyroglutamate
Metabolite levels
2.5
2.0
1.5
1.0
0.5
0.0
0
2
9
Days after treatment
a
a
a
a
a
a
KM
Cont
KM

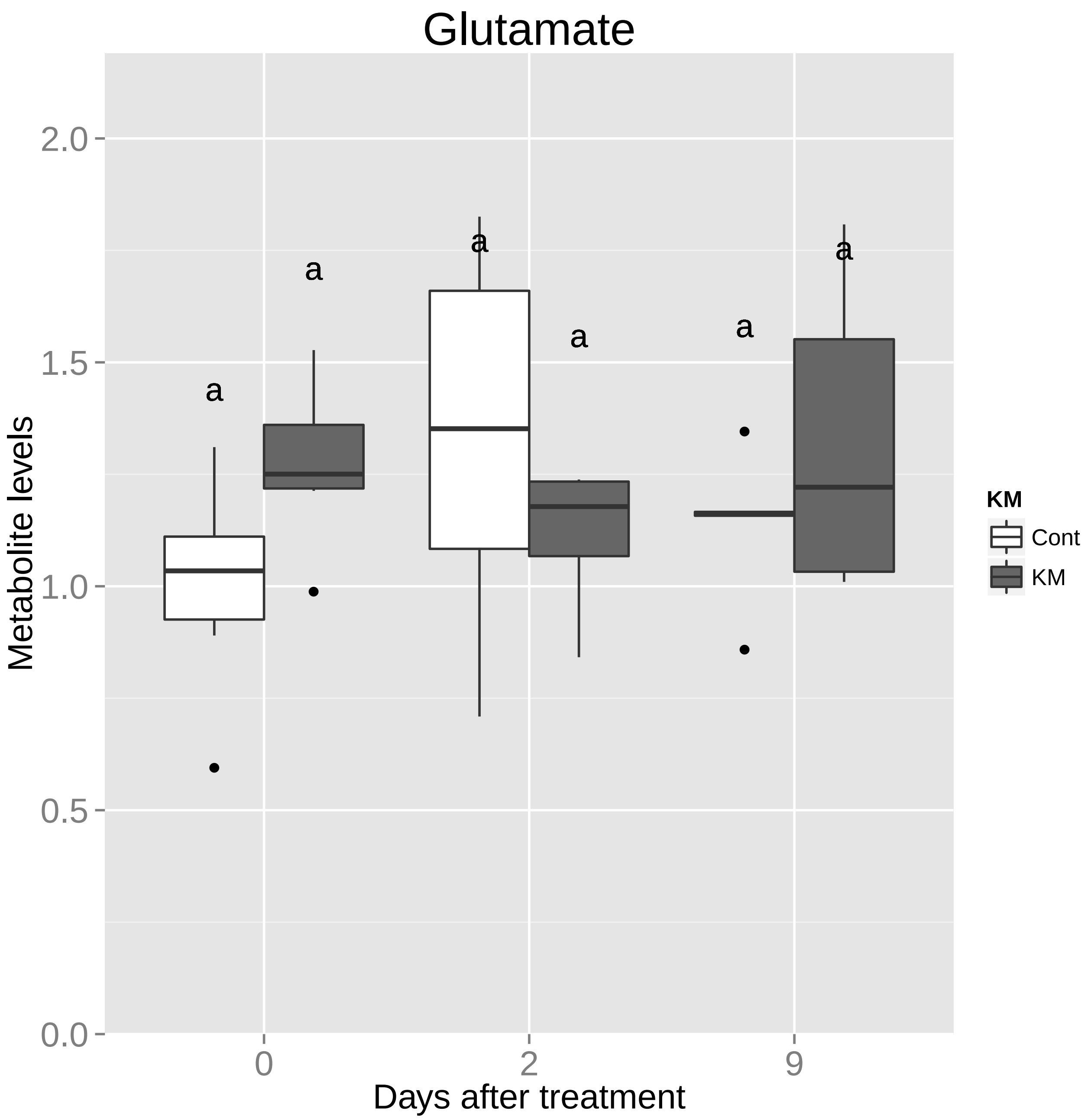
Glutamate
Metabolite levels
Days after treatment
0.0
0.5
1.0
1.5
2.0
0
2
9
a
a
a
a
a
a
KM
Cont
KM

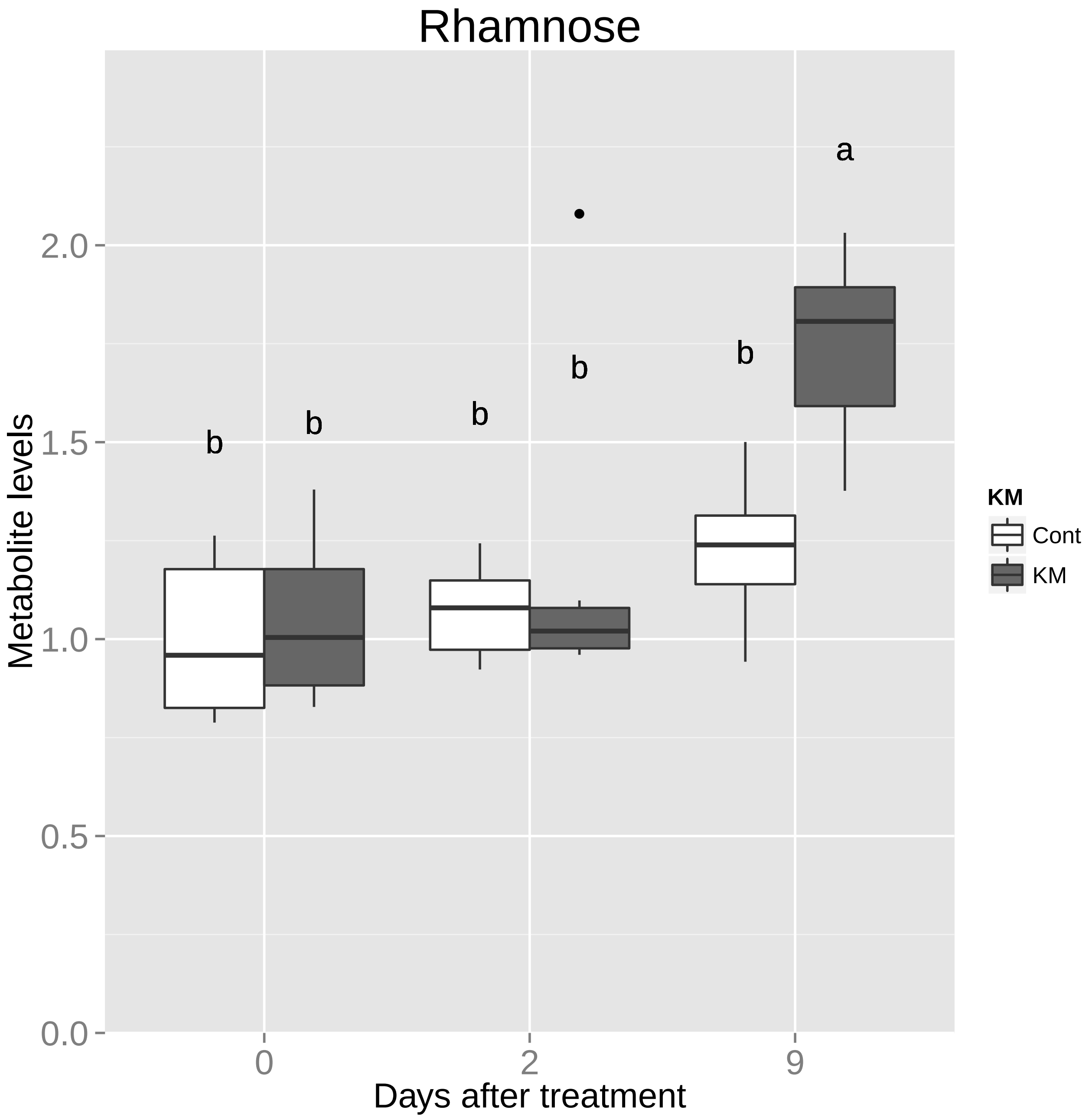
Rhamnose
Metabolite levels
2.0
1.5
1.0
0.5
0.0
b
b
b
b
b
a
0
2
9
Days after treatment
KM
Cont
KM

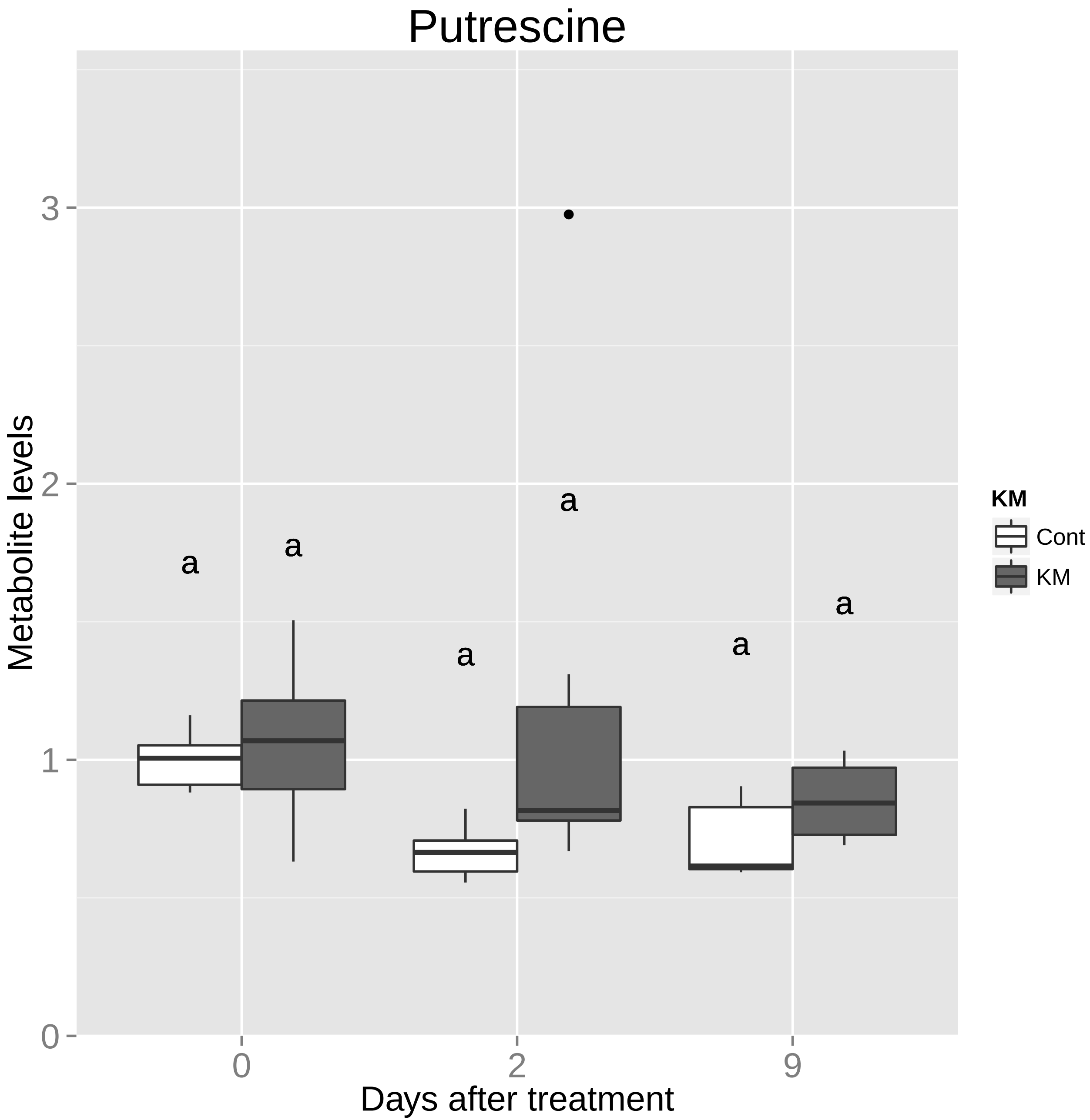

Putrescine
Metabolite levels
0
1
2
3
a
a
a
a
a
a
0
2
9
Days after treatment
KM
Cont
KM

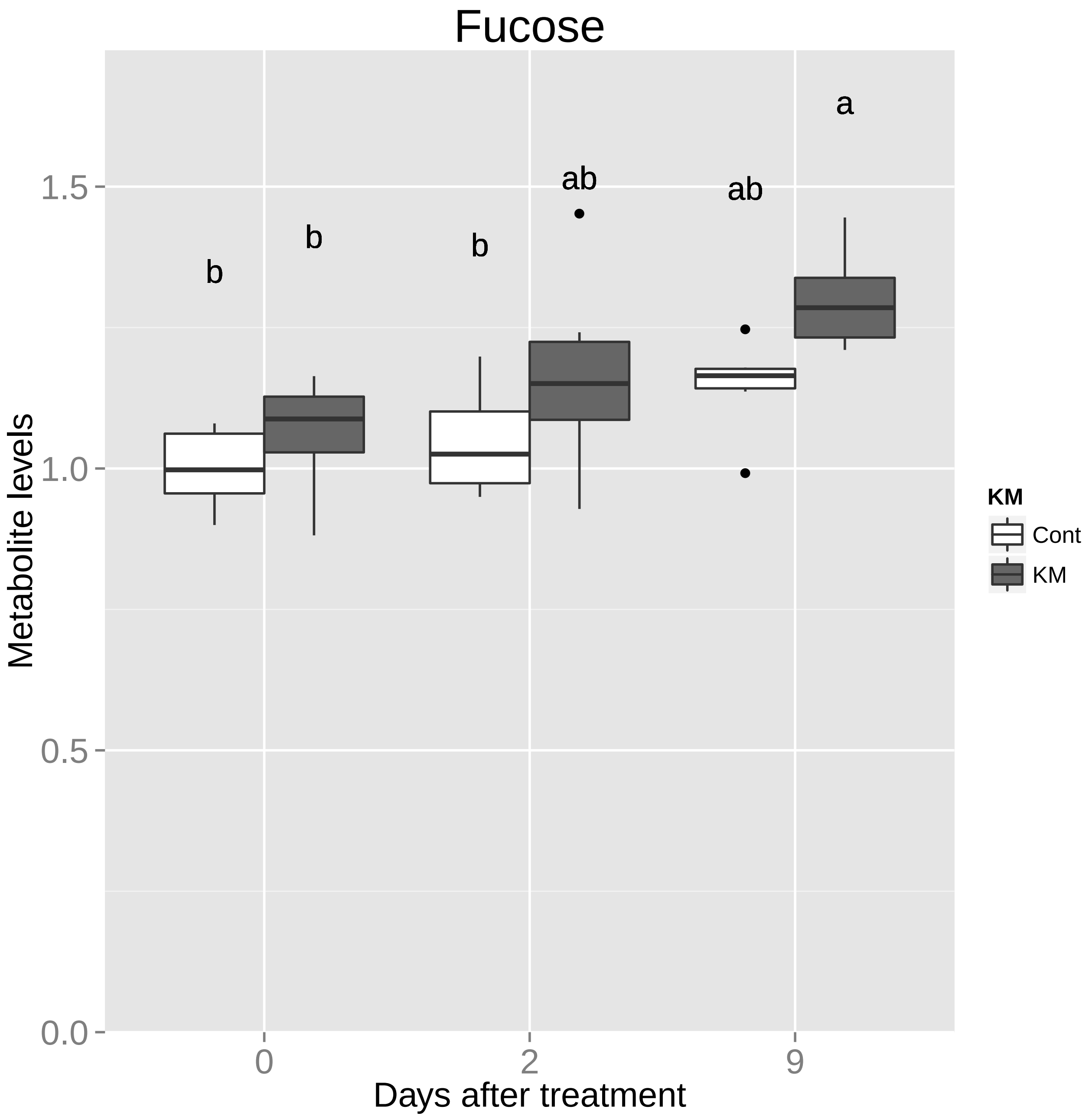
Fucose
Metabolite levels
1.5
1.0
0.5
0.0
b
b
b
ab
ab
a
0
2
9
Days after treatment
KM
Cont
KM

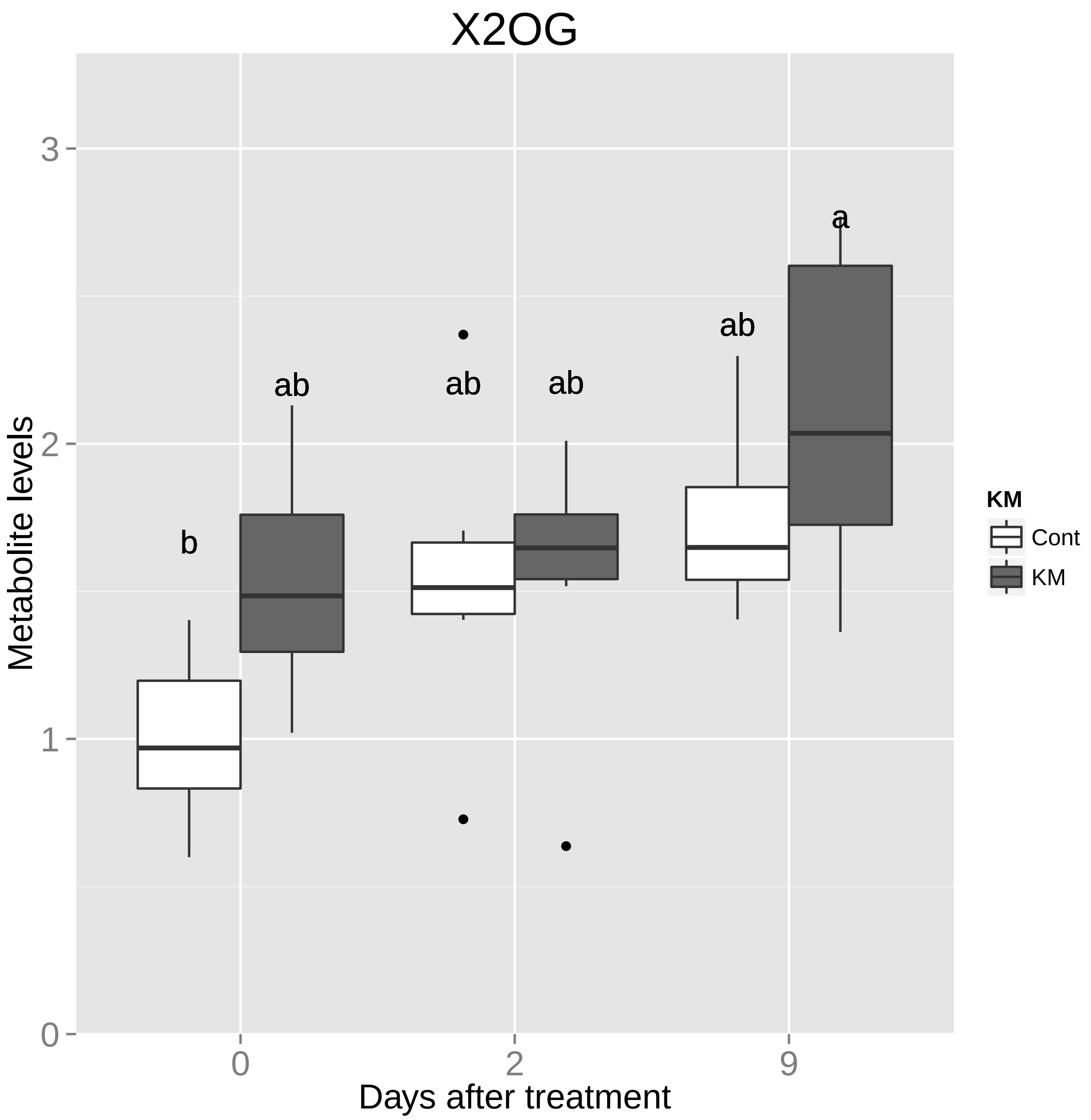
X2OG
Metabolite levels
Days after treatment
0
1
2
3
0
2
9
b
ab
ab
ab
ab
a
KM
Cont
KM

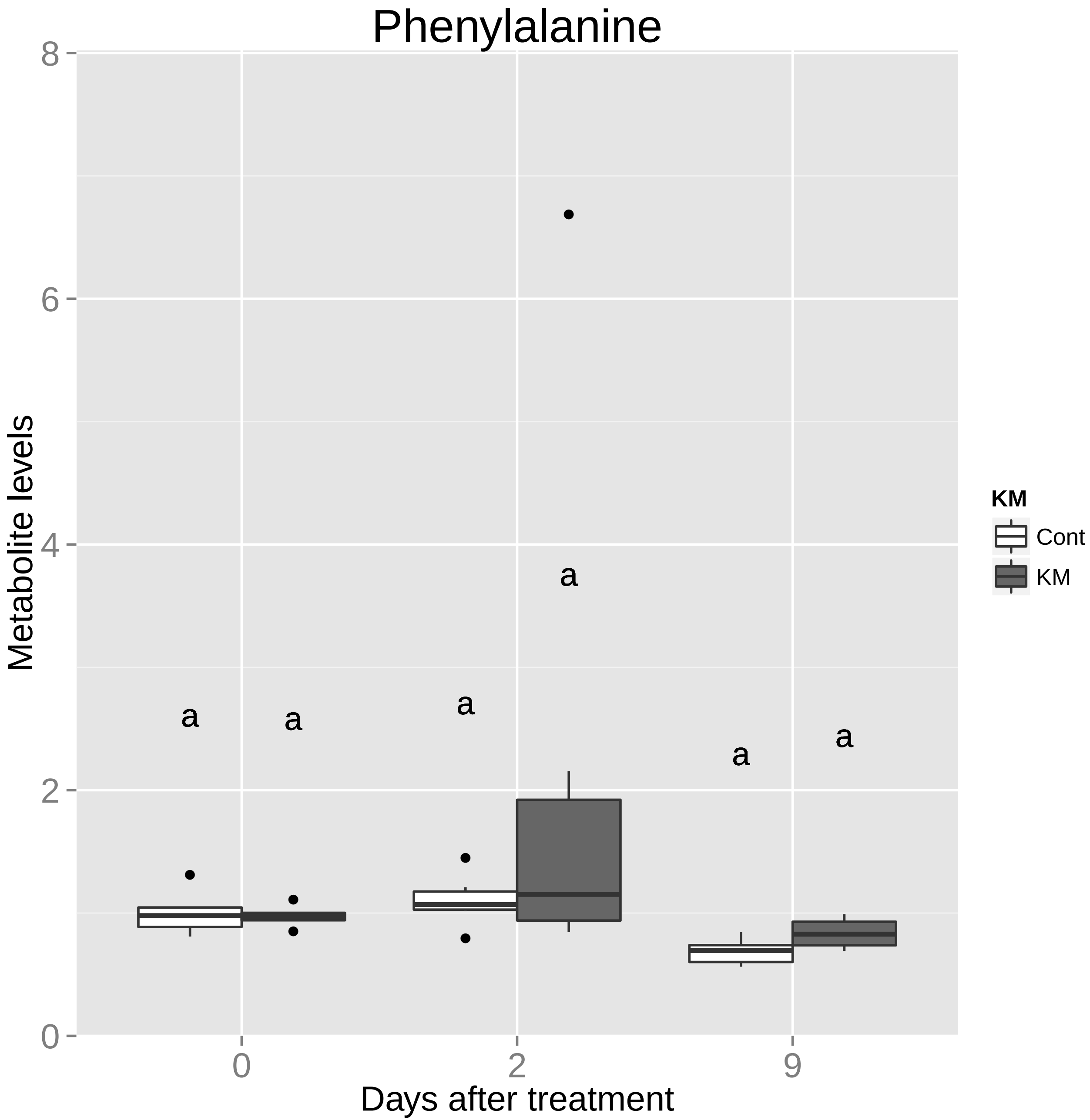
Phenylalanine
Metabolite levels
Days after treatment
0
2
4
6
8
0
2
9
a
a
a
a
a
a
KM
Cont
KM

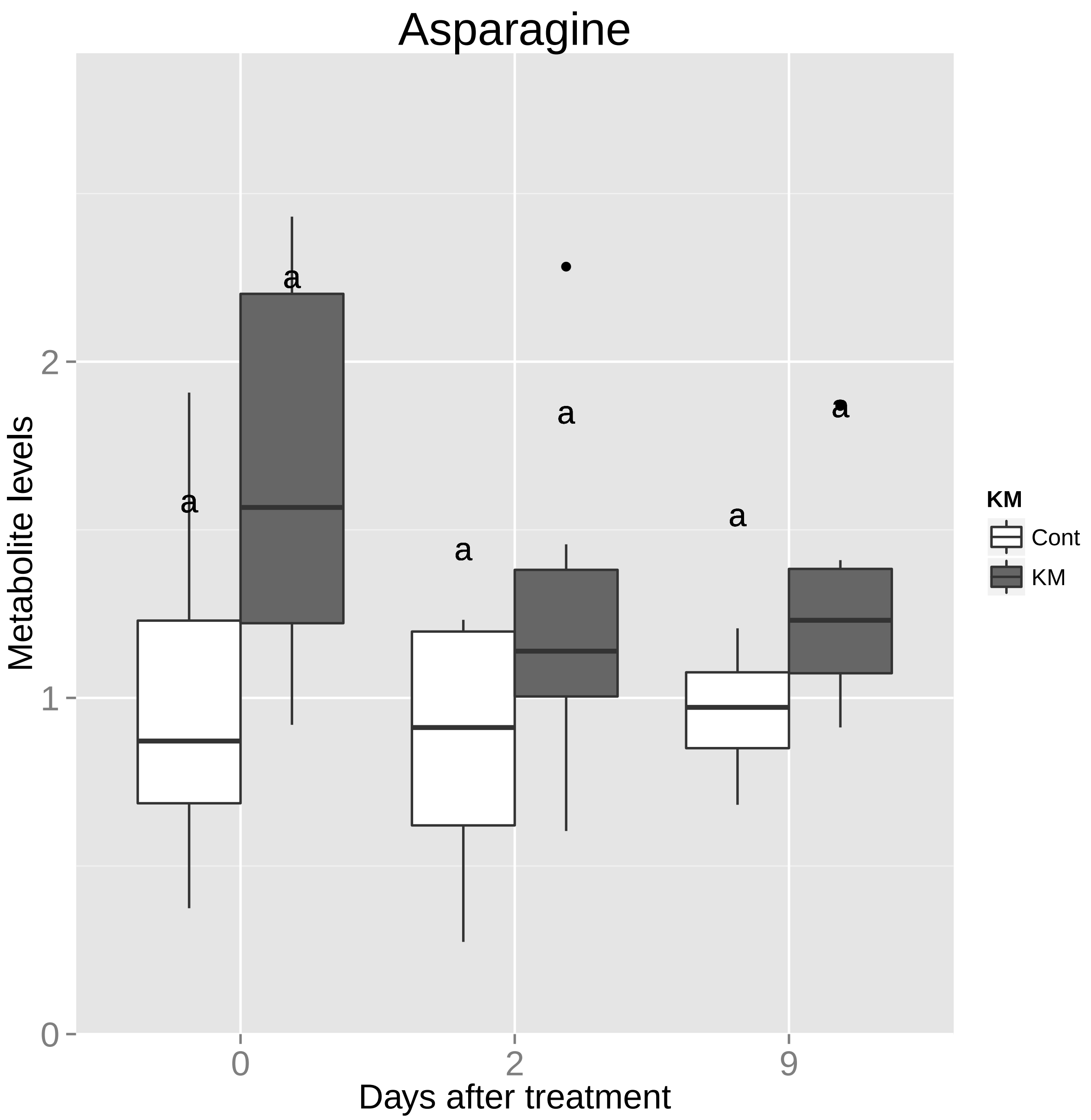

Asparagine
Metabolite levels
Days after treatment
0
1
2
0
2
9
a
a
a
a
a
a
KM
Cont
KM

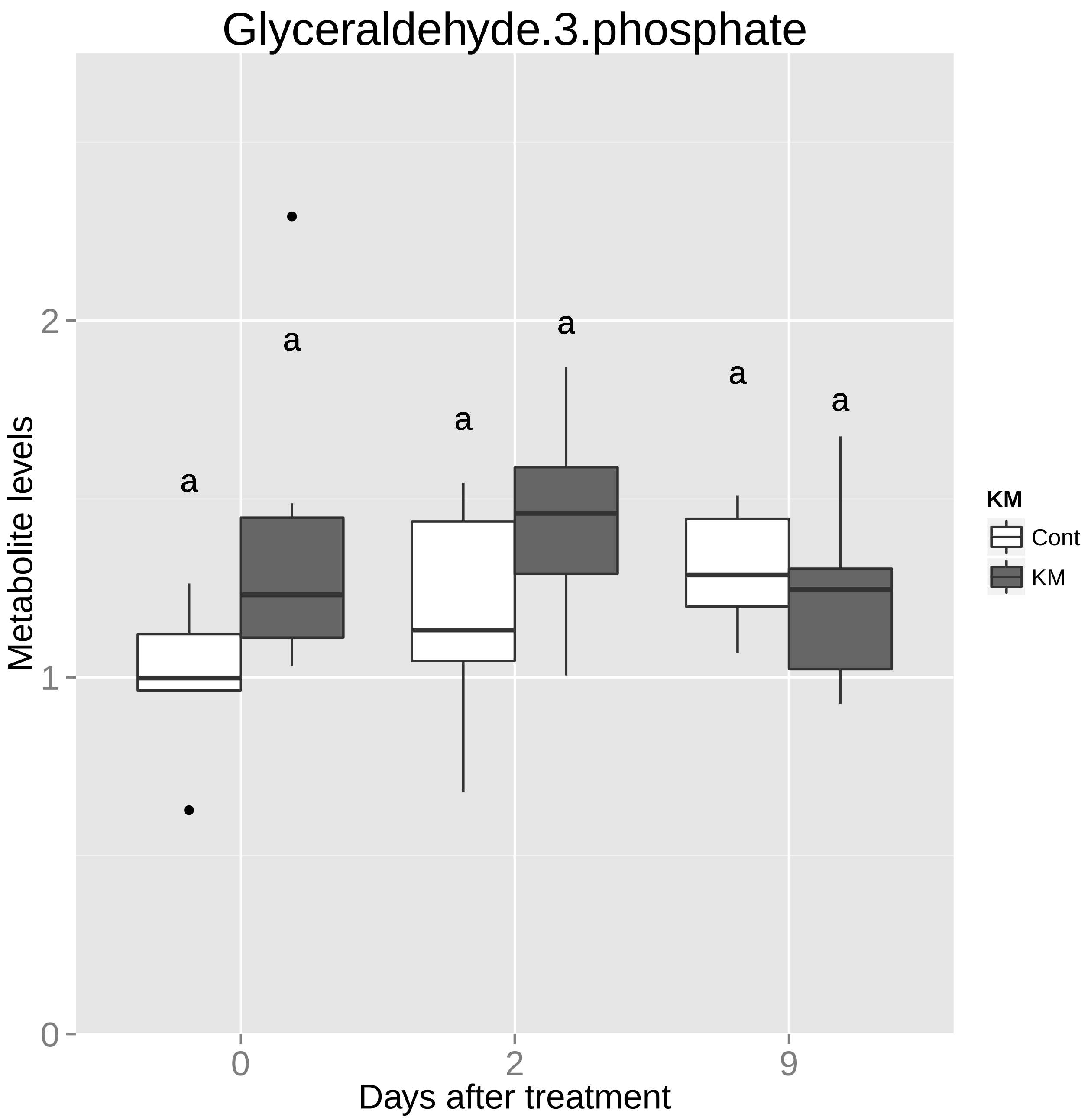

Glyceraldehyde.3.phosphate
Metabolite levels
0
1
2
a
a
a
a
a
a
0
2
9
Days after treatment
KM
Cont
KM

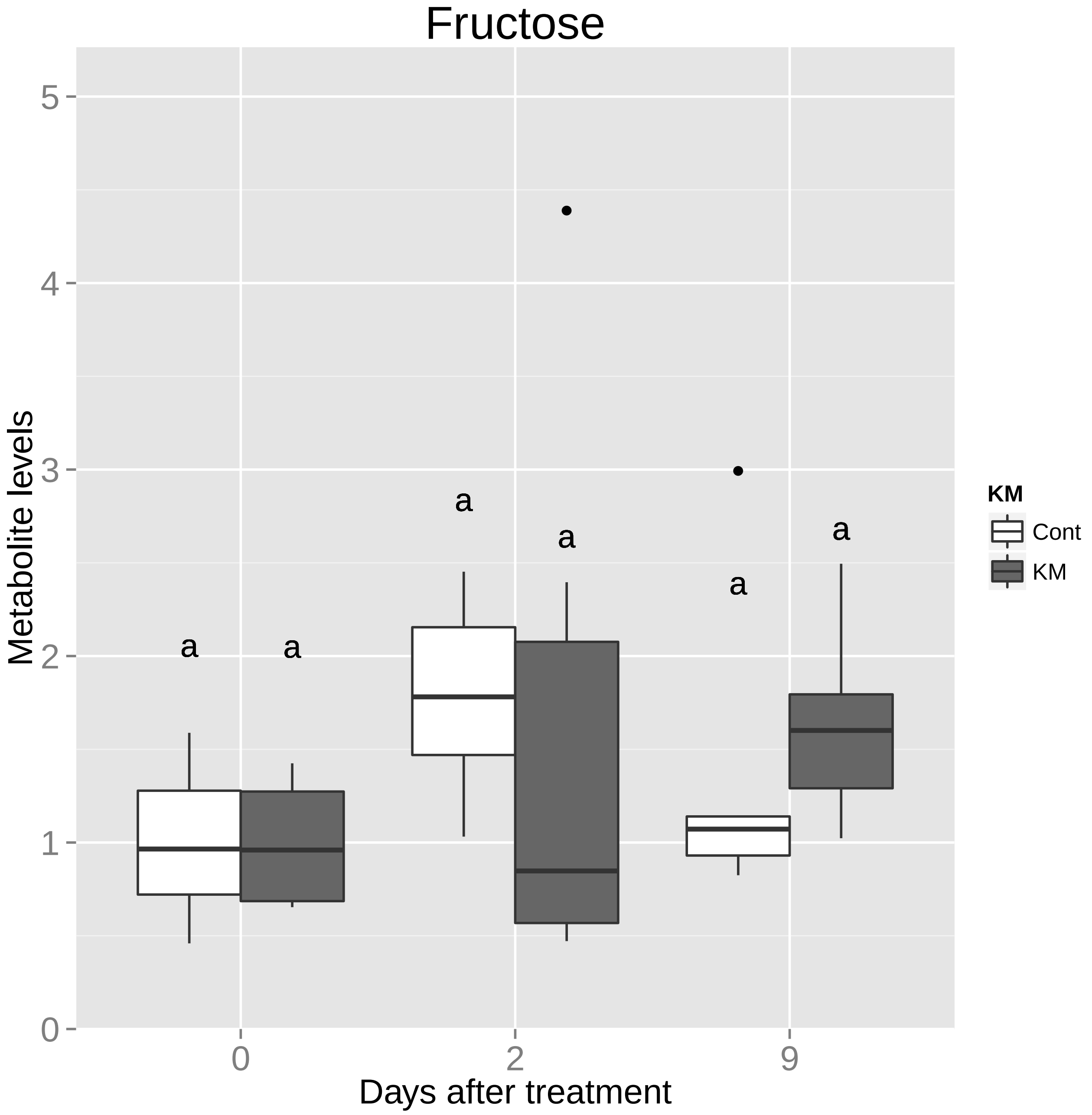
Fructose
Metabolite levels
5
4
3
2
1
0
a
a
a
a
a
a
0
2
9
Days after treatment
KM
Cont
KM

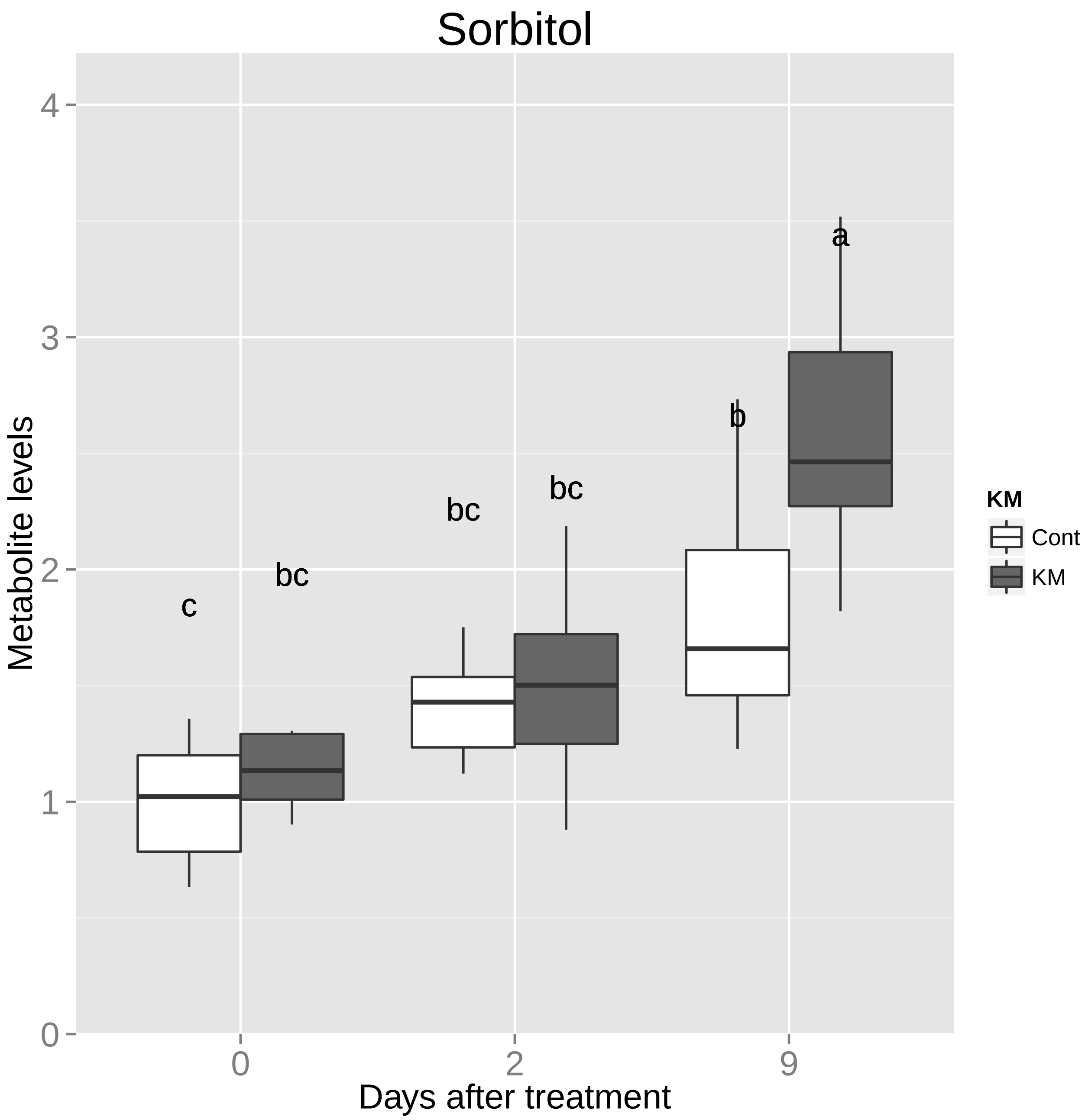
Sorbitol
Metabolite levels
Days after treatment
0
1
2
3
4
0
2
9
c
bc
bc
bc
b
a
KM
Cont
KM

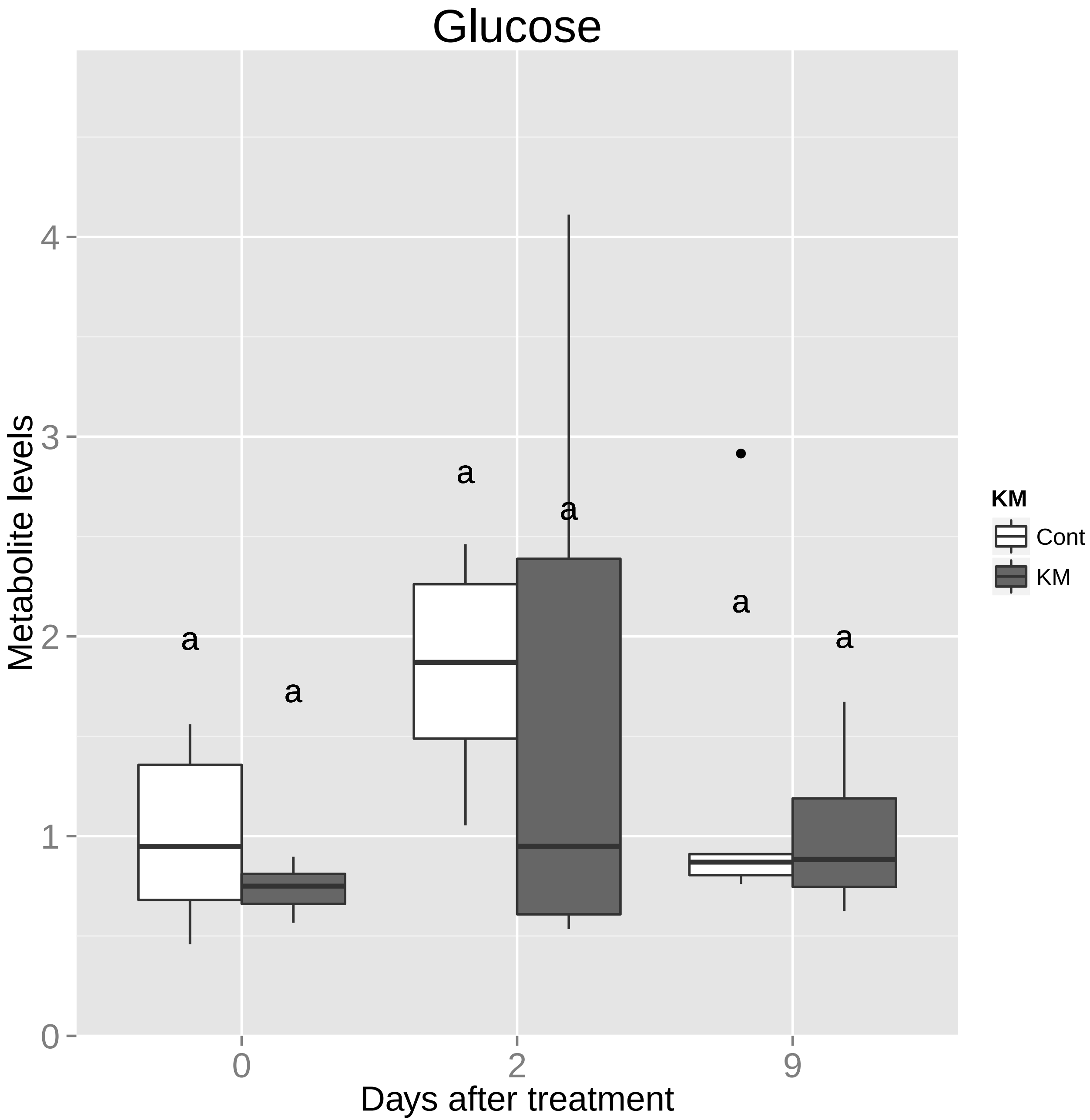
Glucose
Metabolite levels
Days after treatment
0
1
2
3
4
0
2
9
a
a
a
a
a
a
KM
Cont
KM

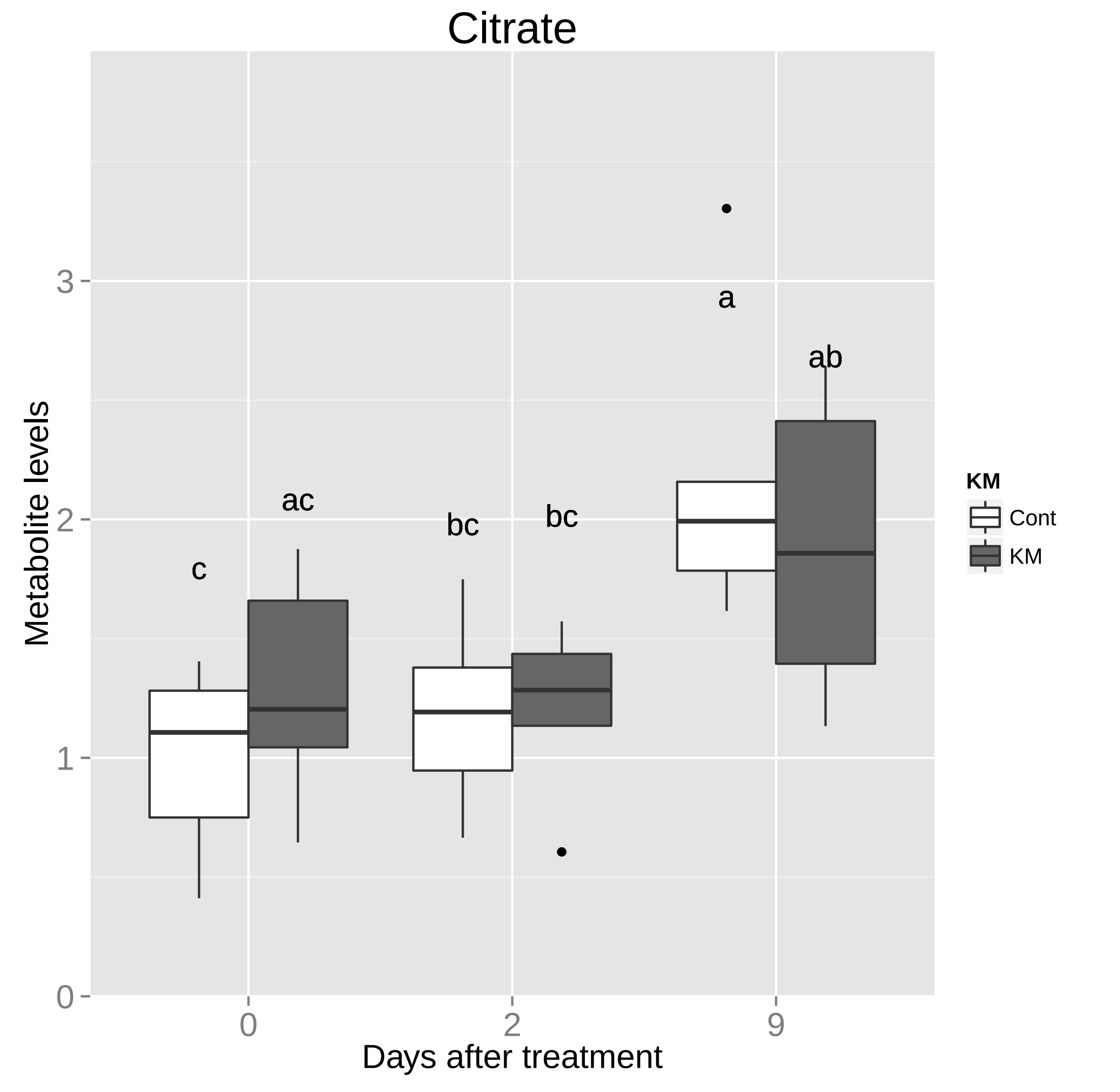

Citrate
Metabolite levels
Days after treatment
0
1
2
3
0
2
9
c
ac
bc
bc
a
ab
KM
Cont
KM

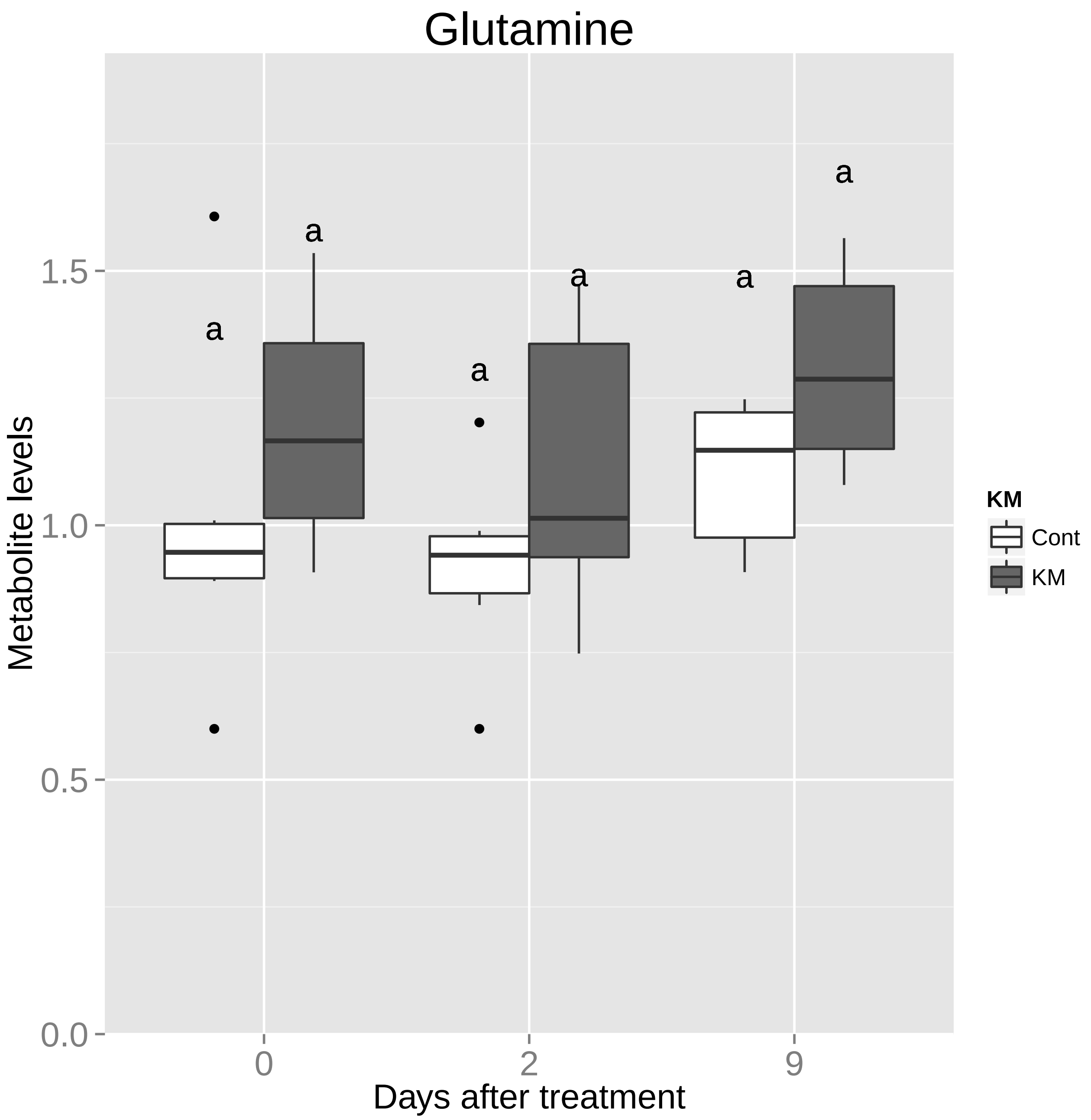

Glutamine
a
a
a
a
a
a
1.5
1.0
0.5
0.0
0
2
9
Days after treatment
Metabolite levels
KM
Cont
KM

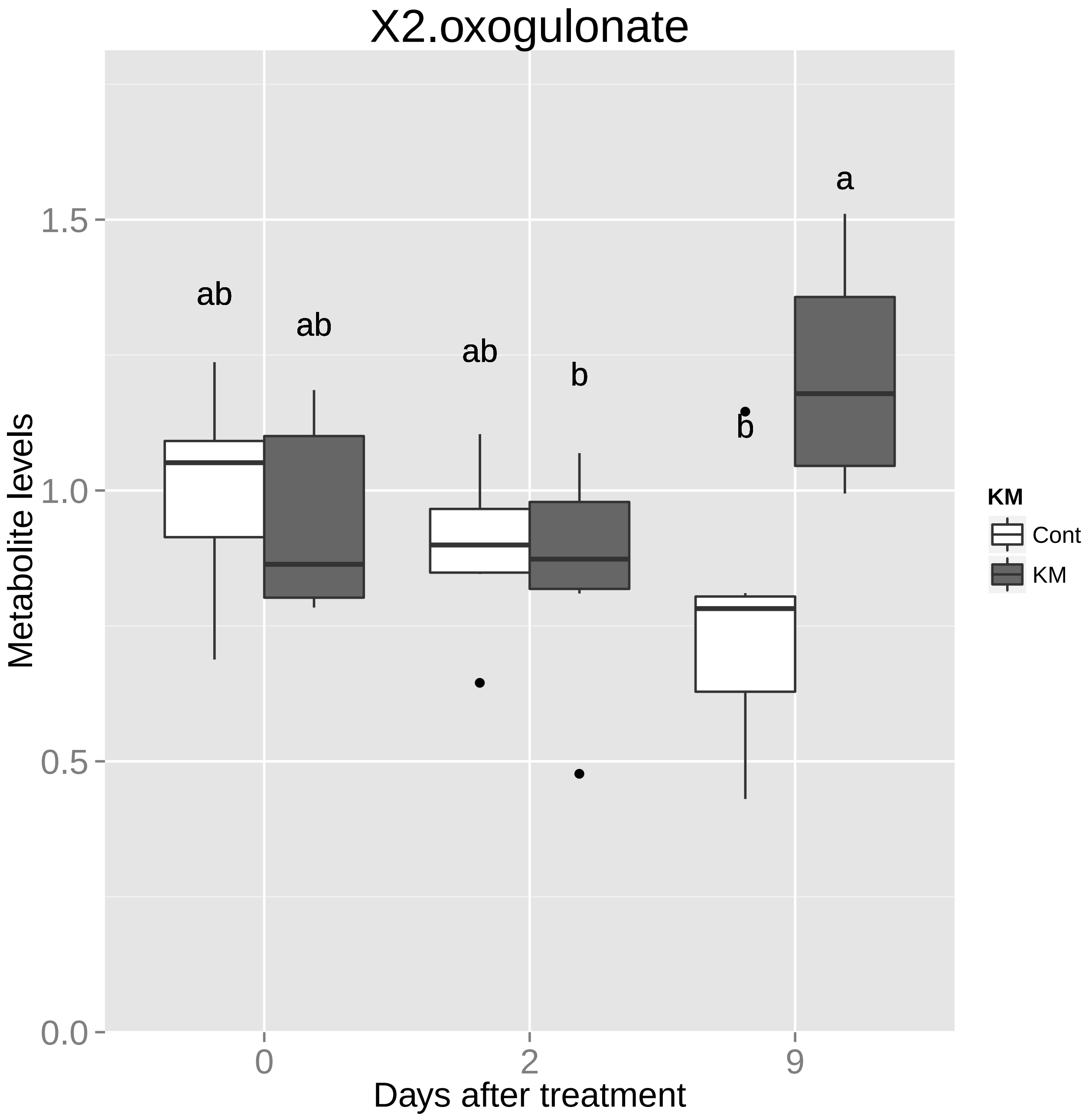
X2.oxogulonate
Metabolite levels
Days after treatment
0.0
0.5
1.0
1.5
0
2
9
ab
ab
ab
b
b
a
KM
Cont
KM

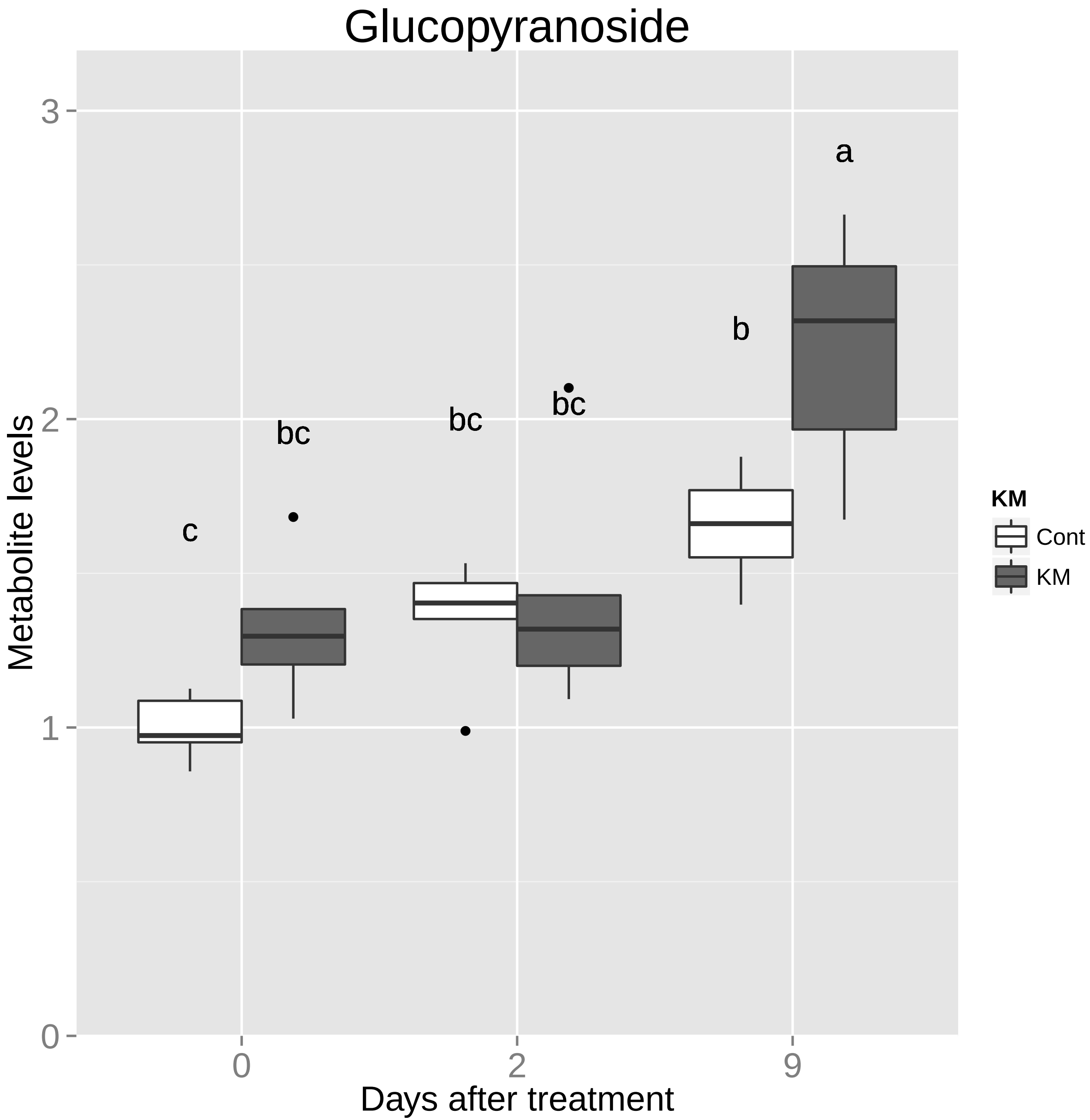

Glucopyranoside
Metabolite levels
Days after treatment
0
1
2
3
0
2
9
c
bc
bc
bc
b
a
KM
Cont
KM

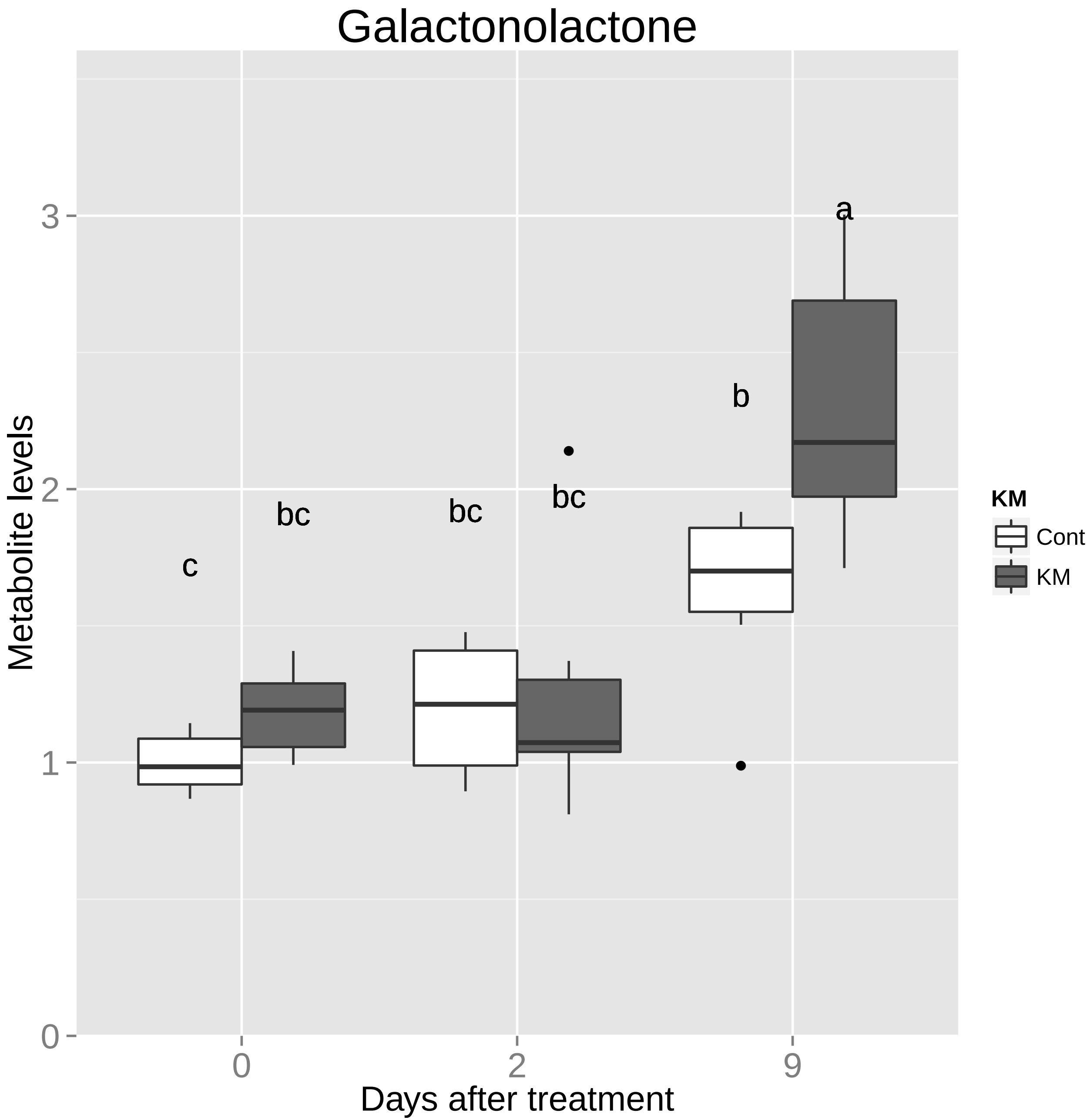
Galactonolactone
Metabolite levels
Days after treatment
0
1
2
3
0
2
9
c
bc
bc
bc
b
a
KM
Cont
KM

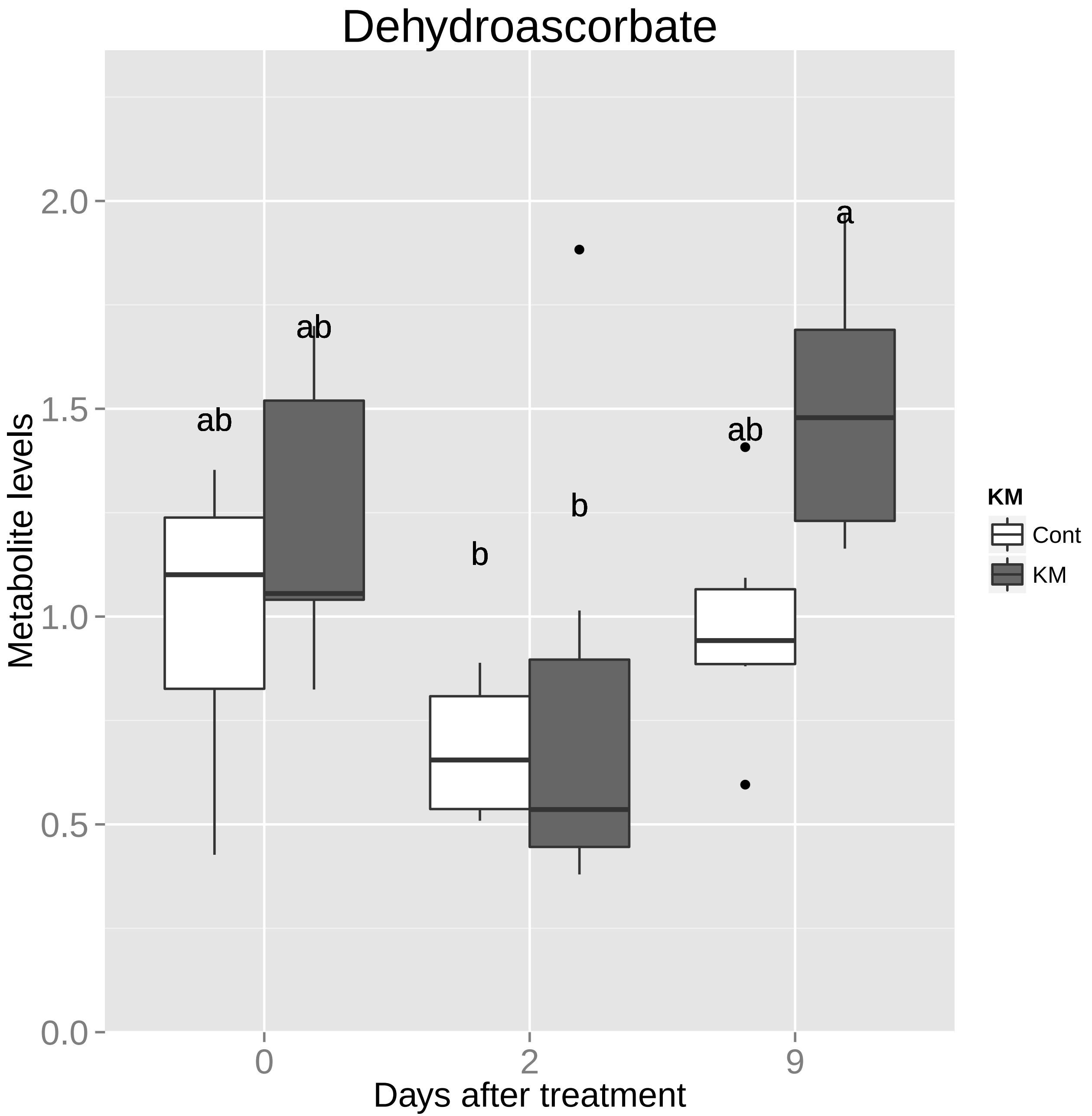

Dehydroascorbate
Metabolite levels
Days after treatment
0.0
0.5
1.0
1.5
2.0
0
2
9
ab
ab
b
b
ab
a
KM
Cont
KM

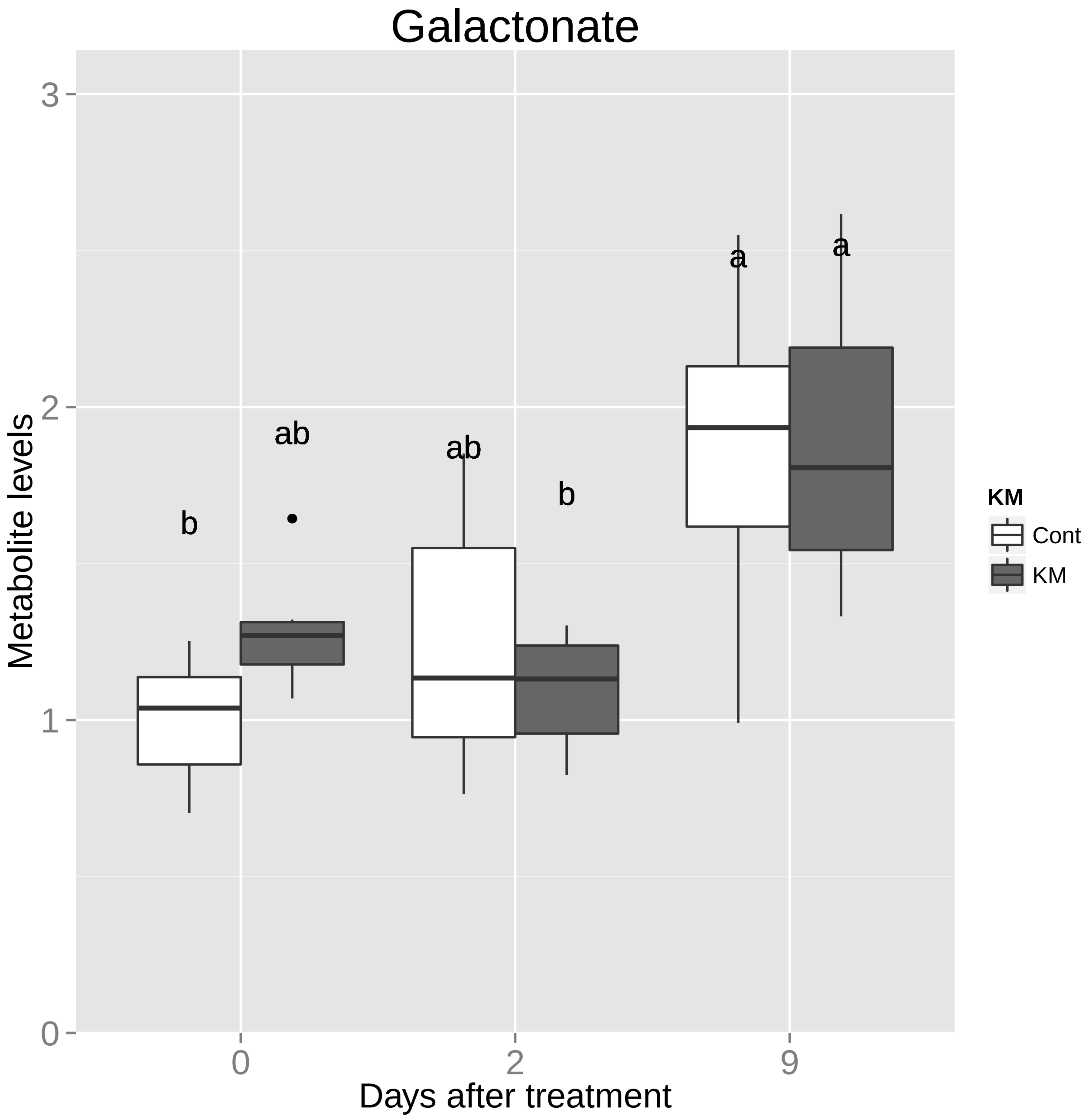
Galactonate
Metabolite levels
Days after treatment
0
1
2
3
0
2
9
b
ab
ab
b
a
a
KM
Cont
KM

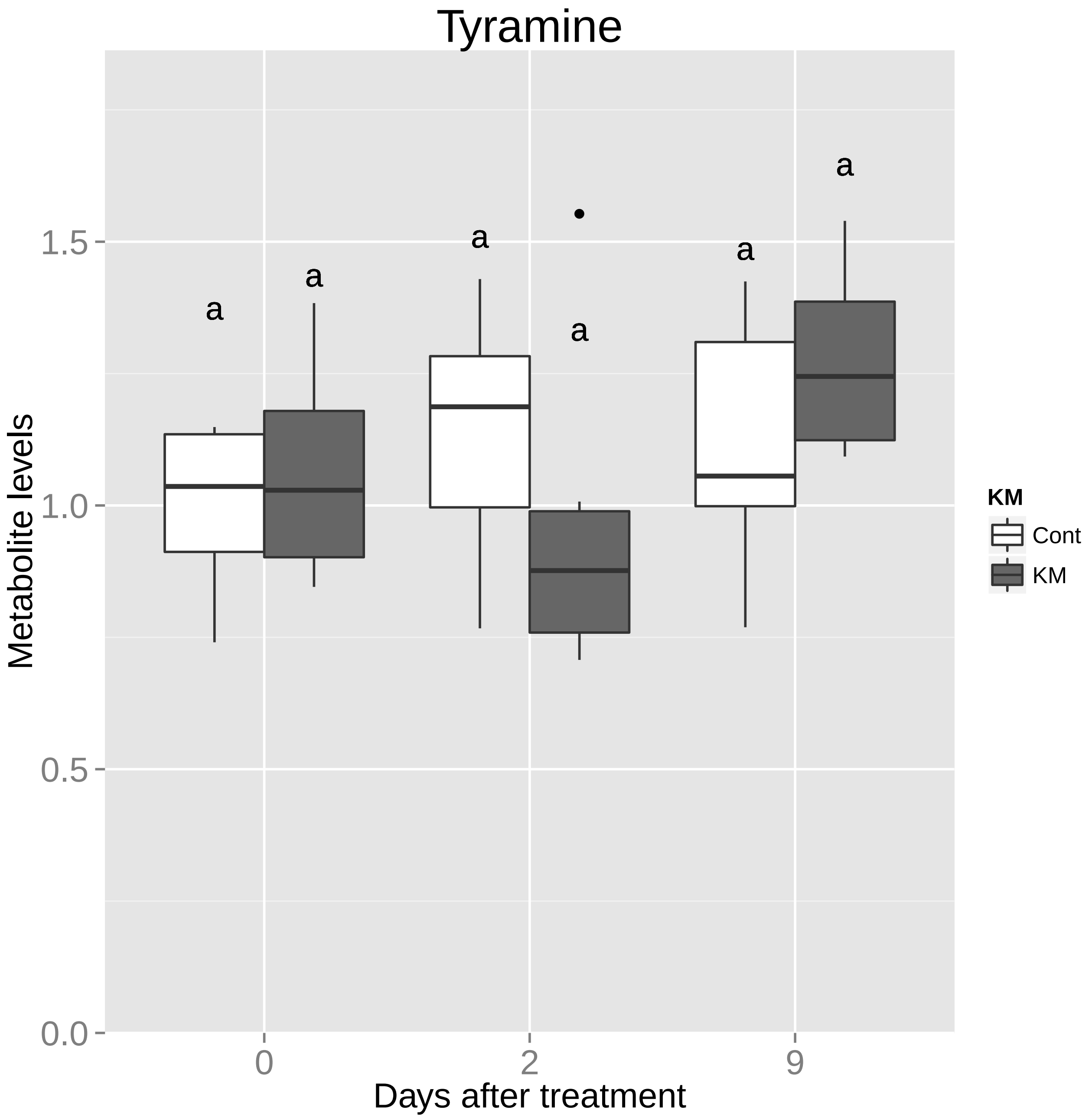
Tyramine
Metabolite levels
Days after treatment
0.0
0.5
1.0
1.5
0
2
9
a
a
a
a
a
a
KM
Cont
KM

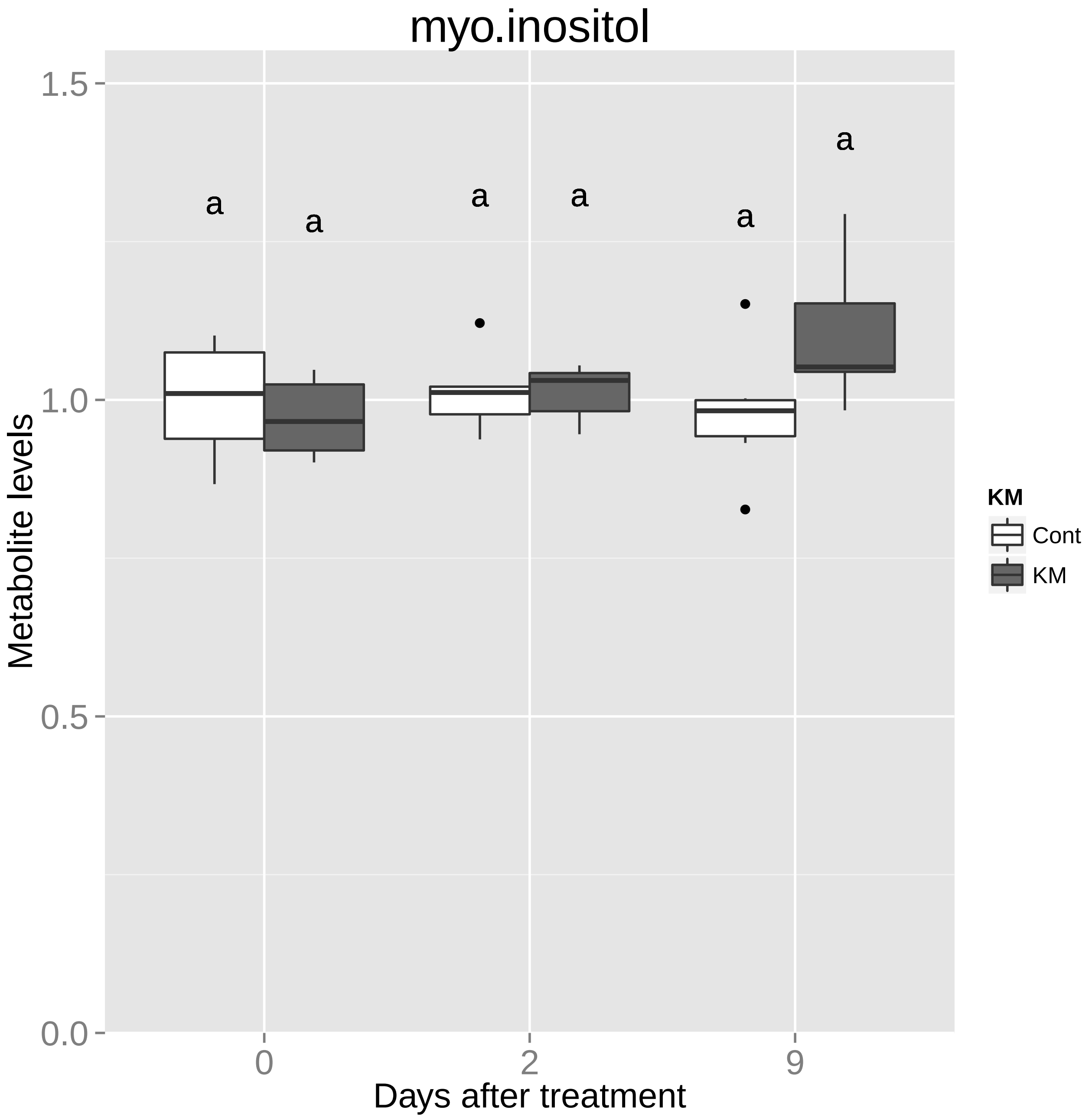

myo.inositol
Metabolite levels
1.5
1.0
0.5
0.0
a
a
a
a
a
a
0
2
9
Days after treatment
KM
Cont
KM

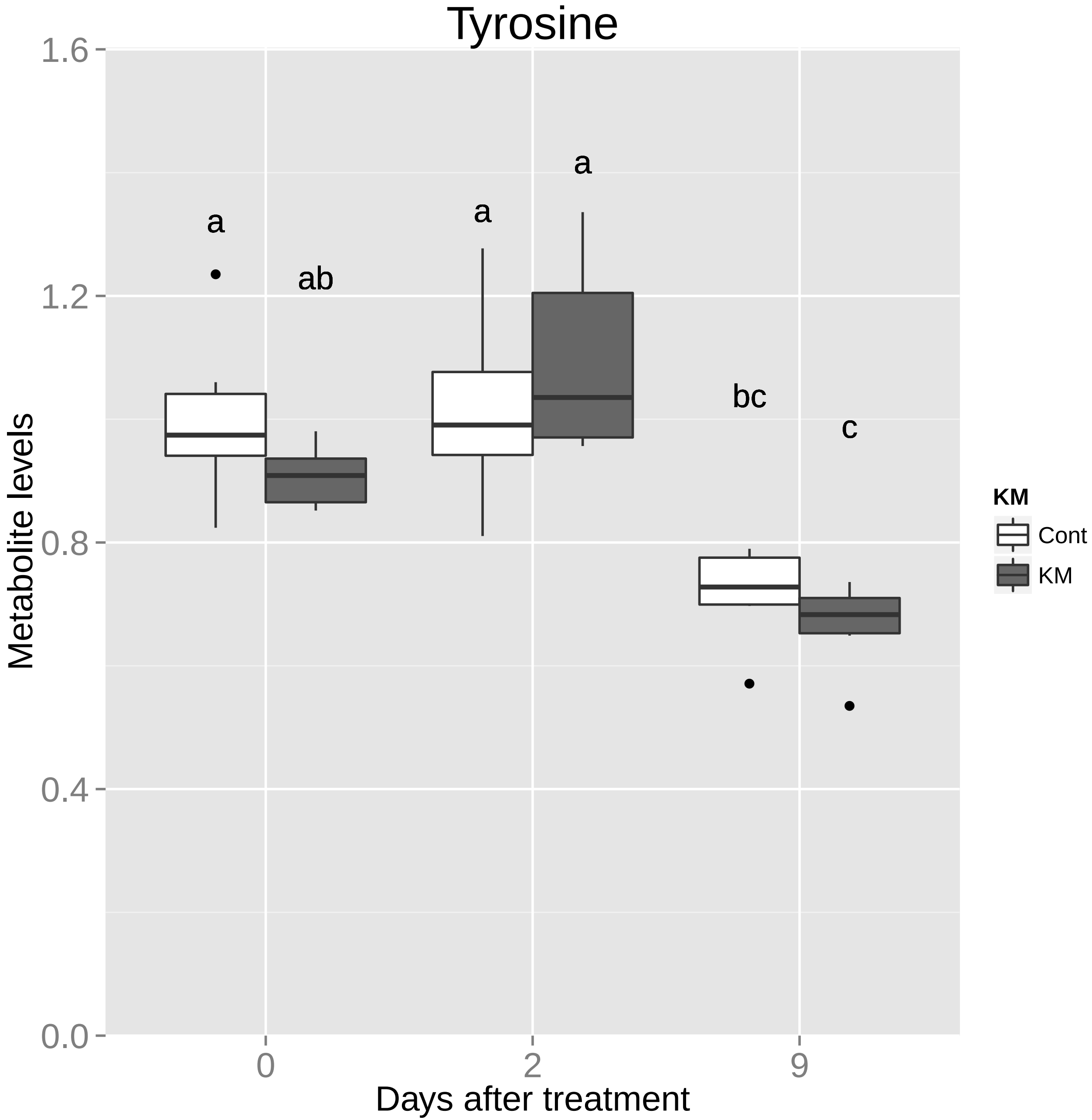

Tyrosine
1.6
1.2
0.8
0.4
0.0
Metabolite levels
a
ab
a
a
bc
c
0
2
9
Days after treatment
KM
Cont
KM

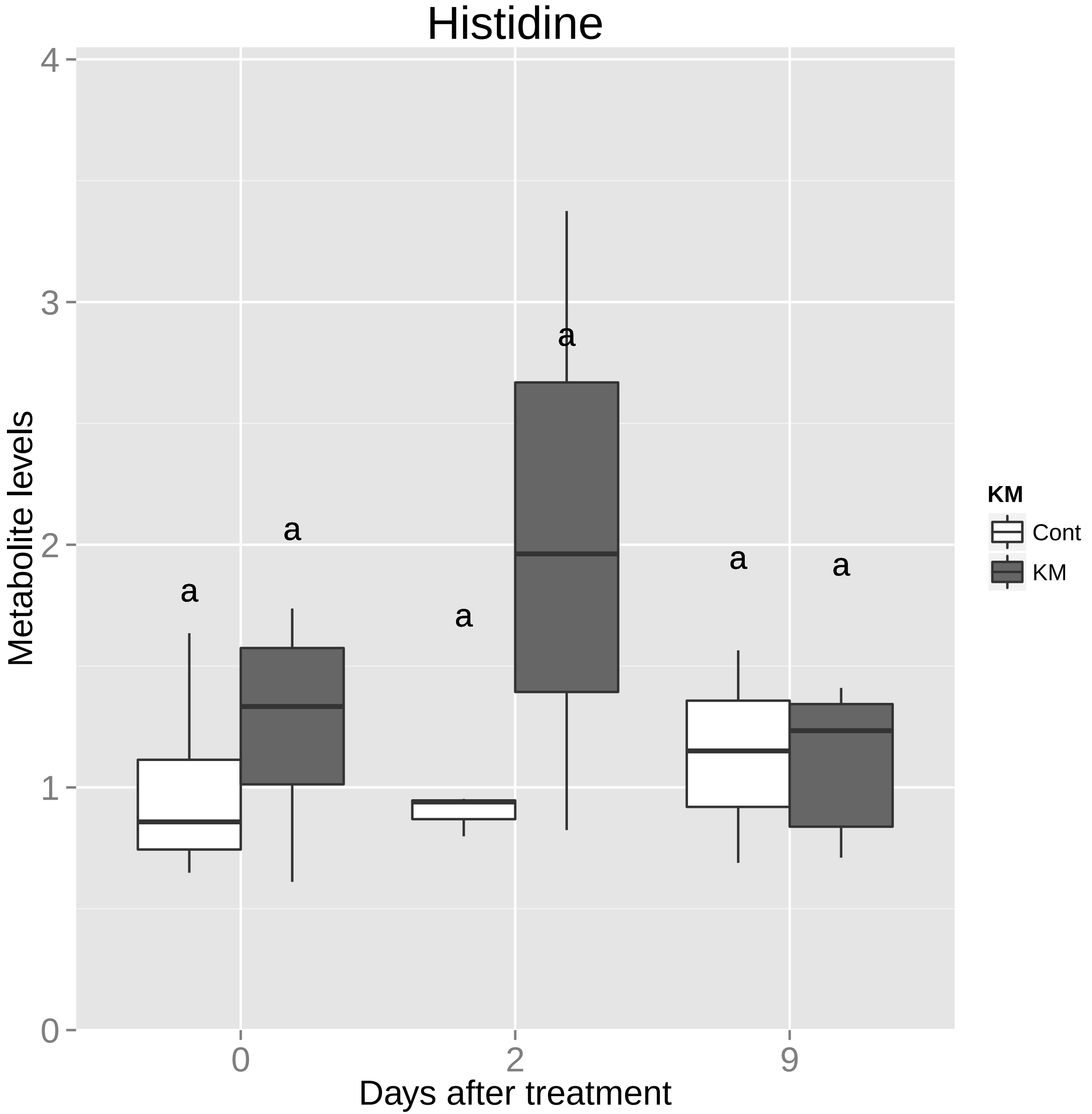

Histidine
Metabolite levels
Days after treatment
0
1
2
3
4
0
2
9
a
a
a
a
a
a
KM
Cont
KM

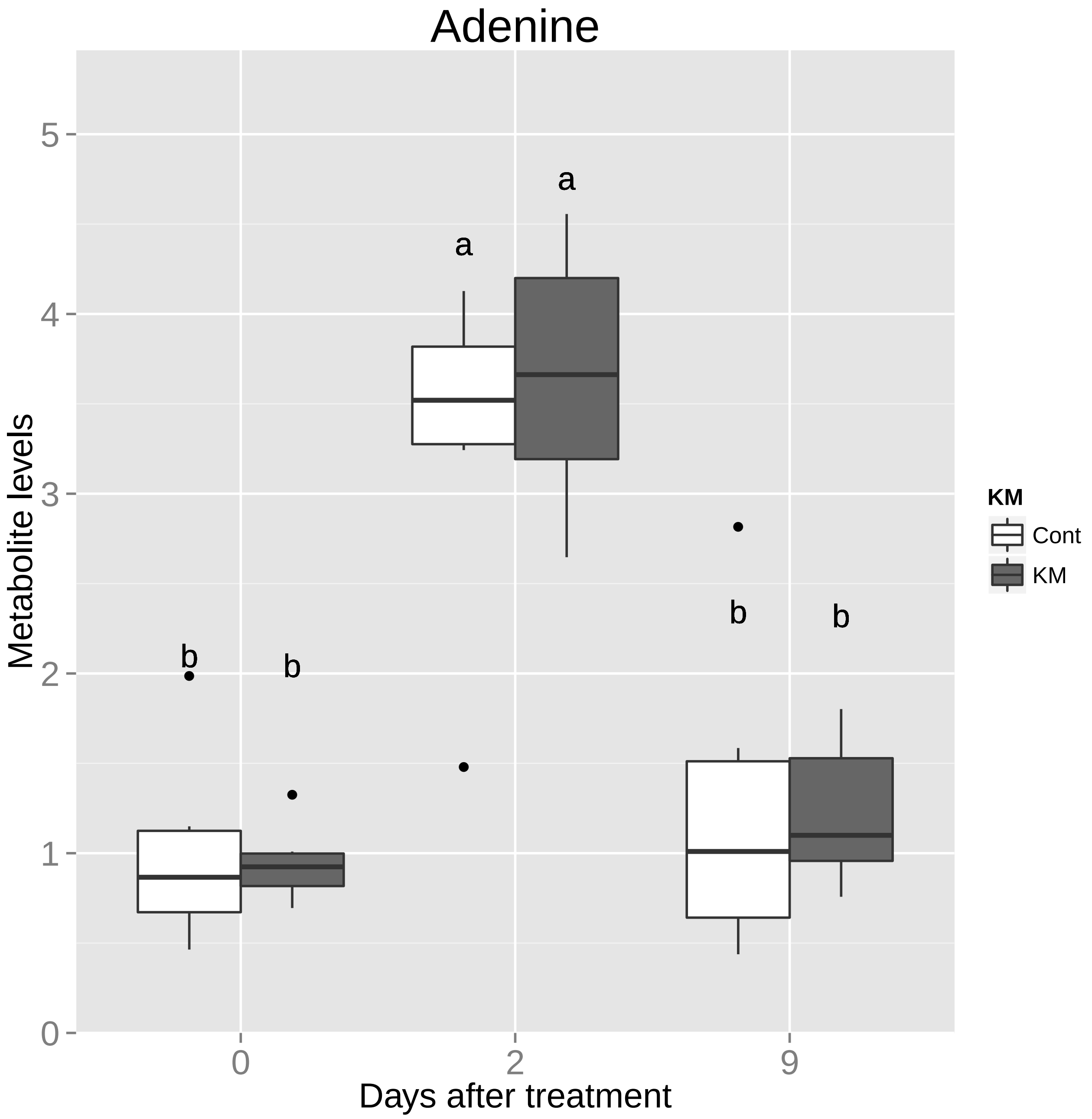
Adenine
Metabolite levels
Days after treatment
0
1
2
3
4
5
0
2
9
a
a
b
b
b
b
KM
Cont
KM

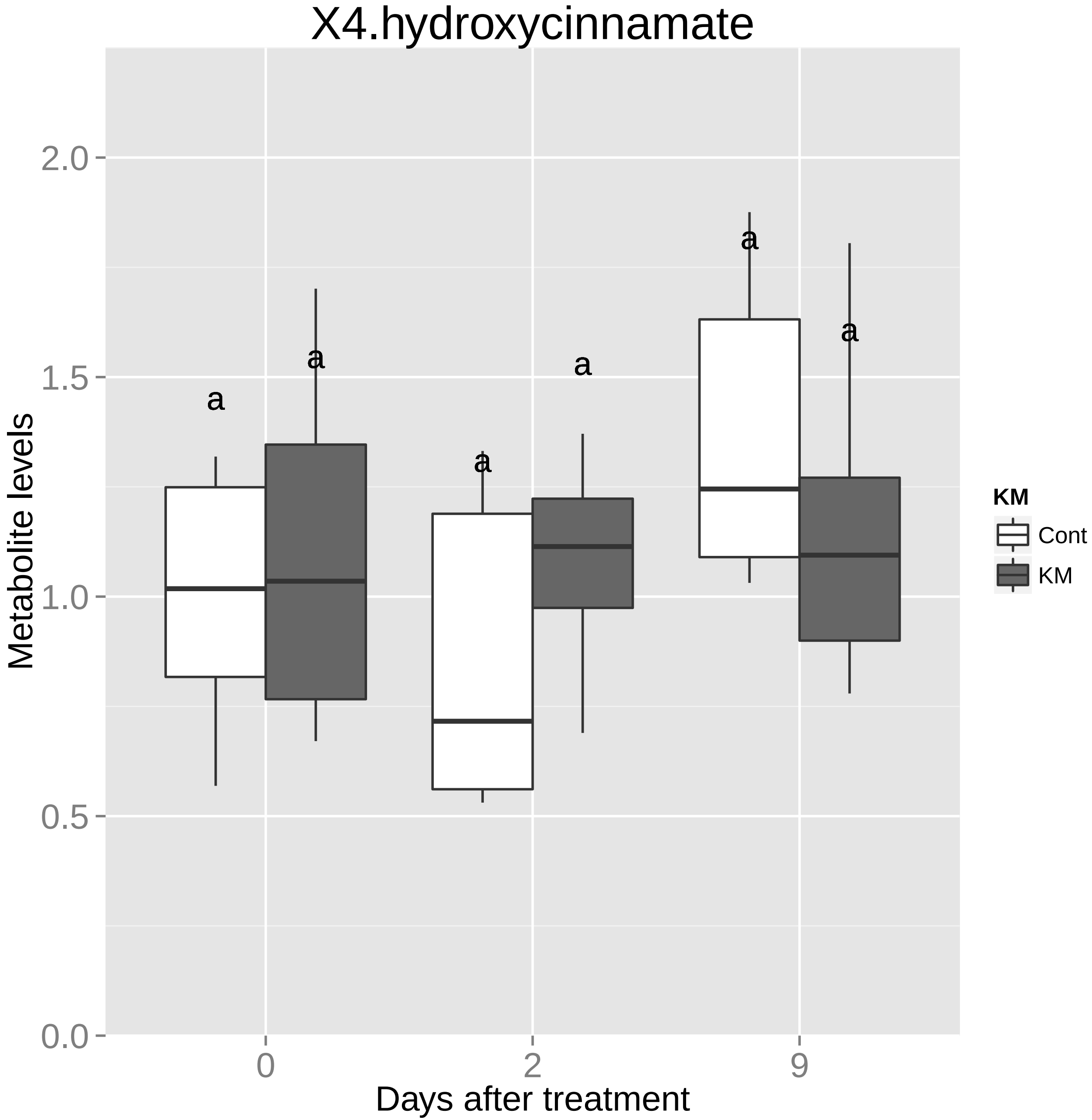

X4.hydroxycinnamate
2.0
1.5
1.0
0.5
0.0
Metabolite levels
a
a
a
a
a
a
0
2
9
Days after treatment
KM
Cont
KM

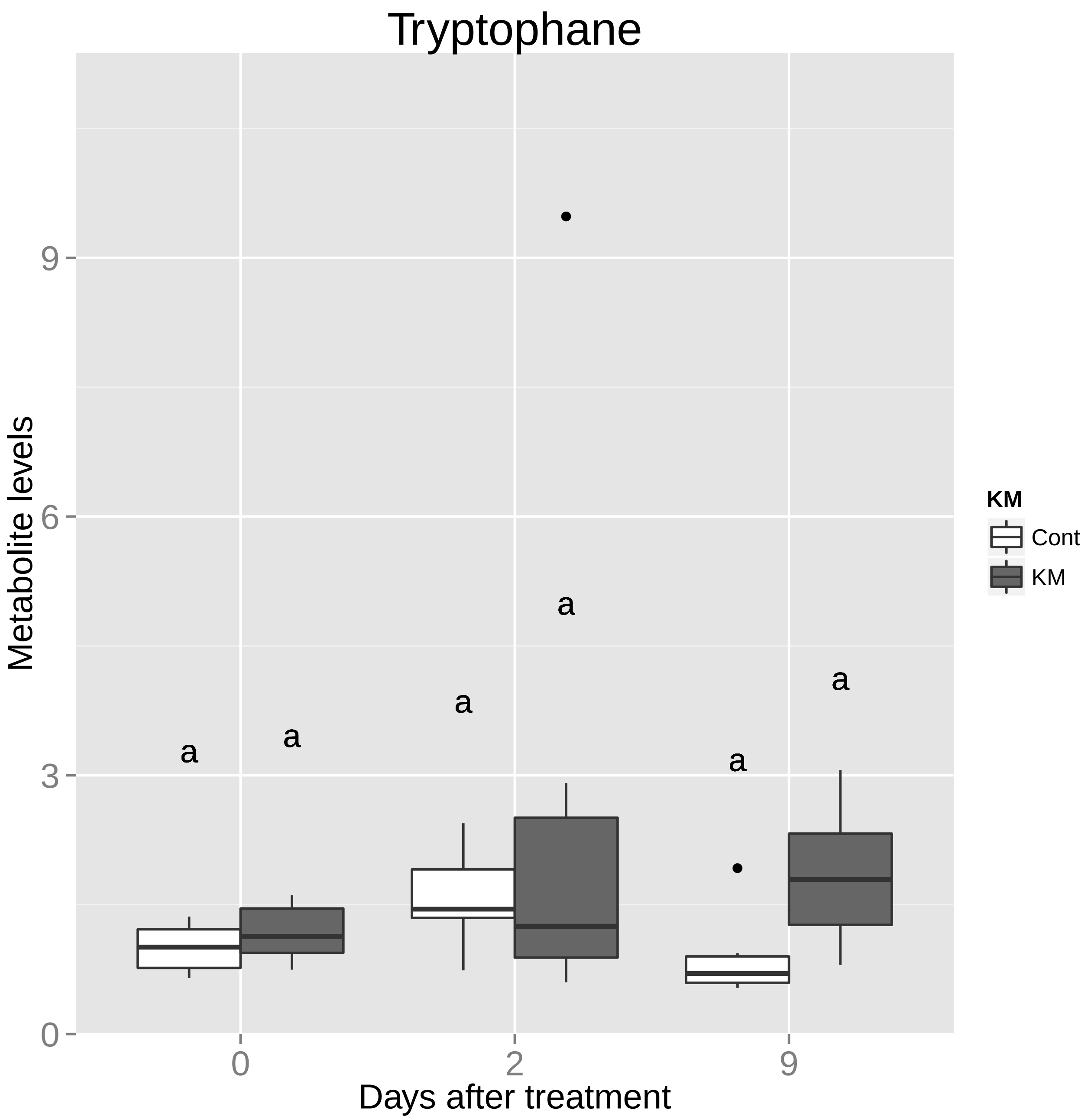
Tryptophane
Metabolite levels
9
6
3
0
a
a
a
a
a
a
0
2
9
Days after treatment
KM
Cont
KM

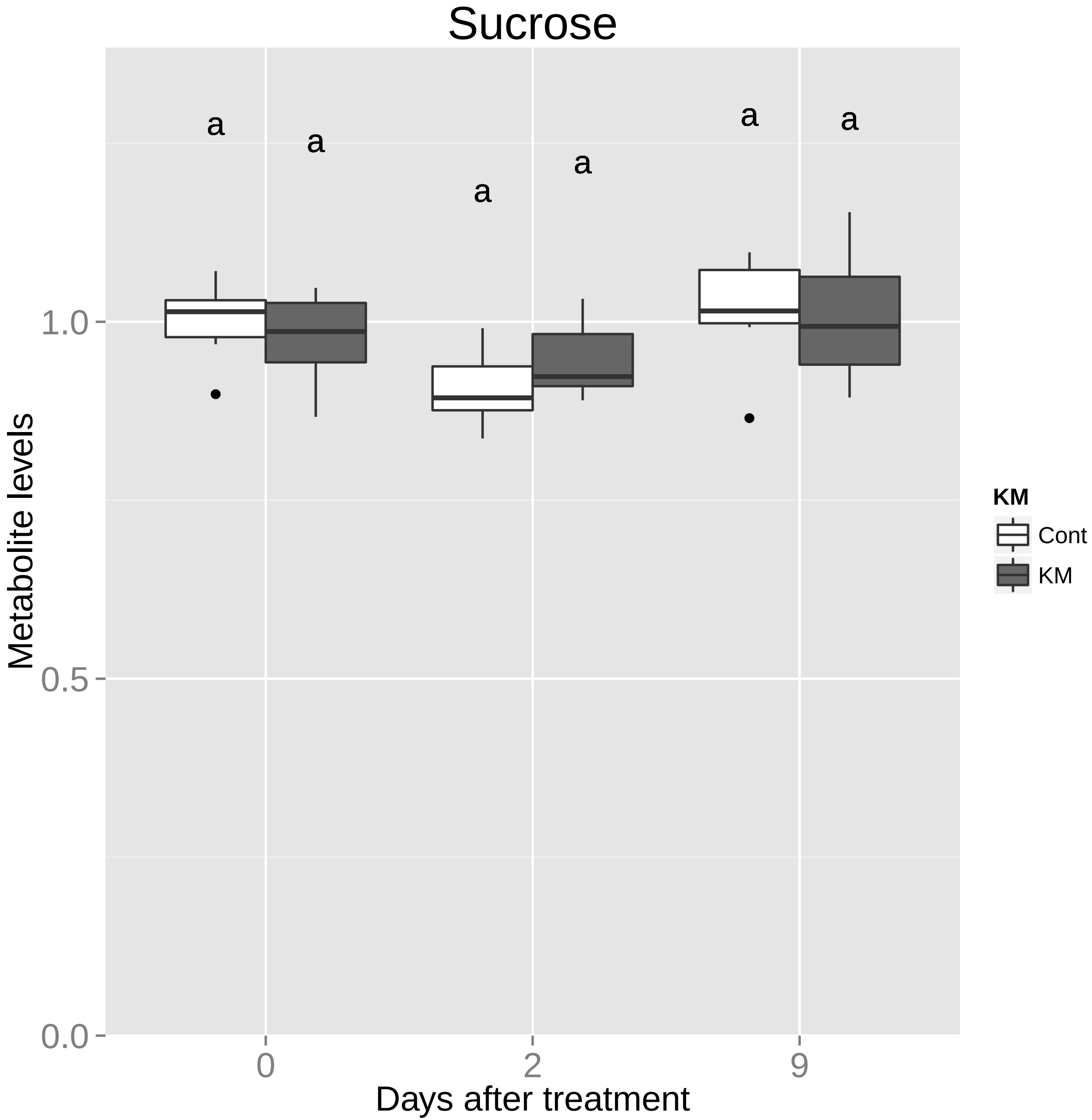
Sucrose
a
a
a
a
a
a
1.0
0.5
0.0
Metabolite levels
0
2
9
Days after treatment
KM
Cont
KM

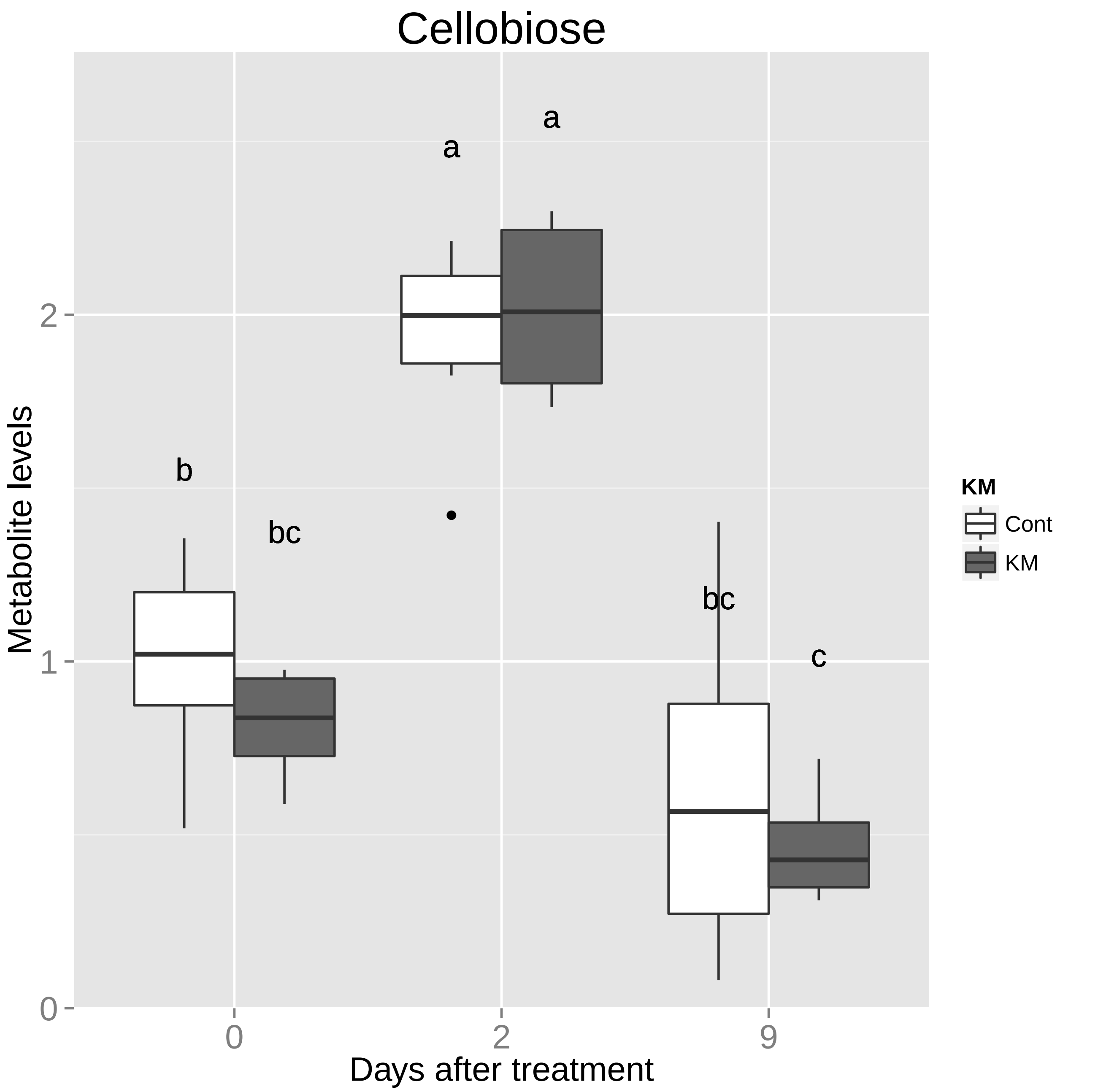
Cellobiose
Metabolite levels
Days after treatment
0
1
2
0
2
9
a
a
b
bc
bc
c
KM
Cont
KM

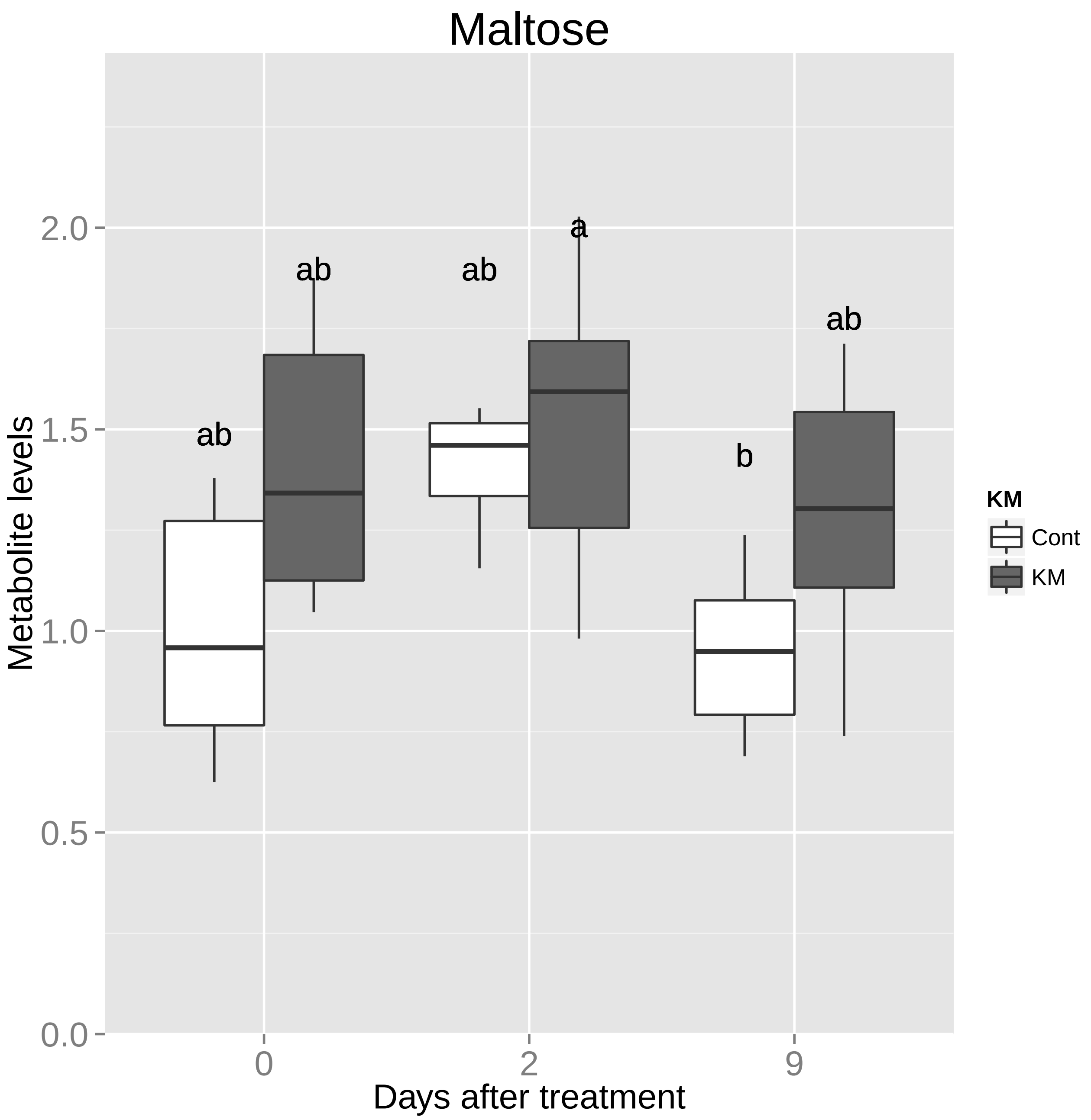
Maltose
Metabolite levels
0.0
0.5
1.0
1.5
2.0
ab
ab
ab
a
b
ab
0
2
9
Days after treatment
KM
Cont
KM

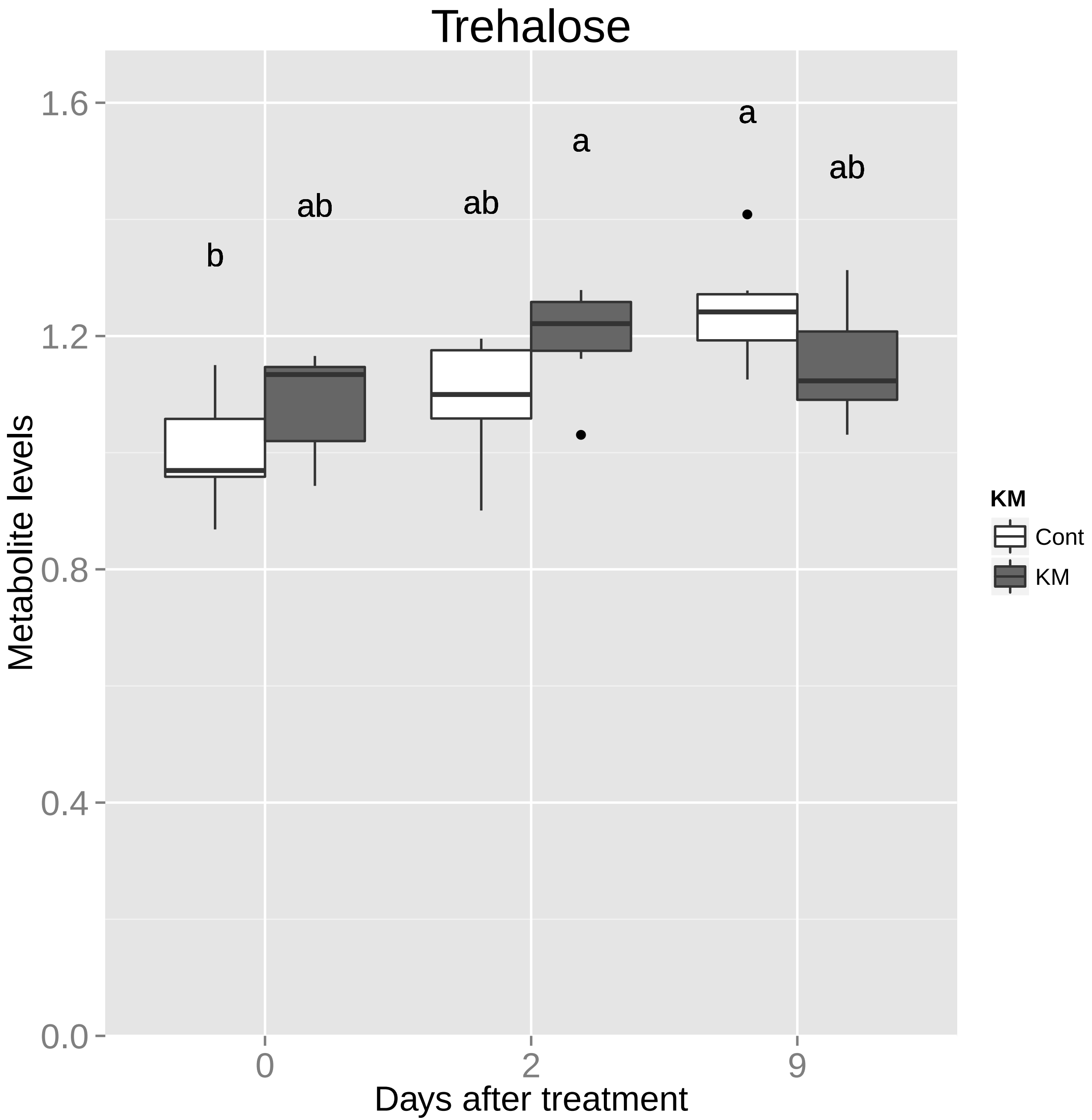
Trehalose
Metabolite levels
1.6
1.2
0.8
0.4
0.0
b
ab
ab
a
a
ab
0
2
9
Days after treatment
KM
Cont
KM

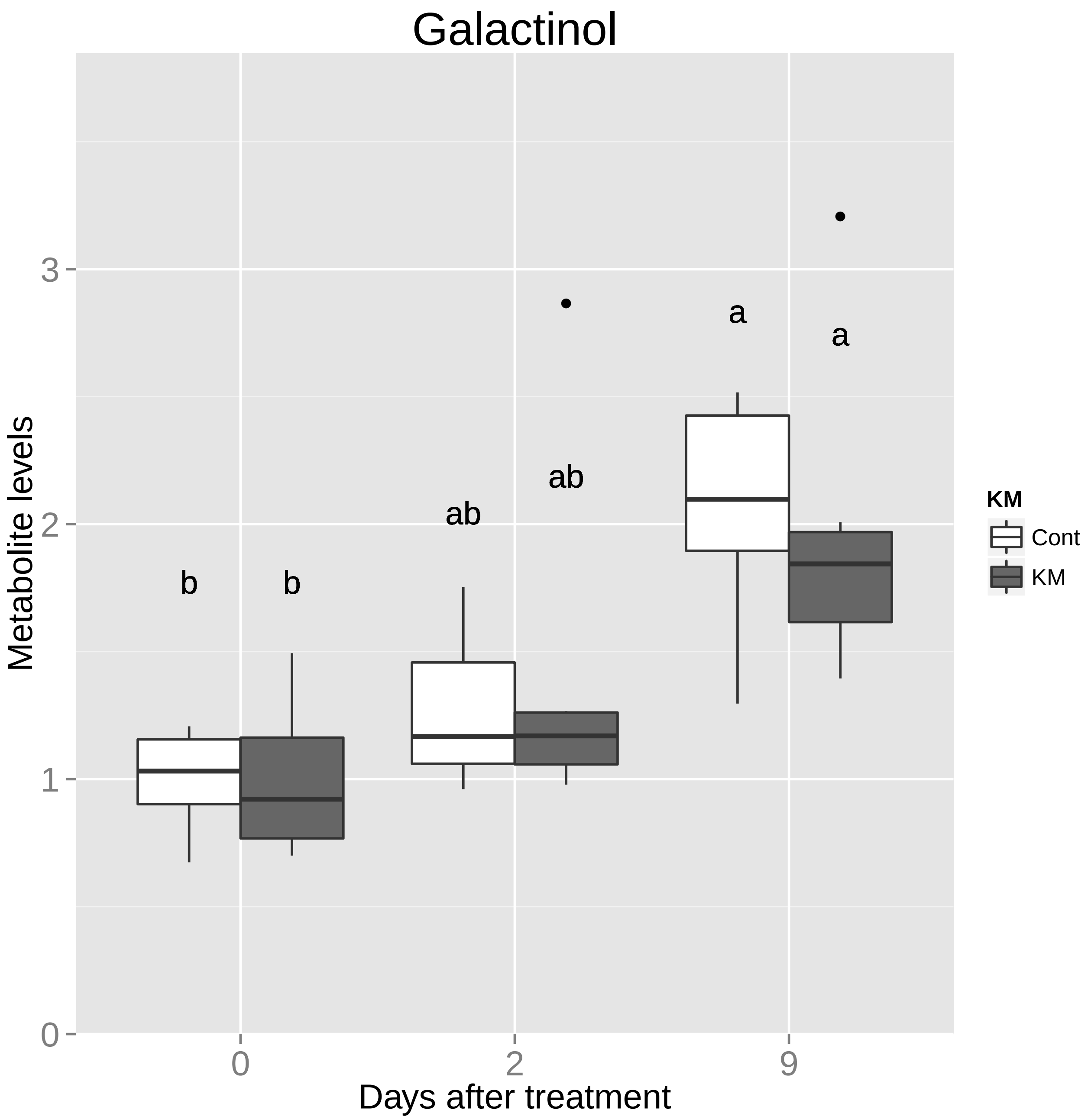

Galactinol
Metabolite levels
Days after treatment
0
1
2
3
0
2
9
b
b
ab
ab
a
a
KM
Cont
KM

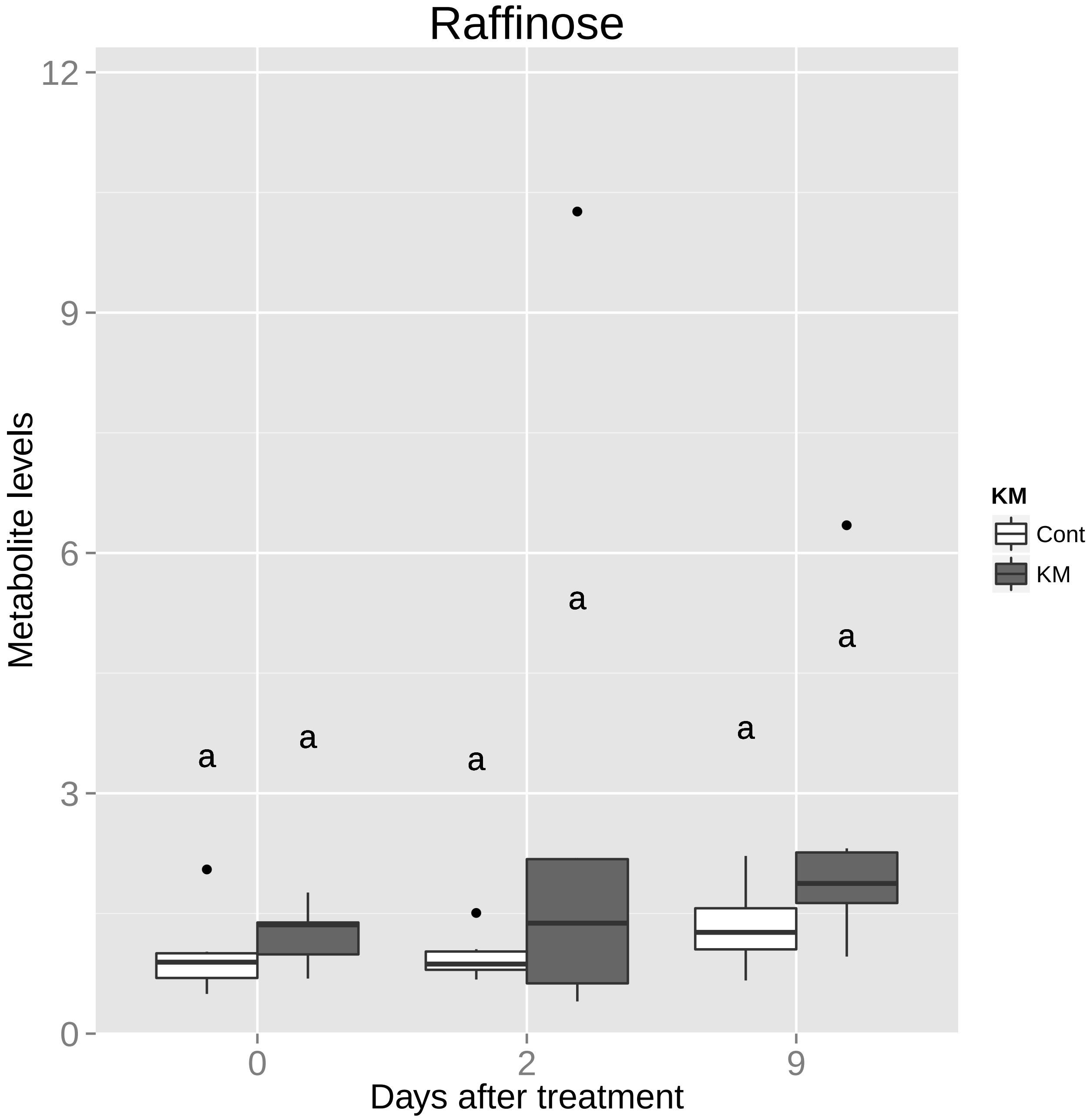
Raffinose
Metabolite levels
Days after treatment
0
3
6
9
12
0
2
9
a
a
a
a
a
a
KM
Cont
KM
